# Phylogenetic Evidence of Local HIV-1 Transmission and Antiretroviral Drug Resistance in the Middle East and North Africa

**DOI:** 10.3390/v18080897

**Published:** 2026-08-14

**Authors:** Esraa Al-Fraihat, Amal Irshaid, Mohammed Sallam, Johan Snygg, Rasha Awawdeh, Hasanain Al-Shakerchi, Sama Al-Baidhani, Malik Sallam

**Affiliations:** 1 Department of Pathology, Microbiology and Forensic Medicine, School of Medicine, The University of Jordan, Amman 11942, Jordan; 2 Department of Clinical Laboratories and Forensic Medicine, Jordan University Hospital, Amman 11942, Jordan; 3 Department of Pharmacy, Mediclinic Parkview Hospital, Mediclinic Middle East, Dubai P.O. Box 505004, United Arab Emirates; 4 Department of Management, Mediclinic Parkview Hospital, Mediclinic Middle East, Dubai P.O. Box 505004, United Arab Emirates; 5 College of Medicine, Mohammed Bin Rashid University of Medicine and Health Sciences (MBRU), Dubai P.O. Box 505055, United Arab Emirates; 6 Department of Management, School of Business, International American University, Los Angeles, CA 90010, USA; 7 Department of Pharmacology and Therapeutics, College of Medicine and Health Sciences, United Arab Emirates University (UAEU), Al Ain P.O. Box 17666, United Arab Emirates; 8 Department of Management, Mediclinic City Hospital, Mediclinic Middle East, Dubai P.O. Box 505004, United Arab Emirates; 9 Department of Anesthesia and Intensive Care, Sahlgrenska Academy, University of Gothenburg, 41345 Gothenburg, Sweden; 10 Department of Internal Medicine, Mediclinic Parkview Hospital, Mediclinic Middle East, Dubai P.O. Box 505004, United Arab Emirates

**Keywords:** HIV, Arab, molecular epidemiology, phylogenetic clustering, transmission networks, antiretroviral therapy, genetic diversity

## Abstract

The molecular epidemiology and antiretroviral (ARV) drug resistance of human immunodeficiency virus type 1 (HIV-1) remain incompletely outlined in the Middle East and North Africa (MENA). The aim of this retrospective molecular epidemiology study was to analyze MENA HIV-1 sequences for phylogenetic clustering and to delineate surveillance drug-resistance mutations (SDRMs) for nucleoside reverse-transcriptase inhibitors (NRTIs), non-nucleoside reverse-transcriptase inhibitors (NNRTIs), and protease inhibitors (PIs) across various periods, locations, and subtypes/circulating recombinant forms (CRFs). Viral sequences were retrieved from the Los Alamos HIV Sequence Database as of 15 April 2026. Analyses were done using multiple sub-gene regions (two *env* regions (*n* = 224 and *n* = 60) and *PR* (*n* = 2413) and *RT* (*n* = 2103) of the *pol* gene). Phylogeny construction was conducted using maximum-likelihood estimation, while ARV drug resistance analysis was conducted using the Stanford HIVdb algorithm. The HIV-1 MENA sequences showed a remarkable genetic diversity, with co-circulation of multiple subtypes/CRFs, including subtype B in the Maghreb, Levant, and Egypt sub-regions, subtypes A1, G, CRF01_AE, and CRF02_AG in the Gulf Cooperation Council (GCC) and Yemen sub-region, and subtypes C and D in the Horn of Africa and Sudan sub-region. The percentage of MENA HIV-1 sequences in clusters was 10.3% for *env1*, 8.3% for *env2*, 22.0% for *PR* and 37.2% for *RT*. Phylogenetic reconstruction hinted at a structured epidemic dominated by small transmission units, with most clusters comprising dyads (*n* = 260) or networks (*n* = 142) and a limited number of large clusters (*n* = 8) that were largely confined within national boundaries, with only occasional cross-border linkages (*n* = 8). Overall SDRM prevalence was 3.2% in the *PR* region and 14.9% in the *RT* region, with a higher percentage of NNRTI-associated mutations (10.0%) than NRTI-associated mutations (9.1%) and dual-class resistance observed in 4.1% of sequences. Phylogenetic clustering was not associated with the probability of harboring SDRMs; however, negative binomial models showed that non-clustered sequences had a greater burden of NRTI-associated mutations, whereas no such association was observed for NNRTI- or PI-associated mutations. The findings showed predominantly localized and fragmented MENA HIV-1 transmission dynamics. Heterogeneous ARV drug resistance dynamics indicated that resistance emergence might be shaped by broader epidemiologic and treatment-related factors rather than ongoing clustered transmission. There is a need for coordinated molecular surveillance and optimized ART strategies across the MENA countries.

## 1. Introduction

The human immunodeficiency virus type 1 (HIV-1) epidemic in the Middle East and North Africa (MENA) region is unique in comparison to the other global regions. Several distinctive features of the HIV-1 MENA epidemic contribute to this uniqueness, as follows. First, the HIV-1 MENA epidemic was described by the Joint United Nations Programme on HIV/AIDS (UNAIDS) as a “very low-prevalence” epidemic as of July 2025 [1]. Specifically, the most updated UNAIDS estimates pointed to HIV-1 prevalence among adults aged 15–49 years in the MENA at a median of 0.07% (0.06–0.10%) [1]. However, this figure might mask the fragmented hyper-epidemics among at-risk groups in the MENA region. These groups include female sex workers (FSWs) [2,3], men who have sex with men (MSM) [4,5], injection drug users (IDUs) [6], and prisoners [7]. Consequently, the HIV-1 epidemic in the MENA can be more accurately described as a “concentrated” epidemic. This concentrated epidemic nature is reflected in the substantially higher HIV-1 prevalence reported among these at-risk groups, reaching 8.2% among MSM, 7.1% among IDUs, 1.6% among FSWs, and 0.8% among individuals in prisons and other closed settings [1].

Second, the MENA region is one of two global regions in which HIV-1 incidence continues to rise. According to the Global Burden of Disease (GBD) study (1990–2021), new HIV-1 infections increased in the MENA by 66% and also increased in Central and Eastern Europe and Central Asia by 113% [8]. Additionally, the UNAIDS 2025 report indicated that the annual number of new HIV-1 infections in the MENA increased by 94% between 2010 and 2024, rising from 12,000 to 23,000 cases [1]. Importantly, these figures likely underestimate the true HIV-1 MENA burden. The underestimation of HIV-1 burden in the MENA region appears a plausible assumption given the challenges in HIV-1 case detection and reporting [9,10]. Consistent with the underestimation hypothesis, the UNAIDS 2025 report estimates that only 63% (44–85%) of people living with HIV/AIDS (PLWHA) in the MENA region are aware of their infection, which is indicative of a considerable gap in diagnosis and surveillance in the region [1]. Projections further suggested an unfavorable MENA HIV-1 epidemic trajectory based on the 2023 study by Khorrami et al., which estimated that both HIV-1 incidence and mortality are likely to increase across several MENA countries by 2030 [11].

A third notable feature of the MENA HIV-1 epidemic is the persistently low coverage of antiretroviral therapy (ART), which remains the lowest of any global region based on the latest UNAIDS regional report [1]. Specifically, it is estimated that only 35% (24–47%) of PLWHA in the MENA region are receiving ART, with particularly low coverage among children, at 44% (35–56%) [1]. Accordingly, the well-established benefits of ART including improved prognosis, improved quality of life (QoL), and reduced transmission at the population level through Treatment as Prevention (TasP) are not fully achieved in the MENA region [12,13,14,15,16]. Importantly, the reduced ART coverage and incomplete viral suppression at the population level create conditions that facilitate onward HIV-1 transmission and possible dissemination of drug-resistant lineages [17,18].

Fourth, the epidemiologic characterization of HIV-1 in the MENA region is challenged by documented limitations in data availability and quality [9,16,19]. Surveillance of HIV/AIDS in the MENA remains incomplete, with inconsistent case detection, limited follow-up, and insufficient inclusion of at-risk groups in HIV-1 care models by health authorities [16,20,21]. In addition, the marked heterogeneity in diagnostic capacity and reporting practices across the MENA countries further undermines the accuracy of HIV/AIDS epidemiologic parameter estimates, which should therefore be interpreted as conservative approximations of a partially observed epidemic [16,22,23].

Underestimation of HIV/AIDS in the MENA is closely linked to socio-cultural and political barriers that impede testing and status disclosure [24,25,26,27]. Persistent stigma toward PLWHA even among health professionals responsible for their care, along with legal and policy constraints in some MENA settings, limits engagement with health services and delays diagnosis, particularly among at-risk groups [28,29,30,31,32]. A systematic review by Mumtaz et al. highlighted substantial gaps in HIV-1 knowledge and widespread discriminatory attitudes across both general and at-risk groups [33]. These factors reduce engagement in testing and preventive measures while promoting concealment. In turn, this leads to obscuring of the true scale and structure of the HIV-1 epidemic in the MENA as reviewed separately by Kteily-Hawa et al. and Mahajan et al. [29,34].

At the individual level, the psycho-social burden of HIV-1 infection in the MENA further undermines engagement in health care [29,33,35,36]. This burden includes anxiety, depression, and fear of stigmatization with variable results reported in several national studies in Egypt, the United Arab Emirates (UAE), Oman, and Morocco [37,38,39,40]. Collectively, these factors have been shown to contribute to delayed HIV-1 infection diagnosis, incomplete ART uptake, and onward virus transmission in the region [41,42,43]. These dynamics contribute to the invisible nature of the HIV-1 epidemic in the MENA region, which is further complicated by the recent shifts in regional coordination and support structures [44]. In this context, molecular epidemiologic approaches can provide a critical means to infer transmission patterns and uncover otherwise unobserved links within partially characterized national HIV-1 epidemics in the MENA [45,46].

At this point, it is important to elaborate on a widely prevalent notion held in the MENA region among other Muslim-majority settings. This notion entails that HIV-1 epidemics in such settings are mainly exogenous, arising largely from repeated external introductions rather than sustained domestic transmission, creating a state of denial [47,48]. Specifically, this framing has influenced both risk perception and public health responses, which were often underpinned by assumptions that behaviors associated with HIV-1 acquisition are rare or socially constrained to collectively forbidden practices termed *haram* (e.g., premarital sex, adultery, prostitution, homosexuality, and IDU) as described by Kelley and Eberstadt [49]. Such assumptions may have contributed to delayed recognition of local HIV-1 transmission dynamics and hindered implementation of timely and effective intervention measures such as ART in the MENA [50,51].

The notion that HIV-1 acquisition in the MENA region is predominantly exogenous is, to some extent, fathomable, given the early epidemiologic observations suggesting multiple and diverse viral introductions into the region [52,53,54]. This interpretation was further supported by the extensive genetic diversity of HIV-1 in the MENA and may be sustained by social and cultural contexts that discourage attribution of HIV-1 transmission to local networks [49,52,55]. However, this notion was often based on incomplete HIV-1 datasets, limited sampling of key at-risk groups, and methodological constraints, which may have preferentially depicted signals of HIV-1 introduction while underestimating onward local transmission in the MENA [9,56,57].

More recent evidence challenges the existing view that HIV-1 infections in the MENA region are predominantly imported. Molecular studies with improved sampling and analytical resolution identified phylogenetic clustering within the MENA region at substantial percentages [55,58,59]. This was particularly evident among at-risk groups such as IDUs, MSM, and FSWs, which indicates shared ancestry over short time scales consistent with sustained domestic transmission [54]. In parallel, epidemiologic patterns—including the rising HIV-1 incidence, concentration of infection within at-risk groups, and persistent gaps in diagnosis—are difficult to reconcile with a model based solely on sporadic, unrelated HIV-1 importation events. Despite acknowledged limitations, a previous regional molecular epidemiology analysis reported that a substantial percentage (54%) of HIV-1 MENA sequences formed phylogenetic clusters, many confined within individual countries and others spanning national boundaries, across subtypes B, C, and circulating recombinant forms (CRFs) such as CRF02_AG and CRF06_cpx [55]. These findings suggest that, alongside repeated HIV-1 introductions, sustained transmission networks were established within and across countries in the MENA region [54,55].

Such findings showed the utility of phylogenetic approaches in HIV-1 epidemiologic research, which stem from key properties of the virus. HIV-1 evolves rapidly as a result of error-prone reverse transcription, high replication rates, and frequent recombination, generating substantial genetic diversity within and between hosts [46,60,61]. This diversity encodes a record of viral ancestry that when analyzed using robust phylogenetic methods enables reconstruction of relationships among infections and identification of sequence clusters sharing a recent common ancestor [60,62,63,64]. When supported by appropriate statistical criteria, phylogenetic clusters serve as proxies for transmission networks; although they do not establish direct epidemiologic linkage or directionality, their presence indicates that infections are not independent but occur within interconnected chains of transmission [64,65,66,67].

Despite this potential, the extent of phylogenetic clustering in the MENA region has been incompletely characterized across large, multi-country datasets. Previous studies, although limited in scope, have identified clusters consistent with local transmission within and across MENA countries [55,58,59]. However, in light of the evolving HIV-1 MENA sub-epidemics and the increasing availability of sequence data, a more comprehensive and systematic analysis is warranted. In parallel, the emergence and spread of antiretroviral (ARV) drug resistance in the MENA region represents an additional area of concern as reported in a recent systematic review by Khodadad et al. [68].

Thus, in the current study, we sought to reassess the molecular epidemiology of HIV-1 in the MENA region in the context of expanded molecular sequence availability in GenBank and the Los Alamos HIV database (LAHDB). Specifically, we aimed (1) to quantify the extent of phylogenetic clustering among HIV-1 sequences as a measure of non-random transmission structure consistent with local onward transmission; (2) to characterize the ARV drug resistance in the MENA region; and (3) to assess whether phylogenetic clustering in the MENA was associated with accumulation of surveillance drug-resistance mutations (SDRMs).

## 2. Materials and Methods

### 2.1. Study Design

This study was designed as a retrospective molecular epidemiologic analysis based on the publicly available MENA HIV-1 sequence data. Viral sequences were retrieved from the LAHDB (https://www.hiv.lanl.gov/, accessed on 25 April 2026) [69] and analyzed to characterize phylogenetic clustering patterns consistent with a local transmission structure and to assess the prevalence and temporal dynamics of ARV drug resistance across the MENA region. The LAHDB is a globally recognized public repository that curates HIV sequences and associated metadata from published studies and direct submissions. Standardized annotation, quality control (QC), and subtype/CRF assignment make it a key resource for HIV molecular epidemiology, phylogenetic analyses, and ARV drug resistance surveillance [69].

Sequences were included from countries defined as part of the MENA region, including Algeria, Bahrain, Djibouti, Egypt, Iraq, Jordan, Kuwait, Lebanon, Libya, Mauritania, Morocco, Oman, Palestine, Qatar, Kingdom of Saudi Arabia (KSA), Somalia, Sudan/South Sudan, Syria, Tunisia, the United Arab Emirates (UAE), and Yemen. The regional definition was based on the UNAIDS classification and previous molecular epidemiologic studies of infectious agents in the MENA region [1,9,54,55,70]. Although some regional frameworks may include Iran, Turkey, Pakistan, Afghanistan, or Israel, recognized epidemiologic characteristics distinguishing these HIV-1 epidemics from those observed across the core MENA region prompted us to adopt a more restrictive definition for the current study [71,72,73,74,75,76,77].

Only HIV-1 sequences with available metadata specifying country of origin and sampling year were considered for inclusion. Sequence pre-processing and QC were performed before downstream analyses and included removal of duplicate sequences and exclusion of sequences with extensive ambiguities or poor coverage. Data cleaning and preliminary processing were conducted using Molecular Evolutionary Genetics Analysis (MEGA) software (version 6.0) and FaBox online tools (version 1.61; https://users-birc.au.dk/~palle/php/fabox/index.php, accessed on 25 April 2026), followed by manual inspection and validation to create the final clean HIV-1 datasets [78,79].

### 2.2. MENA HIV-1 Sequence Retrieval and Selection

HIV-1 nucleotide sequences were retrieved using the advanced search interface of the LAHDB (https://www.hiv.lanl.gov/components/sequence/HIV/asearch/map_db.comp, accessed on 25 April 2026) [69]. Searches were restricted to HIV-1 sequences originating from countries in the MENA region, as defined above, and were filtered by accession number, country of origin, sampling year, and subtype/CRF (when available). Unique patient identifiers were used, when provided, to exclude multiple HIV-1 sequences derived from the same individual and to retain the earliest collected single representative sequence per infected individual. For each country, all available HIV-1 sequences meeting inclusion criteria were downloaded in FASTA format. The sequences were retrieved from the following cited sources per country: Algeria [80,81,82], Djibouti [83,84], Egypt [85,86], Iraq [87], Kuwait [88,89,90,91,92,93], Lebanon [94,95,96], Libya [97,98], Mauritania [99], Morocco [100,101,102,103,104], Oman [105], KSA [106,107,108], Somalia [109], Sudan and South Sudan [110,111,112], Tunisia [59,113], and Yemen [114], in addition to direct submissions that were not linked to publications at the time of sequence retrieval.

Sequences were mapped to the HXB2 genome (GenBank accession number: K03455), which is the HIV-1 reference sequence used for genome coordinate numbering and comparative sequence analyses for positional orientation [115]. Analyses were restricted to homologous genomic regions corresponding to the *pol* gene (HXB2 positions 2358–5096) and the *env* gene (HXB2 positions 6225–8795), which were selected because of their widespread availability and established utility in HIV-1 molecular epidemiologic and ARV drug resistance analyses [116,117].

### 2.3. Sequence Processing, Alignment, and Reference Dataset Construction

Molecular sequence processing and alignment were conducted using a stepwise approach to maximize alignment accuracy relative to the HXB2 reference genome. All sequences were first mapped to the HXB2 reference using the sequence locator tool available through the LAHDB (https://www.hiv.lanl.gov/content/sequence/LOCATE/locate.html, accessed on 25 April 2026) to determine genomic coordinates. Sequences corresponding to the predefined *pol* and *env* regions were subsequently extracted and subjected to multiple sequence alignment using the ClustalW algorithm implemented in MEGA software (version 6.0) [118,119,120,121]. Multiple sequence alignment (MSA) computationally aligns homologous nucleotide positions across multiple sequences, enabling the identification of conserved and variable sites required for accurate phylogenetic inference.

Alignments were manually inspected to remove poorly aligned regions, sequences with excessive gaps, and sequences with high levels of ambiguity to minimize artifacts that could bias phylogenetic inference. To ensure that the included HIV-1 sequences represented viable viruses, nucleotide sequences were translated into amino acid sequences, and those containing disrupted open reading frames (ORFs) were excluded (e.g., those with stop codons). Sequences with incomplete coverage of the target regions or excessive ambiguous nucleotides (≥10 ambiguous nucleotides) were also excluded. Sequences from Libya were excluded from further analyses because they were associated with a well-documented nosocomial outbreak [97,98].

To provide accurate subtype/CRF assignment, a reference dataset of complete HIV-1 group M genomes, including major subtypes and CRFs, was retrieved from the LAHDB (https://www.hiv.lanl.gov/content/sequence/NEWALIGN/align.html, accessed on 25 April 2026). A total of 30 reference HIV-1 sequences were selected from this dataset based on availability up to 2022. These reference sequences were incorporated into the MENA HIV-1-aligned datasets to enable phylogenetic subtype/CRF classification, which is the gold-standard classification approach for HIV-1 lineages (Table A1, Figure S1) [122,123].

### 2.4. Construction of Genomic-Region-Specific HIV-1 Datasets and Phylogenetic Subtyping

To conduct sub-gene region-specific phylogenetic analyses and to assess the phylogenetic clustering across genomic regions with differing evolutionary characteristics, four independent datasets were constructed corresponding to distinct regions of the HIV-1 genome. These included two envelope (*env*) regions and two polymerase (*pol*) sub-regions: protease (*PR*) and reverse transcriptase (*RT*). The *env* datasets comprised (1) HXB2 positions 7767–8350 and (2) HXB2 positions 7071–7283. The *pol* datasets included the *PR* region (HXB2 positions 2253–2549) and the *RT* region (HXB2 positions 2550–3300), with minor boundary adjustments applied where necessary to maximize sequence inclusion across MENA countries while preserving positional homology.

For each genomic region, HIV-1 sequences were extracted according to HXB2 coordinates following mapping and alignment, and only those fully covering the specified regions and meeting QC criteria were retained. Subtype/CRF assignment was performed using maximum-likelihood (ML) phylogenetic inference in PhyML (version 3.0) under a general time-reversible (GTR) nucleotide substitution model with gamma-distributed rate heterogeneity across sites and a proportion of invariant sites (GTR + Γ + I) [124,125], incorporating the curated reference panel of 30 HIV-1 group M sequences representing the major subtypes/CRFs reported in the MENA [52,55]. Accession numbers for all reference sequences are provided in Table A1.

### 2.5. Phylogenetic Analysis and Definition of Clustering

Identification of putative MENA phylogenetic clusters representing groups of genetically related HIV-1 sequences consistent with local onward transmission, rather than direct transmission networks, was performed using a conservative two-step framework integrating phylogenetic support and genetic proximity. Maximum-likelihood phylogenetic trees were reconstructed using PhyML (version 3.0) under a GTR nucleotide substitution model with gamma-distributed rate heterogeneity across sites and a proportion of invariant sites (GTR + Γ + I) [124,125]. Rate variation was modeled using a discrete gamma distribution with four categories. Model parameters, including base frequencies, substitution rates, gamma shape parameters, and the proportion of invariant sites, were estimated directly from the data during tree optimization.

Branch support was assessed using the Shimodaira–Hasegawa-like approximate likelihood ratio test (aLRT-SH) [126]. Phylogenetic trees were exported in a FigTree-compatible format (FigTree v.1.4.4.) [127], in which branch support values were encoded as annotations. To enable downstream analysis, tree files were converted to standard Newick format by replacing FigTree-style annotations with internal node labels.

Cluster inference analyses were conducted in R (version 4.2.3; R Foundation for Statistical Computing; Vienna, Austria) and RStudio (version 2025.05.1; Posit Software; Boston, MA, USA) using the Analysis of Phylogenetics and Evolution (APE) package (version 5.8-1) [128]. Candidate clusters were defined as monophyletic clades with topological support (aLRT-SH ≥ 0.90) based on the previous work on the definition of phylogenetic clusters in HIV-1 epidemics [55,61,129]. For each supported clade, all descendant HIV-1 sequences were identified, and pairwise patristic distances were calculated from the phylogenetic tree. Distances were expressed as substitutions per site (s/s), consistent with branch-length scaling in PhyML. A clade was retained as a molecular cluster only if it satisfied both criteria: (1) robust branch support (aLRT-SH ≥ 0.90) and (2) genetic proximity, defined as a maximum pairwise patristic distance of ≤0.045 s/s among all sequences within the clade [61]. To avoid redundant nesting, only the largest non-overlapping clades meeting these criteria were retained. For each cluster, summary metrics—including cluster size (dyad = 2, network = 3–14 and large ≥ 15), maximum within-cluster genetic distance, branch support, and sequence composition—were recorded [55,61,130]. The percentage of clustered MENA HIV-1 sequences was calculated as the number of unique sequences belonging to at least one cluster divided by the total number of sequences in the dataset. Clusters were interpreted as groups of sequences with non-random genetic relatedness consistent with shared transmission networks or local onward spread; however, no inference was made regarding direct epidemiologic linkage or transmission directionality.

### 2.6. Antiretroviral Drug Resistance Analysis

Resistance against ARV drugs was assessed for sequences spanning the *PR* and *RT* regions of the HIV-1 *pol* gene. Nucleotide sequences were analyzed using the Stanford HIVdb algorithm (Sierra pipeline, version 3.5.5; HIVdb version 10.1, https://hivdb.stanford.edu/cpr/, accessed 25 April 2026) [131,132]. This approach was used to identify resistance-associated mutations, including nucleoside reverse-transcriptase inhibitors (NRTIs), non-nucleoside reverse-transcriptase inhibitors (NNRTIs), and protease inhibitors (PIs). Surveillance drug-resistance mutations (SDRMs) were identified using the calibrated population resistance (CPR) tool (Version 8.1) within the Stanford HIVdb framework for the standardized assessment of population-level resistance based on internationally recognized mutation lists [131,133,134]. Resistance prevalence was calculated for each ART drug class and stratified by the sampling period to assess temporal patterns. Because treatment history was not uniformly available, resistance patterns were interpreted at the population level and were not classified as transmitted or acquired resistance at the individual level.

### 2.7. Statistical and Data Analysis

Statistical analyses were conducted to characterize the distribution and determinants of phylogenetic clustering and ARV drug resistance. To account for regional heterogeneity, countries were grouped into four epidemiologically informed sub-regions: Gulf Cooperation Council (GCC) countries and the Yemen sub-region (KSA, Kuwait, Oman, and Yemen); the Maghreb and Mauritania (Morocco, Algeria, Tunisia, and Mauritania); the Levant and Egypt (Egypt, Lebanon, and Iraq); and the Horn of Africa and Sudan (Somalia, Djibouti, and Sudan/South Sudan).

Time of sequence collection was categorized into periods reflecting major phases of ART scale-up (≤2004, 2005–2012, 2013–2019, and 2020–2025), informed by global guideline changes and regional ART expansion [16,26,135,136,137,138,139]. Subtypes/CRFs were dichotomized as B versus non-B to assess the influence of major HIV-1 lineage structure. Given the disproportionate contribution and distinct subtype distribution of sequences from Oman, analyses were repeated after excluding Oman to determine whether observed patterns reflected regional trends or country-specific effects.

Chi-square (χ^2^) tests were used to compare categorical variables, and a linear-by-linear (LBL) test for association was applied to assess ordered changes in proportions across temporal categories. Associations between phylogenetic clustering and SDRMs were evaluated using mixed-effects logistic regression models, with SDRM status (yes vs. no) as the dependent variable and clustering status as the primary exposure. Separate models were fitted for NRTI, NNRTI, and PI resistance. Models included random effects for the time period, sub-region, and subtype/CRF to account for unequal sampling and unobserved heterogeneity. Odds ratios (ORs) and 95% confidence intervals (CIs) were derived from model coefficients.

To assess whether clustering was associated with the burden of resistance mutations, the number of SDRMs per sequence was modeled using negative binomial regression with a log-link function, selected a priori to account for overdispersion. Models were adjusted for time period, sub-region, and subtype/CRF. Incidence rate ratios (IRRs) and 95% CIs were calculated by exponentiating regression coefficients.

All analyses were performed using R (version 4.2.3; R Foundation for Statistical Computing; Vienna, Austria) and RStudio (version 2025.05.1; Posit Software; Boston, MA, USA), with supplementary analyses conducted in IBM SPSS Statistics for Windows, Version 26.0. Armonk, NY: IBM Corp. All statistical tests were two-sided, and *p* values of less than 0.050 were considered to indicate statistical significance.

## 3. Results

### 3.1. Description of HIV-1 MENA Dataset

**Figure 1:**
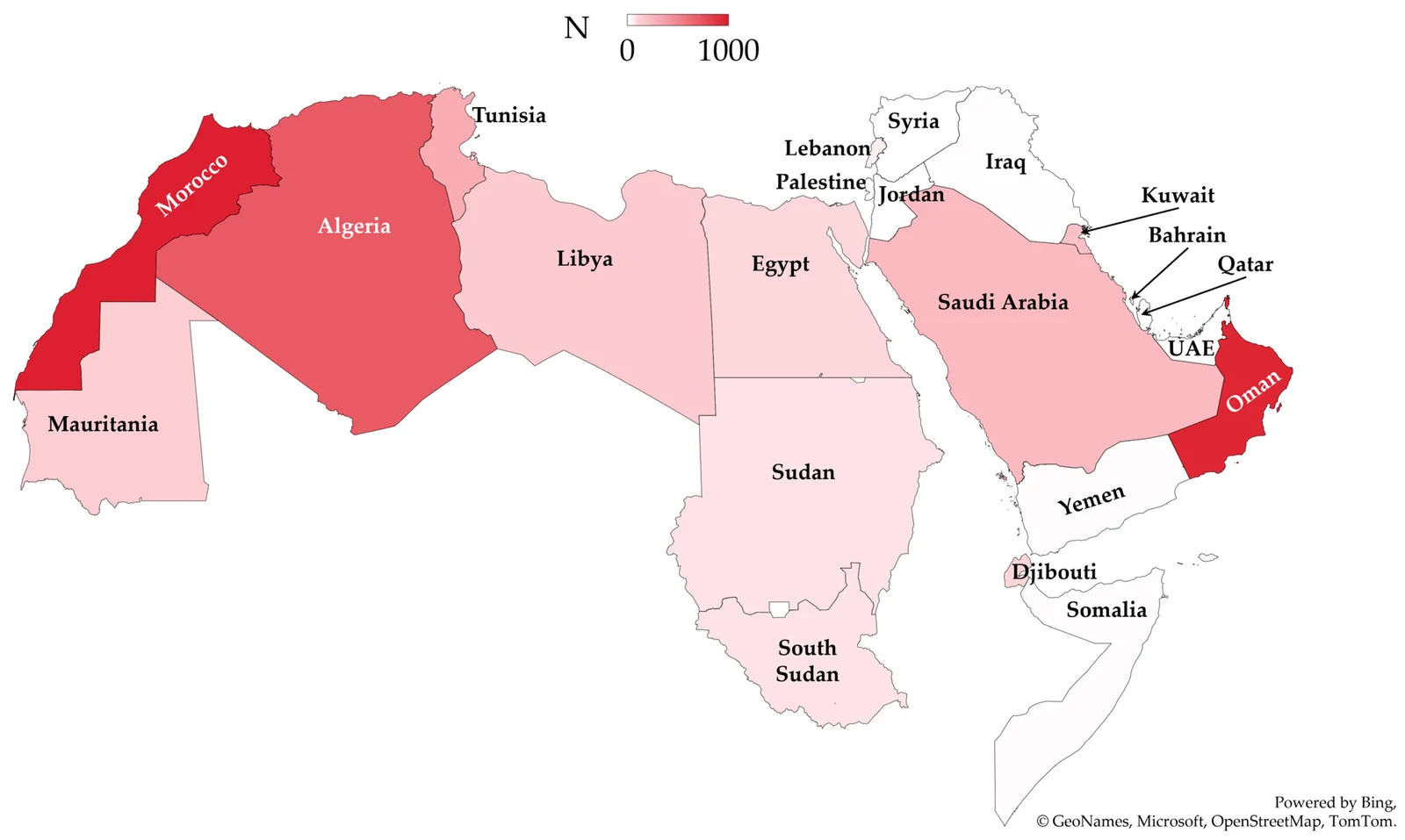
Geographic distribution of HIV-1 sequences from the Middle East and North Africa (MENA) region. Shading intensity reflects the relative number of HIV-1 sequences contributed by each country, with darker shading indicating higher sequence counts. The map was generated in Microsoft Excel, powered by Bing, © GeoNames, Microsoft, OpenStreetMap, TomTom. We are neutral with regard to jurisdictional claims in this map.

The initial retrieved dataset comprised a total of 4024 HIV-1 sequences from countries in the MENA, with substantial heterogeneity in geographic representation. The dataset showed a marked imbalance in sampling across the MENA region, with a disproportionate contribution from a limited number of countries, particularly in the Maghreb and a few GCC countries. As shown in Figure 1, the largest contributions were from Morocco (*n* = 968), Oman (*n* = 950), and Algeria (*n* = 709), followed by Tunisia (*n* = 297), KSA (*n* = 226), and Kuwait (*n* = 191). Additional sequences were obtained from Mauritania (*n* = 127), Djibouti (*n* = 99), Egypt (*n* = 97), Sudan/South Sudan (*n* = 81), and Lebanon (*n* = 60), whereas only a limited number were available from Somalia (*n* = 26), Yemen (*n* = 25), and Iraq (*n* = 20). No sequences were found for Bahrain, Jordan, Palestine, Qatar, Syria, or the UAE (Figure 1).

The aforementioned counts represented the unfiltered MENA HIV-1 LAHDB sequence pool and might include multiple sequences derived from the same individual, as well as sequences containing ambiguous nucleotides and premature stop codons, which were addressed in subsequent QC procedures.

### 3.2. HIV-1 Subtype/CRF Distribution per Country

After QC, four genomic datasets were constructed for analysis. The first *env* datasets comprised 224 sequences (nucleotide position relative to HXB2 genome start: 7071–7283) and the second *env* datasets comprised 60 sequences (nucleotide position relative to HXB2 genome start: 7767–8342). The *pol* datasets included 2413 sequences for the *PR* region and 2103 sequences for the *RT* region. Sequences from Libya were excluded because they were derived exclusively from a well-characterized nosocomial outbreak involving CRF02_AG, which would disproportionately bias subtype/CRF distribution and clustering analyses.

Across the four genomic regions, substantial heterogeneity in subtype/CRF distribution was observed between the MENA countries (Figure S1). In the first *env* dataset, subtype B predominated in the Maghreb countries, including Algeria (64.8%), Morocco (72.7%), and Tunisia (95.0%), whereas more diverse patterns were observed in Lebanon despite the common occurrence of subtype B. In the second *env* dataset, an exclusive presence of subtype A1 was found in Iraq (100%), whereas subtype C predominated in KSA (43.9%), with additional representation of subtype B and other subtypes/CRFs (Table 1).

In the *PR* dataset, marked geographic variation was also found. Subtype B was the most common subtype in Morocco (61.6%), Lebanon (44.0%), and Tunisia (38.7%), whereas CRF02_AG predominated in Mauritania (70.1%) and was also common in Tunisia (46.5%) and Morocco (25.2%). Oman exhibited a distinct subtype/CRF profile, with a high prevalence of subtype G (39.3%) and subtype A1 (30.0%), along with contributions from CRF01_AE and other lineages. In Kuwait, CRF01_AE was the most common (34.3%), while in KSA subtype C was the most common (60.3%), with additional representation of CRF01_AE and subtype B. Similar patterns were observed in the *RT* region, with subtype B predominating in Morocco (77.8%) and Tunisia (38.4%), and CRF02_AG remaining the dominant lineage in Mauritania (75.3%). Oman again demonstrated a distinct distribution characterized by subtype G (38.6%) and subtype A1 (30.3%). Subtype C was predominant in KSA (60.3%) and Sudan/South Sudan (38.7%), whereas Egypt showed a predominance of CRF02_AG (62.5%, Table 1).

In the *PR* dataset, the distribution of subtype B versus non-B subtypes/CRFs varied significantly across time periods and geographic regions (*p* < 0.001 for both). Subtype B increased from 40.7% before 2005 to 56.4% during 2005–2012, followed by a marked decline to 22.7% in 2013–2019 and 4.3% since 2020 (*p* < 0.001 for trend using LBL), with a corresponding increase in non-B subtypes/CRFs to 95.7% in the most recent period. Subtype B was concentrated in the Maghreb and Mauritania (44.3%) and was uncommon in the GCC countries and Yemen (5.8%) and in the Horn of Africa and Sudan (2.1%). After exclusion of Oman, these temporal patterns remained significant but were attenuated, with subtype B declining to 18.8% in the most recent period; geographic differences persisted, although the regional trend was no longer significant (*p* = 0.430 for trend using LBL). Similar patterns were observed in the *RT* dataset. Subtype B increased from 47.7% before 2005 to 55.2% during 2005–2012, followed by a decline to 25.2% in 2013–2019 and 5.1% since 2020 (*p* < 0.001 for trend using LBL). Subtype B remained concentrated in the Maghreb and Mauritania (46.5%) and was infrequent in the GCC countries and Yemen (5.6%) and in the Horn of Africa and Sudan (3.1%). After exclusion of Oman, the temporal decline persisted but was attenuated (16.0% in ≥2020), and although geographic differences remained significant, the regional trend was not (*p* = 0.684 for trend using LBL, Table 2).

### 3.3. Phylogenetic Clustering Across Genomic Regions and HIV-1 Subtypes/CRFs

**Figure 2:**
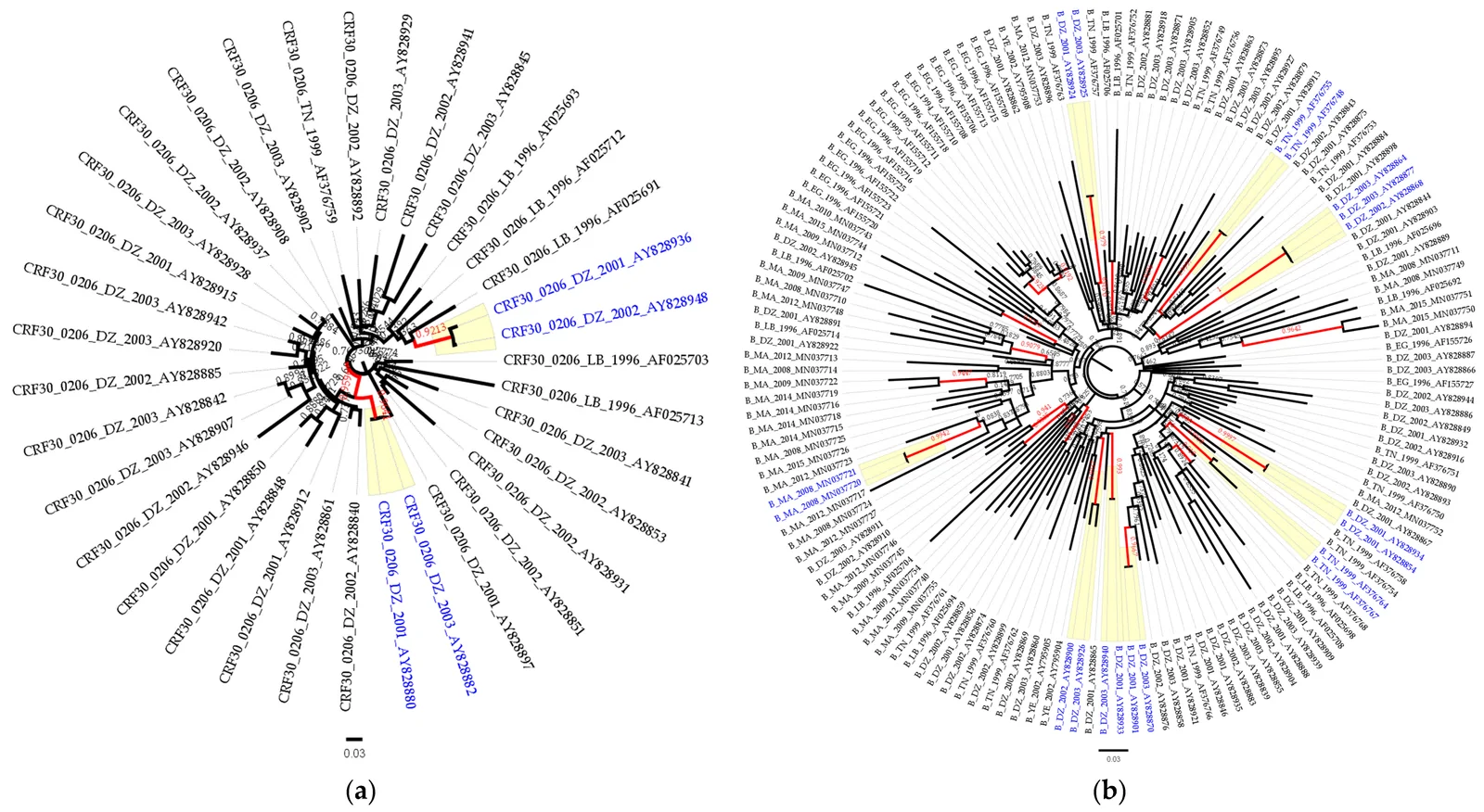
Maximum-likelihood phylogenetic trees of the first Middle East and North Africa (MENA) HIV-1 *env* region (nucleotide position relative to HXB2 genome start: 7071–7283): (**a**) CRF30_0206 tree; (**b**) subtype B tree. Trees were visualized using FigTree v.1.4.4. (https://tree.bio.ed.ac.uk/software/figtree/, accessed 4 May 2026) and midpoint-rooted. Sequence names belonging to phylogenetic clusters are highlighted in blue, and branches with strong statistical support (Shimodaira–Hasegawa-like approximate likelihood ratio test (aLRT-SH) ≥ 0.90) are indicated in red. Phylogenetic clusters denoting statistically supported monophyletic clades with a maximum pairwise genetic distance of ≤0.045 substitutions/site are further highlighted in yellow boxes. Scale bars are shown below each tree. The original tree files (.tre) are supplied in Supplementary File S2.

**Figure 3:**
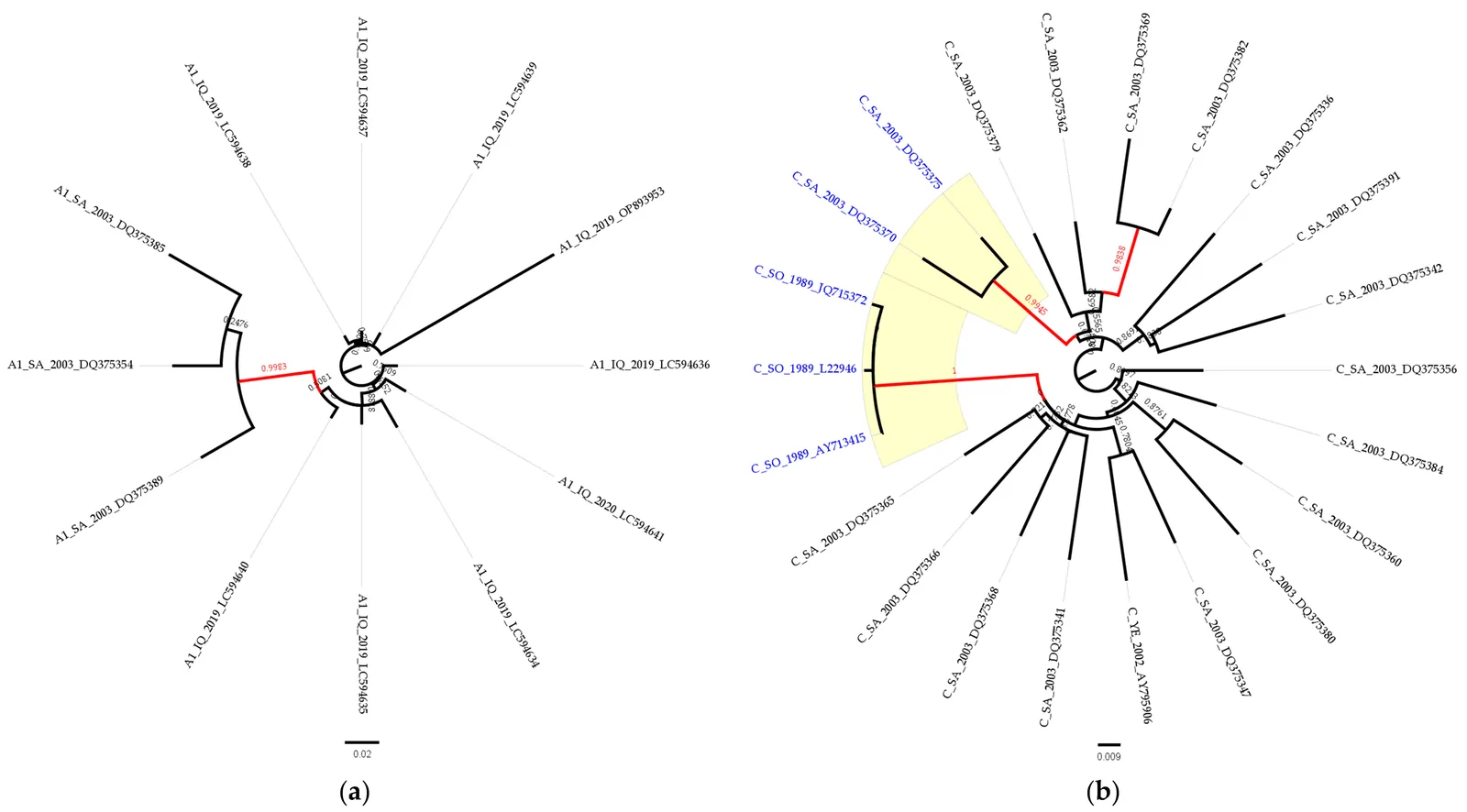
Maximum-likelihood phylogenetic trees of the second Middle East and North Africa (MENA) HIV-1 *env* region (nucleotide position relative to HXB2 genome start: 7767-8342): (**a**) subtype A1 tree; (**b**) subtype C tree. Trees were visualized using FigTree v.1.4.4. (https://tree.bio.ed.ac.uk/software/figtree/, accessed 4 May 2026) and midpoint-rooted. Sequence names belonging to phylogenetic clusters are highlighted in blue, and branches with strong statistical support (Shimodaira–Hasegawa-like approximate likelihood ratio test (aLRT-SH) ≥0.90) are indicated in red. Phylogenetic clusters denoting statistically supported monophyletic clades with a maximum pairwise genetic distance of ≤0.045 substitutions/site are further highlighted in yellow boxes. Scale bars are shown below each tree. The original tree files (.tre) are supplied in Supplementary File S2.

**Figure 4:**
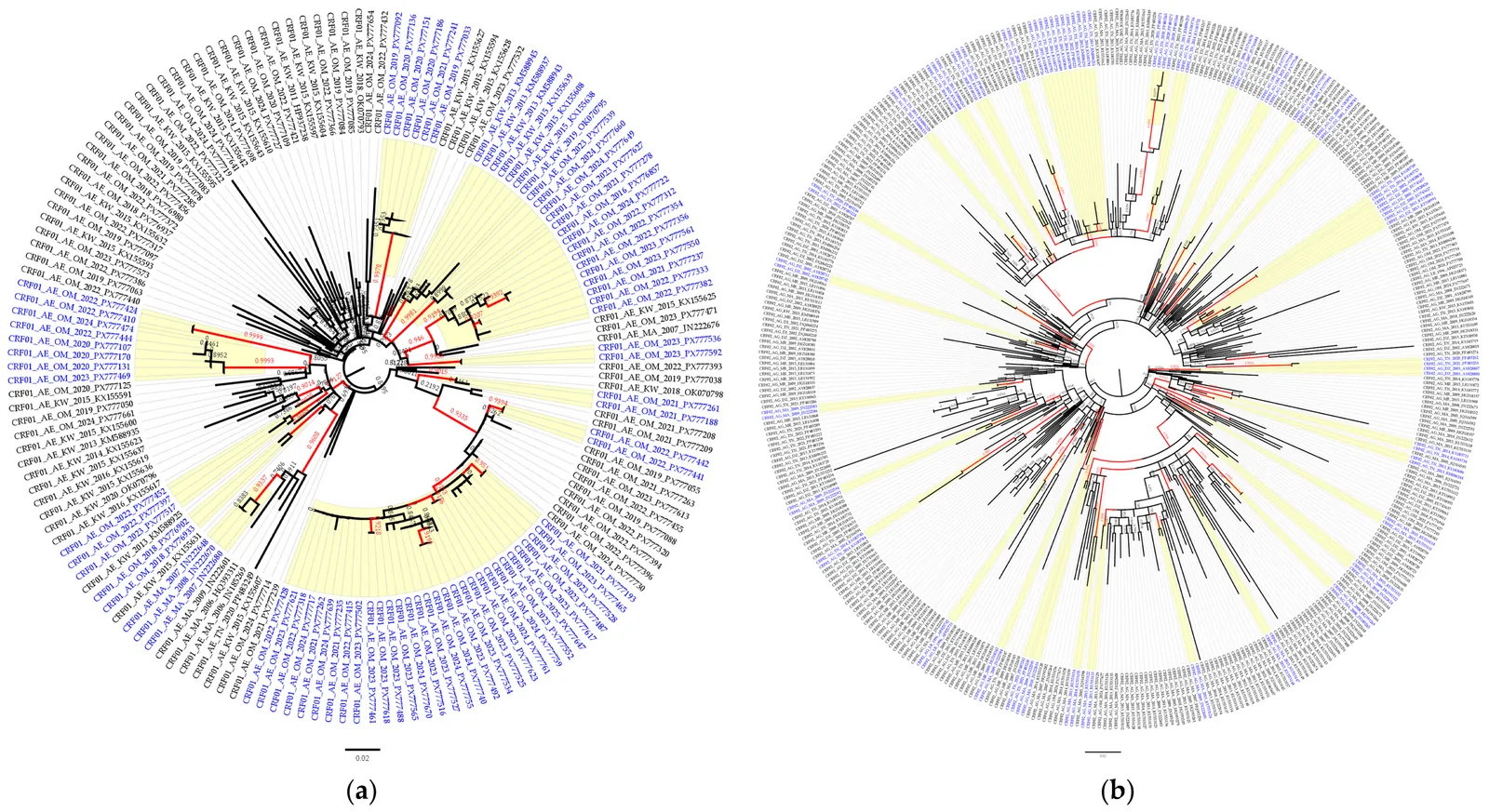

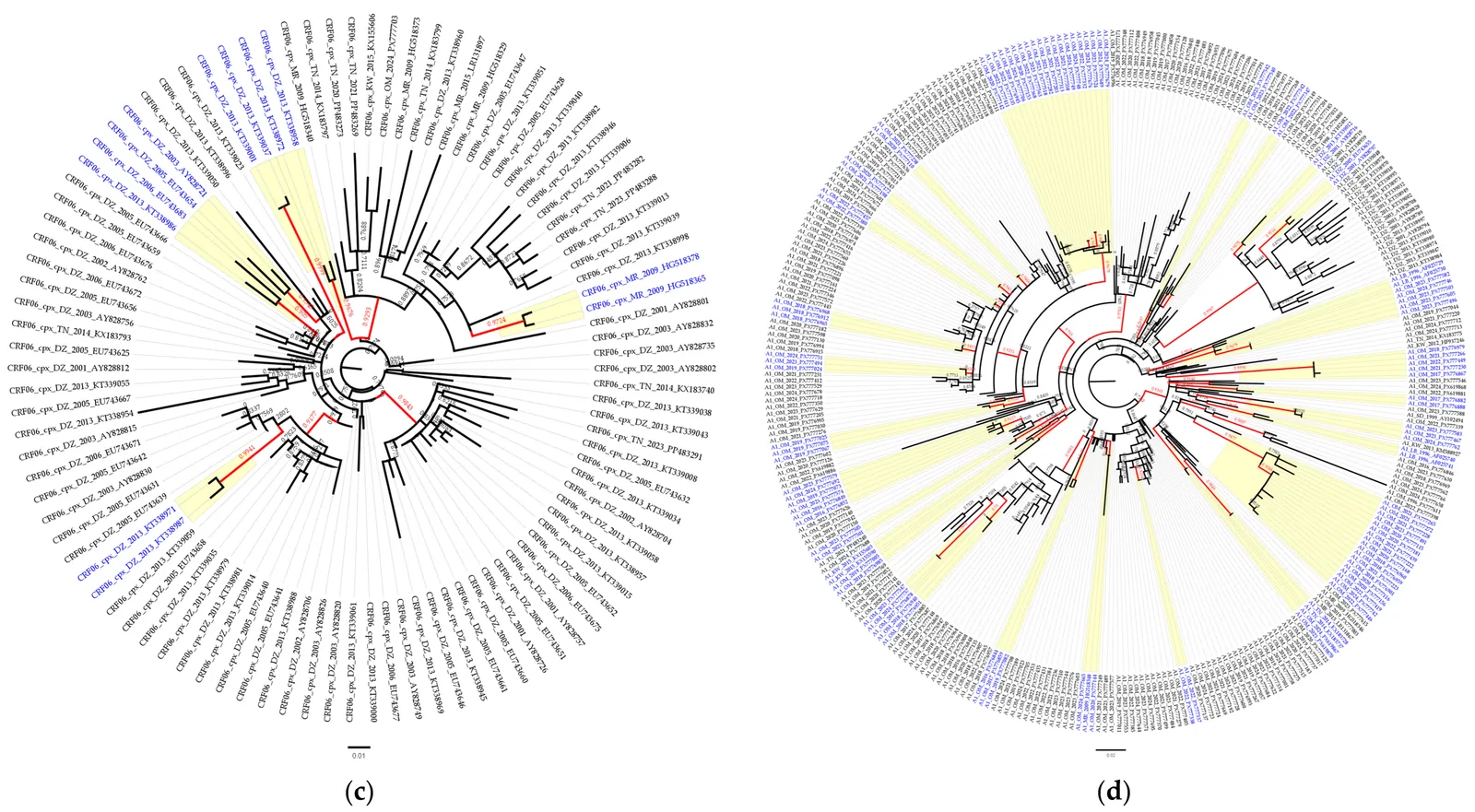

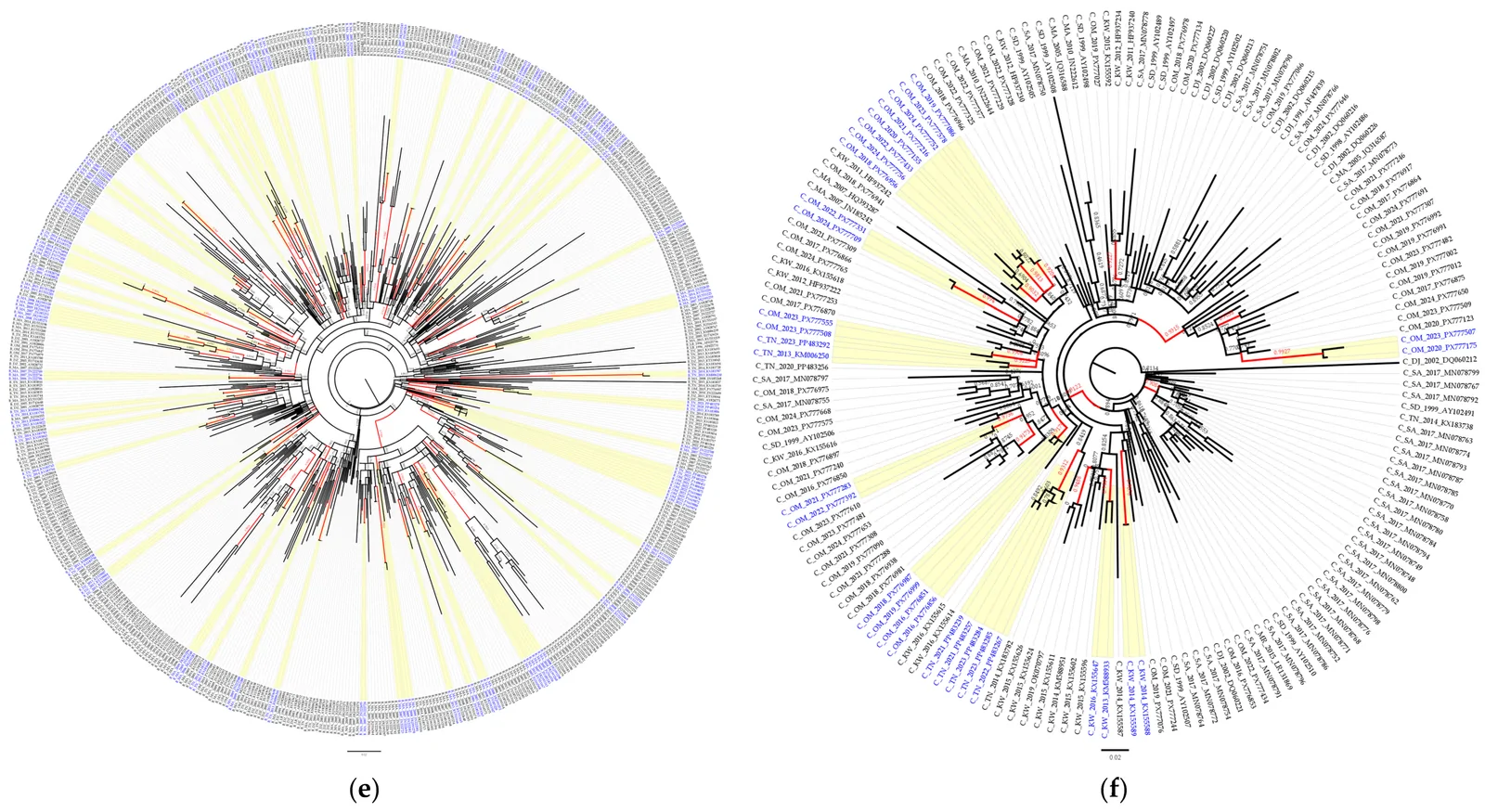

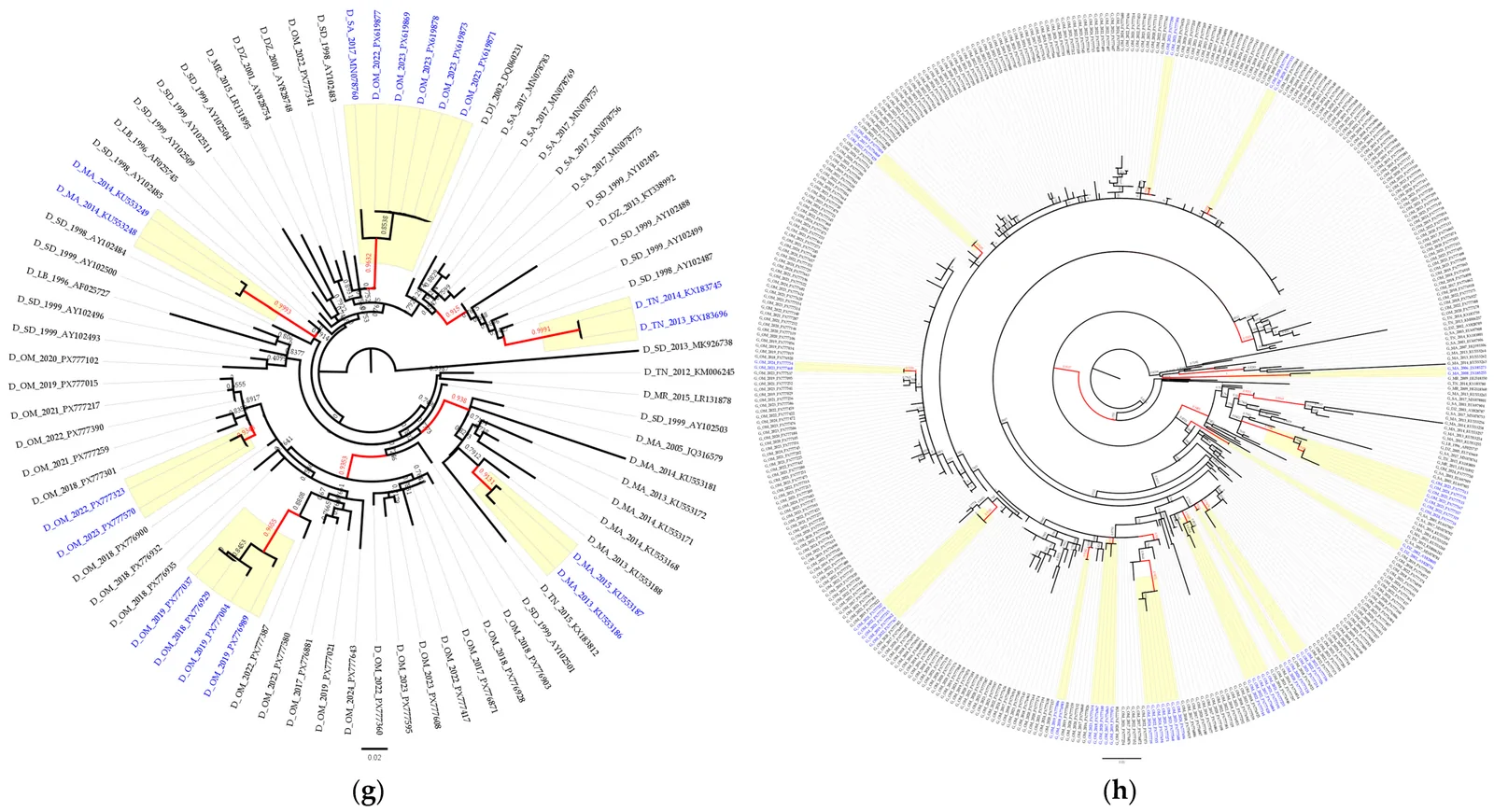
Maximum-likelihood phylogenetic trees of the Middle East and North Africa (MENA) HIV-1 *PR* region: (**a**) CRF01_AE tree; (**b**) CRF02_AG tree; (**c**) CRF06_cpx tree; (**d**) subtype A1 tree; (**e**) subtype B tree; (**f**) subtype C tree; (**g**) subtype D tree; (**h**) subtype G tree. Trees were visualized using FigTree v.1.4.4. (https://tree.bio.ed.ac.uk/software/figtree/, accessed 4 May 2026) and midpoint-rooted. Sequence names belonging to phylogenetic clusters are highlighted in blue, and branches with strong statistical support (Shimodaira–Hasegawa-like approximate likelihood ratio test (aLRT-SH) ≥0.90) are indicated in red. Phylogenetic clusters denoting statistically supported monophyletic clades with a maximum pairwise genetic distance of ≤0.045 substitutions/site are further highlighted in yellow boxes. Scale bars are shown below each tree. The original tree files (.tre) are supplied in Supplementary File S2.

**Figure 5:**
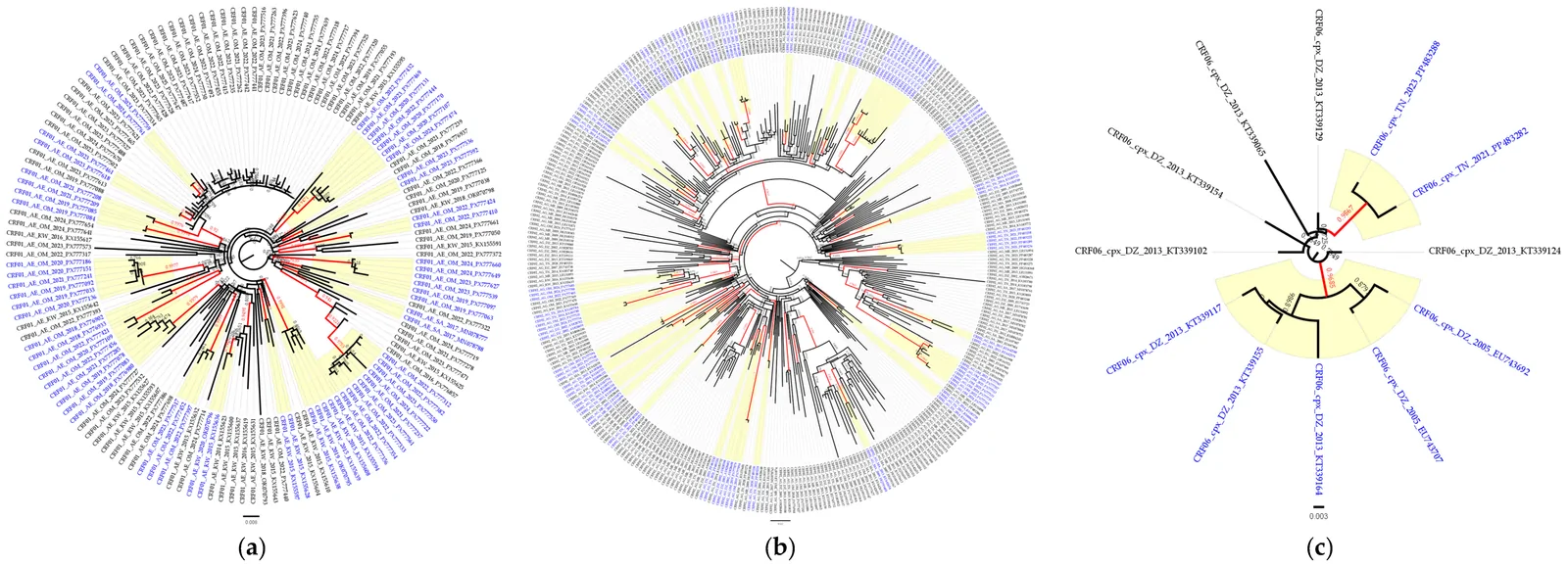

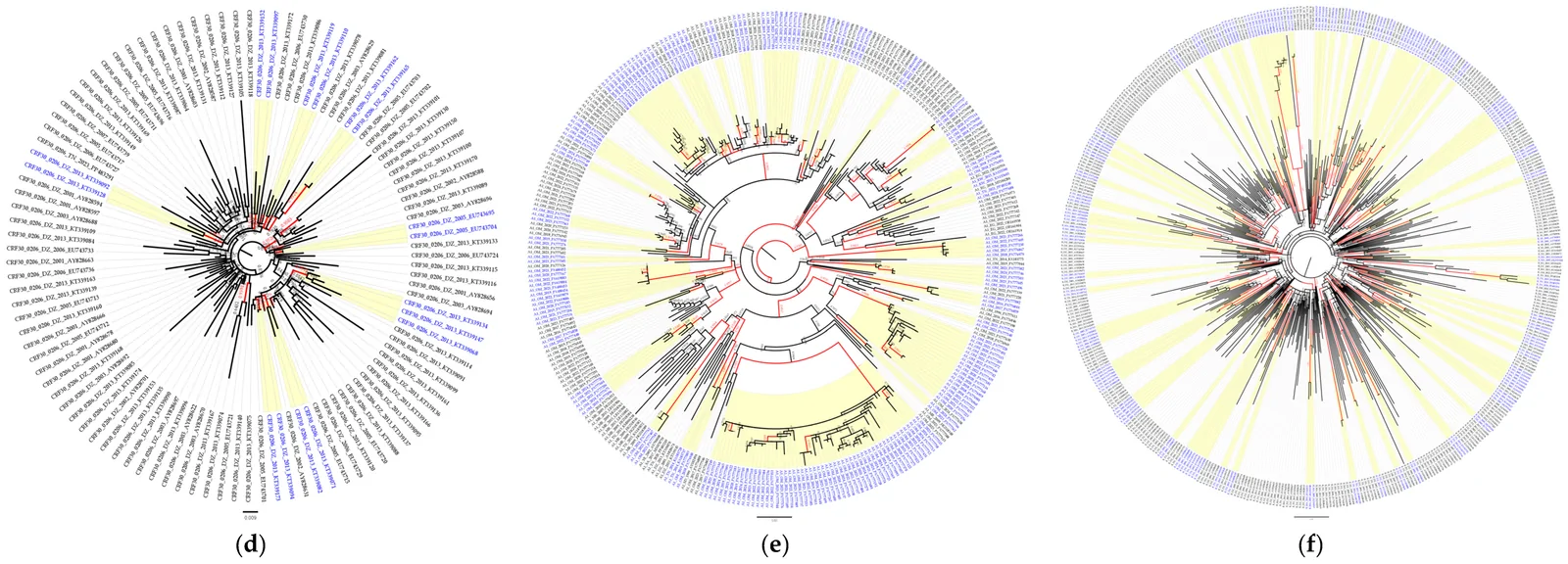

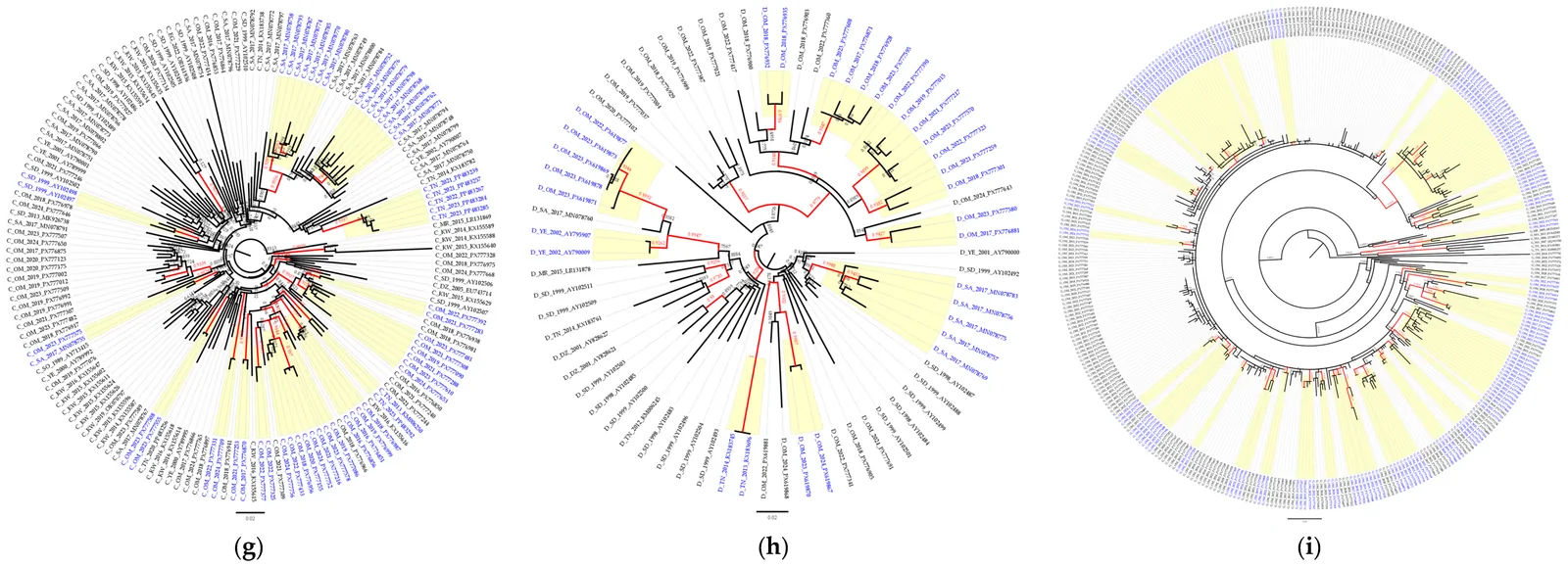
Maximum-likelihood phylogenetic trees of the Middle East and North Africa (MENA) HIV-1 *RT* region: (**a**) CRF01_AE tree; (**b**) CRF02_AG tree; (**c**) CRF06_cpx tree; (**d**) CRF30_0206 tree; (**e**) subtype A1 tree; (**f**) subtype B tree; (**g**) subtype C tree; (**h**) subtype D tree; (**i**) subtype G tree. Trees were visualized using FigTree v.1.4.4. (https://tree.bio.ed.ac.uk/software/figtree/, accessed 4 May 2026) and midpoint-rooted. Sequence names belonging to phylogenetic clusters are highlighted in blue, and branches with strong statistical support (Shimodaira–Hasegawa-like approximate likelihood ratio test (aLRT-SH) ≥0.90) are indicated in red. Phylogenetic clusters denoting statistically supported monophyletic clades with a maximum pairwise genetic distance of ≤0.045 substitutions/site are further highlighted in yellow boxes. Scale bars are shown below each tree. The original tree files (.tre) are supplied in Supplementary File S2.

Phylogenetic trees were reconstructed using ML analyses for each genomic region (two *env* regions, *PR*, and *RT*) to characterize the evolutionary relationships and clustering patterns of HIV-1 sequences across the MENA region. For each sub-gene region, sequences were stratified by subtype and CRFs, and representative trees are shown in (Figure 2, Figure 3, Figure 4 and Figure 5). These phylogenetic ML trees illustrate the distribution of major subtypes/CRFs, the relative genetic diversity within and between lineages, and the occurrence of phylogenetic clusters identified by branch statistical support and genetic distance threshold across countries.

The percentage of clustered HIV-1 MENA sequences varied by subtype/CRF and genomic region (Table 3). Overall, the clustering of the MENA HIV-1 sequences was observed in 23 of 224 sequences (10.3%) in the first *env* dataset and 5 of 60 (8.3%) in the second *env* dataset. In the *PR* dataset, 530 of 2413 sequences (22.0%) were in clusters, whereas in the *RT* dataset, 783 of 2103 sequences (37.2%) were clustered.

Cluster-level analysis showed that most phylogenetic clusters were small dyads or networks, with large clusters detected solely in Oman (Table 3). In the *PR* region, the largest clusters in size were observed for CRF01_AE in Oman (31 sequences, 2021–2025), subtype A1 in Oman (20 and 18 sequences, 2018–2024), and CRF02_AG in Tunisia (12 sequences, 2013–2022). Subtype B clusters in *PR* were generally smaller and were mainly detected in Morocco, Tunisia, Algeria, Kuwait, and Oman. In the *RT* region, larger clusters were more frequent, particularly for subtype A1 in Oman, including a 54-sequence cluster spanning 2019–2024 and an 18-sequence cluster spanning 2018–2024, and for subtype G in Oman, including clusters of 28, 22, and 15 sequences spanning 2016–2024. Most clusters were confined to a single country; however, several small multi-country clusters were identified, including Morocco–Mauritania, Oman–Mauritania, KSA–Oman, Egypt–Tunisia, KSA–Yemen, Algeria–Morocco, and Oman–Tunisia.

Across genomic regions, the percentage of MENA HIV-1 sequences assigned to phylogenetic clusters varied by time period, geographic region, and subtype/CRF grouping. Considering the low numbers in the *env* datasets, analyses focused on the *pol* datasets, as follows. In the *PR* dataset the percentage of clustered sequences increased across the time period, from 12.3% (30 of 243) before 2005 to 30.4% (233 of 767) in sequences collected since 2020 (*p* < 0.001, LBL). Clustering differed by geographic region (*p* < 0.001), with percentages of 25.3% (286 of 1129) in the GCC countries and Yemen and 19.7% (238 of 1211) in the Maghreb and Mauritania, whereas no clustered sequences were observed in the Horn of Africa and Sudan. After exclusion of *PR* HIV-1 sequences from Oman (*n* = 1465), the rising trend of clustering remained statistically significant (*p* = 0.016, LBL) with percentages increasing from 12.4% to 31.3% across the four periods. Nevertheless, the highest clustering was found in the Maghreb and Mauritania instead of the GCC and Yemen sub-region. Additionally, upon excluding the Omani HIV-1 sequences, clustering was higher for subtype B compared to non-B subtypes/CRFs (21.3% vs. 15.7%, *p* = 0.006, Table 4).

In the *RT* region, a similar increasing trend of clustering was identified from 14.4% (25 of 174) before 2005 to 50.6% (414 of 818) in sequences collected since 2020 (*p* < 0.001, LBL, Table 4). Clustering differed by geographic region (*p* < 0.001), with a higher percentage in the GCC countries and Yemen (47.1%) compared with other regions. No significant difference in clustering was observed between subtype B and non-B sequences (35.2% vs. 37.8%, *p* = 0.295). After exclusion of the Omani sequences (*n* = 1155), the increasing trend of clustering remained significant with an increase from 14.5% before 2005 to 33.6% after 2019 (*p* < 0.001, LBL, Table 4). On the other hand, the clustering was similar between the GCC countries and Yemen sub-region (26.8%) and the Maghreb and Mauritania sub-region (29.7%); however, these percentages were higher compared to the two other sub-regions (*p* = 0.010). Additionally, a statistically significant difference between HIV-1 subtype groups was found, with clustering in 33.6% of subtype B sequences and 21.8% of non-B sequences (*p* < 0.001).

### 3.4. Antiretroviral Drug Resistance

Antiretroviral drug resistance was assessed in the *PR* and *RT* datasets using SDRMs. In the *PR* dataset, 77 of 2390 evaluable *PR* sequences contained at least one PI-associated SDRM (3.2%). By contrast, resistance was substantially more frequent in the *RT* dataset. Among 2097 evaluable *RT* sequences, 190 contained at least one NRTI-associated SDRM (9.1%), 209 contained at least one NNRTI-associated SDRM (10.0%), and 85 contained both NRTI- and NNRTI-associated SDRMs (4.1%).

NRTI-associated SDRMs showed marked geographic heterogeneity. The highest prevalence was observed in Mauritania (31/97, 32.0%), followed by Tunisia (50/271, 18.5%), Algeria (56/310, 18.1%), and Morocco (37/252, 14.7%). In contrast, NRTI SDRMs were rare in Oman (5/948, 0.5%), despite it having the largest country-level sample size. Sudan/South Sudan and Yemen had no detected NRTI SDRMs, although these zero estimates should be interpreted cautiously because of limited sample sizes.

The prevalence of NNRTI-associated SDRMs also varied across countries. The highest prevalence was observed in Mauritania (40/97, 41.2%), followed by Tunisia (56/271, 20.7%) and Egypt (10/72, 13.9%). Intermediate levels were observed in Algeria (23/303, 7.6%), Kuwait (6/69, 8.7%), and Oman (63/947, 6.6%), whereas lower prevalence was noted in Morocco (12/252, 4.8%). No NNRTI SDRMs were detected in KSA or Sudan/South Sudan, although these estimates are constrained by limited sample sizes.

The prevalence of PI-associated SDRMs was uniformly low across the region, with the majority of countries demonstrating rates below 5%. The highest prevalence was observed in Algeria (23/305, 7.5%) and Tunisia (20/271, 7.4%), whereas Oman exhibited near-complete absence of PI resistance despite having the largest sample size (2/948, 0.2%). Several countries, including Lebanon, Sudan/South Sudan, and Yemen, had no detected PI SDRMs, although these estimates are limited by small sample sizes.

**Figure 6:**
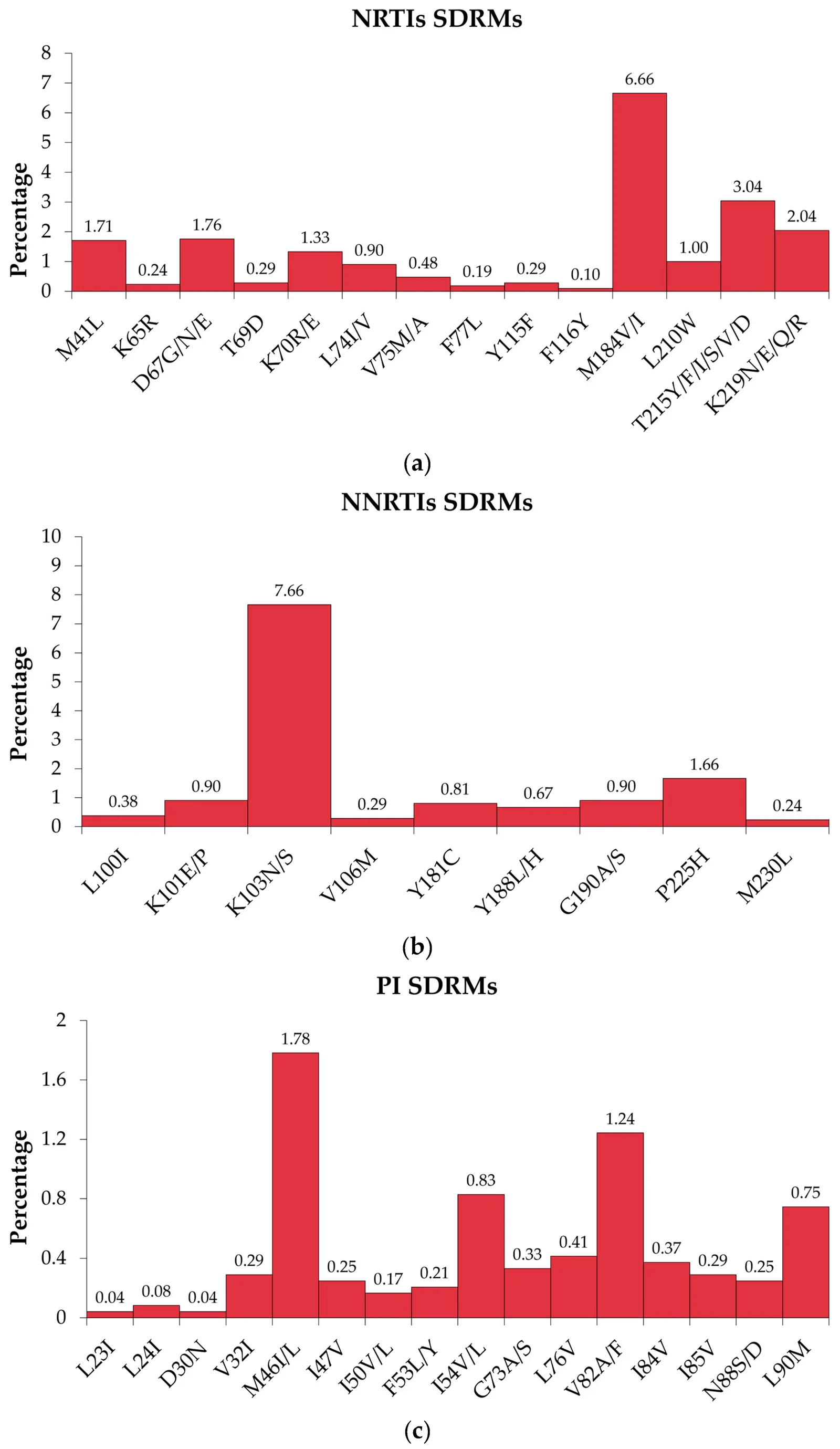
Distribution of HIV-1 drug-resistance mutations across genomic regions: (**a**) distribution of the number of nucleoside reverse-transcriptase inhibitor (NRTI)-associated surveillance drug-resistance mutations per sequence; (**b**) distribution of the number of non-nucleoside reverse-transcriptase inhibitor (NNRTI)-associated surveillance drug-resistance mutations per sequence; (**c**) distribution of the number of protease inhibitor (PI)-associated surveillance drug-resistance mutations per sequence.

The most frequent NRTI mutation pattern was M184V alone (*n* = 67), followed by combinations involving thymidine analog mutations, particularly M41L, D67N, K70R, L210W, T215F/Y, and K219E/Q/R (Figure 6). The dominant NNRTI mutation was K103N alone (*n* = 99), with additional recurrent patterns including K103N plus P225H (*n* = 27), K101E (*n* = 9), Y181C (*n* = 8), G190A (*n* = 8), and Y188L (*n* = 7, Figure 6). The most frequent PI SDRMs were M46I/L (*n* = 43), V82A/F (*n* = 30), I54V/L (*n* = 20), L90M (*n* = 18), L76V (*n* = 10), and I84V (*n* = 9, Figure 6).

### 3.5. Probability of Harboring Surveillance Drug-Resistance Mutations According to Phylogenetic Clustering

**Figure 7:**
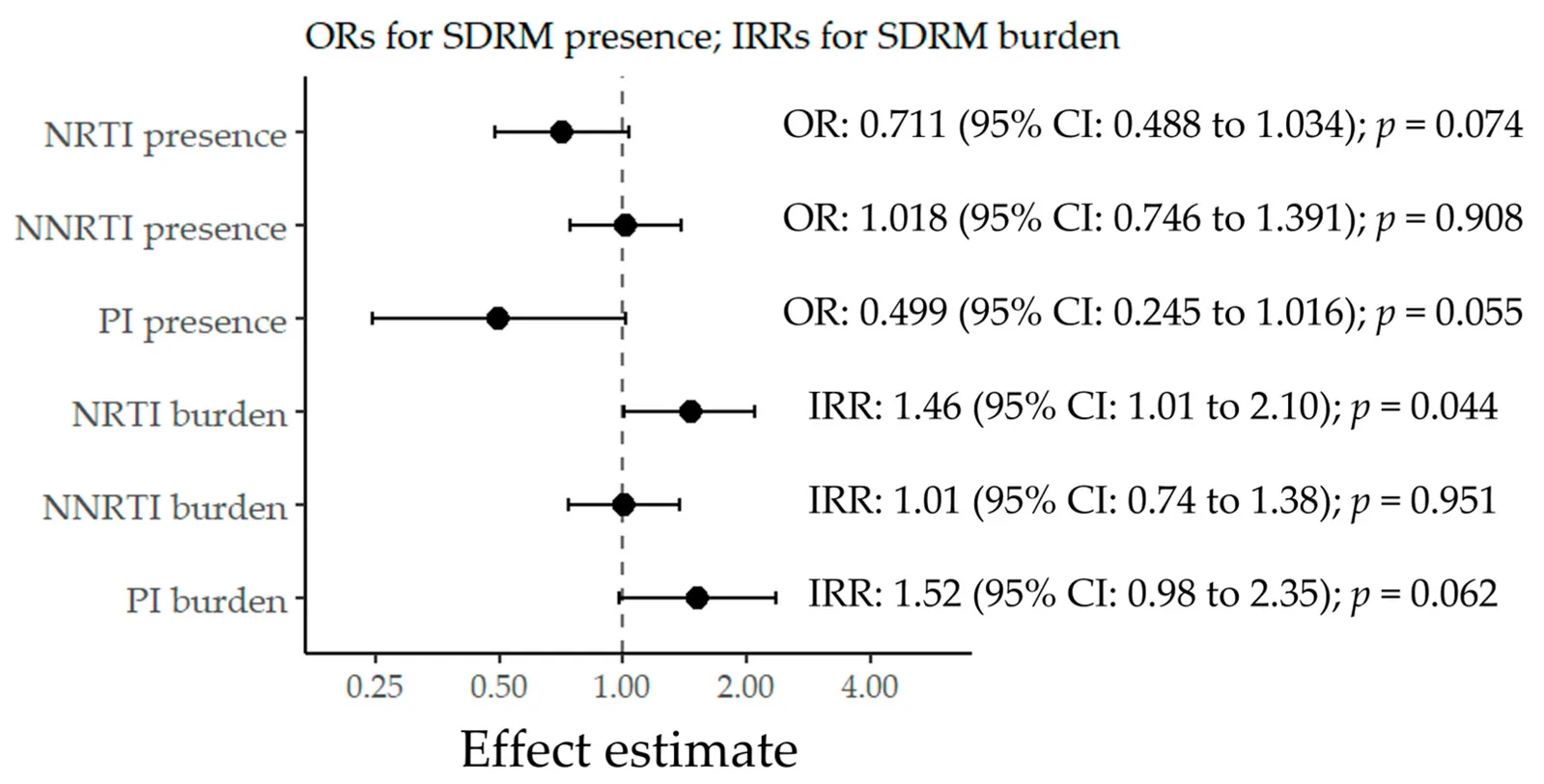
Forest plot showing the association between phylogenetic clustering of HIV-1 Middle East and North Africa (MENA) sequences and the presence and burden of surveillance drug-resistance mutations (SDRMs). Odds ratios (ORs) for the presence of nucleoside reverse-transcriptase inhibitor (NRTI)-, non-nucleoside reverse-transcriptase inhibitor (NNRTI)-, and protease inhibitor (PI)-associated SDRMs were derived from mixed-effects logistic regression models. Incidence rate ratios (IRRs) for SDRM burden, defined as the number of SDRMs per sequence, were derived from negative binomial regression models adjusted for period of sequence collection, geographic region, and subtype/CRF grouping. Horizontal lines indicate 95% confidence intervals, and the vertical dashed line represents the null value (effect estimate, 1.0). The x axis is shown on a logarithmic scale. *p* values are shown for the association between phylogenetic clustering and each outcome. The figure was created using R (version 4.2.3; R Foundation for Statistical Computing; Vienna, Austria) and RStudio (version 2025.05.1; Posit Software; Boston, MA, USA).

In a mixed-effects logistic regression model, phylogenetic clustering was not significantly associated with the presence of NRTI-associated SDRMs (*p* = 0.074). The odds of harboring an NRTI-associated SDRM were lower among non-clustered sequences than among clustered sequences (OR, 0.711; 95% CI, 0.488 to 1.034, Figure 7). There was no evidence of significant random-effect variability according to period of sequence collection (variance estimate, 0.573; *p* = 0.290), geographic region (variance estimate, 1.607; *p* = 0.345), or subtype/CRF grouping (variance estimate, 0.091; *p* = 0.544).

Additionally, phylogenetic clustering was not associated with the presence of NNRTI-associated SDRMs (OR, 1.018; 95% CI, 0.746 to 1.391; *p* = 0.908). There was no evidence of significant random-effect variability according to period of sequence collection (variance estimate, 0.591; *p* = 0.327) or geographic region (variance estimate, 0.248; *p* = 0.368).

Phylogenetic clustering was also not significantly associated with the presence of PI-associated SDRMs (OR, 0.499; 95% CI, 0.245 to 1.016; *p* = 0.055). There was no evidence of significant random-effect variability according to the period of sequence collection (variance estimate, 0.075; *p* = 0.455), geographic region (variance estimate, 1.510; *p* = 0.317), or subtype/CRF grouping (variance estimate, 0.194; *p* = 0.543).

In negative binomial regression models adjusted for period of sequence collection, geographic region, and subtype/CRF, phylogenetic clustering was associated with a higher number of NRTI-associated SDRMs. Non-clustered sequences had a greater mutation burden than clustered sequences (incidence rate ratio, 1.46; 95% CI, 1.01 to 2.10; *p* = 0.044). The number of NRTI-associated SDRMs decreased with more recent categorical periods of sequence collection (incidence rate ratio per period increment, 0.70; 95% CI, 0.58 to 0.83; *p* < 0.001), whereas higher counts were observed across geographic regions (incidence rate ratio, 1.73; 95% CI, 1.34 to 2.24; *p* < 0.001). Non-B subtypes/CRFs were associated with lower mutation counts than subtype B (incidence rate ratio, 0.42; 95% CI, 0.31 to 0.58; *p* < 0.001).

In contrast, phylogenetic clustering was not associated with the number of NNRTI-associated SDRMs (incidence rate ratio, 1.01; 95% CI, 0.74 to 1.38; *p* = 0.951). The number of NNRTI-associated SDRMs did not vary significantly according to period of sequence collection (incidence rate ratio, 1.11; 95% CI, 0.92 to 1.33; *p* = 0.284) or subtype/CRF grouping (incidence rate ratio, 0.80; 95% CI, 0.57 to 1.12; *p* = 0.186) but differed significantly across geographic regions (incidence rate ratio, 1.51; 95% CI, 1.19 to 1.92; *p* = 0.001).

Similarly, phylogenetic clustering was not significantly associated with the number of PI-associated SDRMs (incidence rate ratio, 1.52; 95% CI, 0.98 to 2.35; *p* = 0.062). In contrast, the number of PI-associated SDRMs differed significantly according to period of sequence collection (incidence rate ratio, 1.63; 95% CI, 1.29 to 2.07; *p* < 0.001), geographic region (incidence rate ratio, 3.33; 95% CI, 2.39 to 4.63; *p* < 0.001), and subtype grouping (incidence rate ratio, 0.22; 95% CI, 0.16 to 0.31; *p* < 0.001).

## 4. Discussion

The current study provided one of the most comprehensive molecular epidemiologic assessments of HIV-1 transmission dynamics and ARV drug resistance currently available from the MENA region. The present findings revealed a more complex and epidemiologically interconnected epidemic than previously recognized. Several observations emerge that warrant deeper elaboration, as follows.

First, the examination of the currently available public HIV-1 sequences in the MENA showed a marked heterogeneity in HIV-1 subtype/CRF distribution across countries. Specifically, geographically structured and temporally dynamic patterns of HIV-1 subtype/CRF were found in the MENA region over the past four decades. Such a level of genetic diversity suggests that the HIV-1 MENA epidemic can be described as a mixture of partially interconnected HIV-1 MENA sub-epidemics. These sub-epidemics are likely shaped by historical introductions, local transmission dynamics, and differential population mixing.

In comparison to available evidence from the MENA countries as well as global studies on HIV-1 genetic diversity, a consistent observation was the predominance of subtype B in the Maghreb region. This subtype B dominance was particularly evident in Morocco, Tunisia, and, to a lesser extent, Algeria [55,58,59,103,113,140,141]. This pattern closely follows the subtype B dominance in Western and Southern Europe and likely reflects sustained trans-Mediterranean epidemiologic connectivity as discussed in different epidemiologic reports [142,143,144]. In an early review from Morocco dating back to 2002, El-Harti et al. attributed this link to economic drivers, particularly migration and tourism [145]. In line with the predominance of subtype B in the Maghreb and to complete the regional picture given the absence of Libyan sequences in this study—an early study by Daw et al. reported subtype B as the dominant lineage in Libya as well, at 74% [146].

Nevertheless, a recent study from Morocco by Ahmina et al., although limited to 64 sequenced HIV-1 cases during 2024–2025, found that non-B subtypes, especially CRF02_AG, dominated among recent infections (73%) [58]. This is consistent with the significant increase in non-B subtype/CRF circulation observed in our study. An exception to the broader Maghreb pattern of subtype B dominance, Mauritania exhibited a predominance of CRF02_AG (>70%) as previously reported by Fall-Malick et al. [99]. This pattern is epidemiologically plausible given Mauritania’s geographic and transmission linkages with West Africa, the epicenter of this widely prevent HIV-1 recombinant form [147]. Equally notable was the considerable detection of CRF02_AG in Tunisia and Morocco. This distribution can be attributed to the northward spread of CRF02_AG from West Africa and supports the role of trans-Saharan migration and population mobility in shaping regional HIV-1 diversity in the MENA region [58,142].

In contrast to the subtype B dominance in the Maghreb, the current study indicated that the sub-region of the Horn of Africa and Sudan/South Sudan was characterized by the predominance of non-B subtypes (C and D). However, this finding was constrained by the limited sequence availability from this sub-region. The dominance of subtypes C and D is in line with patterns observed across East Africa and suggests underlying epidemiologic connectivity with neighboring high-prevalence countries such as Ethiopia and Kenya, which are dominated by these two HIV-1 subtypes, as comprehensively reviewed by Giovanetti et al. [148].

The GCC countries and Yemen —collectively referred to as the Arabian Peninsula— exhibited a more complex subtype/CRF distribution. In KSA for example, subtype C predominated, accompanied by various other HIV-1 lineages [106,107,108]. Kuwait likewise showed a marked subtype/CRF heterogeneity, including the frequent detection of CRF01_AE, subtype C, and subtype B, which was reported previously by the notable work of Chehadeh et al. [89,90,91]. Oman, on the other hand, displayed a distinct genetic diversity profile, characterized by high representation of subtypes G and A1 and CRF01_AE with dense sampling. The pattern in Yemen, where subtypes B and C appeared dominant, should also be interpreted cautiously because of the limited number of available sequences.

Rather than implying a single source of HIV-1 in the GCC countries, these patterns are more consistent with repeated introductions from multiple geographic regions, followed by variable degrees of local onward transmission. The large expatriate populations and longstanding migration links with East Africa, South Asia, and Southeast Asia provide a plausible contextual framework for this detected HIV-1 genetic diversity, although direct attribution requires caution [149]. For example, Al-Mozaini et al. reported that between 1984 and 2013, a total of 20,539 HIV-1 cases were identified in KSA, of which 71% occurred in non-Saudi residents [150]. Similarly in Oman, Al-Kindi and Al-Jardani reported that expatriates who constitute approximately 45% of the Omani population largely originate from regions with higher HIV-1 prevalence compared to Oman [151].

In the Levant and Egypt sub-region, HIV-1 subtype/CRF distributions were less clearly delineated, largely owing to the limited and uneven number of sequences retrieved in this study. In Lebanon, subtype B was prominent but coexisted with multiple non-B lineages, including CRF02_AG and CRF01_AE; however, the low sampling density and reliance on older sequences from the 1990s constrain robust inference regarding genetic diversity [94]. Iraqi sequences on other hand showed exclusive subtype A1 presence observed in one *env* dataset [87]. In Egypt, the subtype/CRF distribution appeared to vary by genomic region, with subtype B predominating in *env* and CRF02_AG in *RT*. Although more recent evidence suggests subtype B may represent the predominant lineage in Egypt, as reported by Daw et al. [141], conflicting findings have also been reported by Amer et al. with predominance of CRF02_AG [86]. More broadly, molecular epidemiologic studies in the Levant and Egypt sub-region were frequently limited by the lack of publicly available sequence data, precluding robust phylogenetic analyses in this MENA sub-region. For example, a recent molecular epidemiology study by Bakri et al. reported subtypes B, A1, and CRF01_AE in Jordan, but the absence of accessible sequences limited incorporation into regional HIV-1 phylogenetic and ARV drug resistance analyses [152].

Temporal analyses further revealed a notable shift from subtype B to non-B lineages over time in the MENA region albeit heavily confounded by geographic sampling biases across different time periods. Subtype B increased modestly during the early ART scale-up period but declined sharply in more recent years, with non-B subtypes/CRFs accounting for the overwhelming majority of sequences after 2020. This pattern is unlikely to be explained solely by stochastic variation and instead suggests a structural transition in the HIV-1 MENA epidemic. Several not mutually exclusive mechanisms may underlie this shift. First, increasing regional and international mobility may have facilitated repeated introductions of diverse non-B subtypes/CRFs similar to what has been reported in North America and Europe by Cabello et al. [153]. Second, differential transmission dynamics within key populations may favor the expansion of specific subtypes/CRFs [130,154,155]. Third, improvements in surveillance and sequencing may have broadened detection of previously underrepresented lineages [16]. However, the attenuation of temporal trends in this study after exclusion of the densely sampled Omani sequences indicates that part of this signal may reflect sampling structure rather than a uniform regional transition and thus should be interpreted with appropriate caution.

This shift toward non-B HIV-1 subtypes/CRFs has been reported across diverse epidemiologic settings. For example, Dennis et al. documented an increase in non-B subtype prevalence in North Carolina from 0% in 1997 to 3.46% in 2014 [156]. Similarly, in the Nordic countries, Esbjörnsson et al. showed that while subtype B historically predominated, its percentage declined over time with a concomitant rise in CRFs [130]. Likewise, in the United Kingdom, Ragonnet-Cronin et al. reported an increasing contribution of non-B subtypes/CRFs to the HIV-1 epidemic, particularly among heterosexuals [157]. Collectively, these findings suggest that the HIV-1 genetic diversity observed in the MENA region in our study reflects a broader global trend toward increasing diversification of HIV-1 lineages, likely driven by population mobility and the convergence of previously distinct transmission networks [158].

Second, the phylogenetic clustering findings in this study provided a molecular evidence that HIV-1 transmission in the MENA region is not explained solely by sporadic, unrelated importation events. Across the two *pol* datasets, a substantial percentage of HIV-1 sequences were part of statistically supported clusters, particularly in the *RT* region, where more than one third of sequences were clustered. This finding indicates non-random genetic relatedness among regional MENA HIV-1 sequences and is most consistent with the presence of interconnected transmission networks mainly within and infrequently across the MENA countries. These clustering percentages were lower than those reported in an earlier regional analysis; notably, a 2016 study found phylogenetic clustering in 54% of analyzed MENA *pol* sequences, with 363/675 HIV-1 sequences found within transmission clusters [55]. This disparity partly reflects methodological differences, particularly the use of shorter sequence fragments analyzed in separate datasets in the current study, which can reduce phylogenetic resolution and sensitivity for cluster detection [159]. Nevertheless, the common observation across both studies remains consistent; phylogenetic clustering occurs in considerable percentages within the MENA region, supporting the presence of interconnected, albeit small, transmission networks.

The higher clustering observed in *pol* sequences, especially among *RT* sequences, compared with the two *env* datasets should be interpreted biologically and methodologically. The *env* datasets were substantially smaller and genetically more variable, increasing phylogenetic noise and reducing statistical power for reliable cluster detection as opposed to the *pol* region. By contrast, *PR* and *RT* included substantially larger numbers of HIV-1 sequences that had higher sequence lengths and are were more commonly available because of their use in ARV drug-resistance testing. In addition, the HIV-1 *pol* genomic regions have been shown to be sufficiently informative for transmission reconstruction, as demonstrated by Hué et al. [117]. Therefore, the stronger clustering signal in *pol* likely reflects both greater sampling density and the suitability of these regions for population-level molecular epidemiology, rather than necessarily implying that transmission structure differs fundamentally by genomic region. The importance of sampling density was particularly highlighted in a study by Dasgupta et al., which demonstrated that lower sequence completeness reduces the ability to identify transmission clusters that may warrant public health investigation [160].

An additional key result in this study was the temporal increase in phylogenetic clustering in the MENA. In both *PR* and *RT*, the percentage of clustered HIV-1 sequences increased significantly over successive sampling periods, reaching 30.4% in *PR* and 50.6% in *RT* among sequences collected since 2020. This pattern supports the interpretation that local and regional transmission networks have become increasingly detectable over time. Importantly, this does not prove that transmission itself accelerated during the most recent period. Instead, increased sampling and sequencing of the HIV-1 cases and greater availability of resistance-testing sequences may have also increased the probability of detecting domestic MENA transmission clusters. Nevertheless, the persistence of the temporal trend after excluding the densely sampled HIV-1 Omani cases strengthened the inference that the rise in clustering is not solely a single-country sampling artifact.

The cluster-size distribution was equally informative. Most clusters were dyads or small networks, suggesting either short observed transmission chains or, more plausibly, incomplete capture of larger networks. This pattern is expected in a region where HIV-1 surveillance is uneven and at-risk groups are under-sampled [9,20]. In contrast, the detection of larger clusters—particularly in Oman, where sampling has been more comprehensive and molecular epidemiologic studies have characterized the epidemic across subtypes A1, G, and CRF01_AE—indicates that, in some settings, transmission networks are sufficiently dense and sustained to generate sizeable groups of closely related viruses. The prominent clustering observed in Oman likely reflects sustained local transmission networks rather than isolated introductions alone and the comparatively greater availability of molecular epidemiologic data from the country [161,162]. Several studies from Oman have extensively characterized the national HIV epidemic, including transmission patterns and clinical outcomes by Sannathimmappa et al. [163], HIV-1 care-cascade dynamics by Elgalib et al. [164], and ARV drug resistance by Al-Omairi et al. [139]. Consequently, the larger phylogenetic clusters identified in Oman may partly reflect greater sampling density and surveillance resolution relative to most MENA countries, in addition to true underlying transmission connectivity.

Geographically, clustering was most prominent in the GCC countries and Yemen in the full dataset, but this pattern was attenuated after removal of the Omani sequences. This is an important cautionary finding. It shows that regional estimates can be strongly influenced by high-volume countries with distinctive subtype/CRF distributions and concentrated sampling. After excluding Oman, clustering remained substantial in both the Maghreb–Mauritania and the GCC–Yemen sub-regions, indicating that transmission structure is not confined to Oman. However, the precise ranking of sub-regions should be interpreted cautiously because of unequal HIV-1 sequence availability.

The relatively high clustering observed in the Maghreb is also consistent with the earlier molecular epidemiology analysis from the MENA region conducted in 2016, which identified large transmission clusters in Tunisia and Algeria dating back to the early 1990s and 2000s, supporting sustained onward transmission rather than transient importation alone [55]. These phylogenetic observations are also epidemiologically plausible in light of behavioral transmission data from North Africa. For example, an early study by Mumtaz et al. using the Modes of Transmission mathematical model in Morocco demonstrated that new HIV-1 infections were concentrated disproportionately within high-risk populations, particularly among FSWs [165], which is also supported by the notable work in Morocco by Kouyoumjian et al. [166,167]. Such transmission structures provide a plausible epidemiologic substrate for the persistence and expansion of clustered HIV-1 lineages over time.

The subtype-specific results also require careful interpretation. In the full *pol* datasets, clustering did not differ substantially between subtype B and non-B subtypes/CRFs. After excluding Oman, however, subtype B showed higher clustering than non-B subtypes/CRFs in both *PR* and *RT*. This suggests that subtype B transmission networks, particularly in the Maghreb, may be more established or more consistently sampled. At the same time, prominent non-B clusters—especially A1, G, CRF01_AE, and CRF02_AG—show that onward transmission is not restricted to subtype B. The epidemic is therefore not simply a legacy subtype B epidemic with scattered non-B introductions; rather, several non-B subtypes/CRFs appear to have entered sustained transmission networks in the MENA region.

In this study, the identification of multi-country clusters is especially important. Although most clusters were confined within a single country, small cross-border clusters involving pairs such as Morocco–Mauritania, KSA–Oman, KSA–Yemen, Egypt–Tunisia, Algeria–Morocco, and Oman–Tunisia suggested regional connectivity in transmission networks. These findings should not be interpreted as evidence of direct person-to-person transmission across borders. Rather, they indicate recent shared ancestry among viruses sampled in different countries, compatible with mobility, migration, travel, or shared unsampled intermediate networks. The presence of such mixed-country clusters is consistent with the earlier 2016 molecular epidemiologic analysis from the MENA region, which likewise reported detectable—although relatively uncommon—lineage mixing across countries, particularly in North Africa [55].

The third key finding in this study was related to patterns of ARV drug resistance which revealed a low frequency of PI resistance and substantially higher levels of resistance in the *RT* region among the publicly available HIV-1 sequences included in this study. The low observed prevalence of PI-associated SDRMs across the MENA region—generally below 5%—may reflect the high genetic barrier to resistance of PI-based regimens, as well as their historically more limited and later use in many MENA settings [135,168,169]. This interpretation is supported by findings from Echchakery et al. in Morocco, where more than 94% of treated individuals were receiving NNRTI-based regimens, whereas only 8% were treated with PI-containing regimens [170]. In contrast, the higher prevalence of NRTI and NNRTI resistance—approaching 10% overall—suggests sustained selective pressure from first- and second-line therapies and is consistent with global patterns observed in settings with incomplete viral suppression [135,171,172].

The geographic heterogeneity in SDRM frequencies among the analyzed publicly available sequences in this study is unlikely to be explained by random variation alone. Countries such as Mauritania, Tunisia, Algeria, and Morocco demonstrated substantially higher prevalence of NRTI- and NNRTI-associated SDRMs, whereas the observed SDRM frequencies were notably lower among the analyzed Omani sequences despite the largest sample size. This contrast needs further elaboration. The near absence of resistance in Oman is unlikely to reflect true absence of resistance emergence; rather, it more plausibly reflects a combination of differences in treatment history, testing practices, and sampling frameworks, including potential overrepresentation of treatment-naïve individuals or earlier-stage infections. By contrast, higher resistance levels in North African countries may indicate longer-standing treatment programs, greater historical exposure to earlier-generation regimens, or more frequent sequencing of treatment-experienced individuals and factors known to facilitate resistance emergence, including incomplete viral suppression and suboptimal adherence. Supporting this interpretation, Magdy et al. reported 67% adherence to ART among PLWHA in Alexandria, Egypt, a pattern that may contribute to incomplete viral suppression and facilitate resistance emergence [138]. These interpretations are plausible but not directly testable in the absence of individual-level treatment histories and should therefore be regarded as informed inference rather than definitive explanation.

The relatively high observed NNRTI SDRM frequencies in the analyzed sequences from Mauritania and Tunisia warrant further investigation. NNRTIs have historically formed the backbone of first-line regimens in many low- and middle-income countries (LMICs) [173], and mutations such as K103N—identified here as the dominant NNRTI mutation—are known to confer high-level resistance to efavirenz and nevirapine [174]. The presence of K103N alone, as well as in combination with mutations such as P225H, suggests both selection under therapy and the potential for onward transmission of resistant variants [175,176]. However, because treatment history was not available for the analyzed sequences in this study, it is not possible to distinguish definitively between transmitted and acquired resistance. This distinction is critical and remains unresolved in the present dataset.

The NRTI mutation profile further supports the interpretation of cumulative treatment pressure. The predominance of M184V, often occurring alone, is consistent with widespread use of lamivudine or emtricitabine, as this mutation arises rapidly under drug pressure and confers high-level resistance while simultaneously reducing viral fitness [177]. The presence of thymidine analog mutations (TAMs), including M41L, D67N, K70R, L210W, and T215F/Y, reflects exposure to earlier-generation regimens and suggests that some resistance patterns may represent historical treatment eras rather than current therapeutic practices [178,179,180]. This temporal layering of HIV-1 resistance mutations is a well-recognized phenomenon and indicates that the resistance analyses detect both contemporary and legacy ART effects [181,182].

The PI resistance profile in this study was notable for its consistency across countries. The most frequently observed mutations—M46I/L, V82A/F, I54V/L, and L90M—are classical PI resistance mutations [183,184,185], yet their overall MENA prevalence remains low. This finding is highly reliable given the large dataset and supports the continued effectiveness of PI-based regimens in the region. From a policy perspective, this suggests that PI-based second-line therapies remain a robust option, particularly in settings where NNRTI resistance is increasing.

Finally, the relationship between phylogenetic clustering and ARV drug resistance suggested that there is no consistent evidence that sequences within phylogenetic clusters are more likely to harbor resistance mutations. However, there are signals suggesting differences in the burden of resistance that merit careful interpretation. In the logistic regression analyses, phylogenetic clustering was not significantly associated with the presence of NRTI-, NNRTI-, or PI-associated SDRMs. The direction of effect for NRTI and PI resistance—suggesting lower odds among non-clustered sequences—approached statistical significance but did not meet conventional thresholds. These results are best interpreted as evidence against a strong or uniform association between clustering and the mere presence of resistance, rather than definitive evidence of absence of association. Importantly, the absence of significant random-effect variability across time, geography, and subtype/CRF groupings suggested that this lack of association is not being masked by heterogeneity across these dimensions, lending additional robustness to the null findings.

At first glance, these results might appear to argue against the transmission of resistant viruses within clusters. However, such an interpretation would be overly simplistic. The presence of a resistance mutation in a sequence reflects a binary state, whereas transmission dynamics operate on a continuum. A cluster may contain both resistant and susceptible viruses, and resistance may be lost or diluted over transmission chains, particularly for mutations associated with fitness costs [186]. Therefore, the absence of a strong association with presence of resistance does not preclude ongoing transmission of resistant variants.

The negative binomial models provide a more informative perspective by examining the accumulation of ARV drug-resistance mutations. Here, a significant association emerged; non-clustered HIV-1 MENA sequences exhibited a higher burden of NRTI-associated SDRMs than clustered sequences. This finding suggested that viruses with multiple resistance mutations may be less likely to be embedded within active transmission clusters [186]. A plausible biological explanation is that viruses accumulating multiple NRTI mutations—particularly those involving TAMs—experience reduced replicative fitness, limiting their capacity for onward transmission, as demonstrated by Wertheim et al. [187]. In contrast, viruses within clusters may represent more recently transmitted lineages with fewer accumulated resistance mutations. An alternative plausible explanation is that non-clustered HIV-1 sequences represented older, chronic infections that have had more time to fail therapy and accumulate multiple mutations (and are thus less likely to cluster with recent transmissions), whereas clustered sequences represent recent incident infections.

The temporal trend toward a declining number of NRTI-associated mutations further supports this interpretation. The reduction in mutation burden over time likely reflects the transition away from older regimens associated with accumulation of multiple mutations toward more effective therapies that suppress viral replication earlier and more completely [186,188]. Nevertheless, this finding is contingent on the assumption that sampling across time is comparable—an assumption that may not fully hold in the present dataset. Specifically, this temporal trend appeared confounded by the disproportionate inclusion of recent sequences from Oman, which had a near absence of resistance (0.5%) and were collected predominantly between 2016 and 2024.

For NNRTI-associated resistance, neither the presence nor the number of mutations was associated with clustering. This result is notable given the high prevalence and transmissibility of mutations such as K103N. One possible interpretation is that NNRTI resistance, particularly single mutations, may be sufficiently common and persistent that it is distributed broadly across both clustered and non-clustered sequences, thereby diluting any detectable association. Alternatively, the lack of association may reflect competing processes of selection, transmission, and reversion, resulting in a relatively uniform distribution across the viral population.

Similarly, no statistically significant association was observed between clustering and PI-associated resistance, either in presence or mutation count, although the direction of effect in the count model approached significance. Given the overall low prevalence of PI resistance and the high genetic barrier of PI regimens, these findings should be interpreted cautiously. The limited number of PI-resistant sequences also reduces statistical power and may obscure subtle associations.

The consistent influence of geographic region across all models is an important observation. Resistance burden varied significantly by sub-region, independent of clustering status, underscoring the role of regional treatment practices, access to therapy, and historical regimen use in shaping resistance patterns. In contrast, subtype/CRF differences were more selective, with non-B subtypes associated with lower NRTI and PI mutation burdens. This may reflect differences in treatment exposure, underlying mutation pathways, or the epidemiologic contexts in which these subtypes/CRFs circulate [189].

### 4.1. Study Implications

From epidemiologic and public health perspectives, the study findings carry implications that extend beyond molecular characterization alone. The co-existence of multiple HIV-1 subtypes and CRFs across the MENA region indicates that the epidemic is not epidemiologically static, but dynamically evolving under the influence of migration, population mobility, and heterogeneous transmission networks. Such diversity creates conditions conducive to ongoing recombination and the potential emergence of novel viral forms with distinct biologic and therapeutic characteristics. In addition, subtype diversity may influence mutational pathways under ARV drug pressure, with potential implications for resistance evolution, diagnostic performance, and treatment optimization [190,191].

At the population level, the observed phylogenetic structure suggests that the HIV-1 epidemic in the MENA region is composed of partially interconnected sub-epidemics rather than isolated national epidemics. The predominance of within-country clustering indicates sustained domestic transmission, whereas the detection of mixed-country clusters demonstrates persistent regional connectivity through migration, travel, and shared transmission networks. Collectively, these findings challenge the longstanding perception that HIV-1 transmission in the MENA region is driven predominantly by sporadic external introductions with limited local spread.

The phylogenetic clustering findings also have direct practical implications for HIV-1 prevention and control. Molecular surveillance systems capable of integrating phylogenetic analysis with routine ARV drug-resistance testing may provide an early warning framework for identifying expanding transmission networks before they become clinically or epidemiologically apparent. In settings where stigma, delayed diagnosis, and fragmented surveillance continue to impede conventional case-based approaches, phylogenetic monitoring may offer a complementary strategy for detecting ongoing transmission dynamics in near real time.

From a transmission-dynamics perspective, the most consequential implication may lie not in the absolute prevalence of resistance mutations, but in their potential propagation within active transmission networks. Mutations associated with low fitness cost, such as K103N, may persist even in the absence of sustained drug pressure and continue to circulate within clustered lineages. When interpreted alongside the observed phylogenetic clustering patterns, these findings raise concern that resistant viral variants may already be embedded within ongoing transmission networks in parts of the MENA region. Although this study was not designed to directly establish transmitted drug resistance within specific clusters, the coexistence of clustering and resistance signals strongly supports the need for integrated molecular epidemiologic surveillance linking phylogenetics, ARV drug-resistance testing, and real-time public health response.

Ultimately, the findings of this study argue for a transition from fragmented and reactive HIV surveillance toward coordinated regional molecular epidemiology frameworks as also suggested by Mumtaz et al. [192]. Expanding sequence sharing, improving early diagnosis, strengthening linkage to care, and integrating phylogenetic monitoring into routine surveillance may collectively improve the ability to identify active transmission networks, interrupt onward spread, and limit the dissemination of resistant HIV-1 lineages across the MENA region. Such efforts could complement broader regional progress toward the UNAIDS 95-95-95 targets, which aim by 2030 to ensure that 95% of PLWHA know their status, 95% of diagnosed individuals receive sustained ART, and 95% of treated individuals achieve durable viral suppression [193,194,195].

### 4.2. Study Limitations

Several limitations should be considered when interpreting the findings of this study. First, the available HIV-1 sequences were derived from LAHDB rather than systematic population-based surveillance and are therefore inherently susceptible to sampling bias. The use of the publicly available LAHDB introduced potential sampling and reporting biases because sequence availability and metadata completeness vary according to national surveillance capacity, research activity, and data-sharing practices. Consequently, some countries may be underrepresented, and the findings should not be interpreted as direct estimates of HIV-1 population epidemiology in the MENA region. Sequence availability varied markedly across countries, genomic regions, and time periods, with some countries contributing extensive datasets (e.g., Oman, Morocco) while others remained completely lacking (Bahrain, Jordan, Palestine, Qatar, Syria, and the UAE). Consequently, regional subtype/CRF distributions and clustering estimates may disproportionately reflect countries with more active molecular surveillance or greater sequence sharing.

Second, the limited and uneven sampling density constrained the accuracy of subtype and CRF estimates and may obscure less prevalent lineages or transmission networks. The absence of clustering or ARV drug resistance in countries with few available sequences should therefore not be interpreted as evidence of absence of local transmission or drug resistance. Similarly, differences observed between genomic regions—particularly between *env* and *pol* datasets—may partly reflect variation in sequence availability, fragment length, and phylogenetic resolution rather than true biologic differences in epidemic structure. Because no universally accepted region-specific clustering criteria exist, particularly for *env*, clustering results should be interpreted with appropriate caution.

Third, phylogenetic clusters should be interpreted as molecular proxies of transmission connectivity rather than direct evidence of person-to-person transmission or transmission directionality. Large clusters may therefore represent intense local transmission, enhanced surveillance, or both. Conversely, incomplete datasets within the MENA region or the absence of comprehensive global background sequences may fragment larger international transmission chains into apparently smaller clusters or dyads.

Fourth, the ARV drug resistance analyses carried several important limitations. Resistance estimates were derived from publicly available sequences and may preferentially represent individuals with virologic failure, advanced disease, or access to specialized HIV-1 care. In addition, the absence of treatment history precluded distinction between transmitted and acquired resistance, limiting interpretation of the relationship between clustering and resistance patterns. Without longitudinal follow-up data, the temporal dynamics of ARV drug resistance emergence, persistence, and onward transmission also could not be reliably assessed. This is particularly relevant for mutations with low fitness cost that may persist within transmission networks even after treatment interruption or regimen change.

Fifth, although extensive efforts were undertaken to curate and deduplicate datasets, the possibility of inclusion of multiple sequences from the same individual cannot be completely excluded because of incomplete metadata in publicly available repositories. Such duplication could potentially inflate apparent clustering and affect estimates of subtype prevalence and ARV drug resistance frequencies. Moreover, although the prevalence of surveillance drug-resistance mutations was relatively low, convergent evolution at resistance-associated codons could potentially influence phylogenetic inference in pol-based analyses. Future studies may consider sensitivity analyses using resistance site masking to further evaluate the robustness of phylogenetic clustering.

Finally, integrase sequences were not analyzed because publicly available data from the MENA region were sparse and geographically restricted. As integrase inhibitor-based regimens, particularly dolutegravir, become increasingly adopted across the region, expanded generation and public deposition of integrase sequences will be essential for future molecular surveillance of HIV drug resistance.

## 5. Conclusions

Notwithstanding the study caveats, this large-scale molecular epidemiologic analysis demonstrated that the HIV-1 epidemic in the MENA region is genetically diverse, geographically structured, and sustained predominantly through localized transmission networks rather than repeated unrelated importation events alone. Although most detected phylogenetic clusters were small and country-confined, the presence of statistically supported clustering across multiple sub-regions provided molecular evidence of ongoing domestic transmission, with infrequent but detectable cross-border viral connectivity. The observed temporal shift from the historically dominant subtype B toward increasing circulation of non-B subtypes/CRFs further indicated that the regional epidemic is dynamically evolving under the influence of migration, population mobility, and changing transmission networks.

The MENA ARV drug resistance profile was characterized by low prevalence of PI-associated resistance and comparatively higher resistance within the *RT* region, consistent with differential ARV selective pressures across drug classes. Phylogenetic clustering was not associated with an increased probability of harboring SDRMs, suggesting that resistant variants are not being preferentially amplified within identifiable transmission clusters in the currently available datasets. Nevertheless, the greater burden of NRTI-associated mutations among non-clustered sequences supports a model in which resistance accumulation is driven predominantly by ART exposure, incomplete viral suppression, and long-term selective pressure rather than rapid onward transmission alone.

The study findings support the need for integrated molecular surveillance frameworks that combine phylogenetics, ARV drug resistance monitoring, and epidemiologic data to more accurately define transmission dynamics across the MENA region. Strengthening sequence sharing, expanding early diagnosis and ART coverage, and improving longitudinal treatment monitoring will be essential to limiting both onward HIV-1 transmission and the accumulation of ARV drug resistance in this historically under-characterized region.

## Figures and Tables

**Table 1:** Distribution of HIV-1 subtypes and circulating recombinant forms (CRFs) in the Middle East and North Africa (MENA) according to country and genomic region.

Subtype/CRF ^1^	A1	B	C	CRF01_AE	CRF02_AG	CRF06_cpx	CRF30_0206	D	G	Others ^6^
**Country**	**Genomic region *env* ^3^ (nucleotide position relative to HXB2 genome start: 7071 → 7283)**									
Algeria (*n* = 105)	1 (1.0)	68 (64.8)	0	0	0	0	29 (27.6)	2 (1.9)	3 (2.9)	2 (1.9)
Egypt (*n* = 19)	0	18 (94.7)	0	1 (5.3)	0	0	0	0	0	0
Lebanon (*n* = 23)	2 (8.7)	10 (43.5)	1 (4.3)	0	0	0	5 (21.7)	1 (4.3)	1 (4.3)	3 (13.0)
Morocco (*n* = 44)	2 (4.5)	32 (72.7)	1 (2.3)	0	0	0	0	0	0	9 (20.5)
Tunisia (*n* = 20)	0	19 (95.0)	0	0	0	0	1 (5.0)	0	0	0
**Country**	**Genomic region *env* (nucleotide position relative to HXB2 genome start: 7767 → 8342)**									
Iraq (*n* = 10)	10 (100)	0	0	0	0	0	0	0	0	0
KSA ^2^ (*n* = 41)	3 (7.3)	5 (12.2)	18 (43.9)	0	0	0	0	3 (7.3)	4 (9.8)	8 (19.5)
**Country**	**Genomic region *PR* ^4^**									
Djibouti (*n* = 16)	1 (6.3)	0	9 (56.3)	0	3 (18.8)	0	0	1 (6.3)	0	2 (12.5)
Algeria (*n* = 332)	26 (7.8)	115 (34.6)	0	0	52 (15.7)	81 (24.4)	0	3 (0.9)	5 (1.5)	50 (15.1)
Kuwait (*n* = 99)	4 (4.0)	16 (16.2)	22 (22.2)	34 (34.3)	6 (6.1)	1 (1.0)	0	0	0	16 (16.2)
Lebanon (*n* = 25)	4 (16.0)	11 (44.0)	0	0	5 (20.0)	0	0	2 (8.0)	1 (4.0)	2 (8.0)
Morocco (*n* = 511)	0	315 (61.6)	8 (1.6)	9 (1.8)	129 (25.2)	0	0	10 (2.0)	15 (2.9)	25 (4.9)
Mauritania (*n* = 97)	3 (3.1)	2 (2.1)	1 (1.0)	0	68 (70.1)	6 (6.2)	0	2 (2.1)	3 (3.1)	12 (12.3)
Oman (*n* = 948)	284 (30.0)	36 (3.8)	68 (7.2)	116 (12.2)	20 (2.1)	1 (0.1)	0	33 (3.5)	373 (39.3)	17 (1.8)
KSA (*n* = 63)	0	4 (6.3)	38 (60.3)	2 (3.2)	1 (1.6)	0	0	6 (9.5)	11 (17.5)	1 (1.6)
Sudan/South Sudan (*n* = 31)	2 (6.5)	1 (3.2)	11 (35.5)	0	0	0	0	16 (51.6)	0	1 (3.2)
Tunisia (*n* = 271)	4 (1.5)	105 (38.7)	10 (3.7)	1 (0.4)	126 (46.5)	9 (3.3)	0	4 (1.5)	6 (2.2)	6 (2.2)
Yemen (*n* = 19)	0	9 (47.4)	6 (31.6)	1 (5.3)	0	0	0	2 (10.5)	1 (5.3)	0
**Country**	**Genomic region *RT* ^5^**									
Algeria (*n* = 277)	3 (1.1)	115 (41.5)	1 (0.4)	0	32 (11.6)	10 (3.6)	112 (40.4)	2 (0.7)	0	2 (0.8)
Egypt (*n* = 72)	10 (13.9)	11 (15.3)	1 (1.4)	0	45 (62.5)	0	0	0	0	5 (6.9)
Kuwait (*n* = 69)	2 (2.9)	9 (13.0)	20 (29.0)	28 (40.6)	7 (10.1)	0	0	0	0	3 (4.3)
Morocco (*n* = 252)	10 (4.0)	196 (77.8)	7 (2.8)	0	31 (12.3)	0	0	0	3 (1.2)	5 (2.0)
Mauritania (*n* = 97)	10 (10.3)	2 (2.1)	1 (1.0)	0	73 (75.3)	0	0	1 (1.0)	1 (1.0)	9 (9.2)
Oman (*n* = 948)	287 (30.3)	39 (4.1)	68 (7.2)	114 (12.0)	27 (2.8)	0	0	39 (4.1)	366 (38.6)	8 (0.8)
KSA (*n* = 63)	1 (1.6)	4 (6.3)	38 (60.3)	2 (3.2)	10 (15.9)	0	0	6 (9.5)	0	2 (3.2)
Sudan/South Sudan (*n* = 31)	2 (6.5)	1 (3.2)	12 (38.7)	0	0	0	0	15 (48.4)	0	1 (3.2)
Tunisia (*n* = 271)	7 (2.6)	104 (38.4)	10 (3.7)	0	134 (49.4)	2 (0.7)	1 (0.4)	4 (1.5)	2 (0.7)	7 (2.6)
Yemen (*n* = 21)	1 (4.8)	10 (47.6)	6 (28.6)	0	0	0	0	3 (14.3)	0	1 (4.8)

^1^ CRF: Circulating recombinant form; ^2^ KSA: Kingdom of Saudi Arabia; ^3^ *env*: envelope gene; ^4^ *PR*: protease gene; ^5^ *RT*: reverse-transcriptase gene; ^6^ Others: minor or unassigned HIV-1 subtypes or CRFs. Only countries with ≥ 10 sequences per dataset are shown.

**Table 2:** Distribution of HIV-1 subtype B versus non-B subtypes/CRFs in the Middle East and North Africa (MENA) region according to genomic region, period of sequence collection, and geographic region, with analysis excluding Oman.

Genomic Region	Variable	Category	Subtype B n ^10^ (%)	Non-B Subtypes/CRFs ^11^ n (%)	*p* Value, χ^2^; LBL ^12^ *p* Value
*PR* ^1^, full dataset	Period	≤2004	99 (40.7)	144 (59.3)	<0.001, 459.523; <0.001
		2005–2012	274 (56.4)	212 (43.6)	
		2013–2019	208 (22.7)	709 (77.3)	
		≥2020	33 (4.3)	734 (95.7)	
*PR,* full dataset	Region	GCC and Yemen ^4^	65 (5.8)	1064 (94.2)	<0.001, 477.010; <0.001
		Maghreb and Mauritania ^5^	537 (44.3)	674 (55.7)	
		Levant and Egypt ^6^	11 (44.0)	14 (56.0)	
		Horn of Africa and Sudan ^7^	1 (2.1)	47 (97.9)	
*PR*, excluding Oman ^2^	Period	≤2004	99 (40.9)	143 (59.1)	<0.001, 103.370; <0.001
		2005–2012	274 (56.4)	212 (43.6)	
		2013–2019	190 (28.9)	467 (71.1)	
		≥2020	15 (18.8)	65 (81.3)	
*PR*, excluding Oman	Region	GCC and Yemen	29 (16.0)	152 (84.0)	<0.001, 82.001; 0.430
		Maghreb and Mauritania	537 (44.3)	674 (55.7)	
		Levant and Egypt	11 (44.0)	14 (56.0)	
		Horn of Africa and Sudan	1 (2.1)	47 (97.9)	
*RT* ^3^, full dataset	Period	≤2004	83 (47.7)	91 (52.3)	<0.001, 374.137; <0.001
		2005–2012	159 (55.2)	129 (44.8)	
		2013–2019	207 (25.2)	616 (74.8)	
		≥2020	42 (5.1)	776 (94.9)	
*RT,* full dataset	Region	GCC and Yemen	62 (5.6)	1039 (94.4)	<0.001, 471.599; <0.001
		Maghreb and Mauritania	417 (46.5)	480 (53.5)	
		Levant and Egypt ^8^	11 (15.1)	62 (84.9)	
		Horn of Africa and Sudan ^9^	1 (3.1)	31 (96.9)	
*RT*, excluding Oman	Period	≤2004	83 (48.0)	90 (52.0)	<0.001, 73.594; <0.001
		2005–2012	159 (55.2)	129 (44.8)	
		2013–2019	189 (33.6)	374 (66.4)	
		≥2020	21 (16.0)	110 (84.0)	
*RT*, excluding Oman	Region	GCC and Yemen	23 (15.0)	130 (85.0)	<0.001, 92.849; 0.684
		Maghreb and Mauritania	417 (46.5)	480 (53.5)	
		Levant and Egypt	11 (15.1)	62 (84.9)	
		Horn of Africa and Sudan	1 (3.1)	31 (96.9)	

^1^ *PR*: Protease gene sequences dataset; ^2^ excluding Oman: analyses performed after removal of sequences from Oman to assess whether observed patterns reflected regional trends rather than country-specific effects, given the disproportionate contribution and distinct subtype/CRF distribution of sequences from Oman.; ^3^ *RT*: reverse-transcriptase gene sequences dataset; ^4^ GCC and Yemen: included Gulf Cooperation Council countries (Kingdom of Saudi Arabia (KSA), Kuwait, and Oman) in addition to Yemen; ^5^ Maghreb and Mauritania: included Algeria, Morocco, and Tunisia in addition to Mauritania; ^6^ Levant and Egypt: included Lebanon only in the *PR* dataset; ^7^ Horn of Africa and Sudan: included Djibouti, Somalia, Sudan, and South Sudan in the *PR* dataset; ^8^ Levant and Egypt: included Iraq and Egypt in the *RT* dataset; ^9^ Horn of Africa and Sudan: included Somalia, Sudan, and South Sudan in the *RT* dataset; ^10^ n: number; ^11^ CRF: circulating recombinant form; ^12^ LBL: linear-by-linear test for association.

**Table 3:** Distribution of phylogenetic clustering across HIV-1 subtypes/CRFs by genomic region in the Middle East and North Africa (MENA).

Subtype/CRF ^1^, Genomic Region Analyzed	Total n ^6^ of HIV-1 Sequences	n and Classification of Clusters ^7^	Cluster Size (Mean, Range)	n of Clustered Sequences (%)
CRF01_AE, *PR* ^2^	158	13 (large = 1, network = 7, dyad = 5)	6.2, 2–31	81 (51.3%)
CRF01_AE, *RT* ^3^	144	19 (large = 0, network = 7, dyad = 12)	3.3, 2–9	63 (43.8%)
CRF02_AG, *PR*	406	33 (large = 0, network = 8, dyad = 25)	2.6, 2–12	87 (21.4%)
CRF02_AG, *RT*	352	34 (large = 0, network = 13, dyad = 21)	2.8, 2–8	94 (26.7%)
CRF06_cpx, *PR*	98	6 (large = 0, network = 0, dyad = 6)	2	12 (12.2%)
CRF06_cpx, *RT*	12	2 (large = 0, network = 1, dyad = 1)	3.5, 2–5	7 (58.3%)
CRF30_0206, *RT*	107	8 (large = 0, network = 1, dyad = 7)	2.1, 2–3	17 (15.9%)
CRF30_0206, *env1* ^4^	35	2 (large = 0, network = 0, dyad = 2)	2	4 (11.4)
A1, *env2* ^5^	12	0	-	0
A1, *PR*	326	29 (large = 1, network = 11, dyad = 17)	3.8, 2–20	109 (33.4%)
A1, *RT*	321	35 (large = 2, network = 17, dyad = 16)	5.2, 2–54	183 (57.0%)
B, *env1*	150	10 (large = 0, network = 1, dyad = 9)	2.1, 2–3	21 (14.0%)
B, *PR*	600	57 (large = 0, network = 16, dyad = 41)	2.5, 2–8	143 (23.8%)
B, *RT*	471	64 (large = 0, network = 21, dyad = 43)	2.7, 2–9	173 (36.7%)
C, *env2*	22	2 (large = 0, network = 1, dyad = 1)	2.5, 2–3	5 (22.7%)
C, *PR*	165	12 (large = 0, network = 4, dyad = 8)	2.6, 2–5	31 (18.8%)
C, *RT*	157	16 (large = 0, network = 6, dyad = 10)	3.5, 2–8	56 (35.7%)
D, *PR*	77	6 (large = 0, network = 2, dyad = 4)	3.0, 2–6	18 (23.4%)
D, *RT*	70	11 (large = 0, network = 4, dyad = 7)	3.0, 2–5	33 (47.1%)
G, *PR*	414	14 (large = 0, network = 7, dyad = 7)	3.5, 2–7	48 (11.8%)
G, *RT*	372	37 (large = 3, network = 16, dyad = 18)	4.4, 2–28	161 (43.3%)

^1^ CRF: Circulating recombinant form; ^2^ *PR*: protease gene sequences dataset; ^3^ *RT*: reverse-transcriptase gene sequences dataset; ^4^ *env1*: genomic region *env* dataset (nucleotide position relative to HXB2 genome start: 7071–7283); ^5^ *env2*: genomic region *env* (nucleotide position relative to HXB2 genome start: 7767–8342); ^6^ n: number; ^7^ classification of clusters: dyad is a cluster comprising two sequences, network a cluster of 3–14 sequences, and a large cluster has 15 or more sequences.

**Table 4:** Association of phylogenetic clustering with temporal, geographic, and subtype characteristics across genomic regions of HIV-1 in the Middle East and North Africa (MENA).

Genomic Region	Variable	Category	Clustered n ^19^/N ^20^ (%)	*p* Value, χ^2^; LBL ^21^ *p* Value
Genomic region *env* ^1^ (nucleotide position relative to HXB2 genome start: 7071 → 7283)	Period	≤2004	21/179 (11.7)	0.309, 2.348; 0.126
		2005–2012	2/35 (5.7)	
		2013–2019	0/10 (0)	
		≥2020	0	
	Region	GCC and Yemen ^6^	0/6 (0)	0.039, 8.342
		Maghreb and Mauritania ^7^	23/169 (13.6)	
		Levant and Egypt ^8^	0/42 (0)	
		Horn of Africa and Sudan ^9^	0/7 (0)	
	Subtype/CRF ^5^	B	19/151 (12.6)	0.101, 2.695
		Non-B	4/73 (5.5)	
Genomic region *env* (nucleotide position relative to HXB2 genome start: 7767 → 8342)	Period	≤2004	5/50 (10.0)	0.580, 1.091; 0.306
		2005–2012	0	
		2013–2019	0/9 (0)	
		≥2020	0/1 (0)	
	Region	GCC and Yemen ^10^	2/47 (4.3)	<0.001, 34.932
		Maghreb and Mauritania	0	
		Levant and Egypt ^11^	0/10 (0)	
		Horn of Africa and Sudan ^12^	3 (100.0)	
	Subtype/CRF	B	0/8 (0.0)	0.360, 0.839
		Non-B	5/52 (9.6)	
*PR* ^2^*,* full dataset	Period	≤2004	30/243 (12.3)	<0.001, 51.956; <0.001
		2005–2012	95/486 (19.5)	
		2013–2019	172/917 (18.8)	
		≥2020	233/767 (30.4)	
	Region	GCC and Yemen ^13^	286/1129 (25.3)	<0.001, 24.816
		Maghreb and Mauritania ^14^	238/1211 (19.7)	
		Levant and Egypt ^15^	6/25 (24.0)	
		Horn of Africa and Sudan ^16^	0/48 (0)	
	Subtype/CRF	B	143/614 (23.3)	0.358, 0.844
		Non-B	387/1799 (21.5)	
*PR*, excluding Oman ^3^	Period	≤2004	30/242 (12.4)	0.001, 15.923; 0.016
		2005–2012	95/486 (19.5)	
		2013–2019	112/657 (17.0)	
		≥2020	25/80 (31.3)	
	Region	GCC and Yemen	18/181 (9.9)	<0.001, 21.440
		Maghreb and Mauritania	238/1211 (19.7)	
		Levant and Egypt	6/25 (24.0)	
		Horn of Africa and Sudan	0/48 (0)	
	Subtype/CRF	B	123/578 (21.3)	0.006, 7.498
		Non-B	139/887 (15.7)	
*RT* ^4^*,* full dataset	Period	≤2004	25/174 (14.4)	<0.001, 122.765; <0.001
		2005–2012	78/288 (27.1)	
		2013–2019	266/823 (32.3)	
		≥2020	414/818 (50.6)	
	Region	GCC and Yemen	519/1101 (47.1)	<0.001, 106.525
		Maghreb and Mauritania	250/897 (27.9)	
		Levant and Egypt ^17^	12/73 (16.4)	
		Horn of Africa and Sudan ^18^	2/32 (6.3)	
	Subtype/CRF	B	173/491 (35.2)	0.295, 1.095
		Non-B	610/1612 (37.8)	
*RT*, excluding Oman	Period	≤2004	25/173 (14.5)	<0.001, 17.064; <0.001
		2005–2012	78/288 (27.1)	
		2013–2019	158/563 (28.1)	
		≥2020	44/131 (33.6)	
	Region	GCC and Yemen	41/153 (26.8)	0.010, 11.424
		Maghreb and Mauritania	250/897 (27.9)	
		Levant and Egypt	12/73 (16.4)	
		Horn of Africa and Sudan	2/32 (6.3)	
	Subtype/CRF	B	152/452 (33.6)	<0.001, 19.927
		Non-B	153/703 (21.8)	

^1^ *env*: Envelope gene; ^2^ *PR*: protease gene sequences dataset; ^3^ excluding Oman: analyses performed after removal of sequences from Oman to assess whether observed patterns reflected regional trends rather than country-specific effects, given the disproportionate contribution and distinct subtype/CRF distribution of sequences from Oman.; ^4^ *RT*: reverse-transcriptase gene sequences dataset; ^5^ CRF: circulating recombinant form; ^6^ GCC and Yemen: No Gulf Cooperation Council countries were included in this dataset and only sequences from Yemen were found; ^7^ Maghreb and Mauritania: included Algeria, Morocco, and Tunisia without any sequences from Mauritania; ^8^ Levant and Egypt: included sequences from Lebanon and Egypt; ^9^ Horn of Africa and Sudan: included sequences from Somalia, Sudan, and South Sudan; ^10^ GCC and Yemen: included sequences from Kingdom of Saudi Arabia (KSA) and Yemen; ^11^ Levant and Egypt: included sequences from Iraq only; ^12^ Horn of Africa and Sudan: included sequences from Somalia only; ^13^ GCC and Yemen: included Gulf Cooperation Council countries (Kingdom of Saudi Arabia (KSA), Kuwait, and Oman) in addition to Yemen; ^14^ Maghreb and Mauritania: included Algeria, Morocco, and Tunisia in addition to Mauritania; ^15^ Levant and Egypt: included Lebanon only in the *PR* dataset; ^16^ Horn of Africa and Sudan: included Djibouti, Somalia, Sudan, and South Sudan in the *PR* dataset; ^17^ Levant and Egypt: included Iraq and Egypt in the *RT* dataset; ^18^ Horn of Africa and Sudan: included Somalia, Sudan, and South Sudan in the *RT* dataset; ^19^ n: number of clustered sequences; ^20^ N: total number of HIV-1 sequences; ^21^ LBL: linear-by-linear test for association.

## Data Availability

The original contributions presented in this study are included in the article/Supplementary Material. Further inquiries can be directed to the corresponding author (MaS).

## Supplementary Materials

The following supporting information can be downloaded at https://www.mdpi.com/article/10.3390/v18080897/s1, Supplementary File S1: Figure S1. HIV-1 phylogenetic subtyping. The original tree files (.tre) are supplied in Supplementary File S2.

## Abbreviations

The following abbreviations are used in this manuscript:

AIDS	Acquired immunodeficiency syndrome
aLRT-SH	Shimodaira–Hasegawa-like approximate likelihood ratio test
APE	Analysis of phylogenetics and evolution
ART	Antiretroviral therapy
ARV	Antiretroviral
CI	Confidence interval
CPR	Calibrated population resistance
CRF	Circulating recombinant form
*env*	HIV envelope gene
FSWs	Female sex workers
GBD	The global burden of disease study
GCC	The Gulf Cooperation Council
GTR	Generalized time-reversible model
HIV	Human immunodeficiency virus
HIV-1	Human immunodeficiency virus type 1
IDU	Injection drug use
IDUs	Injection drug users
IRRs	Incidence rate ratios
KSA	Kingdom of Saudi Arabia
LAHDB	The Los Alamos HIV sequence database
LBL	Linear-by-linear test for association
LMICs	Low- and middle-income countries
MEGA	Molecular evolutionary genetics analysis
MENA	The Middle East and North Africa
ML	Maximum likelihood
MSA	Multiple sequence alignment
MSM	Men who have sex with men
NNRTIs	Non-nucleoside reverse-transcriptase inhibitors
NRTIs	Nucleoside reverse-transcriptase inhibitors
OR	Odds ratio
ORF	Open reading frame
PIs	Protease inhibitors
PLWHA	People living with HIV/AIDS
*pol*	HIV polymerase gene
*PR*	HIV protease gene region
QC	Quality control
QoL	Quality of life
*RT*	HIV reverse-transcriptase gene region
SDRMs	Surveillance drug-resistance mutations
s/s	Substitutions/site
TAMs	Thymidine analog mutations
TasP	Treatment as prevention
UAE	The United Arab Emirates
UNAIDS	Joint United Nations programme on HIV/AIDS

## Appendix A

**Table A1:** Reference HIV-1 sequences used for maximum-likelihood phylogenetic subtyping.

Accession Number	Country	Year	Subtype/CRF
AY093607	United States	1999	CRF09_cpx
AY371149	Cameroon	2001	CRF11_cpx
AY444809	United States	1999	CRF02_AG
AY588971	Cuba	1999	CRF19_cpx
AY945713	Thailand	2001	CRF01_AE
DQ396400	South Africa	2004	A
DQ845388	Cameroon	2002	CRF13_cpx
EF158043	Afghanistan	2005	CRF35_A1D
EU693240	Cameroon	2006	CRF25_cpx
FJ900266	Angola	2006	F1
FN392874	Democratic Republic of the Congo	1997	CRF45_cpx
HQ385477	Gambia	2002	CRF49_cpx
JN248585	Nigeria	2009	CRF02_AG
JQ403028	Switzerland	2003	A
JQ403106	United States	2003	B
JX112861	China	2002	CRF01_AE
K03455	France	1983	B
KF716476	Kenya	2011	D
KJ787684	Brazil	2010	D
KJ849758	Brazil	2010	CRF70_BF1
KP109516	South Africa	2012	C
KX907394	Tanzania	2004	C
KY968401	Argentina	2000	B
MH705144	Cameroon	2001	F2
MH705157	Democratic Republic of the Congo	1987	A
MN153475	Cameroon	2010	CRF18_cpx
MN485984	Belgium	2015	C
MN486044	Belgium	2008	G
MN486046	Belgium	2006	H
MW006072	Uganda	2010	D
