# Supplementary material for: Phylogenetic Evidence of Local HIV-1 Transmission and Antiretroviral Drug Resistance in the Middle East and North Africa
Source: Viruses. 2026 Aug 14;18(8):897. doi: 10.3390/v18080897 (PMC13517895; doi:10.3390/v18080897)
Supplement: Supplementary file 1 [file viruses-18-00897-s001.zip › Supplementary File S1.zip!/Figure_S1.pdf]

Figure S1
HIV-1 Phylogenetic Subtyping

The four figures below show maximum-likelihood phylogenetic trees used for HIV-1 subtyping of Middle East and North Africa (MENA) HIV-1 sequences in comparison with reference HIV-1 sequences for the *env*1, *env*2, protease (*PR*), and reverse-transcriptase (*RT*) regions. Analysis was done using maximum-likelihood (ML) phylogenetic trees were reconstructed using PhyML (version 3.0) under a general time-reversible nucleotide substitution model with gamma-distributed rate heterogeneity across sites and a proportion of invariant sites (GTR+Γ+I) [1,2]. Rate variation was modeled using a discrete gamma distribution with four categories. Model parameters, including base frequencies, substitution rates, gamma shape parameter, and proportion of invariant sites, were estimated directly from the data during tree optimization.

Branch support was assessed using the Shimodaira-Hasegawa-like approximate likelihood ratio test (aLRT-SH) [3]. Phylogenetic trees were exported in FigTree-compatible format (FigTree v.1.4.4.) [4].

References

1. Guindon, S.; Dufayard, J.F.; Lefort, V.; Anisimova, M.; Hordijk, W.; Gascuel, O. New algorithms and methods to estimate maximum-likelihood phylogenies: assessing the performance of PhyML 3.0. *Syst Biol*. 2010;**59**(3):307-321, doi:10.1093/sysbio/syq010.
2. Guindon, S.; Gascuel, O. A simple, fast, and accurate algorithm to estimate large phylogenies by maximum likelihood. *Syst Biol*. 2003;**52**(5):696-704, doi:10.1080/10635150390235520.
3. Anisimova, M.; Gil, M.; Dufayard, J.F.; Dessimoz, C.; Gascuel, O. Survey of branch support methods demonstrates accuracy, power, and robustness of fast likelihood-based approximation schemes. *Syst Biol*. 2011;**60**(5):685-699, doi:10.1093/sysbio/syr041.
4. Rambaut, A.; Drummond, A.J. Molecular evolution, phylogenetics and epidemiology: FigTree v.1.4.4. Available online: https://tree.bio.ed.ac.uk/software/figtree/ (accessed on 4 May 2026).

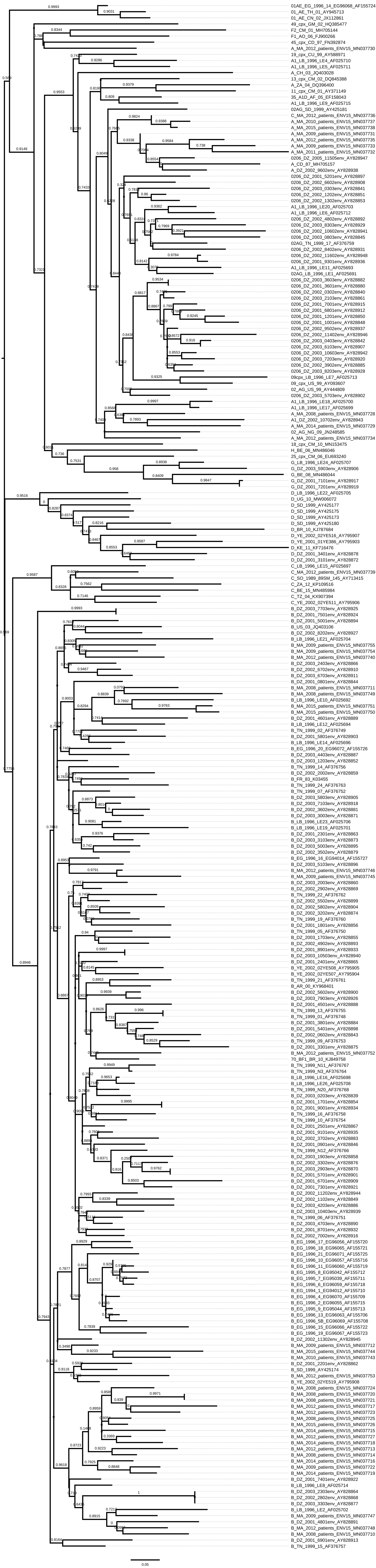
01AE_EG_1996_14_EG96068_AF155724
01_AE_TH_01_AY945713
01_AE_CN_02_JX112861
49_cpx_GM_02_HQ385477
F2_CM_01_MH705144
F1_AO_06_FJ900266
45_cpx_CD_97_FN392874
A_MA_2012_patients_ENV15_MN037730
19_cpx_CU_99_AY588971
A1_LB_1996_LE4_AF025710
A1_LB_1996_LE5_AF025711
A_CH_03_JQ403028
13_cpx_CM_02_DQ845388
A_ZA_04_DQ396400
11_cpx_CM_01_AY371149
35_A1D_AF_05_EF158043
A1_LB_1996_LE9_AF025715
02AG_SD_1999_AY425181
C_MA_2012_patients_ENV15_MN037736
A_MA_2010_patients_ENV15_MN037737
A_MA_2015_patients_ENV15_MN037738
A_MA_2009_patients_ENV15_MN037731
A_MA_2012_patients_ENV15_MN037735
A_MA_2009_patients_ENV15_MN037733
A_MA_2011_patients_ENV15_MN037732
0206_DZ_2005_11505env_AY828947
A_CD_87_MH705157
A_DZ_2002_9602env_AY828938
0206_DZ_2001_5201env_AY828897
0206_DZ_2002_6602env_AY828908
0206_DZ_2003_0303env_AY828841
0206_DZ_2002_1202env_AY828851
0206_DZ_2002_1302env_AY828853
A1_LB_1996_LE20_AF025703
A1_LB_1996_LE6_AF025712
0206_DZ_2002_4802env_AY828892
0206_DZ_2003_8303env_AY828929
0206_DZ_2002_10602env_AY828941
0206_DZ_2003_0803env_AY828845
02AG_TN_1999_17_AF376759
0206_DZ_2002_8402env_AY828931
0206_DZ_2002_11602env_AY828948
0206_DZ_2001_9301env_AY828936
A1_LB_1996_LE11_AF025693
02AG_LB_1996_LE1_AF025691
0206_DZ_2003_3603env_AY828882
0206_DZ_2001_3601env_AY828880
0206_DZ_2002_0302env_AY828840
0206_DZ_2003_2103env_AY828861
0206_DZ_2001_7001env_AY828915
0206_DZ_2001_6801env_AY828912
0206_DZ_2001_1201env_AY828850
0206_DZ_2001_1001env_AY828848
0206_DZ_2002_9502env_AY828937
0206_DZ_2002_11402env_AY828946
0206_DZ_2003_0403env_AY828842
0206_DZ_2003_6103env_AY828907
0206_DZ_2003_10603env_AY828942
0206_DZ_2003_7203env_AY828920
0206_DZ_2002_3902env_AY828885
0206_DZ_2003_8203env_AY828928
09cpx_LB_1996_LE7_AF025713
09_cpx_US_99_AY093607
A1_AG_US_99_AY444809
0206_DZ_2003_5703env_AY828902
A1_LB_1996_LE18_AF025700
A1_LB_1996_LE17_AF025699
A_MA_2008_patients_ENV15_MN037728
A1_DZ_2002_10702env_AY828943
A_MA_2014_patients_ENV15_MN037729
02_AG_NG_09_JN248585
A_MA_2012_patients_ENV15_MN037734
18_cpx_CN_10_MN153475
H_BE_06_MN486046
25_cpx_CM_06_EU693240
G_LB_1996_LE24_AF025707
G_DZ_2003_5903env_AY828906
G_BE_08_MN486044
G_DZ_2001_7101env_AY828917
G_DZ_2001_7201env_AY828919
D_LB_1996_LE22_AF025705
D_UG_10_MW006072
D_SD_1999_AY425177
D_SD_1999_AY425175
D_SD_1999_AY425173
D_SD_1999_AY425180
D_BR_10_KJ787684
D_YE_2002_02YE516_AY795907
D_YE_2001_01YE386_AY795903
D_KE_11_KF716476
D_DZ_2001_3401env_AY828878
D_DZ_2001_3101env_AY828872
C_LB_1996_LE15_AF025697
C_MA_2012_patients_ENV15_MN037739
C_SO_1989_89SM_145_AY713415
C_ZA_12_KP109516
C_BE_15_MN485984
C_TZ_04_KX907394
C_YE_2002_02YE511_AY795906
B_DZ_2003_7703env_AY828925
B_DZ_2001_7501env_AY828924
B_DZ_2001_5001env_AY828894
B_US_03_JQ403106
B_DZ_2002_8202env_AY828927
B_LB_1996_LE21_AF025704
B_MA_2009_patients_ENV15_MN037755
B_MA_2009_patients_ENV15_MN037754
B_MA_2012_patients_ENV15_MN037740
B_DZ_2003_2403env_AY828866
B_DZ_2002_6702env_AY828910
B_DZ_2003_6703env_AY828911
B_DZ_2001_0801env_AY828844
B_MA_2008_patients_ENV15_MN037711
B_MA_2008_patients_ENV15_MN037749
B_LB_1996_LE10_AF025692
B_MA_2015_patients_ENV15_MN037751
B_MA_2015_patients_ENV15_MN037750
B_DZ_2001_4601env_AY828889
B_LB_1996_LE12_AF025694
B_DZ_2001_5801env_AY828903
B_LB_1996_LE14_AF025696
B_EG_1996_20_EG96072_AF155726
B_DZ_2003_4403env_AY828887
B_DZ_2003_1203env_AY828852
B_TN_1999_14_AF376756
B_DZ_2002_2002env_AY828859
B_FR_83_K03455
B_TN_1999_24_AF376763
B_TN_1999_07_AF376752
B_DZ_2003_5803env_AY828905
B_DZ_2003_7103env_AY828918
B_DZ_2002_3602env_AY828881
B_DZ_2003_3003env_AY828871
B_LB_1996_LE23_AF025706
B_LB_1996_LE19_AF025701
B_DZ_2001_2301env_AY828863
B_DZ_2003_3103env_AY828873
B_DZ_2003_5003env_AY828895
B_DZ_2002_3502env_AY828879
B_EG_1996_16_EG94014_AF155717
B_DZ_2003_5103env_AY828896
B_MA_2012_patients_ENV15_MN037746
B_MA_2009_patients_ENV15_MN037745
B_DZ_2003_2003env_AY828860
B_DZ_2002_2902env_AY828869
B_TN_1999_22_AF376762
B_DZ_2002_5502env_AY828899
B_DZ_2002_5802env_AY828904
B_DZ_2002_3202env_AY828874
B_TN_1999_19_AF376760
B_DZ_2001_1801env_AY828856
B_TN_1999_05_AF376750
B_DZ_2003_1703env_AY828855
B_DZ_2002_4902env_AY828893
B_DZ_2001_8901env_AY828933
B_DZ_2003_10503env_AY828940
B_DZ_2001_2401env_AY828865
B_YE_2002_02YE508_AY795905
B_YE_2002_02YE507_AY795904
B_TN_1999_21_AF376761
B_AR_00_KY968401
B_DZ_2002_5602env_AY828900
B_DZ_2003_7903env_AY828926
B_DZ_2001_4501env_AY828888
B_TN_1999_13_AF376755
B_TN_1999_01_AF376748
B_DZ_2001_3801env_AY828884
B_DZ_2001_5401env_AY828898
B_DZ_2002_0602env_AY828843
B_TN_1999_09_AF376753
B_DZ_2001_3301env_AY828875
B_MA_2012_patients_ENV15_MN037752
70_BF1_BR_10_KJ849758
B_TN_1999_N11_AF376767
B_TN_1999_N3_AF376764
B_LB_1996_LE16_AF025698
B_LB_1996_LE26_AF025708
B_TN_1999_N20_AF376768
B_DZ_2003_0203env_AY828839
B_DZ_2001_1701env_AY828854
B_DZ_2001_9001env_AY828934
B_TN_1999_16_AF376758
B_TN_1999_10_AF376754
B_DZ_2001_2501env_AY828867
B_DZ_2001_9101env_AY828935
B_DZ_2002_3702env_AY828883
B_DZ_2001_0901env_AY828846
B_TN_1999_N12_AF376766
B_DZ_2003_1903env_AY828858
B_DZ_2002_3302env_AY828876
B_DZ_2003_2903env_AY828870
B_DZ_2001_5701env_AY828901
B_DZ_2001_6701env_AY828909
B_DZ_2001_7301env_AY828921
B_DZ_2002_11202env_AY828944
B_DZ_2002_1102env_AY828849
B_DZ_2003_4203env_AY828886
B_DZ_2003_10403env_AY828939
B_TN_1999_06_AF376751
B_DZ_2003_4703env_AY828890
B_DZ_2001_8701env_AY828932
B_DZ_2002_7002env_AY828916
B_EG_1996_17_EG96056_AF155720
B_EG_1996_18_EG96065_AF155721
B_EG_1996_21_EG96071_AF155725
B_EG_1996_12_EG96057_AF155716
B_EG_1996_11_EG96060_AF155719
B_EG_1995_8_EG95042_AF155712
B_EG_1995_7_EG95039_AF155711
B_EG_1996_6_EG96059_AF155718
B_EG_1994_1_EG94012_AF155710
B_EG_1996_4_EG96070_AF155709
B_EG_1996_2_EG96055_AF155715
B_EG_1995_9_EG95044_AF155713
B_EG_1996_13_EG96063_AF155706
B_EG_1996_5B_EG96069_AF155708
B_EG_1996_15_EG96066_AF155722
B_EG_1996_19_EG96067_AF155723
B_DZ_2002_11302env_AY828945
B_MA_2009_patients_ENV15_MN037712
B_MA_2015_patients_ENV15_MN037744
B_MA_2010_patients_ENV15_MN037743
B_DZ_2001_2201env_AY828862
B_SD_1999_AY425174
B_MA_2012_patients_ENV15_MN037753
B_YE_2002_02YE519_AY795908
B_MA_2008_patients_ENV15_MN037724
B_MA_2008_patients_ENV15_MN037720
B_MA_2008_patients_ENV15_MN037721
B_MA_2012_patients_ENV15_MN037717
B_MA_2012_patients_ENV15_MN037723
B_MA_2008_patients_ENV15_MN037725
B_MA_2015_patients_ENV15_MN037726
B_MA_2014_patients_ENV15_MN037715
B_MA_2012_patients_ENV15_MN037727
B_MA_2014_patients_ENV15_MN037718
B_MA_2012_patients_ENV15_MN037713
B_MA_2008_patients_ENV15_MN037714
B_MA_2014_patients_ENV15_MN037716
B_MA_2009_patients_ENV15_MN037722
B_MA_2014_patients_ENV15_MN037719
B_DZ_2001_7401env_AY828922
B_LB_1996_LE8_AF025714
B_DZ_2003_2303env_AY828864
B_DZ_2002_2802env_AY828868
B_DZ_2003_3303env_AY828877
B_LB_1996_LE2_AF025702
B_MA_2009_patients_ENV15_MN037747
B_DZ_2001_4801env_AY828891
B_MA_2012_patients_ENV15_MN037748
B_MA_2008_patients_ENV15_MN037710
B_DZ_2001_6901env_AY828913
B_TN_1999_15_AF376757
0.05

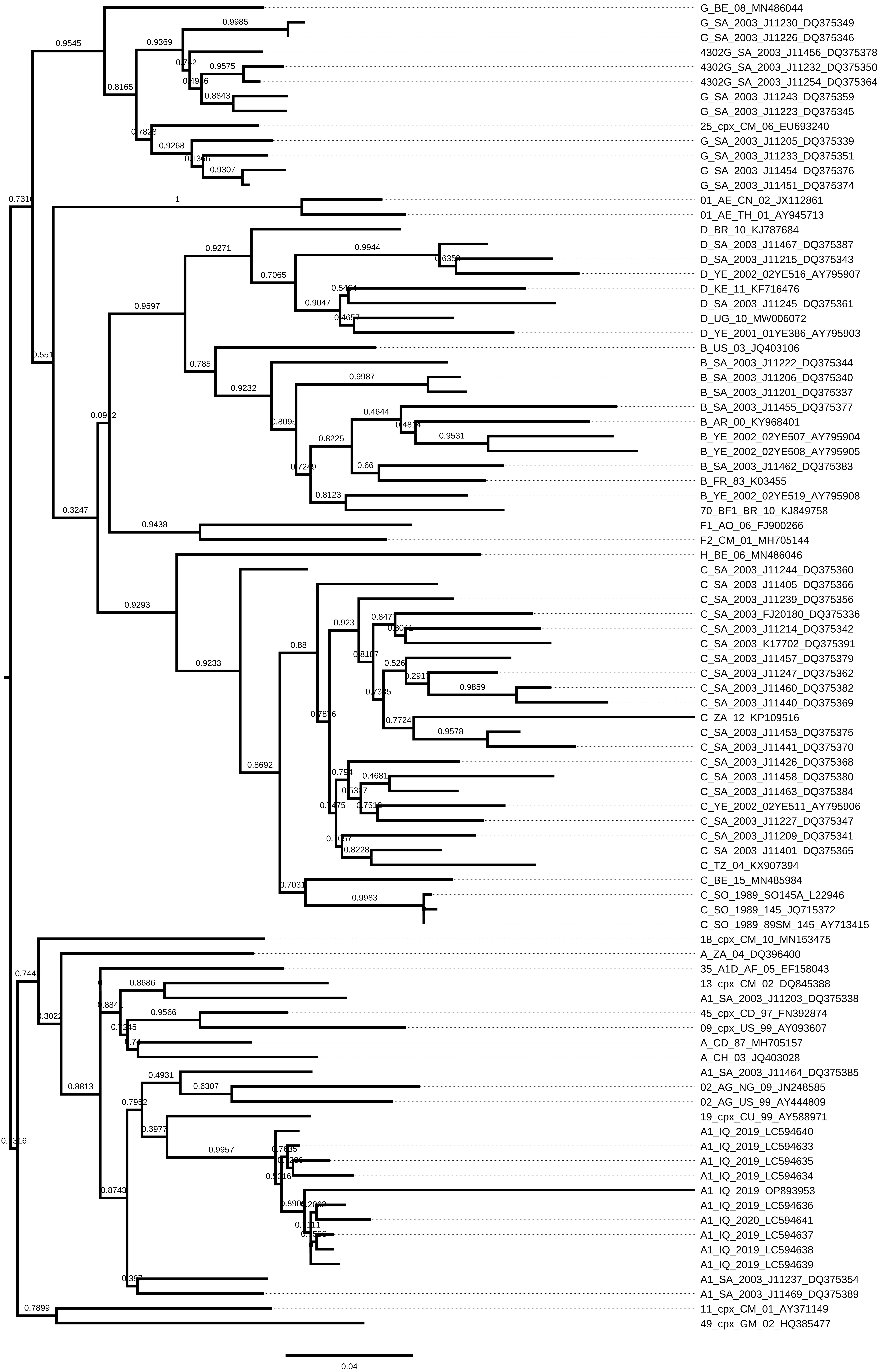
G_BE_08_MN486044
G_SA_2003_J11230_DQ375349
G_SA_2003_J11226_DQ375346
4302G_SA_2003_J11456_DQ375378
4302G_SA_2003_J11232_DQ375350
4302G_SA_2003_J11254_DQ375364
G_SA_2003_J11243_DQ375359
G_SA_2003_J11223_DQ375345
25_cpx_CM_06_EU693240
G_SA_2003_J11205_DQ375339
G_SA_2003_J11233_DQ375351
G_SA_2003_J11454_DQ375376
G_SA_2003_J11451_DQ375374
01_AE_CN_02_JX112861
01_AE_TH_01_AY945713
D_BR_10_KJ787684
D_SA_2003_J11467_DQ375387
D_SA_2003_J11215_DQ375343
D_YE_2002_02YE516_AY795907
D_KE_11_KF716476
D_SA_2003_J11245_DQ375361
D_UG_10_MW006072
D_YE_2001_01YE386_AY795903
B_US_03_JQ403106
B_SA_2003_J11222_DQ375344
B_SA_2003_J11206_DQ375340
B_SA_2003_J11201_DQ375337
B_SA_2003_J11455_DQ375377
B_AR_00_KY968401
B_YE_2002_02YE507_AY795904
B_YE_2002_02YE508_AY795905
B_SA_2003_J11462_DQ375383
B_FR_83_K03455
B_YE_2002_02YE519_AY795908
70_BF1_BR_10_KJ849758
F1_AO_06_FJ900266
F2_CM_01_MH705144
H_BE_06_MN486046
C_SA_2003_J11244_DQ375360
C_SA_2003_J11405_DQ375366
C_SA_2003_J11239_DQ375356
C_SA_2003_FJ20180_DQ375336
C_SA_2003_J11214_DQ375342
C_SA_2003_K17702_DQ375391
C_SA_2003_J11457_DQ375379
C_SA_2003_J11247_DQ375362
C_SA_2003_J11460_DQ375382
C_SA_2003_J11440_DQ375369
C_ZA_12_KP109516
C_SA_2003_J11453_DQ375375
C_SA_2003_J11441_DQ375370
C_SA_2003_J11426_DQ375368
C_SA_2003_J11458_DQ375380
C_SA_2003_J11463_DQ375384
C_YE_2002_02YE511_AY795906
C_SA_2003_J11227_DQ375347
C_SA_2003_J11209_DQ375341
C_SA_2003_J11401_DQ375365
C_TZ_04_KX907394
C_BE_15_MN485984
C_SO_1989_SO145A_L22946
C_SO_1989_145_JQ715372
C_SO_1989_89SM_145_AY713415
18_cpx_CM_10_MN153475
A_ZA_04_DQ396400
35_A1D_AF_05_EF158043
13_cpx_CM_02_DQ845388
A1_SA_2003_J11203_DQ375338
45_cpx_CD_97_FN392874
09_cpx_US_99_AY093607
A_CD_87_MH705157
A_CH_03_JQ403028
A1_SA_2003_J11464_DQ375385
02_AG_NG_09_JN248585
02_AG_US_99_AY444809
19_cpx_CU_99_AY588971
A1_IQ_2019_LC594640
A1_IQ_2019_LC594633
A1_IQ_2019_LC594635
A1_IQ_2019_LC594634
A1_IQ_2019_OP893953
A1_IQ_2019_LC594636
A1_IQ_2020_LC594641
A1_IQ_2019_LC594637
A1_IQ_2019_LC594638
A1_IQ_2019_LC594639
A1_SA_2003_J11237_DQ375354
A1_SA_2003_J11469_DQ375389
11_cpx_CM_01_AY371149
49_cpx_GM_02_HQ385477
0.04

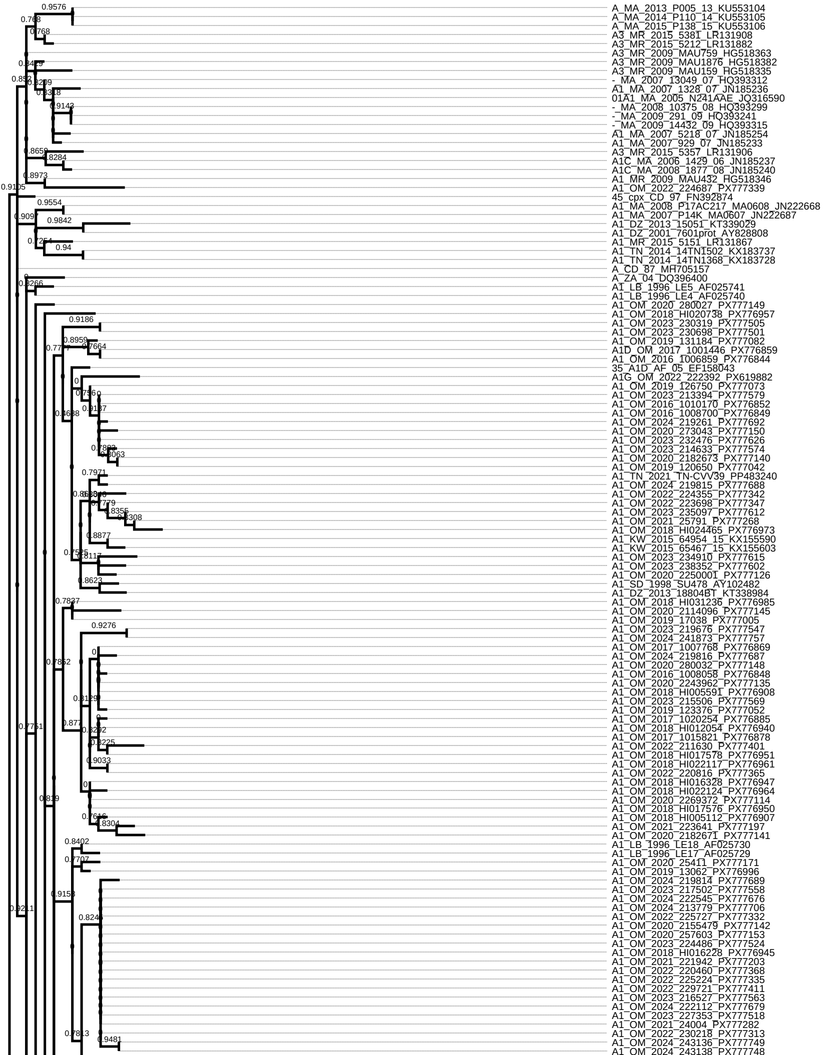
A_MA_2013_P005_13_KU553104
A_MA_2014_P110_14_KU553105
A_MA_2015_P138_15_KU553106
A3_MR_2015_5381_LR131908
A3_MR_2015_5212_LR131882
A3_MR_2009_MAU759_HG518363
A3_MR_2009_MAU1876_HG518382
A3_MR_2009_MAU159_HG518335
-_MA_2007_13049_07_HQ393312
A1_MA_2007_1328_07_JN185236
01A1_MA_2005_N241AAE_JQ316590
-_MA_2008_10375_08_HQ393299
-_MA_2009_291_09_HQ393241
-_MA_2009_14432_09_HQ393315
A1_MA_2007_5218_07_JN185254
A1_MA_2007_929_07_JN185233
A3_MR_2015_5357_LR131906
A1C_MA_2006_1429_06_JN185237
A1C_MA_2008_1877_08_JN185240
A1_MR_2009_MAU432_HG518346
A1_OM_2022_224687_PX777339
45_cpx_CD_97_FN392874
A1_MA_2008_P17AC217_MA0608_JN222668
A1_MA_2007_P14K_MA0607_JN222687
A1_DZ_2013_15051_KT339029
A1_DZ_2001_7601prot_AY828808
A1_MR_2015_5151_LR131867
A1_TN_2014_14TN1502_KX183737
A1_TN_2014_14TN1368_KX183728
A_CD_87_MH705157
A_ZA_04_DQ396400
A1_LB_1996_LE5_AF025741
A1_LB_1996_LE4_AF025740
A1_OM_2020_280027_PX777149
A1_OM_2018_HI020738_PX776957
A1_OM_2023_230319_PX777505
A1_OM_2023_230698_PX777501
A1_OM_2019_131184_PX777082
A1D_OM_2017_1001446_PX776859
A1_OM_2016_1006859_PX776844
35_A1D_AF_05_EF158043
A1G_OM_2022_222392_PX619882
A1_OM_2019_126750_PX777073
A1_OM_2023_213394_PX777579
A1_OM_2016_1010170_PX776852
A1_OM_2016_1008700_PX776849
A1_OM_2024_219261_PX777692
A1_OM_2020_273043_PX777150
A1_OM_2023_232476_PX777626
A1_OM_2023_214633_PX777574
A1_OM_2020_2182673_PX777140
A1_OM_2019_120650_PX777042
A1_TN_2021_TN-CVV39_PP483240
A1_OM_2024_219815_PX777688
A1_OM_2022_224355_PX777342
A1_OM_2022_223698_PX777347
A1_OM_2023_235097_PX777612
A1_OM_2021_25791_PX777268
A1_OM_2018_HI024465_PX776973
A1_KW_2015_64954_15_KX155590
A1_KW_2015_65467_15_KX155603
A1_OM_2023_234910_PX777615
A1_OM_2023_238352_PX777602
A1_OM_2020_2250001_PX777126
A1_SD_1998_SU478_AY102482
A1_DZ_2013_18804BT_KT338984
A1_OM_2018_HI031236_PX776985
A1_OM_2020_2114096_PX777145
A1_OM_2019_17038_PX777005
A1_OM_2023_219676_PX777547
A1_OM_2024_241873_PX777757
A1_OM_2017_1007768_PX776869
A1_OM_2024_219816_PX777687
A1_OM_2020_280032_PX777148
A1_OM_2016_1008058_PX776848
A1_OM_2020_2243962_PX777135
A1_OM_2018_HI005591_PX776908
A1_OM_2023_215506_PX777569
A1_OM_2019_123376_PX777052
A1_OM_2017_1020254_PX776885
A1_OM_2018_HI012054_PX776940
A1_OM_2017_1015821_PX776878
A1_OM_2022_211630_PX777401
A1_OM_2018_HI017578_PX776951
A1_OM_2018_HI022117_PX776961
A1_OM_2022_220816_PX777365
A1_OM_2018_HI016328_PX776947
A1_OM_2018_HI022124_PX776964
A1_OM_2020_2269372_PX777114
A1_OM_2018_HI017576_PX776950
A1_OM_2018_HI005112_PX776907
A1_OM_2021_223641_PX777197
A1_OM_2020_2182671_PX777141
A1_LB_1996_LE18_AF025730
A1_LB_1996_LE17_AF025729
A1_OM_2020_25411_PX777171
A1_OM_2019_13062_PX776996
A1_OM_2024_219814_PX777689
A1_OM_2023_217502_PX777558
A1_OM_2024_222545_PX777676
A1_OM_2024_213779_PX777706
A1_OM_2022_225727_PX777332
A1_OM_2020_2155479_PX777142
A1_OM_2020_257603_PX777153
A1_OM_2023_224486_PX777524
A1_OM_2018_HI016228_PX776945
A1_OM_2021_221942_PX777203
A1_OM_2022_220460_PX777368
A1_OM_2022_225224_PX777335
A1_OM_2022_229721_PX777411
A1_OM_2023_216527_PX777563
A1_OM_2024_222112_PX777679
A1_OM_2023_227353_PX777518
A1_OM_2021_24004_PX777282
A1_OM_2022_230218_PX777313
A1_OM_2024_243136_PX777749
A1_OM_2024_243138_PX777748

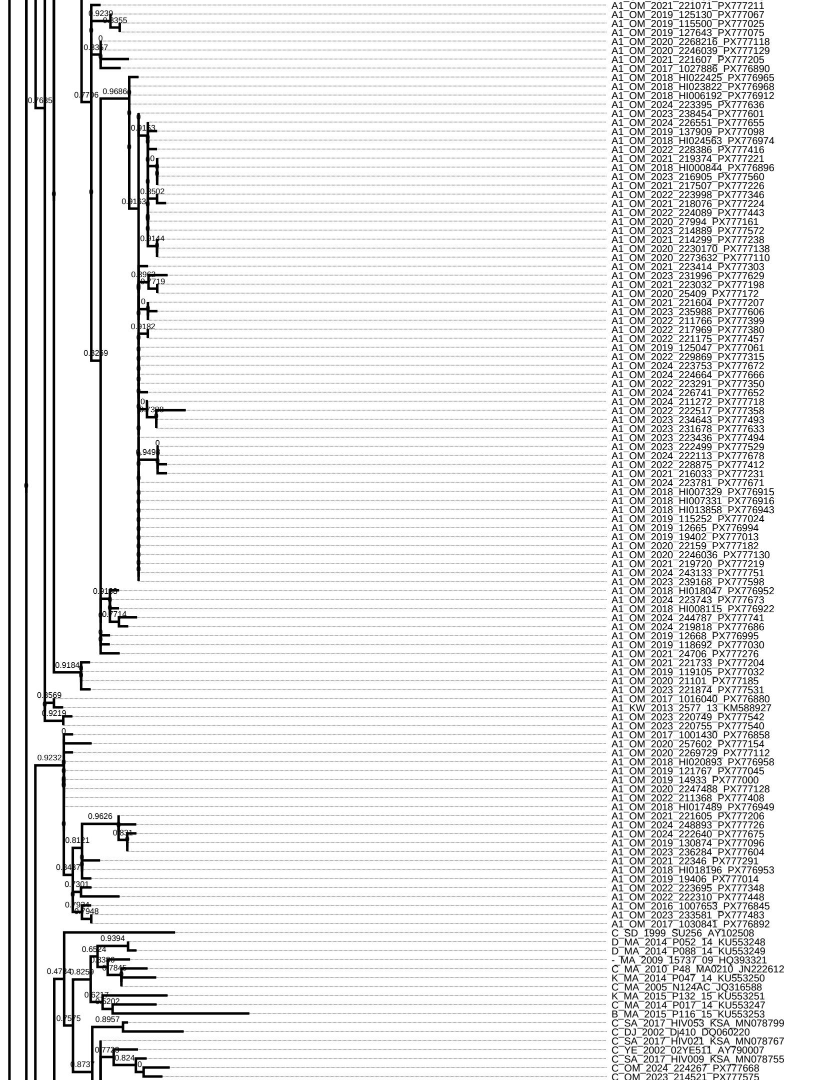
0.9239
0.8355
0
0.8357
0.7635
0.7706
0.9686
0.9153
0
0.8502
0.9153
0.9144
0.8962
0.7719
0
0.9182
0.8269
0
0.7388
0
0.9498
0.9188
0.7714
0.9184
0.8569
0.9219
0
0.9232
0.9626
0.831
0.8121
0.8487
0.7301
0.7924
0.7948
0.9394
0.6524
0.8386
0.4784
0.8259
0.7845
0.6217
0.5202
0.7575
0.8957
0.7729
0.824
0
0.8737
A1_OM_2021_221071_PX777211
A1_OM_2019_125130_PX777067
A1_OM_2019_115500_PX777025
A1_OM_2019_127643_PX777075
A1_OM_2020_2268216_PX777118
A1_OM_2020_2246039_PX777129
A1_OM_2021_221607_PX777205
A1_OM_2017_1027886_PX776890
A1_OM_2018_HI022425_PX776965
A1_OM_2018_HI023822_PX776968
A1_OM_2018_HI006192_PX776912
A1_OM_2024_223395_PX777636
A1_OM_2023_238454_PX777601
A1_OM_2024_226551_PX777655
A1_OM_2019_137909_PX777098
A1_OM_2018_HI024563_PX776974
A1_OM_2022_228386_PX777416
A1_OM_2021_219374_PX777221
A1_OM_2018_HI000844_PX776896
A1_OM_2023_216905_PX777560
A1_OM_2021_217507_PX777226
A1_OM_2022_223998_PX777346
A1_OM_2021_218076_PX777224
A1_OM_2022_224089_PX777443
A1_OM_2020_27994_PX777161
A1_OM_2023_214889_PX777572
A1_OM_2021_214299_PX777238
A1_OM_2020_2230170_PX777138
A1_OM_2020_2273632_PX777110
A1_OM_2021_223414_PX777303
A1_OM_2023_231996_PX777629
A1_OM_2021_223032_PX777198
A1_OM_2020_25409_PX777172
A1_OM_2021_221604_PX777207
A1_OM_2023_235988_PX777606
A1_OM_2022_211766_PX777399
A1_OM_2022_217969_PX777380
A1_OM_2022_221175_PX777457
A1_OM_2019_125047_PX777061
A1_OM_2022_229869_PX777315
A1_OM_2024_223753_PX777672
A1_OM_2024_224664_PX777666
A1_OM_2022_223291_PX777350
A1_OM_2024_226741_PX777652
A1_OM_2024_211272_PX777718
A1_OM_2022_222517_PX777358
A1_OM_2023_234643_PX777493
A1_OM_2023_231678_PX777633
A1_OM_2023_223436_PX777494
A1_OM_2023_222499_PX777529
A1_OM_2024_222113_PX777678
A1_OM_2022_228875_PX777412
A1_OM_2021_216033_PX777231
A1_OM_2024_223781_PX777671
A1_OM_2018_HI007329_PX776915
A1_OM_2018_HI007331_PX776916
A1_OM_2018_HI013858_PX776943
A1_OM_2019_115252_PX777024
A1_OM_2019_12665_PX776994
A1_OM_2019_19402_PX777013
A1_OM_2020_22159_PX777182
A1_OM_2020_2246036_PX777130
A1_OM_2021_219720_PX777219
A1_OM_2024_243133_PX777751
A1_OM_2023_239168_PX777598
A1_OM_2018_HI018047_PX776952
A1_OM_2024_223743_PX777673
A1_OM_2018_HI008115_PX776922
A1_OM_2024_244787_PX777741
A1_OM_2024_219818_PX777686
A1_OM_2019_12668_PX776995
A1_OM_2019_118692_PX777030
A1_OM_2021_24706_PX777276
A1_OM_2021_221733_PX777204
A1_OM_2019_119105_PX777032
A1_OM_2020_21101_PX777185
A1_OM_2023_221874_PX777531
A1_OM_2017_1016040_PX776880
A1_KW_2013_2577_13_KM588927
A1_OM_2023_220749_PX777542
A1_OM_2023_220755_PX777540
A1_OM_2017_1001430_PX776858
A1_OM_2020_257602_PX777154
A1_OM_2020_2269729_PX777112
A1_OM_2018_HI020893_PX776958
A1_OM_2019_121767_PX777045
A1_OM_2019_14933_PX777000
A1_OM_2020_2247488_PX777128
A1_OM_2022_211368_PX777408
A1_OM_2018_HI017489_PX776949
A1_OM_2021_221605_PX777206
A1_OM_2024_248893_PX777726
A1_OM_2024_222640_PX777675
A1_OM_2019_130874_PX777096
A1_OM_2023_236284_PX777604
A1_OM_2021_22346_PX777291
A1_OM_2018_HI018196_PX776953
A1_OM_2019_19406_PX777014
A1_OM_2022_223695_PX777348
A1_OM_2022_222310_PX777448
A1_OM_2016_1007653_PX776845
A1_OM_2023_233581_PX777483
A1_OM_2017_1030841_PX776892
C_SD_1999_SU256_AY102508
D_MA_2014_P052_14_KU553248
D_MA_2014_P088_14_KU553249
-_MA_2009_15737_09_HQ393321
C_MA_2010_P48_MA0210_JN222612
K_MA_2014_P047_14_KU553250
C_MA_2005_N124AC_JQ316588
K_MA_2015_P132_15_KU553251
C_MA_2014_P017_14_KU553247
B_MA_2015_P116_15_KU553253
C_SA_2017_HIV053_KSA_MN078799
C_DJ_2002_Dj410_DQ060220
C_SA_2017_HIV021_KSA_MN078767
C_YE_2002_02YE511_AY790007
C_SA_2017_HIV009_KSA_MN078755
C_OM_2024_224267_PX777668
C_OM_2023_214521_PX777575

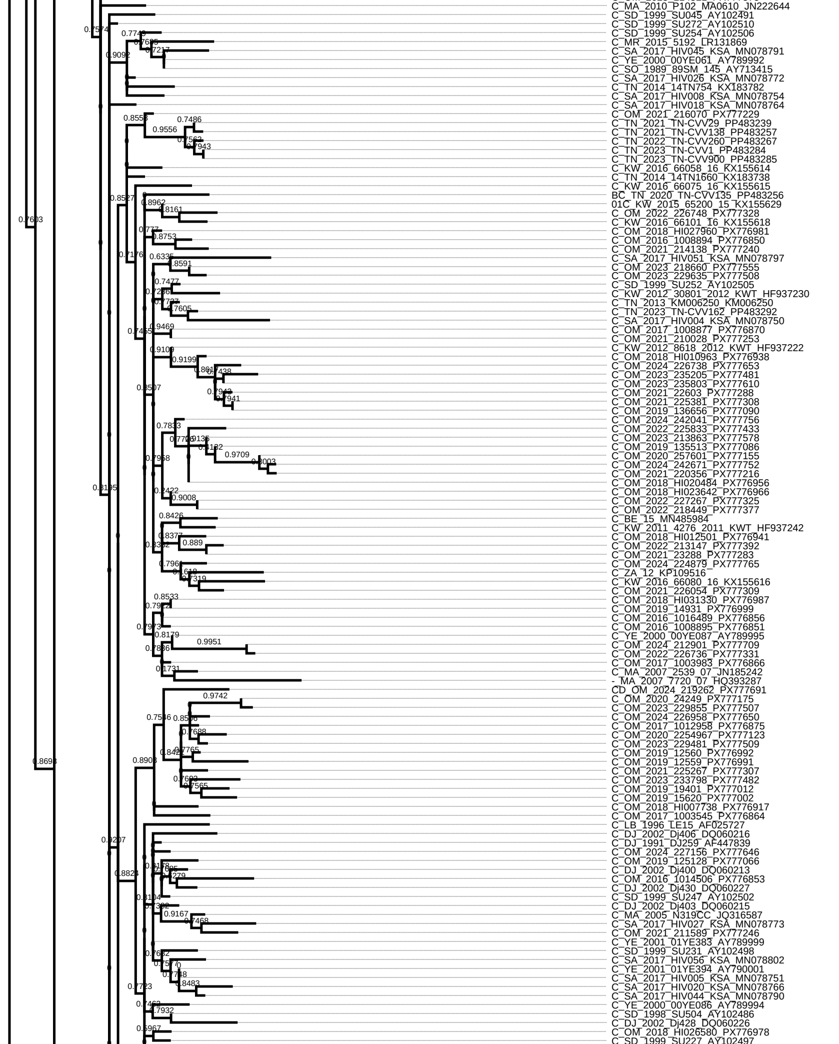

C_MA_2010_P102_MA0610_JN222644
C_SD_1999_SU045_AY102491
C_SD_1999_SU272_AY102510
C_SD_1999_SU254_AY102506
C_MR_2015_5192_LR131869
C_SA_2017_HIV045_KSA_MN078791
C_YE_2000_00YE061_AY789992
C_SO_1989_89SM_145_AY713415
C_SA_2017_HIV026_KSA_MN078772
C_TN_2014_14TN754_KX183782
C_SA_2017_HIV008_KSA_MN078754
C_SA_2017_HIV018_KSA_MN078764
C_OM_2021_216070_PX777229
C_TN_2021_TN-CVV29_PP483239
C_TN_2021_TN-CVV138_PP483257
C_TN_2022_TN-CVV260_PP483267
C_TN_2023_TN-CVV1_PP483284
C_TN_2023_TN-CVV900_PP483285
C_KW_2016_66058_16_KX155614
C_TN_2014_14TN1660_KX183738
C_KW_2016_66075_16_KX155615
BC_TN_2020_TN-CVV135_PP483256
01C_KW_2015_65200_15_KX155629
C_OM_2022_226748_PX777328
C_KW_2016_66101_16_KX155618
C_OM_2018_HI027960_PX776981
C_OM_2016_1008894_PX776850
C_OM_2021_214138_PX777240
C_SA_2017_HIV051_KSA_MN078797
C_OM_2023_218660_PX777555
C_OM_2023_229635_PX777508
C_SD_1999_SU252_AY102505
C_KW_2012_30801_2012_KWT_HF937230
C_TN_2013_KM006250_KM006250
C_TN_2023_TN-CVV162_PP483292
C_SA_2017_HIV004_KSA_MN078750
C_OM_2017_1008877_PX776870
C_OM_2021_210028_PX777253
C_KW_2012_8618_2012_KWT_HF937222
C_OM_2018_HI010963_PX776938
C_OM_2024_226738_PX777653
C_OM_2023_235205_PX777481
C_OM_2023_235803_PX777610
C_OM_2021_22603_PX777288
C_OM_2021_225381_PX777308
C_OM_2019_136656_PX777090
C_OM_2024_242041_PX777756
C_OM_2022_225833_PX777433
C_OM_2023_213863_PX777578
C_OM_2019_135513_PX777086
C_OM_2020_257601_PX777155
C_OM_2024_242671_PX777752
C_OM_2021_220356_PX777216
C_OM_2018_HI020484_PX776956
C_OM_2018_HI023642_PX776966
C_OM_2022_227267_PX777325
C_OM_2022_218449_PX777377
C_BE_15_MN485984
C_KW_2011_4276_2011_KWT_HF937242
C_OM_2018_HI012501_PX776941
C_OM_2022_213147_PX777392
C_OM_2021_23288_PX777283
C_OM_2024_224879_PX777765
C_ZA_12_KP109516
C_KW_2016_66080_16_KX155616
C_OM_2021_226054_PX777309
C_OM_2018_HI031330_PX776987
C_OM_2019_14931_PX776999
C_OM_2016_1016489_PX776856
C_OM_2016_1008895_PX776851
C_YE_2000_00YE087_AY789995
C_OM_2024_212901_PX777709
C_OM_2022_226736_PX777331
C_OM_2017_1003983_PX776866
C_MA_2007_2539_07_JN185242
-_MA_2007_7720_07_HQ393287
CD_OM_2024_219262_PX777691
C_OM_2020_24249_PX777175
C_OM_2023_229855_PX777507
C_OM_2024_226958_PX777650
C_OM_2017_1012958_PX776875
C_OM_2020_2254967_PX777123
C_OM_2023_229481_PX777509
C_OM_2019_12560_PX776992
C_OM_2019_12559_PX776991
C_OM_2021_225267_PX777307
C_OM_2023_233798_PX777482
C_OM_2019_19401_PX777012
C_OM_2019_15620_PX777002
C_OM_2018_HI007738_PX776917
C_OM_2017_1003545_PX776864
C_LB_1996_LE15_AF025727
C_DJ_2002_Dj406_DQ060216
C_DJ_1991_DJ259_AF447839
C_OM_2024_227156_PX777646
C_OM_2019_125128_PX777066
C_DJ_2002_Dj400_DQ060213
C_OM_2016_1014506_PX776853
C_DJ_2002_Dj430_DQ060227
C_SD_1999_SU247_AY102502
C_DJ_2002_Dj403_DQ060215
C_MA_2005_N319CC_JQ316587
C_SA_2017_HIV027_KSA_MN078773
C_OM_2021_211589_PX777246
C_YE_2001_01YE383_AY789999
C_SD_1999_SU231_AY102498
C_SA_2017_HIV056_KSA_MN078802
C_YE_2001_01YE394_AY790001
C_SA_2017_HIV005_KSA_MN078751
C_SA_2017_HIV020_KSA_MN078766
C_SA_2017_HIV044_KSA_MN078790
C_YE_2000_00YE086_AY789994
C_SD_1998_SU504_AY102486
C_DJ_2002_Dj428_DQ060226
C_OM_2018_HI026580_PX776978
C_SD_1999_SU227_AY102497

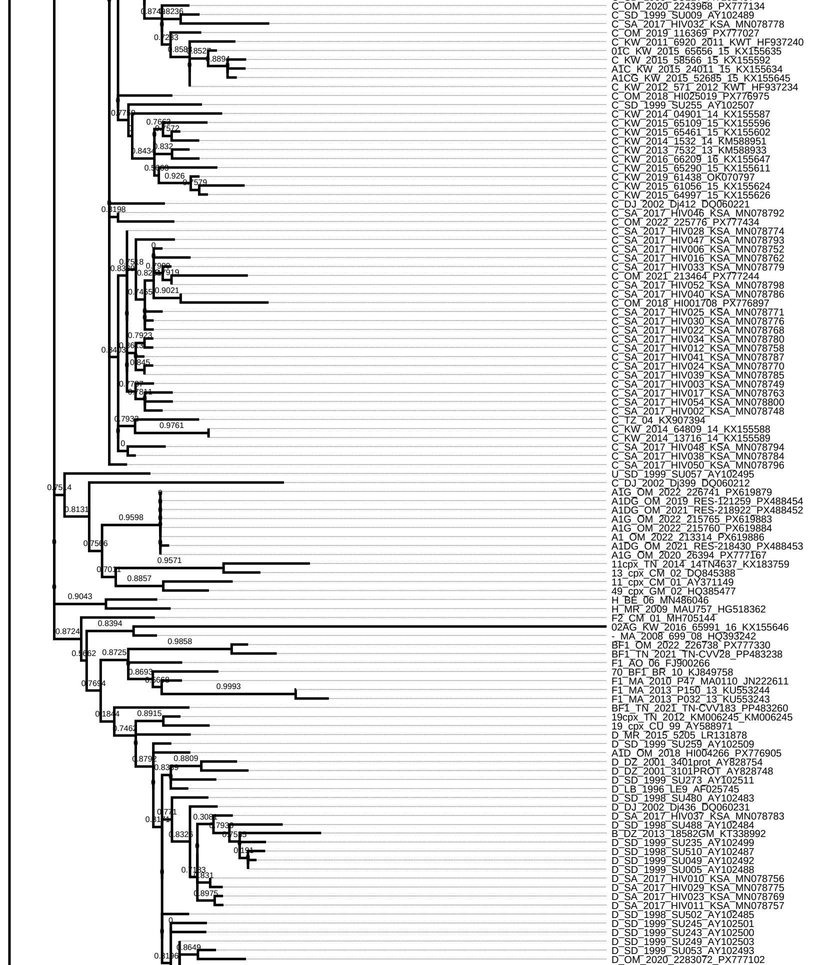

C_OM_2020_2243968_PX777134
C_SD_1999_SU009_AY102489
C_SA_2017_HIV032_KSA_MN078778
C_OM_2019_116369_PX777027
C_KW_2011_6920_2011_KWT_HF937240
01C_KW_2015_65656_15_KX155635
C_KW_2015_58566_15_KX155592
A1C_KW_2015_24011_15_KX155634
A1CG_KW_2015_52685_15_KX155645
C_KW_2012_571_2012_KWT_HF937234
C_OM_2018_HI025019_PX776975
C_SD_1999_SU255_AY102507
C_KW_2014_04901_14_KX155587
C_KW_2015_65109_15_KX155596
C_KW_2015_65461_15_KX155602
C_KW_2014_1532_14_KM588951
C_KW_2013_7532_13_KM588933
C_KW_2016_66209_16_KX155647
C_KW_2015_65290_15_KX155611
C_KW_2019_61438_OK070797
C_KW_2015_61056_15_KX155624
C_KW_2015_64997_15_KX155626
C_DJ_2002_Dj412_DQ060221
C_SA_2017_HIV046_KSA_MN078792
C_OM_2022_225776_PX777434
C_SA_2017_HIV028_KSA_MN078774
C_SA_2017_HIV047_KSA_MN078793
C_SA_2017_HIV006_KSA_MN078752
C_SA_2017_HIV016_KSA_MN078762
C_SA_2017_HIV033_KSA_MN078779
C_OM_2021_213464_PX777244
C_SA_2017_HIV052_KSA_MN078798
C_SA_2017_HIV040_KSA_MN078786
C_OM_2018_HI001708_PX776897
C_SA_2017_HIV025_KSA_MN078771
C_SA_2017_HIV030_KSA_MN078776
C_SA_2017_HIV022_KSA_MN078768
C_SA_2017_HIV034_KSA_MN078780
C_SA_2017_HIV012_KSA_MN078758
C_SA_2017_HIV041_KSA_MN078787
C_SA_2017_HIV024_KSA_MN078770
C_SA_2017_HIV039_KSA_MN078785
C_SA_2017_HIV003_KSA_MN078749
C_SA_2017_HIV017_KSA_MN078763
C_SA_2017_HIV054_KSA_MN078800
C_SA_2017_HIV002_KSA_MN078748
C_TZ_04_KX907394
C_KW_2014_64809_14_KX155588
C_KW_2014_13716_14_KX155589
C_SA_2017_HIV048_KSA_MN078794
C_SA_2017_HIV038_KSA_MN078784
C_SA_2017_HIV050_KSA_MN078796
U_SD_1999_SU057_AY102495
C_DJ_2002_Dj399_DQ060212
A1G_OM_2022_226741_PX619879
A1DG_OM_2019_RES-121259_PX488454
A1DG_OM_2021_RES-218922_PX488452
A1G_OM_2022_215765_PX619883
A1G_OM_2022_215760_PX619884
A1_OM_2022_213314_PX619886
A1DG_OM_2021_RES-218430_PX488453
A1G_OM_2020_26394_PX777167
11cpx_TN_2014_14TN4637_KX183759
13_cpx_CM_02_DQ845388
11_cpx_CM_01_AY371149
49_cpx_GM_02_HQ385477
H_BE_06_MN486046
H_MR_2009_MAU757_HG518362
F2_CM_01_MH705144
02AG_KW_2016_65991_16_KX155646
-_MA_2008_699_08_HQ393242
BF1_OM_2022_226738_PX777330
BF1_TN_2021_TN-CVV28_PP483238
F1_AO_06_FJ900266
70_BF1_BR_10_KJ849758
F1_MA_2010_P47_MA0110_JN222611
F1_MA_2013_P150_13_KU553244
F1_MA_2013_P032_13_KU553243
BF1_TN_2021_TN-CVV183_PP483260
19cpx_TN_2012_KM006245_KM006245
19_cpx_CU_99_AY588971
D_MR_2015_5205_LR131878
D_SD_1999_SU259_AY102509
A1D_OM_2018_HI004266_PX776905
D_DZ_2001_3401prot_AY828754
D_DZ_2001_3101PROT_AY828748
D_SD_1999_SU273_AY102511
D_LB_1996_LE9_AF025745
D_SD_1998_SU480_AY102483
D_DJ_2002_Dj436_DQ060231
D_SA_2017_HIV037_KSA_MN078783
D_SD_1998_SU488_AY102484
B_DZ_2013_18582GM_KT338992
D_SD_1999_SU235_AY102499
D_SD_1998_SU510_AY102487
D_SD_1999_SU049_AY102492
D_SD_1999_SU005_AY102488
D_SA_2017_HIV010_KSA_MN078756
D_SA_2017_HIV029_KSA_MN078775
D_SA_2017_HIV023_KSA_MN078769
D_SA_2017_HIV011_KSA_MN078757
D_SD_1998_SU502_AY102485
D_SD_1999_SU245_AY102501
D_SD_1999_SU243_AY102500
D_SD_1999_SU249_AY102503
D_SD_1999_SU053_AY102493
D_OM_2020_2283072_PX777102

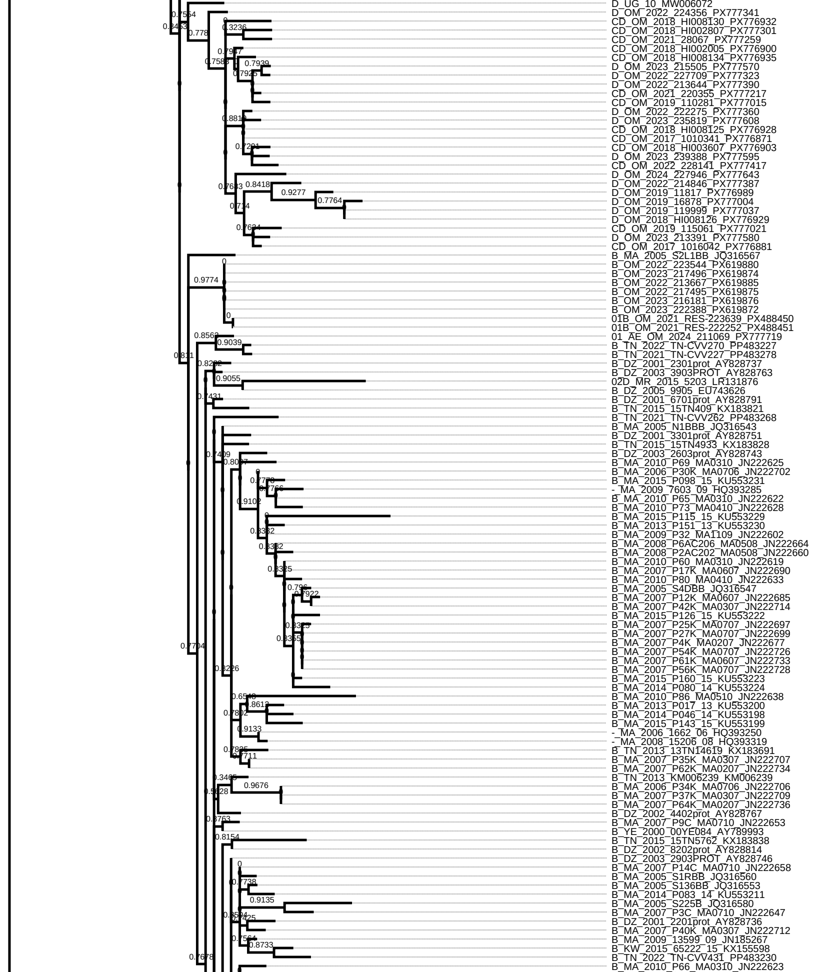
D_UG_10_MW006072
D_OM_2022_224356_PX777341
CD_OM_2018_HI008130_PX776932
CD_OM_2018_HI002807_PX777301
CD_OM_2021_28067_PX777259
CD_OM_2018_HI002005_PX776900
CD_OM_2018_HI008134_PX776935
D_OM_2023_215505_PX777570
D_OM_2022_227709_PX777323
D_OM_2022_213644_PX777390
CD_OM_2021_220355_PX777217
CD_OM_2019_110281_PX777015
D_OM_2022_222275_PX777360
D_OM_2023_235819_PX777608
CD_OM_2018_HI008125_PX776928
CD_OM_2017_1010341_PX776871
CD_OM_2018_HI003607_PX776903
D_OM_2023_239388_PX777595
CD_OM_2022_228141_PX777417
D_OM_2024_227946_PX777643
D_OM_2022_214846_PX777387
D_OM_2019_11817_PX776989
D_OM_2019_16878_PX777004
D_OM_2019_119999_PX777037
D_OM_2018_HI008126_PX776929
CD_OM_2019_115061_PX777021
D_OM_2023_213391_PX777580
CD_OM_2017_1016042_PX776881
B_MA_2005_S2L1BB_JQ316567
B_OM_2022_223544_PX619880
B_OM_2023_217496_PX619874
B_OM_2022_213667_PX619885
B_OM_2022_217495_PX619875
B_OM_2023_216181_PX619876
B_OM_2023_222388_PX619872
01B_OM_2021_RES-223639_PX488450
01B_OM_2021_RES-222252_PX488451
01_AE_OM_2024_211069_PX777719
B_TN_2022_TN-CVV270_PP483227
B_TN_2021_TN-CVV227_PP483278
B_DZ_2001_2301prot_AY828737
B_DZ_2003_3903PROT_AY828763
02D_MR_2015_5203_LR131876
B_DZ_2005_9905_EU743626
B_DZ_2001_6701prot_AY828791
B_TN_2015_15TN409_KX183821
B_TN_2021_TN-CVV262_PP483268
B_MA_2005_N1BBB_JQ316543
B_DZ_2001_3301prot_AY828751
B_TN_2015_15TN4933_KX183828
B_DZ_2003_2603prot_AY828743
B_MA_2010_P69_MA0310_JN222625
B_MA_2006_P30K_MA0706_JN222702
B_MA_2015_P098_15_KU553231
-_MA_2009_7603_09_HQ393285
B_MA_2010_P65_MA0310_JN222622
B_MA_2010_P73_MA0410_JN222628
B_MA_2015_P115_15_KU553229
B_MA_2013_P151_13_KU553230
B_MA_2009_P32_MA1109_JN222602
B_MA_2008_P6AC206_MA0508_JN222664
B_MA_2008_P2AC202_MA0508_JN222660
B_MA_2010_P60_MA0310_JN222619
B_MA_2007_P17K_MA0607_JN222690
B_MA_2010_P80_MA0410_JN222633
B_MA_2005_S4DBB_JQ316547
B_MA_2007_P12K_MA0607_JN222685
B_MA_2007_P42K_MA0307_JN222714
B_MA_2015_P126_15_KU553222
B_MA_2007_P25K_MA0707_JN222697
B_MA_2007_P27K_MA0707_JN222699
B_MA_2007_P4K_MA0207_JN222677
B_MA_2007_P54K_MA0707_JN222726
B_MA_2007_P61K_MA0607_JN222733
B_MA_2007_P56K_MA0707_JN222728
B_MA_2015_P160_15_KU553223
B_MA_2014_P080_14_KU553224
B_MA_2010_P86_MA0510_JN222638
B_MA_2013_P017_13_KU553200
B_MA_2014_P046_14_KU553198
B_MA_2015_P143_15_KU553199
-_MA_2006_1662_06_HQ393250
-_MA_2008_15206_08_HQ393319
B_TN_2013_13TN14619_KX183691
B_MA_2007_P35K_MA0307_JN222707
B_MA_2007_P62K_MA0207_JN222734
B_TN_2013_KM006239_KM006239
B_MA_2006_P34K_MA0706_JN222706
B_MA_2007_P37K_MA0307_JN222709
B_MA_2007_P64K_MA0207_JN222736
B_DZ_2002_4402prot_AY828767
B_MA_2007_P9C_MA0710_JN222653
B_YE_2000_00YE084_AY789993
B_TN_2015_15TN5762_KX183838
B_DZ_2002_8202prot_AY828814
B_DZ_2003_2903PROT_AY828746
B_MA_2007_P14C_MA0710_JN222658
B_MA_2005_S1RBB_JQ316560
B_MA_2005_S136BB_JQ316553
B_MA_2014_P083_14_KU553211
B_MA_2005_S225B_JQ316580
B_MA_2007_P3C_MA0710_JN222647
B_DZ_2001_2201prot_AY828736
B_MA_2007_P40K_MA0307_JN222712
B_MA_2009_13599_09_JN185267
B_KW_2015_65222_15_KX155598
B_TN_2022_TN-CVV431_PP483230
B_MA_2010_P66_MA0310_JN222623

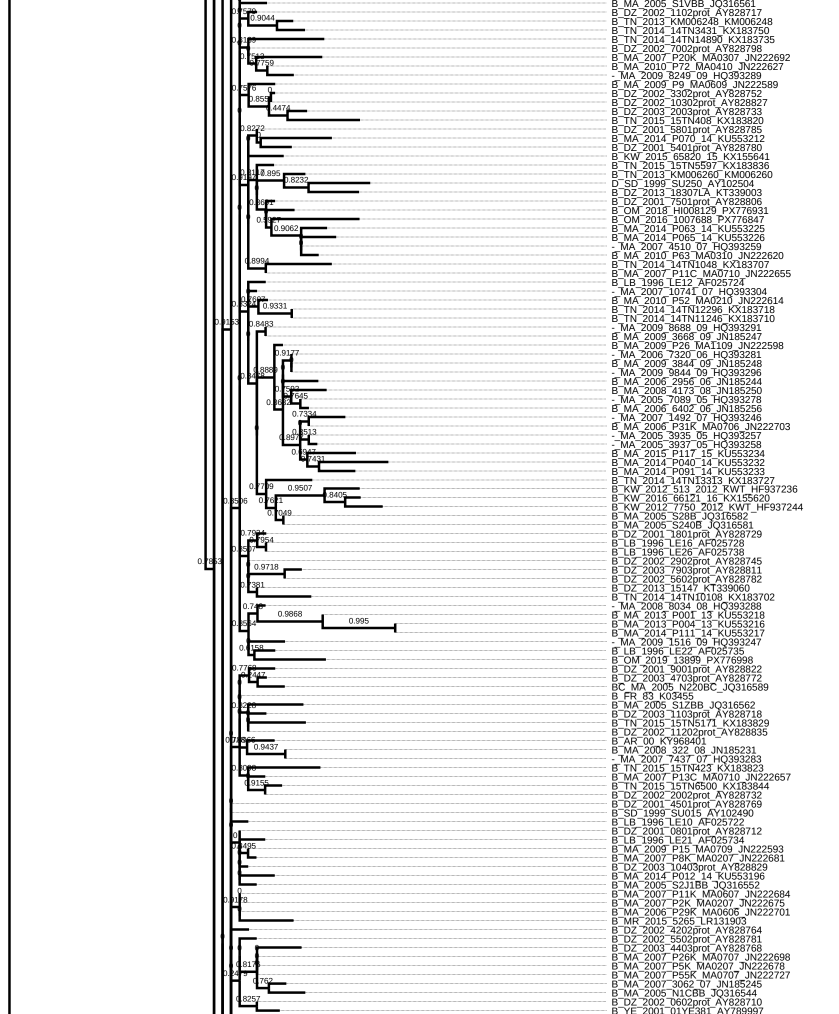
B_MA_2005_S1VBB_JQ316561
B_DZ_2002_1102prot_AY828717
B_TN_2013_KM006248_KM006248
B_TN_2014_14TN3431_KX183750
B_TN_2014_14TN14890_KX183735
B_DZ_2002_7002prot_AY828798
B_MA_2007_P20K_MA0307_JN222692
B_MA_2010_P72_MA0410_JN222627
-_MA_2009_8249_09_HQ393289
B_MA_2009_P9_MA0609_JN222589
B_DZ_2002_3302prot_AY828752
B_DZ_2002_10302prot_AY828827
B_DZ_2003_2003prot_AY828733
B_TN_2015_15TN408_KX183820
B_DZ_2001_5801prot_AY828785
B_MA_2014_P070_14_KU553212
B_DZ_2001_5401prot_AY828780
B_KW_2015_65820_15_KX155641
B_TN_2015_15TN5597_KX183836
B_TN_2013_KM006260_KM006260
D_SD_1999_SU250_AY102504
B_DZ_2013_18307LA_KT339003
B_DZ_2001_7501prot_AY828806
B_OM_2018_HI008129_PX776931
B_OM_2016_1007688_PX776847
B_MA_2014_P063_14_KU553225
B_MA_2014_P065_14_KU553226
-_MA_2007_4510_07_HQ393259
B_MA_2010_P63_MA0310_JN222620
B_TN_2014_14TN1048_KX183707
B_MA_2007_P11C_MA0710_JN222655
B_LB_1996_LE12_AF025724
-_MA_2007_10741_07_HQ393304
B_MA_2010_P52_MA0210_JN222614
B_TN_2014_14TN12296_KX183718
B_TN_2014_14TN11246_KX183710
-_MA_2009_8688_09_HQ393291
B_MA_2009_3668_09_JN185247
B_MA_2009_P26_MA1109_JN222598
-_MA_2006_7320_06_HQ393281
B_MA_2009_3844_09_JN185248
-_MA_2009_9844_09_HQ393296
B_MA_2006_2956_06_JN185244
B_MA_2008_4173_08_JN185250
-_MA_2005_7089_05_HQ393278
B_MA_2006_6402_06_JN185256
-_MA_2007_1492_07_HQ393246
B_MA_2006_P31K_MA0706_JN222703
-_MA_2005_3935_05_HQ393257
-_MA_2005_3937_05_HQ393258
B_MA_2015_P117_15_KU553234
B_MA_2014_P040_14_KU553232
B_MA_2014_P091_14_KU553233
B_TN_2014_14TN13313_KX183727
B_KW_2012_513_2012_KWT_HF937236
B_KW_2016_66121_16_KX155620
B_KW_2012_7750_2012_KWT_HF937244
B_MA_2005_S28B_JQ316582
B_MA_2005_S240B_JQ316581
B_DZ_2001_1801prot_AY828729
B_LB_1996_LE16_AF025728
B_LB_1996_LE26_AF025738
B_DZ_2002_2902prot_AY828745
B_DZ_2003_7903prot_AY828811
B_DZ_2002_5602prot_AY828782
B_DZ_2013_15147_KT339060
B_TN_2014_14TN10108_KX183702
-_MA_2008_8034_08_HQ393288
B_MA_2013_P001_13_KU553218
B_MA_2013_P004_13_KU553216
B_MA_2014_P111_14_KU553217
-_MA_2009_1516_09_HQ393247
B_LB_1996_LE22_AF025735
B_OM_2019_13899_PX776998
B_DZ_2001_9001prot_AY828822
B_DZ_2003_4703prot_AY828772
BC_MA_2005_N220BC_JQ316589
B_FR_83_K03455
B_MA_2005_S1ZBB_JQ316562
B_DZ_2003_1103prot_AY828718
B_TN_2015_15TN5171_KX183829
B_DZ_2002_11202prot_AY828835
B_AR_00_KY968401
B_MA_2008_322_08_JN185231
-_MA_2007_7437_07_HQ393283
B_TN_2015_15TN423_KX183823
B_MA_2007_P13C_MA0710_JN222657
B_TN_2015_15TN6500_KX183844
B_DZ_2002_2002prot_AY828732
B_DZ_2001_4501prot_AY828769
B_SD_1999_SU015_AY102490
B_LB_1996_LE10_AF025722
B_DZ_2001_0801prot_AY828712
B_LB_1996_LE21_AF025734
B_MA_2009_P15_MA0709_JN222593
B_MA_2007_P8K_MA0207_JN222681
B_DZ_2003_10403prot_AY828829
B_MA_2014_P012_14_KU553196
B_MA_2005_S2J1BB_JQ316552
B_MA_2007_P11K_MA0607_JN222684
B_MA_2007_P2K_MA0207_JN222675
B_MA_2006_P29K_MA0606_JN222701
B_MR_2015_5265_LR131903
B_DZ_2002_4202prot_AY828764
B_DZ_2002_5502prot_AY828781
B_DZ_2003_4403prot_AY828768
B_MA_2007_P26K_MA0707_JN222698
B_MA_2007_P5K_MA0207_JN222678
B_MA_2007_P55K_MA0707_JN222727
B_MA_2007_3062_07_JN185245
B_MA_2005_N1CBB_JQ316544
B_DZ_2002_0602prot_AY828710
B_YE_2001_01YE381_AY789997

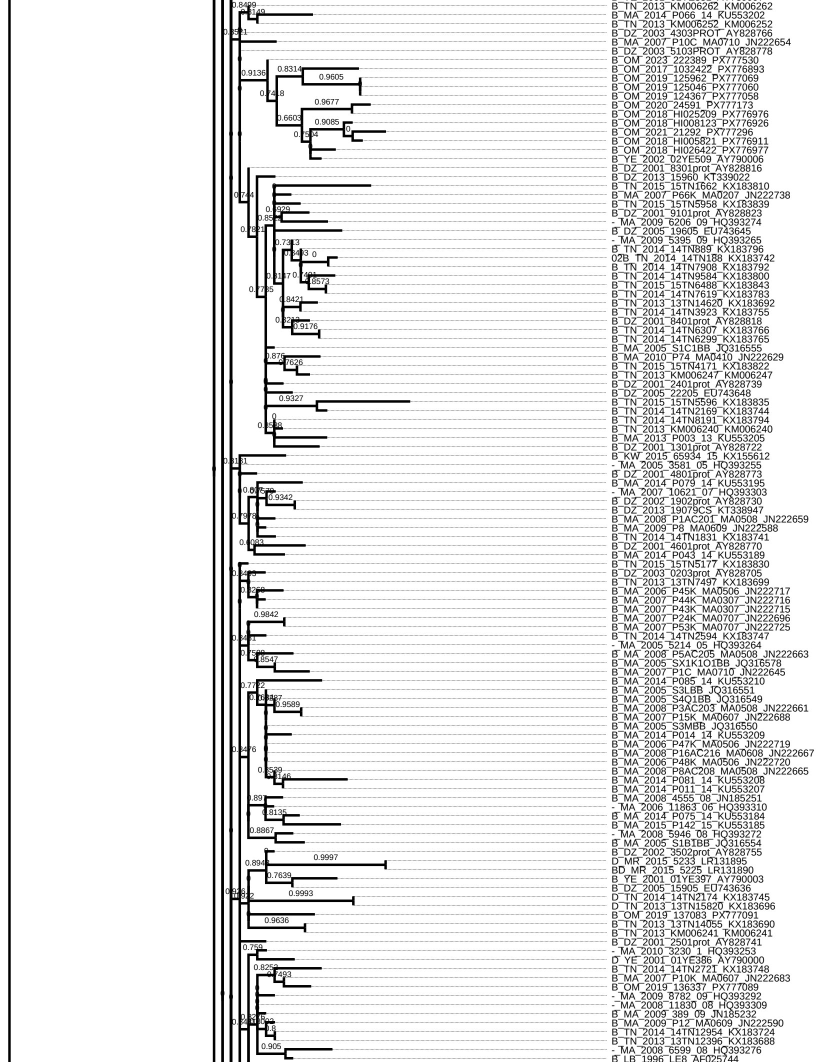
B_TN_2013_KM006262_KM006262
B_MA_2014_P066_14_KU553202
B_TN_2013_KM006252_KM006252
B_DZ_2003_4303PROT_AY828766
B_MA_2007_P10C_MA0710_JN222654
B_DZ_2003_5103PROT_AY828778
B_OM_2023_222389_PX777530
B_OM_2017_1032422_PX776893
B_OM_2019_125962_PX777069
B_OM_2019_125046_PX777060
B_OM_2019_124367_PX777058
B_OM_2020_24591_PX777173
B_OM_2018_HI025209_PX776976
B_OM_2018_HI008123_PX776926
B_OM_2021_21292_PX777296
B_OM_2018_HI005821_PX776911
B_OM_2018_HI026422_PX776977
B_YE_2002_02YE509_AY790006
B_DZ_2001_8301prot_AY828816
B_DZ_2013_15960_KT339022
B_TN_2015_15TN1662_KX183810
B_MA_2007_P66K_MA0207_JN222738
B_TN_2015_15TN5958_KX183839
B_DZ_2001_9101prot_AY828823
-_MA_2009_6206_09_HQ393274
B_DZ_2005_19605_EU743645
-_MA_2009_5395_09_HQ393265
B_TN_2014_14TN889_KX183796
02B_TN_2014_14TN188_KX183742
B_TN_2014_14TN7908_KX183792
B_TN_2014_14TN9584_KX183800
B_TN_2015_15TN6488_KX183843
B_TN_2014_14TN7619_KX183783
B_TN_2013_13TN14620_KX183692
B_TN_2014_14TN3923_KX183755
B_DZ_2001_8401prot_AY828818
B_TN_2014_14TN6307_KX183766
B_TN_2014_14TN6299_KX183765
B_MA_2005_S1C1BB_JQ316555
B_MA_2010_P74_MA0410_JN222629
B_TN_2015_15TN4171_KX183822
B_TN_2013_KM006247_KM006247
B_DZ_2001_2401prot_AY828739
B_DZ_2005_22205_EU743648
B_TN_2015_15TN5596_KX183835
B_TN_2014_14TN2169_KX183744
B_TN_2014_14TN8191_KX183794
B_TN_2013_KM006240_KM006240
B_MA_2013_P003_13_KU553205
B_DZ_2001_1301prot_AY828722
B_KW_2015_65934_15_KX155612
-_MA_2005_3581_05_HQ393255
B_DZ_2001_4801prot_AY828773
B_MA_2014_P079_14_KU553195
-_MA_2007_10621_07_HQ393303
B_DZ_2002_1902prot_AY828730
B_DZ_2013_19079CS_KT338947
B_MA_2008_P1AC201_MA0508_JN222659
B_MA_2009_P8_MA0609_JN222588
B_TN_2014_14TN1831_KX183741
B_DZ_2001_4601prot_AY828770
B_MA_2014_P043_14_KU553189
B_TN_2015_15TN5177_KX183830
B_DZ_2003_0203prot_AY828705
B_TN_2013_13TN7497_KX183699
B_MA_2006_P45K_MA0506_JN222717
B_MA_2007_P44K_MA0307_JN222716
B_MA_2007_P43K_MA0307_JN222715
B_MA_2007_P24K_MA0707_JN222696
B_MA_2007_P53K_MA0707_JN222725
B_TN_2014_14TN2594_KX183747
-_MA_2005_5214_05_HQ393264
B_MA_2008_P5AC205_MA0508_JN222663
B_MA_2005_SX1K1O1BB_JQ316578
B_MA_2007_P1C_MA0710_JN222645
B_MA_2014_P085_14_KU553210
B_MA_2005_S3LBB_JQ316551
B_MA_2005_S4Q1BB_JQ316549
B_MA_2008_P3AC203_MA0508_JN222661
B_MA_2007_P15K_MA0607_JN222688
B_MA_2005_S3MBB_JQ316550
B_MA_2014_P014_14_KU553209
B_MA_2006_P47K_MA0506_JN222719
B_MA_2008_P16AC216_MA0608_JN222667
B_MA_2006_P48K_MA0506_JN222720
B_MA_2008_P8AC208_MA0508_JN222665
B_MA_2014_P081_14_KU553208
B_MA_2014_P011_14_KU553207
B_MA_2008_4555_08_JN185251
-_MA_2006_11863_06_HQ393310
B_MA_2014_P075_14_KU553184
B_MA_2015_P142_15_KU553185
-_MA_2008_5946_08_HQ393272
B_MA_2005_S1B1BB_JQ316554
B_DZ_2002_3502prot_AY828755
D_MR_2015_5233_LR131895
BD_MR_2015_5225_LR131890
B_YE_2001_01YE397_AY790003
B_DZ_2005_15905_EU743636
D_TN_2014_14TN2174_KX183745
D_TN_2013_13TN15820_KX183696
B_OM_2019_137083_PX777091
B_TN_2013_13TN14055_KX183690
B_TN_2013_KM006241_KM006241
B_DZ_2001_2501prot_AY828741
-_MA_2010_3230_1_HQ393253
D_YE_2001_01YE386_AY790000
B_TN_2014_14TN2721_KX183748
B_MA_2007_P10K_MA0607_JN222683
B_OM_2019_136337_PX777089
-_MA_2009_8782_09_HQ393292
-_MA_2008_11830_08_HQ393309
B_MA_2009_389_09_JN185232
B_MA_2009_P12_MA0609_JN222590
B_TN_2014_14TN12954_KX183724
B_TN_2013_13TN12396_KX183688
-_MA_2008_6599_08_HQ393276
B_LB_1996_LE8_AF025744

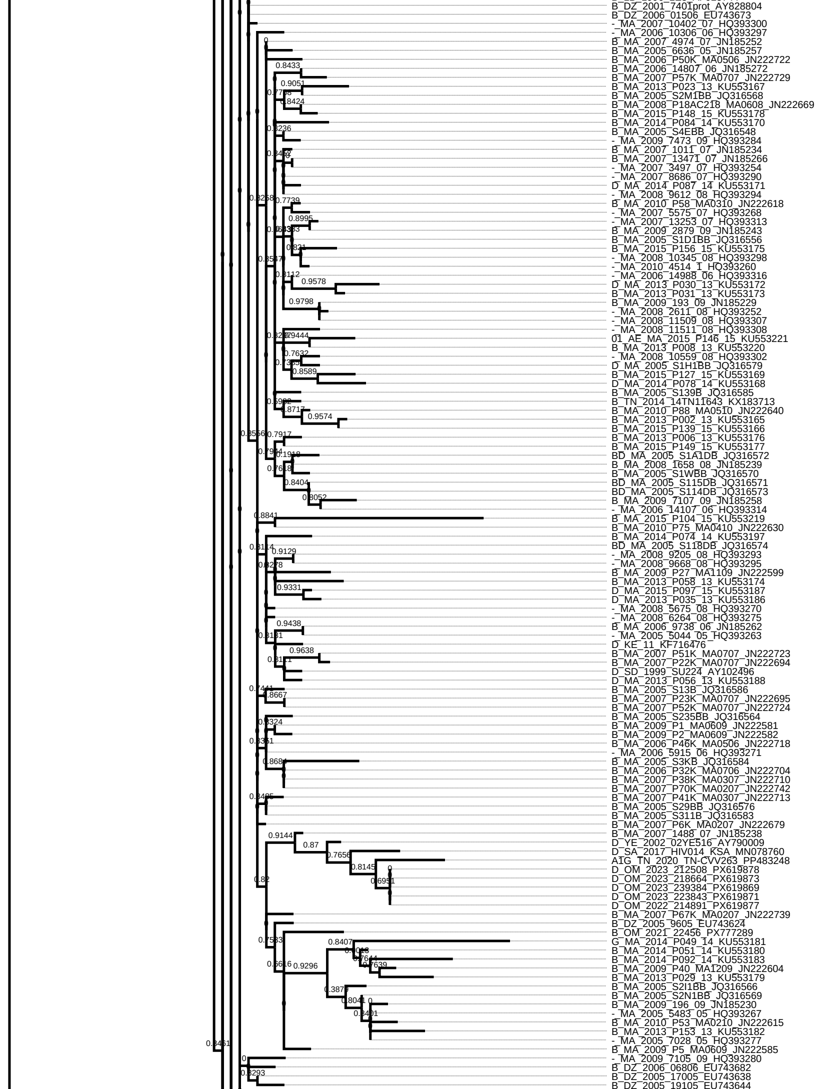
B_DZ_2001_7401prot_AY828804
B_DZ_2006_01506_EU743673
-_MA_2007_10402_07_HQ393300
-_MA_2006_10306_06_HQ393297
B_MA_2007_4974_07_JN185252
B_MA_2005_6636_05_JN185257
B_MA_2006_P50K_MA0506_JN222722
B_MA_2006_14807_06_JN185272
B_MA_2007_P57K_MA0707_JN222729
B_MA_2013_P023_13_KU553167
B_MA_2005_S2M1BB_JQ316568
B_MA_2008_P18AC218_MA0608_JN222669
B_MA_2015_P148_15_KU553178
B_MA_2014_P084_14_KU553170
B_MA_2005_S4EBB_JQ316548
-_MA_2009_7473_09_HQ393284
B_MA_2007_1011_07_JN185234
B_MA_2007_13471_07_JN185266
-_MA_2007_3497_07_HQ393254
-_MA_2007_8686_07_HQ393290
D_MA_2014_P087_14_KU553171
-_MA_2008_9612_08_HQ393294
B_MA_2010_P58_MA0310_JN222618
-_MA_2007_5575_07_HQ393268
-_MA_2007_13253_07_HQ393313
B_MA_2009_2879_09_JN185243
B_MA_2005_S1D1BB_JQ316556
B_MA_2015_P156_15_KU553175
-_MA_2008_10345_08_HQ393298
-_MA_2010_4514_1_HQ393260
-_MA_2006_14988_06_HQ393316
D_MA_2013_P030_13_KU553172
B_MA_2013_P031_13_KU553173
B_MA_2009_193_09_JN185229
-_MA_2008_2611_08_HQ393252
-_MA_2008_11509_08_HQ393307
-_MA_2008_11511_08_HQ393308
01_AE_MA_2015_P146_15_KU553221
B_MA_2013_P008_13_KU553220
-_MA_2008_10559_08_HQ393302
D_MA_2005_S1H1BB_JQ316579
B_MA_2015_P127_15_KU553169
D_MA_2014_P078_14_KU553168
B_MA_2005_S139B_JQ316585
B_TN_2014_14TN11643_KX183713
B_MA_2010_P88_MA0510_JN222640
B_MA_2013_P002_13_KU553165
B_MA_2015_P139_15_KU553166
B_MA_2013_P006_13_KU553176
B_MA_2015_P149_15_KU553177
BD_MA_2005_S1A1DB_JQ316572
B_MA_2008_1658_08_JN185239
B_MA_2005_S1WBB_JQ316570
BD_MA_2005_S115DB_JQ316571
BD_MA_2005_S114DB_JQ316573
B_MA_2009_7107_09_JN185258
-_MA_2006_14107_06_HQ393314
B_MA_2015_P104_15_KU553219
B_MA_2010_P75_MA0410_JN222630
B_MA_2014_P074_14_KU553197
BD_MA_2005_S118DB_JQ316574
-_MA_2008_9205_08_HQ393293
-_MA_2008_9668_08_HQ393295
B_MA_2009_P27_MA1109_JN222599
B_MA_2013_P058_13_KU553174
D_MA_2015_P097_15_KU553187
D_MA_2013_P035_13_KU553186
-_MA_2008_5675_08_HQ393270
-_MA_2008_6264_08_HQ393275
B_MA_2006_9738_06_JN185262
-_MA_2005_5044_05_HQ393263
D_KE_11_KF716476
B_MA_2007_P51K_MA0707_JN222723
B_MA_2007_P22K_MA0707_JN222694
D_SD_1999_SU224_AY102496
D_MA_2013_P056_13_KU553188
B_MA_2005_S13B_JQ316586
B_MA_2007_P23K_MA0707_JN222695
B_MA_2007_P52K_MA0707_JN222724
B_MA_2005_S235BB_JQ316564
B_MA_2009_P1_MA0609_JN222581
B_MA_2009_P2_MA0609_JN222582
B_MA_2006_P46K_MA0506_JN222718
-_MA_2006_5915_06_HQ393271
B_MA_2005_S3KB_JQ316584
B_MA_2006_P32K_MA0706_JN222704
B_MA_2007_P38K_MA0307_JN222710
B_MA_2007_P70K_MA0207_JN222742
B_MA_2007_P41K_MA0307_JN222713
B_MA_2005_S29BB_JQ316576
B_MA_2005_S311B_JQ316583
B_MA_2007_P6K_MA0207_JN222679
B_MA_2007_1488_07_JN185238
D_YE_2002_02YE516_AY790009
D_SA_2017_HIV014_KSA_MN078760
A1G_TN_2020_TN-CVV263_PP483248
D_OM_2023_212508_PX619878
D_OM_2023_218664_PX619873
D_OM_2023_239384_PX619869
D_OM_2023_223843_PX619871
D_OM_2022_214891_PX619877
B_MA_2007_P67K_MA0207_JN222739
B_DZ_2005_9605_EU743624
B_OM_2021_22456_PX777289
G_MA_2014_P049_14_KU553181
B_MA_2014_P051_14_KU553180
B_MA_2014_P092_14_KU553183
B_MA_2009_P40_MA1209_JN222604
B_MA_2013_P029_13_KU553179
B_MA_2005_S2I1BB_JQ316566
B_MA_2005_S2N1BB_JQ316569
B_MA_2009_196_09_JN185230
-_MA_2005_5483_05_HQ393267
B_MA_2010_P53_MA0210_JN222615
B_MA_2013_P153_13_KU553182
-_MA_2005_7028_05_HQ393277
B_MA_2009_P5_MA0609_JN222585
-_MA_2009_7105_09_HQ393280
B_DZ_2006_06806_EU743682
B_DZ_2005_17005_EU743638
B_DZ_2005_19105_EU743644

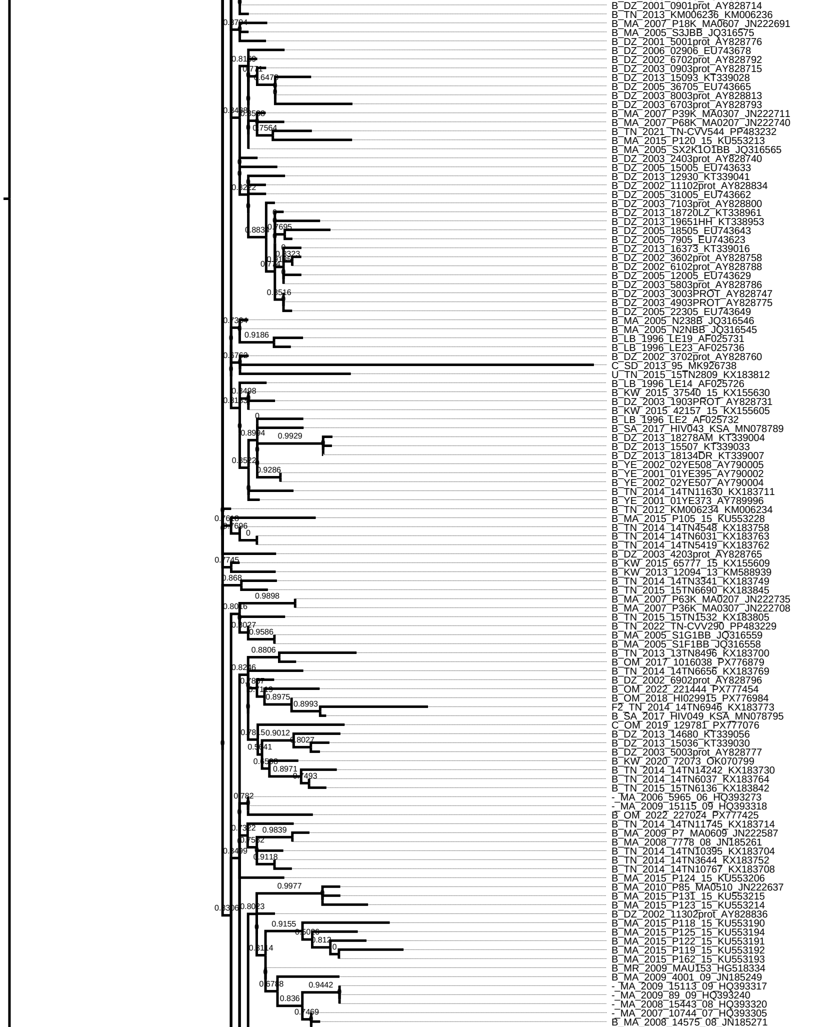
B_DZ_2001_0901prot_AY828714
B_TN_2013_KM006236_KM006236
B_MA_2007_P18K_MA0607_JN222691
B_MA_2005_S3JBB_JQ316575
B_DZ_2001_5001prot_AY828776
B_DZ_2006_02906_EU743678
B_DZ_2002_6702prot_AY828792
B_DZ_2003_0903prot_AY828715
B_DZ_2013_15093_KT339028
B_DZ_2005_36705_EU743665
B_DZ_2003_8003prot_AY828813
B_DZ_2003_6703prot_AY828793
B_MA_2007_P39K_MA0307_JN222711
B_MA_2007_P68K_MA0207_JN222740
B_TN_2021_TN-CVV544_PP483232
B_MA_2015_P120_15_KU553213
B_MA_2005_SX2K1O1BB_JQ316565
B_DZ_2003_2403prot_AY828740
B_DZ_2005_15005_EU743633
B_DZ_2013_12930_KT339041
B_DZ_2002_11102prot_AY828834
B_DZ_2005_31005_EU743662
B_DZ_2003_7103prot_AY828800
B_DZ_2013_18720LZ_KT338961
B_DZ_2013_19651HH_KT338953
B_DZ_2005_18505_EU743643
B_DZ_2005_7905_EU743623
B_DZ_2013_16373_KT339016
B_DZ_2002_3602prot_AY828758
B_DZ_2002_6102prot_AY828788
B_DZ_2005_12005_EU743629
B_DZ_2003_5803prot_AY828786
B_DZ_2003_3003PROT_AY828747
B_DZ_2003_4903PROT_AY828775
B_DZ_2005_22305_EU743649
B_MA_2005_N238B_JQ316546
B_MA_2005_N2NBB_JQ316545
B_LB_1996_LE19_AF025731
B_LB_1996_LE23_AF025736
B_DZ_2002_3702prot_AY828760
C_SD_2013_95_MK926738
U_TN_2015_15TN2809_KX183812
B_LB_1996_LE14_AF025726
B_KW_2015_37540_15_KX155630
B_DZ_2003_1903PROT_AY828731
B_KW_2015_42157_15_KX155605
B_LB_1996_LE2_AF025732
B_SA_2017_HIV043_KSA_MN078789
B_DZ_2013_18278AM_KT339004
B_DZ_2013_15507_KT339033
B_DZ_2013_18134DR_KT339007
B_YE_2002_02YE508_AY790005
B_YE_2001_01YE395_AY790002
B_YE_2002_02YE507_AY790004
B_TN_2014_14TN11630_KX183711
B_YE_2001_01YE373_AY789996
B_TN_2012_KM006234_KM006234
B_MA_2015_P105_15_KU553228
B_TN_2014_14TN4548_KX183758
B_TN_2014_14TN6031_KX183763
B_TN_2014_14TN5419_KX183762
B_DZ_2003_4203prot_AY828765
B_KW_2015_65777_15_KX155609
B_KW_2013_12094_13_KM588939
B_TN_2014_14TN3341_KX183749
B_TN_2015_15TN6690_KX183845
B_MA_2007_P63K_MA0207_JN222735
B_MA_2007_P36K_MA0307_JN222708
B_TN_2015_15TN1532_KX183805
B_TN_2022_TN-CVV290_PP483229
B_MA_2005_S1G1BB_JQ316559
B_MA_2005_S1F1BB_JQ316558
B_TN_2013_13TN8496_KX183700
B_OM_2017_1016038_PX776879
B_TN_2014_14TN6656_KX183769
B_DZ_2002_6902prot_AY828796
B_OM_2022_221444_PX777454
B_OM_2018_HI029915_PX776984
F2_TN_2014_14TN6946_KX183773
B_SA_2017_HIV049_KSA_MN078795
C_OM_2019_129781_PX777076
B_DZ_2013_14680_KT339056
B_DZ_2013_15036_KT339030
B_DZ_2003_5003prot_AY828777
B_KW_2020_72073_OK070799
B_TN_2014_14TN14242_KX183730
B_TN_2014_14TN6037_KX183764
B_TN_2015_15TN6136_KX183842
-_MA_2006_5965_06_HQ393273
-_MA_2009_15115_09_HQ393318
B_OM_2022_227024_PX777425
B_TN_2014_14TN11745_KX183714
B_MA_2009_P7_MA0609_JN222587
B_MA_2008_7778_08_JN185261
B_TN_2014_14TN10395_KX183704
B_TN_2014_14TN3644_KX183752
B_TN_2014_14TN10767_KX183708
B_MA_2015_P124_15_KU553206
B_MA_2010_P85_MA0510_JN222637
B_MA_2015_P131_15_KU553215
B_MA_2015_P123_15_KU553214
B_DZ_2002_11302prot_AY828836
B_MA_2015_P118_15_KU553190
B_MA_2015_P125_15_KU553194
B_MA_2015_P122_15_KU553191
B_MA_2015_P119_15_KU553192
B_MA_2015_P162_15_KU553193
B_MR_2009_MAU153_HG518334
B_MA_2009_4001_09_JN185249
-_MA_2009_15113_09_HQ393317
-_MA_2009_89_09_HQ393240
-_MA_2008_15443_08_HQ393320
-_MA_2007_10744_07_HQ393305
B_MA_2008_14575_08_JN185271

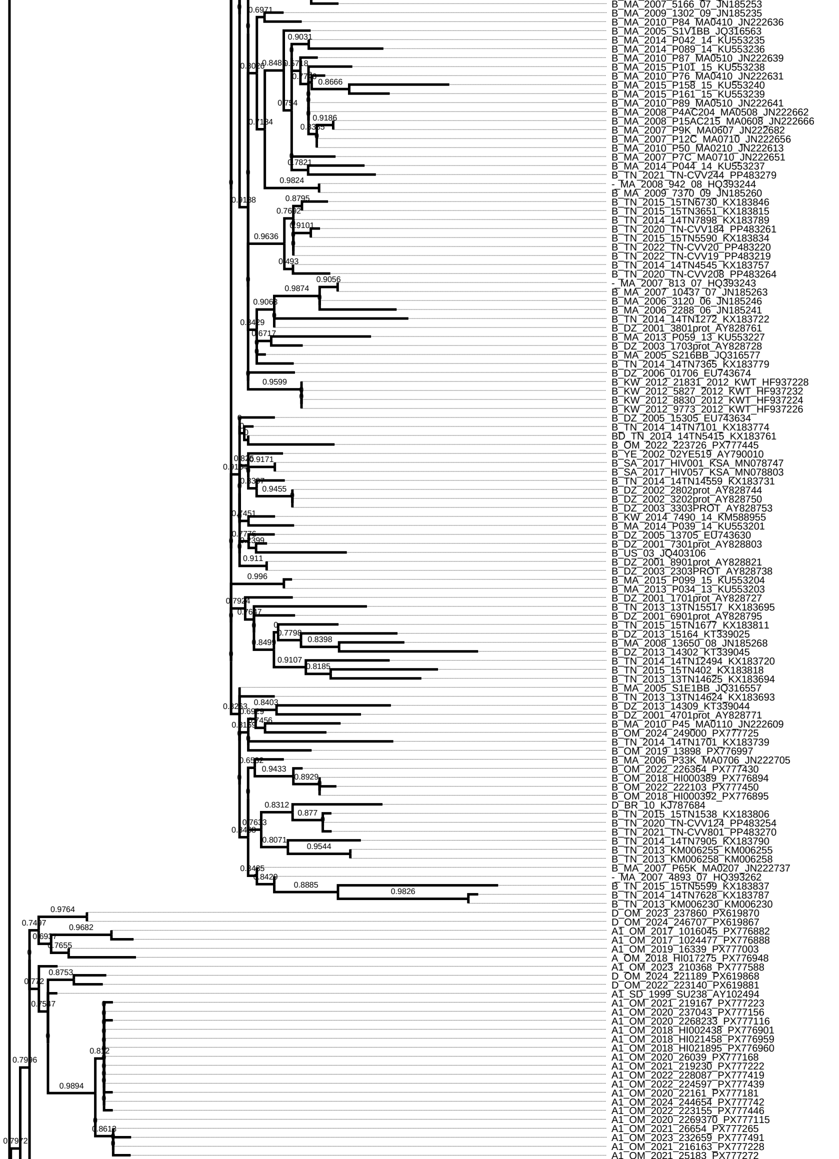
B_MA_2007_5166_07_JN185253
B_MA_2009_1302_09_JN185235
B_MA_2010_P84_MA0410_JN222636
B_MA_2005_S1V1BB_JQ316563
B_MA_2014_P042_14_KU553235
B_MA_2014_P089_14_KU553236
B_MA_2010_P87_MA0510_JN222639
B_MA_2015_P101_15_KU553238
B_MA_2010_P76_MA0410_JN222631
B_MA_2015_P158_15_KU553240
B_MA_2015_P161_15_KU553239
B_MA_2010_P89_MA0510_JN222641
B_MA_2008_P4AC204_MA0508_JN222662
B_MA_2008_P15AC215_MA0608_JN222666
B_MA_2007_P9K_MA0607_JN222682
B_MA_2007_P12C_MA0710_JN222656
B_MA_2010_P50_MA0210_JN222613
B_MA_2007_P7C_MA0710_JN222651
B_MA_2014_P044_14_KU553237
B_TN_2021_TN-CVV244_PP483279
-_MA_2008_942_08_HQ393244
B_MA_2009_7370_09_JN185260
B_TN_2015_15TN6730_KX183846
B_TN_2015_15TN3651_KX183815
B_TN_2014_14TN7898_KX183789
B_TN_2020_TN-CVV184_PP483261
B_TN_2015_15TN5590_KX183834
B_TN_2022_TN-CVV20_PP483220
B_TN_2022_TN-CVV19_PP483219
B_TN_2014_14TN4545_KX183757
B_TN_2020_TN-CVV208_PP483264
-_MA_2007_813_07_HQ393243
B_MA_2007_10437_07_JN185263
B_MA_2006_3120_06_JN185246
B_MA_2006_2288_06_JN185241
B_TN_2014_14TN1272_KX183722
B_DZ_2001_3801prot_AY828761
B_MA_2013_P059_13_KU553227
B_DZ_2003_1703prot_AY828728
B_MA_2005_S216BB_JQ316577
B_TN_2014_14TN7365_KX183779
B_DZ_2006_01706_EU743674
B_KW_2012_21831_2012_KWT_HF937228
B_KW_2012_5827_2012_KWT_HF937232
B_KW_2012_8830_2012_KWT_HF937224
B_KW_2012_9773_2012_KWT_HF937226
B_DZ_2005_15305_EU743634
B_TN_2014_14TN7101_KX183774
BD_TN_2014_14TN5415_KX183761
B_OM_2022_223726_PX777445
B_YE_2002_02YE519_AY790010
B_SA_2017_HIV001_KSA_MN078747
B_SA_2017_HIV057_KSA_MN078803
B_TN_2014_14TN14559_KX183731
B_DZ_2002_2802prot_AY828744
B_DZ_2002_3202prot_AY828750
B_DZ_2003_3303PROT_AY828753
B_KW_2014_7490_14_KM588955
B_MA_2014_P039_14_KU553201
B_DZ_2005_13705_EU743630
B_DZ_2001_7301prot_AY828803
B_US_03_JQ403106
B_DZ_2001_8901prot_AY828821
B_DZ_2003_2303PROT_AY828738
B_MA_2015_P099_15_KU553204
B_MA_2013_P034_13_KU553203
B_DZ_2001_1701prot_AY828727
B_TN_2013_13TN15517_KX183695
B_DZ_2001_6901prot_AY828795
B_TN_2015_15TN1677_KX183811
B_DZ_2013_15164_KT339025
B_MA_2008_13650_08_JN185268
B_DZ_2013_14302_KT339045
B_TN_2014_14TN12494_KX183720
B_TN_2015_15TN402_KX183818
B_TN_2013_13TN14625_KX183694
B_MA_2005_S1E1BB_JQ316557
B_TN_2013_13TN14624_KX183693
B_DZ_2013_14309_KT339044
B_DZ_2001_4701prot_AY828771
B_MA_2010_P45_MA0110_JN222609
B_OM_2024_249000_PX777725
B_TN_2014_14TN1701_KX183739
B_OM_2019_13898_PX776997
B_MA_2006_P33K_MA0706_JN222705
B_OM_2022_226364_PX777430
B_OM_2018_HI000389_PX776894
B_OM_2022_222103_PX777450
B_OM_2018_HI000392_PX776895
D_BR_10_KJ787684
B_TN_2015_15TN1538_KX183806
B_TN_2020_TN-CVV124_PP483254
B_TN_2021_TN-CVV801_PP483270
B_TN_2014_14TN7905_KX183790
B_TN_2013_KM006255_KM006255
B_TN_2013_KM006258_KM006258
B_MA_2007_P65K_MA0207_JN222737
-_MA_2007_4893_07_HQ393262
B_TN_2015_15TN5599_KX183837
B_TN_2014_14TN7628_KX183787
B_TN_2013_KM006230_KM006230
D_OM_2023_237860_PX619870
D_OM_2024_246707_PX619867
A1_OM_2017_1016045_PX776882
A1_OM_2017_1024477_PX776888
A1_OM_2019_16339_PX777003
A_OM_2018_HI017275_PX776948
A1_OM_2023_210368_PX777588
D_OM_2024_221189_PX619868
D_OM_2022_223140_PX619881
A1_SD_1999_SU238_AY102494
A1_OM_2021_219167_PX777223
A1_OM_2020_237043_PX777156
A1_OM_2020_2268233_PX777116
A1_OM_2018_HI002438_PX776901
A1_OM_2018_HI021458_PX776959
A1_OM_2018_HI021895_PX776960
A1_OM_2020_26039_PX777168
A1_OM_2021_219230_PX777222
A1_OM_2022_228087_PX777419
A1_OM_2022_224597_PX777439
A1_OM_2020_22161_PX777181
A1_OM_2024_244654_PX777742
A1_OM_2022_223155_PX777446
A1_OM_2020_2269370_PX777115
A1_OM_2021_26654_PX777265
A1_OM_2023_232659_PX777491
A1_OM_2021_216163_PX777228
A1_OM_2021_25183_PX777272

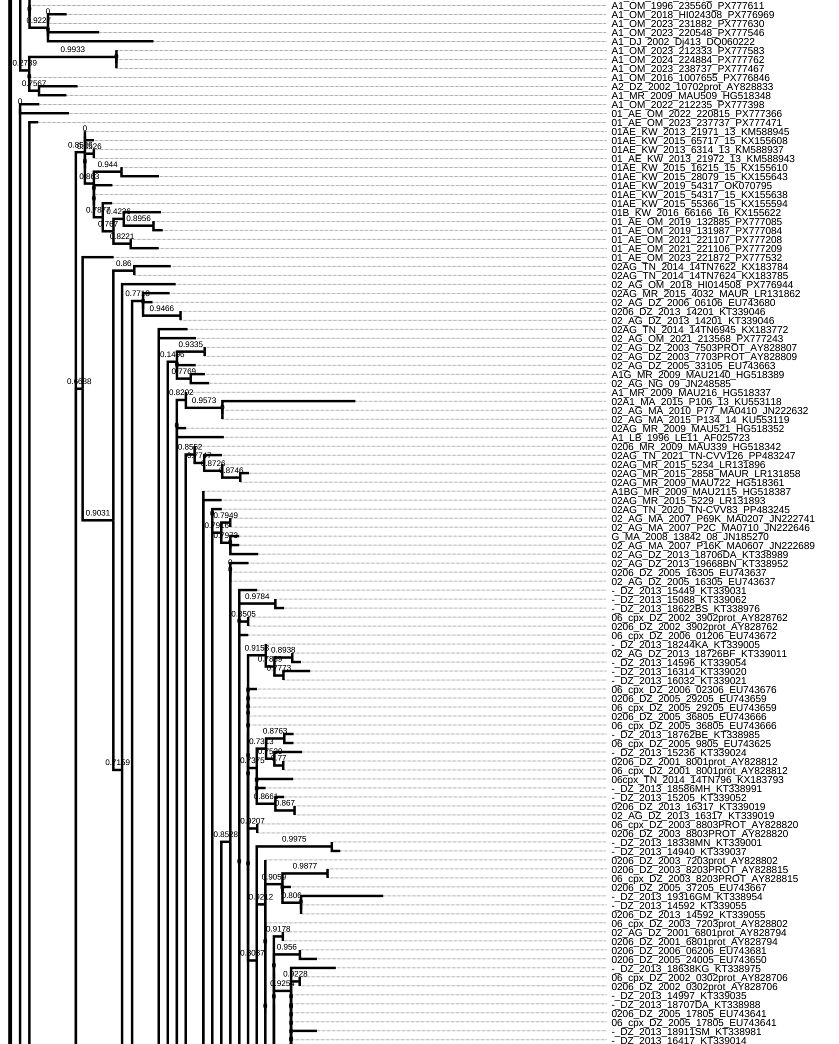
A1_OM_1996_235560_PX777611
A1_OM_2018_HI024308_PX776969
A1_OM_2023_231882_PX777630
A1_OM_2023_220548_PX777546
A1_DJ_2002_Dj413_DQ060222
A1_OM_2023_212333_PX777583
A1_OM_2024_224884_PX777762
A1_OM_2023_238737_PX777467
A1_OM_2016_1007655_PX776846
A2_DZ_2002_10702prot_AY828833
A1_MR_2009_MAU509_HG518348
A1_OM_2022_212235_PX777398
01_AE_OM_2022_220815_PX777366
01_AE_OM_2023_237737_PX777471
01AE_KW_2013_21971_13_KM588945
01AE_KW_2015_65717_15_KX155608
01AE_KW_2013_6314_13_KM588937
01_AE_KW_2013_21972_13_KM588943
01AE_KW_2015_16215_15_KX155610
01AE_KW_2015_28079_15_KX155643
01AE_KW_2019_54317_OK070795
01AE_KW_2015_54317_15_KX155638
01AE_KW_2015_55366_15_KX155594
01B_KW_2016_66166_16_KX155622
01_AE_OM_2019_132885_PX777085
01_AE_OM_2019_131987_PX777084
01_AE_OM_2021_221107_PX777208
01_AE_OM_2021_221106_PX777209
01_AE_OM_2023_221872_PX777532
02AG_TN_2014_14TN7622_KX183784
02AG_TN_2014_14TN7624_KX183785
02_AG_OM_2018_HI014508_PX776944
02AG_MR_2015_4032_MAUR_LR131862
02_AG_DZ_2006_06106_EU743680
0206_DZ_2013_14201_KT339046
02_AG_DZ_2013_14201_KT339046
02AG_TN_2014_14TN6945_KX183772
02_AG_OM_2021_213568_PX777243
02_AG_DZ_2003_7503PROT_AY828807
02_AG_DZ_2003_7703PROT_AY828809
02_AG_DZ_2005_33105_EU743663
A1G_MR_2009_MAU2140_HG518389
02_AG_NG_09_JN248585
A1_MR_2009_MAU216_HG518337
02A1_MA_2015_P106_13_KU553118
02_AG_MA_2010_P77_MA0410_JN222632
02_AG_MA_2015_P134_14_KU553119
02AG_MR_2009_MAU521_HG518352
A1_LB_1996_LE11_AF025723
0206_MR_2009_MAU339_HG518342
02AG_TN_2021_TN-CVV126_PP483247
02AG_MR_2015_5234_LR131896
02AG_MR_2015_2858_MAUR_LR131858
02AG_MR_2009_MAU722_HG518361
A1BG_MR_2009_MAU2115_HG518387
02AG_MR_2015_5229_LR131893
02AG_TN_2020_TN-CVV83_PP483245
02_AG_MA_2007_P69K_MA0207_JN222741
02_AG_MA_2007_P2C_MA0710_JN222646
G_MA_2008_13842_08_JN185270
02_AG_MA_2007_P16K_MA0607_JN222689
02_AG_DZ_2013_18706DA_KT338989
02_AG_DZ_2013_19668BN_KT338952
0206_DZ_2005_16305_EU743637
02_AG_DZ_2005_16305_EU743637
-_DZ_2013_15449_KT339031
-_DZ_2013_15088_KT339062
-_DZ_2013_18622BS_KT338976
06_cpx_DZ_2002_3902prot_AY828762
0206_DZ_2002_3902prot_AY828762
06_cpx_DZ_2006_01206_EU743672
-_DZ_2013_18244KA_KT339005
02_AG_DZ_2013_18726BF_KT339011
-_DZ_2013_14596_KT339054
-_DZ_2013_16314_KT339020
-_DZ_2013_16032_KT339021
06_cpx_DZ_2006_02306_EU743676
0206_DZ_2005_29205_EU743659
06_cpx_DZ_2005_29205_EU743659
0206_DZ_2005_36805_EU743666
06_cpx_DZ_2005_36805_EU743666
-_DZ_2013_18762BE_KT338985
06_cpx_DZ_2005_9805_EU743625
-_DZ_2013_15236_KT339024
0206_DZ_2001_8001prot_AY828812
06_cpx_DZ_2001_8001prot_AY828812
06cpx_TN_2014_14TN796_KX183793
-_DZ_2013_18586MH_KT338991
-_DZ_2013_15205_KT339052
0206_DZ_2013_16317_KT339019
02_AG_DZ_2013_16317_KT339019
06_cpx_DZ_2003_8803PROT_AY828820
0206_DZ_2003_8803PROT_AY828820
-_DZ_2013_18338MN_KT339001
-_DZ_2013_14940_KT339037
0206_DZ_2003_7203prot_AY828802
0206_DZ_2003_8203PROT_AY828815
06_cpx_DZ_2003_8203PROT_AY828815
0206_DZ_2005_37205_EU743667
-_DZ_2013_19316GM_KT338954
-_DZ_2013_14592_KT339055
0206_DZ_2013_14592_KT339055
06_cpx_DZ_2003_7203prot_AY828802
02_AG_DZ_2001_6801prot_AY828794
0206_DZ_2001_6801prot_AY828794
0206_DZ_2006_06206_EU743681
0206_DZ_2005_24005_EU743650
-_DZ_2013_18638KG_KT338975
06_cpx_DZ_2002_0302prot_AY828706
0206_DZ_2002_0302prot_AY828706
-_DZ_2013_14997_KT339035
-_DZ_2013_18707DA_KT338988
0206_DZ_2005_17805_EU743641
06_cpx_DZ_2005_17805_EU743641
-_DZ_2013_18911SM_KT338981
-_DZ_2013_16417_KT339014

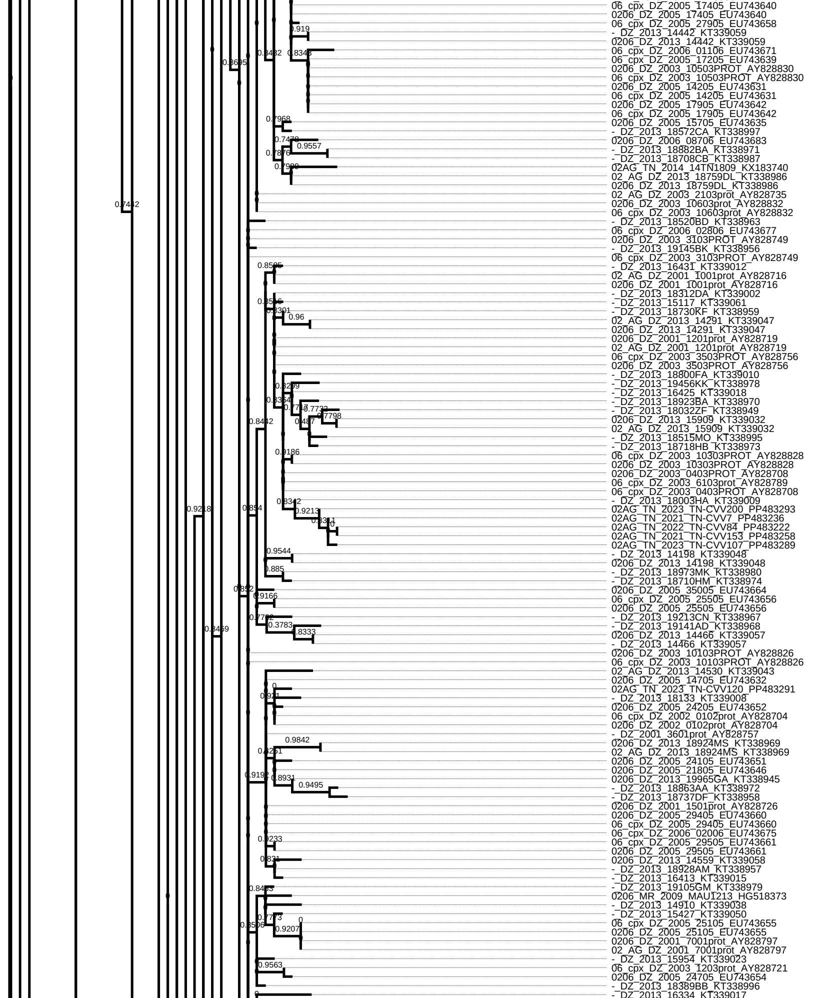
06_cpx_DZ_2005_17405_EU743640
0206_DZ_2005_17405_EU743640
06_cpx_DZ_2005_27905_EU743658
-_DZ_2013_14442_KT339059
0206_DZ_2013_14442_KT339059
06_cpx_DZ_2006_01106_EU743671
06_cpx_DZ_2005_17205_EU743639
0206_DZ_2003_10503PROT_AY828830
06_cpx_DZ_2003_10503PROT_AY828830
0206_DZ_2005_14205_EU743631
06_cpx_DZ_2005_14205_EU743631
0206_DZ_2005_17905_EU743642
06_cpx_DZ_2005_17905_EU743642
0206_DZ_2005_15705_EU743635
-_DZ_2013_18572CA_KT338997
0206_DZ_2006_08706_EU743683
-_DZ_2013_18882BA_KT338971
-_DZ_2013_18708CB_KT338987
02AG_TN_2014_14TN1809_KX183740
02_AG_DZ_2013_18759DL_KT338986
0206_DZ_2013_18759DL_KT338986
02_AG_DZ_2003_2103prot_AY828735
0206_DZ_2003_10603prot_AY828832
06_cpx_DZ_2003_10603prot_AY828832
-_DZ_2013_18520BD_KT338963
06_cpx_DZ_2006_02806_EU743677
0206_DZ_2003_3103PROT_AY828749
-_DZ_2013_19145BK_KT338956
06_cpx_DZ_2003_3103PROT_AY828749
-_DZ_2013_16431_KT339012
02_AG_DZ_2001_1001prot_AY828716
0206_DZ_2001_1001prot_AY828716
-_DZ_2013_18312DA_KT339002
-_DZ_2013_15117_KT339061
-_DZ_2013_18730KF_KT338959
02_AG_DZ_2013_14291_KT339047
0206_DZ_2013_14291_KT339047
0206_DZ_2001_1201prot_AY828719
02_AG_DZ_2001_1201prot_AY828719
06_cpx_DZ_2003_3503PROT_AY828756
0206_DZ_2003_3503PROT_AY828756
-_DZ_2013_18800FA_KT339010
-_DZ_2013_19456KK_KT338978
-_DZ_2013_16425_KT339018
-_DZ_2013_18923BA_KT338970
-_DZ_2013_18032ZF_KT338949
0206_DZ_2013_15909_KT339032
02_AG_DZ_2013_15909_KT339032
-_DZ_2013_18515MO_KT338995
-_DZ_2013_18718HB_KT338973
06_cpx_DZ_2003_10303PROT_AY828828
0206_DZ_2003_10303PROT_AY828828
0206_DZ_2003_0403PROT_AY828708
06_cpx_DZ_2003_6103prot_AY828789
06_cpx_DZ_2003_0403PROT_AY828708
-_DZ_2013_18003HA_KT339009
02AG_TN_2023_TN-CVV200_PP483293
02AG_TN_2021_TN-CVV7_PP483236
02AG_TN_2022_TN-CVV84_PP483222
02AG_TN_2021_TN-CVV153_PP483258
02AG_TN_2023_TN-CVV107_PP483289
-_DZ_2013_14198_KT339048
0206_DZ_2013_14198_KT339048
-_DZ_2013_18973MK_KT338980
-_DZ_2013_18710HM_KT338974
0206_DZ_2005_35005_EU743664
06_cpx_DZ_2005_25505_EU743656
0206_DZ_2005_25505_EU743656
-_DZ_2013_19213CN_KT338967
-_DZ_2013_19141AD_KT338968
0206_DZ_2013_14466_KT339057
-_DZ_2013_14466_KT339057
0206_DZ_2003_10103PROT_AY828826
06_cpx_DZ_2003_10103PROT_AY828826
02_AG_DZ_2013_14530_KT339043
0206_DZ_2005_14705_EU743632
02AG_TN_2023_TN-CVV120_PP483291
-_DZ_2013_18133_KT339008
0206_DZ_2005_24205_EU743652
06_cpx_DZ_2002_0102prot_AY828704
0206_DZ_2002_0102prot_AY828704
-_DZ_2001_3601prot_AY828757
0206_DZ_2013_18924MS_KT338969
02_AG_DZ_2013_18924MS_KT338969
0206_DZ_2005_24105_EU743651
0206_DZ_2005_21805_EU743646
0206_DZ_2013_19965GA_KT338945
-_DZ_2013_18863AA_KT338972
-_DZ_2013_18737DF_KT338958
0206_DZ_2001_1501prot_AY828726
0206_DZ_2005_29405_EU743660
06_cpx_DZ_2005_29405_EU743660
06_cpx_DZ_2006_02006_EU743675
06_cpx_DZ_2005_29505_EU743661
0206_DZ_2005_29505_EU743661
0206_DZ_2013_14559_KT339058
-_DZ_2013_18928AM_KT338957
-_DZ_2013_16413_KT339015
-_DZ_2013_19105GM_KT338979
0206_MR_2009_MAU1213_HG518373
-_DZ_2013_14910_KT339038
-_DZ_2013_15427_KT339050
06_cpx_DZ_2005_25105_EU743655
0206_DZ_2005_25105_EU743655
0206_DZ_2001_7001prot_AY828797
02_AG_DZ_2001_7001prot_AY828797
-_DZ_2013_15954_KT339023
06_cpx_DZ_2003_1203prot_AY828721
0206_DZ_2005_24705_EU743654
-_DZ_2013_18389BB_KT338996
-_DZ_2013_16334_KT339017

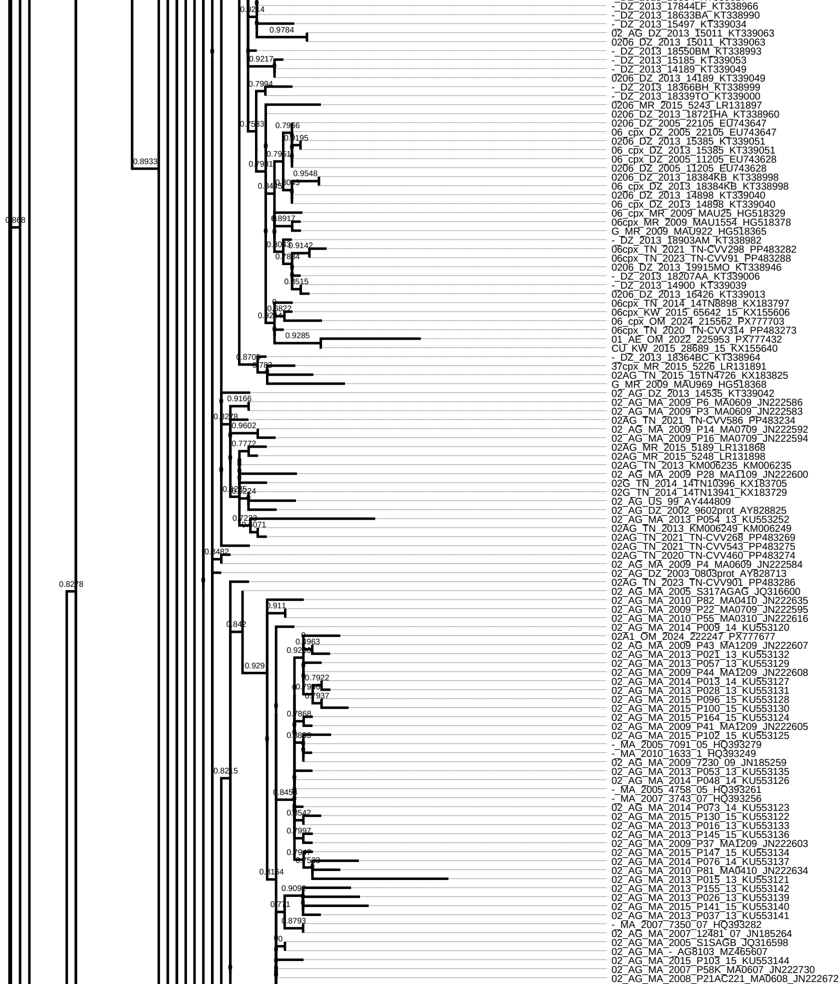

-_DZ_2013_17844LF_KT338966
-_DZ_2013_18633BA_KT338990
-_DZ_2013_15497_KT339034
02_AG_DZ_2013_15011_KT339063
0206_DZ_2013_15011_KT339063
-_DZ_2013_18550BM_KT338993
-_DZ_2013_15185_KT339053
-_DZ_2013_14189_KT339049
0206_DZ_2013_14189_KT339049
-_DZ_2013_18366BH_KT338999
-_DZ_2013_18339TO_KT339000
0206_MR_2015_5243_LR131897
0206_DZ_2013_18721HA_KT338960
0206_DZ_2005_22105_EU743647
06_cpx_DZ_2005_22105_EU743647
0206_DZ_2013_15385_KT339051
06_cpx_DZ_2013_15385_KT339051
06_cpx_DZ_2005_11205_EU743628
0206_DZ_2005_11205_EU743628
0206_DZ_2013_18384KB_KT338998
06_cpx_DZ_2013_18384KB_KT338998
0206_DZ_2013_14898_KT339040
06_cpx_DZ_2013_14898_KT339040
06_cpx_MR_2009_MAU25_HG518329
06cpx_MR_2009_MAU1554_HG518378
G_MR_2009_MAU922_HG518365
-_DZ_2013_18903AM_KT338982
06cpx_TN_2021_TN-CVV298_PP483282
06cpx_TN_2023_TN-CVV91_PP483288
0206_DZ_2013_19915MO_KT338946
-_DZ_2013_18207AA_KT339006
-_DZ_2013_14900_KT339039
0206_DZ_2013_16426_KT339013
06cpx_TN_2014_14TN8898_KX183797
06cpx_KW_2015_65642_15_KX155606
06_cpx_OM_2024_215562_PX777703
06cpx_TN_2020_TN-CVV314_PP483273
01_AE_OM_2022_225953_PX777432
CU_KW_2015_28689_15_KX155640
-_DZ_2013_18364BC_KT338964
37cpx_MR_2015_5226_LR131891
02AG_TN_2015_15TN4726_KX183825
G_MR_2009_MAU969_HG518368
02_AG_DZ_2013_14535_KT339042
02_AG_MA_2009_P6_MA0609_JN222586
02_AG_MA_2009_P3_MA0609_JN222583
02AG_TN_2021_TN-CVV586_PP483234
02_AG_MA_2009_P14_MA0709_JN222592
02_AG_MA_2009_P16_MA0709_JN222594
02AG_MR_2015_5189_LR131868
02AG_MR_2015_5248_LR131898
02AG_TN_2013_KM006235_KM006235
02_AG_MA_2009_P28_MA1109_JN222600
02G_TN_2014_14TN10396_KX183705
02G_TN_2014_14TN13941_KX183729
02_AG_US_99_AY444809
02_AG_DZ_2002_9602prot_AY828825
02_AG_MA_2013_P054_13_KU553252
02AG_TN_2013_KM006249_KM006249
02AG_TN_2021_TN-CVV268_PP483269
02AG_TN_2021_TN-CVV543_PP483275
02AG_TN_2020_TN-CVV460_PP483274
02_AG_MA_2009_P4_MA0609_JN222584
02_AG_DZ_2003_0803prot_AY828713
02AG_TN_2023_TN-CVV901_PP483286
02_AG_MA_2005_S317AGAG_JQ316600
02_AG_MA_2010_P82_MA0410_JN222635
02_AG_MA_2009_P22_MA0709_JN222595
02_AG_MA_2010_P55_MA0310_JN222616
02_AG_MA_2014_P009_14_KU553120
02A1_OM_2024_222247_PX777677
02_AG_MA_2009_P43_MA1209_JN222607
02_AG_MA_2013_P021_13_KU553132
02_AG_MA_2013_P057_13_KU553129
02_AG_MA_2009_P44_MA1209_JN222608
02_AG_MA_2014_P013_14_KU553127
02_AG_MA_2013_P028_13_KU553131
02_AG_MA_2015_P096_15_KU553128
02_AG_MA_2015_P100_15_KU553130
02_AG_MA_2015_P164_15_KU553124
02_AG_MA_2009_P41_MA1209_JN222605
02_AG_MA_2015_P102_15_KU553125
-_MA_2005_7091_05_HQ393279
-_MA_2010_1633_1_HQ393249
02_AG_MA_2009_7230_09_JN185259
02_AG_MA_2013_P053_13_KU553135
02_AG_MA_2014_P048_14_KU553126
-_MA_2005_4758_05_HQ393261
-_MA_2007_3743_07_HQ393256
02_AG_MA_2014_P073_14_KU553123
02_AG_MA_2015_P130_15_KU553122
02_AG_MA_2013_P016_13_KU553133
02_AG_MA_2013_P145_15_KU553136
02_AG_MA_2009_P37_MA1209_JN222603
02_AG_MA_2015_P147_15_KU553134
02_AG_MA_2014_P076_14_KU553137
02_AG_MA_2010_P81_MA0410_JN222634
02_AG_MA_2013_P015_13_KU553121
02_AG_MA_2013_P155_13_KU553142
02_AG_MA_2013_P026_13_KU553139
02_AG_MA_2015_P141_15_KU553140
02_AG_MA_2013_P037_13_KU553141
-_MA_2007_7350_07_HQ393282
02_AG_MA_2007_12481_07_JN185264
02_AG_MA_2005_S1SAGB_JQ316598
02_AG_MA_-_AG8103_MZ465607
02_AG_MA_2015_P103_15_KU553144
02_AG_MA_2007_P58K_MA0607_JN222730
02_AG_MA_2008_P21AC221_MA0608_JN222672
0.9784
0.9217
0.7994
0.8933
0.9548
0.8917
0.9142
0.9285
0.9166
0.9602
0.7772
0.842
0.929
0.911
0.7922
0.7937
0.7868
0.7997
0.9092
0.771
0.8793

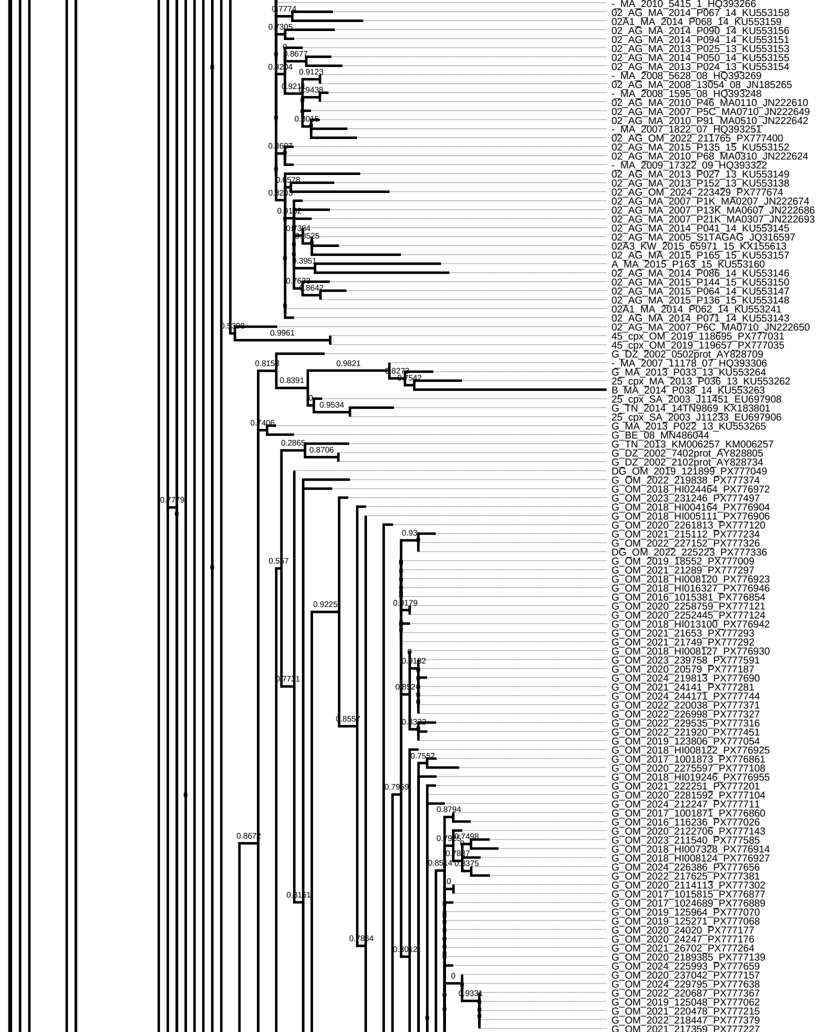
-_MA_2010_5415_1_HQ393266
02_AG_MA_2014_P067_14_KU553158
02A1_MA_2014_P068_14_KU553159
02_AG_MA_2014_P090_14_KU553156
02_AG_MA_2014_P094_14_KU553151
02_AG_MA_2013_P025_13_KU553153
02_AG_MA_2014_P050_14_KU553155
02_AG_MA_2013_P024_13_KU553154
-_MA_2008_5628_08_HQ393269
02_AG_MA_2008_13054_08_JN185265
-_MA_2008_1595_08_HQ393248
02_AG_MA_2010_P46_MA0110_JN222610
02_AG_MA_2007_P5C_MA0710_JN222649
02_AG_MA_2010_P91_MA0510_JN222642
-_MA_2007_1822_07_HQ393251
02_AG_OM_2022_211765_PX777400
02_AG_MA_2015_P135_15_KU553152
02_AG_MA_2010_P68_MA0310_JN222624
-_MA_2009_17322_09_HQ393322
02_AG_MA_2013_P027_13_KU553149
02_AG_MA_2013_P152_13_KU553138
02_AG_OM_2024_223429_PX777674
02_AG_MA_2007_P1K_MA0207_JN222674
02_AG_MA_2007_P13K_MA0607_JN222686
02_AG_MA_2007_P21K_MA0307_JN222693
02_AG_MA_2014_P041_14_KU553145
02_AG_MA_2005_S1TAGAG_JQ316597
02A3_KW_2015_65971_15_KX155613
02_AG_MA_2015_P165_15_KU553157
A_MA_2015_P163_15_KU553160
02_AG_MA_2014_P086_14_KU553146
02_AG_MA_2015_P144_15_KU553150
02_AG_MA_2015_P064_14_KU553147
02_AG_MA_2015_P136_15_KU553148
02A1_MA_2014_P062_14_KU553241
02_AG_MA_2014_P071_14_KU553143
02_AG_MA_2007_P6C_MA0710_JN222650
45_cpx_OM_2019_118695_PX777031
45_cpx_OM_2019_119657_PX777035
G_DZ_2002_0502prot_AY828709
-_MA_2007_11178_07_HQ393306
G_MA_2013_P033_13_KU553264
25_cpx_MA_2013_P036_13_KU553262
B_MA_2014_P038_14_KU553263
25_cpx_SA_2003_J11451_EU697908
G_TN_2014_14TN9869_KX183801
25_cpx_SA_2003_J11233_EU697906
G_MA_2013_P022_13_KU553265
G_BE_08_MN486044
G_TN_2013_KM006257_KM006257
G_DZ_2002_7402prot_AY828805
G_DZ_2002_2102prot_AY828734
DG_OM_2019_121899_PX777049
G_OM_2022_219838_PX777374
G_OM_2018_HI024464_PX776972
G_OM_2023_231246_PX777497
G_OM_2018_HI004164_PX776904
G_OM_2018_HI005111_PX776906
G_OM_2020_2261813_PX777120
G_OM_2021_215112_PX777234
G_OM_2022_227152_PX777326
DG_OM_2022_225223_PX777336
G_OM_2019_18552_PX777009
G_OM_2021_21289_PX777297
G_OM_2018_HI008120_PX776923
G_OM_2018_HI016327_PX776946
G_OM_2016_1015381_PX776854
G_OM_2020_2258759_PX777121
G_OM_2020_2252445_PX777124
G_OM_2018_HI013100_PX776942
G_OM_2021_21653_PX777293
G_OM_2021_21749_PX777292
G_OM_2018_HI008127_PX776930
G_OM_2023_239758_PX777591
G_OM_2020_20579_PX777187
G_OM_2024_219813_PX777690
G_OM_2021_24141_PX777281
G_OM_2024_244171_PX777744
G_OM_2022_220038_PX777371
G_OM_2022_226998_PX777327
G_OM_2022_229535_PX777316
G_OM_2022_221920_PX777451
G_OM_2019_123806_PX777054
G_OM_2018_HI008122_PX776925
G_OM_2017_1001873_PX776861
G_OM_2020_2275597_PX777108
G_OM_2018_HI019246_PX776955
G_OM_2021_222251_PX777201
G_OM_2020_2281592_PX777104
G_OM_2024_212247_PX777711
G_OM_2017_1001871_PX776860
G_OM_2016_116236_PX777026
G_OM_2020_2122706_PX777143
G_OM_2023_211540_PX777585
G_OM_2018_HI007328_PX776914
G_OM_2018_HI008124_PX776927
G_OM_2024_226386_PX777656
G_OM_2022_217625_PX777381
G_OM_2020_2114113_PX777302
G_OM_2017_1015815_PX776877
G_OM_2017_1024689_PX776889
G_OM_2019_125964_PX777070
G_OM_2019_125271_PX777068
G_OM_2020_24020_PX777177
G_OM_2020_24247_PX777176
G_OM_2021_26702_PX777264
G_OM_2020_2189385_PX777139
G_OM_2024_225993_PX777659
G_OM_2020_237042_PX777157
G_OM_2024_229795_PX777638
G_OM_2022_220687_PX777367
G_OM_2019_125048_PX777062
G_OM_2021_220478_PX777215
G_OM_2022_218447_PX777379
G_OM_2021_217359_PX777227

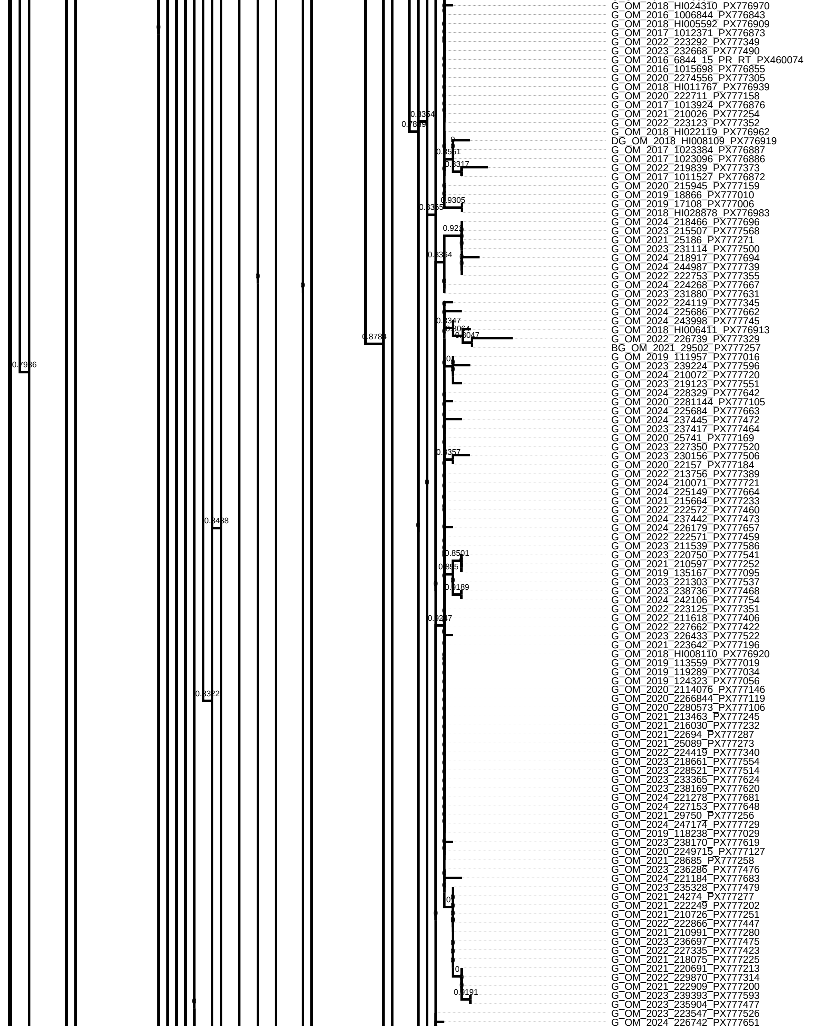
G_OM_2018_HI024310_PX776970
G_OM_2016_1006844_PX776843
G_OM_2018_HI005592_PX776909
G_OM_2017_1012371_PX776873
G_OM_2022_223292_PX777349
G_OM_2023_232668_PX777490
G_OM_2016_6844_15_PR_RT_PX460074
G_OM_2016_1015698_PX776855
G_OM_2020_2274556_PX777305
G_OM_2018_HI011767_PX776939
G_OM_2020_222711_PX777158
G_OM_2017_1013924_PX776876
G_OM_2021_210026_PX777254
G_OM_2022_223123_PX777352
G_OM_2018_HI022119_PX776962
DG_OM_2018_HI008109_PX776919
G_OM_2017_1023384_PX776887
G_OM_2017_1023096_PX776886
G_OM_2022_219839_PX777373
G_OM_2017_1011527_PX776872
G_OM_2020_215945_PX777159
G_OM_2019_18866_PX777010
G_OM_2019_17108_PX777006
G_OM_2018_HI028878_PX776983
G_OM_2024_218466_PX777696
G_OM_2023_215507_PX777568
G_OM_2021_25186_PX777271
G_OM_2023_231114_PX777500
G_OM_2024_218917_PX777694
G_OM_2024_244987_PX777739
G_OM_2022_222753_PX777355
G_OM_2024_224268_PX777667
G_OM_2023_231880_PX777631
G_OM_2022_224119_PX777345
G_OM_2024_225686_PX777662
G_OM_2024_243998_PX777745
G_OM_2018_HI006411_PX776913
G_OM_2022_226739_PX777329
BG_OM_2021_29502_PX777257
G_OM_2019_111957_PX777016
G_OM_2023_239224_PX777596
G_OM_2024_210072_PX777720
G_OM_2023_219123_PX777551
G_OM_2024_228329_PX777642
G_OM_2020_2281144_PX777105
G_OM_2024_225684_PX777663
G_OM_2024_237445_PX777472
G_OM_2023_237417_PX777464
G_OM_2020_25741_PX777169
G_OM_2023_227350_PX777520
G_OM_2023_230156_PX777506
G_OM_2020_22157_PX777184
G_OM_2022_213756_PX777389
G_OM_2024_210071_PX777721
G_OM_2024_225149_PX777664
G_OM_2021_215664_PX777233
G_OM_2022_222572_PX777460
G_OM_2024_237442_PX777473
G_OM_2024_226179_PX777657
G_OM_2022_222571_PX777459
G_OM_2023_211539_PX777586
G_OM_2023_220750_PX777541
G_OM_2021_210597_PX777252
G_OM_2019_135167_PX777095
G_OM_2023_221303_PX777537
G_OM_2023_238736_PX777468
G_OM_2024_242106_PX777754
G_OM_2022_223125_PX777351
G_OM_2022_211618_PX777406
G_OM_2022_227662_PX777422
G_OM_2023_226433_PX777522
G_OM_2021_223642_PX777196
G_OM_2018_HI008110_PX776920
G_OM_2019_113559_PX777019
G_OM_2019_119289_PX777034
G_OM_2019_124323_PX777056
G_OM_2020_2114076_PX777146
G_OM_2020_2266844_PX777119
G_OM_2020_2280573_PX777106
G_OM_2021_213463_PX777245
G_OM_2021_216030_PX777232
G_OM_2021_22694_PX777287
G_OM_2021_25089_PX777273
G_OM_2022_224419_PX777340
G_OM_2023_218661_PX777554
G_OM_2023_228521_PX777514
G_OM_2023_233365_PX777624
G_OM_2023_238169_PX777620
G_OM_2024_221278_PX777681
G_OM_2024_227153_PX777648
G_OM_2021_29750_PX777256
G_OM_2024_247174_PX777729
G_OM_2019_118238_PX777029
G_OM_2023_238170_PX777619
G_OM_2020_2249715_PX777127
G_OM_2021_28685_PX777258
G_OM_2023_236286_PX777476
G_OM_2024_221184_PX777683
G_OM_2023_235328_PX777479
G_OM_2021_24274_PX777277
G_OM_2021_222249_PX777202
G_OM_2021_210726_PX777251
G_OM_2022_222866_PX777447
G_OM_2021_210991_PX777280
G_OM_2023_236697_PX777475
G_OM_2022_227335_PX777423
G_OM_2021_218075_PX777225
G_OM_2021_220691_PX777213
G_OM_2022_229870_PX777314
G_OM_2021_222909_PX777200
G_OM_2023_239393_PX777593
G_OM_2023_235904_PX777477
G_OM_2023_223547_PX777526
G_OM_2024_226742_PX777651
0.7986
0.8784
0.3438
0.3322
0.8354
0.7889
0
0.3551
0.3317
0.9305
0.3365
0.927
0.3364
0.3347
0.8064
0.8047
0
0.3357
0.8501
0.855
0.9189
0.9247
0
0
0.9191

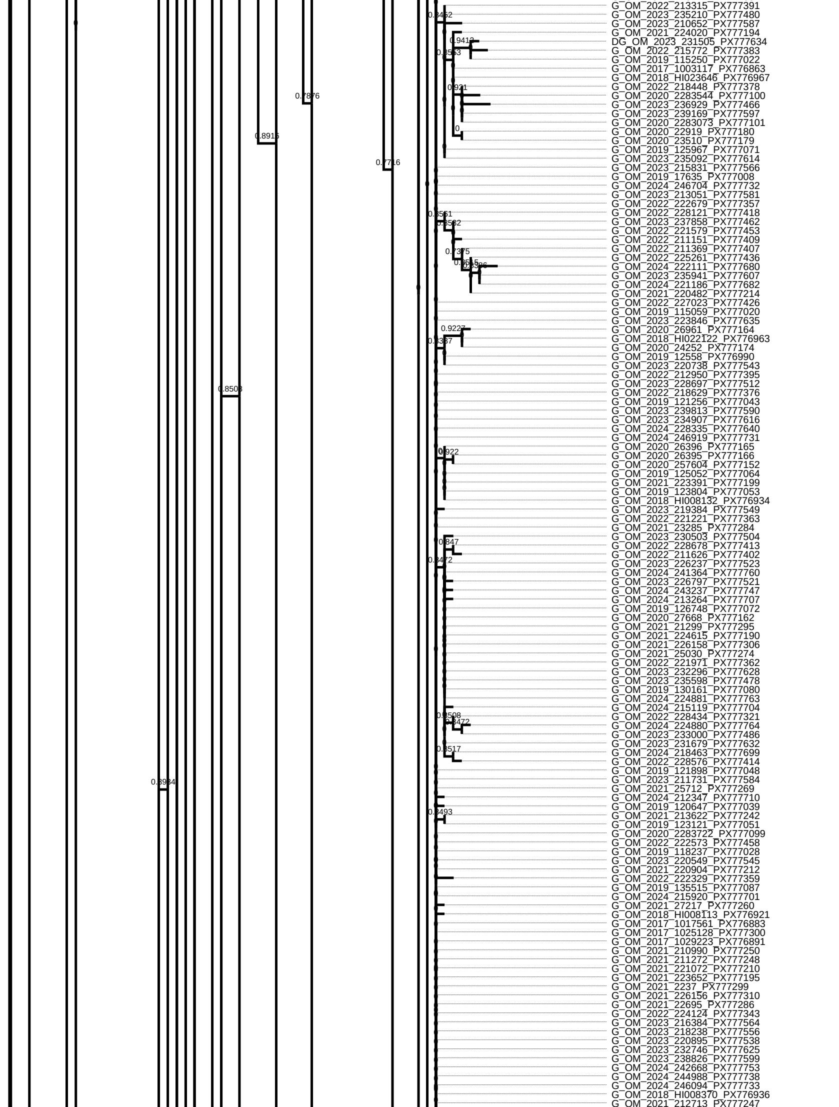
0.9412
0.7876
0.921
0
0.8916
0.7716
0.7375
0.9227
0.8503
0.922
0.847
0.8472
0.8508
0.8472
0.8517
0.8493
G_OM_2022_213315_PX777391
G_OM_2023_235210_PX777480
G_OM_2023_210652_PX777587
G_OM_2021_224020_PX777194
DG_OM_2023_231505_PX777634
G_OM_2022_215772_PX777383
G_OM_2019_115250_PX777022
G_OM_2017_1003117_PX776863
G_OM_2018_HI023646_PX776967
G_OM_2022_218448_PX777378
G_OM_2020_2283544_PX777100
G_OM_2023_236929_PX777466
G_OM_2023_239169_PX777597
G_OM_2020_2283073_PX777101
G_OM_2020_22919_PX777180
G_OM_2020_23510_PX777179
G_OM_2019_125967_PX777071
G_OM_2023_235092_PX777614
G_OM_2023_215831_PX777566
G_OM_2019_17635_PX777008
G_OM_2024_246704_PX777732
G_OM_2023_213051_PX777581
G_OM_2022_222679_PX777357
G_OM_2022_228121_PX777418
G_OM_2023_237858_PX777462
G_OM_2022_221579_PX777453
G_OM_2022_211151_PX777409
G_OM_2022_211369_PX777407
G_OM_2022_225261_PX777436
G_OM_2024_222111_PX777680
G_OM_2023_235941_PX777607
G_OM_2024_221186_PX777682
G_OM_2021_220482_PX777214
G_OM_2022_227023_PX777426
G_OM_2019_115059_PX777020
G_OM_2023_223846_PX777635
G_OM_2020_26961_PX777164
G_OM_2018_HI022122_PX776963
G_OM_2020_24252_PX777174
G_OM_2019_12558_PX776990
G_OM_2023_220738_PX777543
G_OM_2022_212950_PX777395
G_OM_2023_228697_PX777512
G_OM_2022_218629_PX777376
G_OM_2019_121256_PX777043
G_OM_2023_239813_PX777590
G_OM_2023_234907_PX777616
G_OM_2024_228335_PX777640
G_OM_2024_246919_PX777731
G_OM_2020_26396_PX777165
G_OM_2020_26395_PX777166
G_OM_2020_257604_PX777152
G_OM_2019_125052_PX777064
G_OM_2021_223391_PX777199
G_OM_2019_123804_PX777053
G_OM_2018_HI008132_PX776934
G_OM_2023_219384_PX777549
G_OM_2022_221221_PX777363
G_OM_2021_23285_PX777284
G_OM_2023_230503_PX777504
G_OM_2022_228678_PX777413
G_OM_2022_211626_PX777402
G_OM_2023_226237_PX777523
G_OM_2024_241364_PX777760
G_OM_2023_226797_PX777521
G_OM_2024_243237_PX777747
G_OM_2024_213264_PX777707
G_OM_2019_126748_PX777072
G_OM_2020_27668_PX777162
G_OM_2021_21299_PX777295
G_OM_2021_224615_PX777190
G_OM_2021_226158_PX777306
G_OM_2021_25030_PX777274
G_OM_2022_221971_PX777362
G_OM_2023_232296_PX777628
G_OM_2023_235598_PX777478
G_OM_2019_130161_PX777080
G_OM_2024_224881_PX777763
G_OM_2024_215119_PX777704
G_OM_2022_228434_PX777321
G_OM_2024_224880_PX777764
G_OM_2023_233000_PX777486
G_OM_2023_231679_PX777632
G_OM_2024_218463_PX777699
G_OM_2022_228576_PX777414
G_OM_2019_121898_PX777048
G_OM_2023_211731_PX777584
G_OM_2021_25712_PX777269
G_OM_2024_212347_PX777710
G_OM_2019_120647_PX777039
G_OM_2021_213622_PX777242
G_OM_2019_123121_PX777051
G_OM_2020_2283722_PX777099
G_OM_2022_222573_PX777458
G_OM_2019_118237_PX777028
G_OM_2023_220549_PX777545
G_OM_2021_220904_PX777212
G_OM_2022_222329_PX777359
G_OM_2019_135515_PX777087
G_OM_2024_215920_PX777701
G_OM_2021_27217_PX777260
G_OM_2018_HI008113_PX776921
G_OM_2017_1017561_PX776883
G_OM_2017_1025128_PX777300
G_OM_2017_1029223_PX776891
G_OM_2021_210990_PX777250
G_OM_2021_211272_PX777248
G_OM_2021_221072_PX777210
G_OM_2021_223652_PX777195
G_OM_2021_2237_PX777299
G_OM_2021_226156_PX777310
G_OM_2021_22695_PX777286
G_OM_2022_224124_PX777343
G_OM_2023_216384_PX777564
G_OM_2023_218238_PX777556
G_OM_2023_220895_PX777538
G_OM_2023_232746_PX777625
G_OM_2023_238826_PX777599
G_OM_2024_242668_PX777753
G_OM_2024_244988_PX777738
G_OM_2024_246094_PX777733
G_OM_2018_HI008370_PX776936
G_OM_2021_212713_PX777247

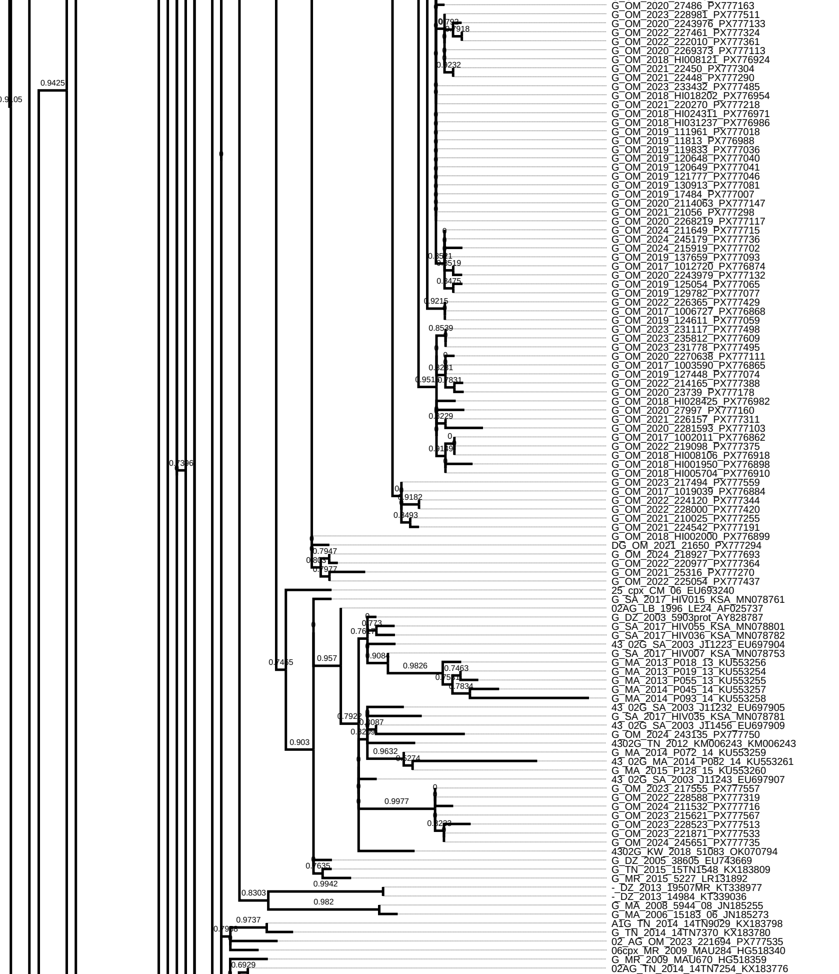

G_OM_2020_27486_PX777163
G_OM_2023_228981_PX777511
G_OM_2020_2243976_PX777133
G_OM_2022_227461_PX777324
G_OM_2022_222010_PX777361
G_OM_2020_2269373_PX777113
G_OM_2018_HI008121_PX776924
G_OM_2021_22450_PX777304
G_OM_2021_22448_PX777290
G_OM_2023_233432_PX777485
G_OM_2018_HI018202_PX776954
G_OM_2021_220270_PX777218
G_OM_2018_HI024311_PX776971
G_OM_2018_HI031237_PX776986
G_OM_2019_111961_PX777018
G_OM_2019_11813_PX776988
G_OM_2019_119833_PX777036
G_OM_2019_120648_PX777040
G_OM_2019_120649_PX777041
G_OM_2019_121777_PX777046
G_OM_2019_130913_PX777081
G_OM_2019_17484_PX777007
G_OM_2020_2114063_PX777147
G_OM_2021_21056_PX777298
G_OM_2020_2268219_PX777117
G_OM_2024_211649_PX777715
G_OM_2024_245179_PX777736
G_OM_2024_215919_PX777702
G_OM_2019_137659_PX777093
G_OM_2017_1012720_PX776874
G_OM_2020_2243979_PX777132
G_OM_2019_125054_PX777065
G_OM_2019_129782_PX777077
G_OM_2022_226365_PX777429
G_OM_2017_1006727_PX776868
G_OM_2019_124611_PX777059
G_OM_2023_231117_PX777498
G_OM_2023_235812_PX777609
G_OM_2023_231778_PX777495
G_OM_2020_2270638_PX777111
G_OM_2017_1003590_PX776865
G_OM_2019_127448_PX777074
G_OM_2022_214165_PX777388
G_OM_2020_23739_PX777178
G_OM_2018_HI028425_PX776982
G_OM_2020_27997_PX777160
G_OM_2021_226157_PX777311
G_OM_2020_2281593_PX777103
G_OM_2017_1002011_PX776862
G_OM_2022_219098_PX777375
G_OM_2018_HI008106_PX776918
G_OM_2018_HI001950_PX776898
G_OM_2018_HI005704_PX776910
G_OM_2023_217494_PX777559
G_OM_2017_1019039_PX776884
G_OM_2022_224120_PX777344
G_OM_2022_228000_PX777420
G_OM_2021_210025_PX777255
G_OM_2021_224542_PX777191
G_OM_2018_HI002000_PX776899
DG_OM_2021_21650_PX777294
G_OM_2024_218927_PX777693
G_OM_2022_220977_PX777364
G_OM_2021_25316_PX777270
G_OM_2022_225054_PX777437
25_cpx_CM_06_EU693240
G_SA_2017_HIV015_KSA_MN078761
02AG_LB_1996_LE24_AF025737
G_DZ_2003_5903prot_AY828787
G_SA_2017_HIV055_KSA_MN078801
G_SA_2017_HIV036_KSA_MN078782
43_02G_SA_2003_J11223_EU697904
G_SA_2017_HIV007_KSA_MN078753
G_MA_2013_P018_13_KU553256
G_MA_2013_P019_13_KU553254
G_MA_2013_P055_13_KU553255
G_MA_2014_P045_14_KU553257
G_MA_2014_P093_14_KU553258
43_02G_SA_2003_J11232_EU697905
G_SA_2017_HIV035_KSA_MN078781
43_02G_SA_2003_J11456_EU697909
G_OM_2024_243135_PX777750
4302G_TN_2012_KM006243_KM006243
G_MA_2014_P072_14_KU553259
43_02G_MA_2014_P082_14_KU553261
G_MA_2015_P128_15_KU553260
43_02G_SA_2003_J11243_EU697907
G_OM_2023_217555_PX777557
G_OM_2022_228588_PX777319
G_OM_2024_211532_PX777716
G_OM_2023_215621_PX777567
G_OM_2023_228523_PX777513
G_OM_2023_221871_PX777533
G_OM_2024_245651_PX777735
4302G_KW_2018_51083_OK070794
G_DZ_2005_38605_EU743669
G_TN_2015_15TN1548_KX183809
G_MR_2015_5227_LR131892
-_DZ_2013_19507MR_KT338977
-_DZ_2013_14984_KT339036
G_MA_2008_5944_08_JN185255
G_MA_2006_15183_06_JN185273
AIG_TN_2014_14TN9029_KX183798
G_TN_2014_14TN7370_KX183780
02_AG_OM_2023_221694_PX777535
06cpx_MR_2009_MAU284_HG518340
G_MR_2009_MAU670_HG518359
02AG_TN_2014_14TN7254_KX183776
0.9425
0.7396
0.9232
0.9215
0.8539
0.8229
0.9182
0.8493
0.7947
0.7977
0.7465
0.957
0.9826
0.7463
0.7834
0.7922
0.903
0.9632
0.9977
0.7635
0.9942
0.8303
0.982
0.9737
0.6929

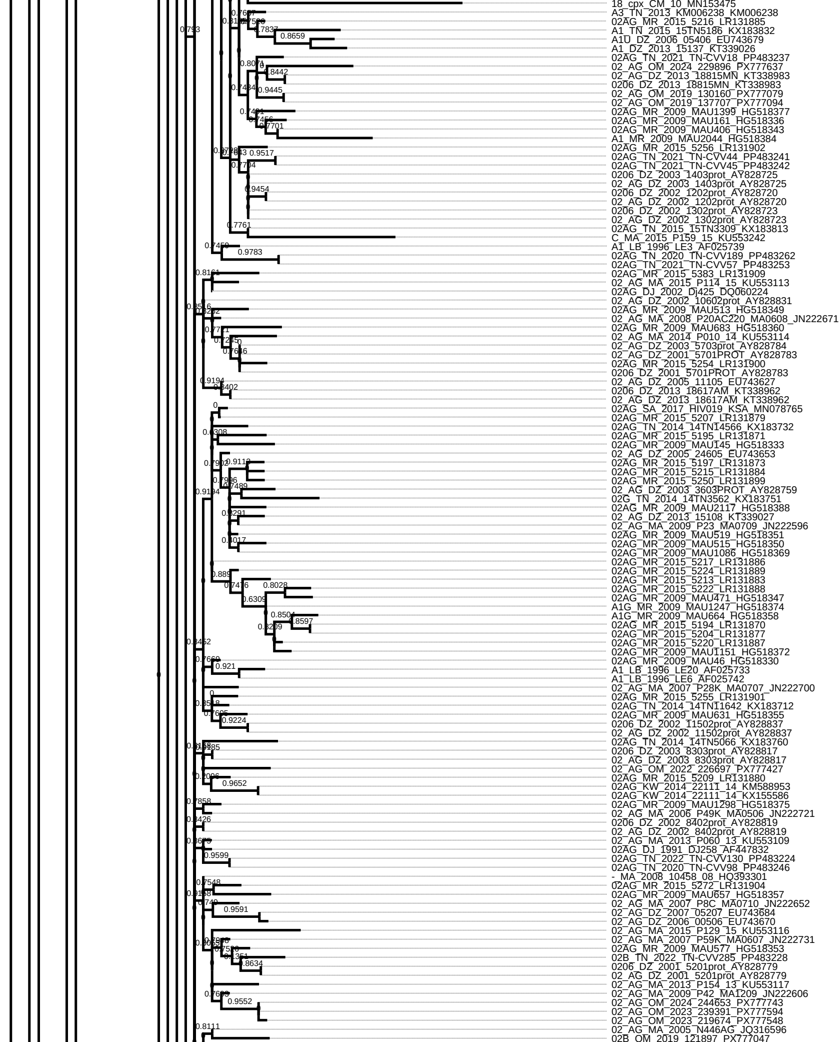
18_cpx_CM_10_MN153475
A3_TN_2013_KM006238_KM006238
02AG_MR_2015_5216_LR131885
A1_TN_2015_15TN5186_KX183832
A1U_DZ_2006_05406_EU743679
A1_DZ_2013_15137_KT339026
02AG_TN_2021_TN-CVV18_PP483237
02_AG_OM_2024_229896_PX777637
02_AG_DZ_2013_18815MN_KT338983
0206_DZ_2013_18815MN_KT338983
02_AG_OM_2019_130160_PX777079
02_AG_OM_2019_137707_PX777094
02AG_MR_2009_MAU1399_HG518377
02AG_MR_2009_MAU161_HG518336
02AG_MR_2009_MAU406_HG518343
A1_MR_2009_MAU2044_HG518384
02AG_MR_2015_5256_LR131902
02AG_TN_2021_TN-CVV44_PP483241
02AG_TN_2021_TN-CVV45_PP483242
0206_DZ_2003_1403prot_AY828725
02_AG_DZ_2003_1403prot_AY828725
0206_DZ_2002_1202prot_AY828720
02_AG_DZ_2002_1202prot_AY828720
0206_DZ_2002_1302prot_AY828723
02_AG_DZ_2002_1302prot_AY828723
02AG_TN_2015_15TN3309_KX183813
C_MA_2015_P159_15_KU553242
A1_LB_1996_LE3_AF025739
02AG_TN_2020_TN-CVV189_PP483262
02AG_TN_2021_TN-CVV57_PP483253
02AG_MR_2015_5383_LR131909
02_AG_MA_2015_P114_15_KU553113
02AG_DJ_2002_Dj425_DQ060224
02_AG_DZ_2002_10602prot_AY828831
02AG_MR_2009_MAU513_HG518349
02_AG_MA_2008_P20AC220_MA0608_JN222671
02AG_MR_2009_MAU683_HG518360
02_AG_MA_2014_P010_14_KU553114
02_AG_DZ_2003_5703prot_AY828784
02_AG_DZ_2001_5701PROT_AY828783
02AG_MR_2015_5254_LR131900
0206_DZ_2001_5701PROT_AY828783
02_AG_DZ_2005_11105_EU743627
0206_DZ_2013_18617AM_KT338962
02_AG_DZ_2013_18617AM_KT338962
02AG_SA_2017_HIV019_KSA_MN078765
02AG_MR_2015_5207_LR131879
02AG_TN_2014_14TN14566_KX183732
02AG_MR_2015_5195_LR131871
02AG_MR_2009_MAU145_HG518333
02_AG_DZ_2005_24605_EU743653
02AG_MR_2015_5197_LR131873
02AG_MR_2015_5215_LR131884
02AG_MR_2015_5250_LR131899
02_AG_DZ_2003_3603PROT_AY828759
02G_TN_2014_14TN3562_KX183751
02AG_MR_2009_MAU2117_HG518388
02_AG_DZ_2013_15108_KT339027
02_AG_MA_2009_P23_MA0709_JN222596
02AG_MR_2009_MAU519_HG518351
02AG_MR_2009_MAU515_HG518350
02AG_MR_2009_MAU1086_HG518369
02AG_MR_2015_5217_LR131886
02AG_MR_2015_5224_LR131889
02AG_MR_2015_5213_LR131883
02AG_MR_2015_5222_LR131888
02AG_MR_2009_MAU471_HG518347
A1G_MR_2009_MAU1247_HG518374
A1G_MR_2009_MAU664_HG518358
02AG_MR_2015_5194_LR131870
02AG_MR_2015_5204_LR131877
02AG_MR_2015_5220_LR131887
02AG_MR_2009_MAU1151_HG518372
02AG_MR_2009_MAU46_HG518330
A1_LB_1996_LE20_AF025733
A1_LB_1996_LE6_AF025742
02_AG_MA_2007_P28K_MA0707_JN222700
02AG_MR_2015_5255_LR131901
02AG_TN_2014_14TN11642_KX183712
02AG_MR_2009_MAU631_HG518355
0206_DZ_2002_11502prot_AY828837
02_AG_DZ_2002_11502prot_AY828837
02AG_TN_2014_14TN5066_KX183760
0206_DZ_2003_8303prot_AY828817
02_AG_DZ_2003_8303prot_AY828817
02_AG_OM_2022_226697_PX777427
02AG_MR_2015_5209_LR131880
02AG_KW_2014_22111_14_KM588953
02AG_KW_2014_22111_14_KX155586
02AG_MR_2009_MAU1298_HG518375
02_AG_MA_2006_P49K_MA0506_JN222721
0206_DZ_2002_8402prot_AY828819
02_AG_DZ_2002_8402prot_AY828819
02_AG_MA_2013_P060_13_KU553109
02AG_DJ_1991_DJ258_AF447832
02AG_TN_2022_TN-CVV130_PP483224
02AG_TN_2020_TN-CVV98_PP483246
-_MA_2008_10458_08_HQ393301
02AG_MR_2015_5272_LR131904
02AG_MR_2009_MAU657_HG518357
02_AG_MA_2007_P8C_MA0710_JN222652
02_AG_DZ_2007_05207_EU743684
02_AG_DZ_2006_00506_EU743670
02_AG_MA_2015_P129_15_KU553116
02_AG_MA_2007_P59K_MA0607_JN222731
02AG_MR_2009_MAU577_HG518353
02B_TN_2022_TN-CVV285_PP483228
0206_DZ_2001_5201prot_AY828779
02_AG_DZ_2001_5201prot_AY828779
02_AG_MA_2013_P154_13_KU553117
02_AG_MA_2009_P42_MA1209_JN222606
02_AG_OM_2024_244653_PX777743
02_AG_OM_2023_239391_PX777594
02_AG_OM_2023_219674_PX777548
02_AG_MA_2005_N446AG_JQ316596
02B_OM_2019_121897_PX777047

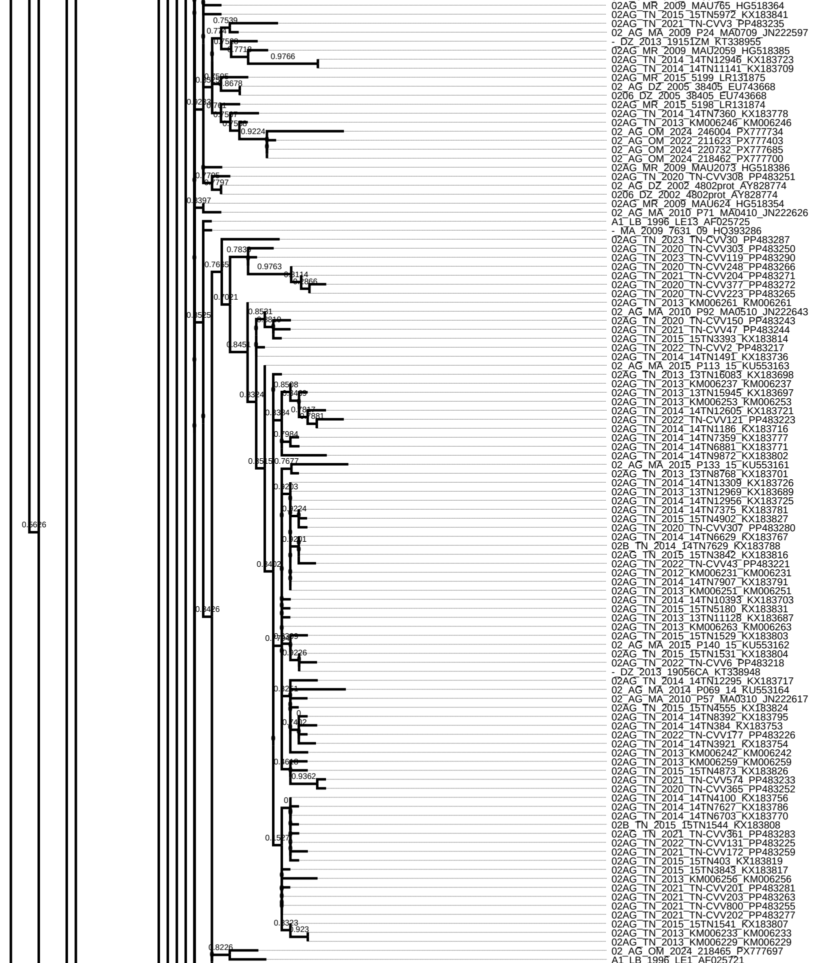
02AG_MR_2009_MAU765_HG518364
02AG_TN_2015_15TN5972_KX183841
02AG_TN_2021_TN-CVV3_PP483235
02_AG_MA_2009_P24_MA0709_JN222597
-_DZ_2013_19151ZM_KT338955
02AG_MR_2009_MAU2059_HG518385
02AG_TN_2014_14TN12946_KX183723
02AG_TN_2014_14TN11141_KX183709
02AG_MR_2015_5199_LR131875
02_AG_DZ_2005_38405_EU743668
0206_DZ_2005_38405_EU743668
02AG_MR_2015_5198_LR131874
02AG_TN_2014_14TN7360_KX183778
02AG_TN_2013_KM006246_KM006246
02_AG_OM_2024_246004_PX777734
02_AG_OM_2022_211623_PX777403
02_AG_OM_2024_220732_PX777685
02_AG_OM_2024_218462_PX777700
02AG_MR_2009_MAU2073_HG518386
02AG_TN_2020_TN-CVV308_PP483251
02_AG_DZ_2002_4802prot_AY828774
0206_DZ_2002_4802prot_AY828774
02AG_MR_2009_MAU624_HG518354
02_AG_MA_2010_P71_MA0410_JN222626
A1_LB_1996_LE13_AF025725
-_MA_2009_7631_09_HQ393286
02AG_TN_2023_TN-CVV30_PP483287
02AG_TN_2020_TN-CVV303_PP483250
02AG_TN_2023_TN-CVV119_PP483290
02AG_TN_2020_TN-CVV248_PP483266
02AG_TN_2021_TN-CVV204_PP483271
02AG_TN_2020_TN-CVV377_PP483272
02AG_TN_2020_TN-CVV223_PP483265
02AG_TN_2013_KM006261_KM006261
02_AG_MA_2010_P92_MA0510_JN222643
02AG_TN_2020_TN-CVV150_PP483243
02AG_TN_2021_TN-CVV47_PP483244
02AG_TN_2015_15TN3393_KX183814
02AG_TN_2022_TN-CVV2_PP483217
02AG_TN_2014_14TN1491_KX183736
02_AG_MA_2015_P113_15_KU553163
02AG_TN_2013_13TN16083_KX183698
02AG_TN_2013_KM006237_KM006237
02AG_TN_2013_13TN15945_KX183697
02AG_TN_2013_KM006253_KM006253
02AG_TN_2014_14TN12605_KX183721
02AG_TN_2022_TN-CVV121_PP483223
02AG_TN_2014_14TN1186_KX183716
02AG_TN_2014_14TN7359_KX183777
02AG_TN_2014_14TN6881_KX183771
02AG_TN_2014_14TN9872_KX183802
02_AG_MA_2015_P133_15_KU553161
02AG_TN_2013_13TN8768_KX183701
02AG_TN_2014_14TN13309_KX183726
02AG_TN_2013_13TN12969_KX183689
02AG_TN_2014_14TN12956_KX183725
02AG_TN_2014_14TN7375_KX183781
02AG_TN_2015_15TN4902_KX183827
02AG_TN_2020_TN-CVV307_PP483280
02AG_TN_2014_14TN6629_KX183767
02B_TN_2014_14TN7629_KX183788
02AG_TN_2015_15TN3842_KX183816
02AG_TN_2022_TN-CVV43_PP483221
02AG_TN_2012_KM006231_KM006231
02AG_TN_2014_14TN7907_KX183791
02AG_TN_2013_KM006251_KM006251
02AG_TN_2014_14TN10393_KX183703
02AG_TN_2015_15TN5180_KX183831
02AG_TN_2013_13TN11128_KX183687
02AG_TN_2013_KM006263_KM006263
02AG_TN_2015_15TN1529_KX183803
02_AG_MA_2015_P140_15_KU553162
02AG_TN_2015_15TN1531_KX183804
02AG_TN_2022_TN-CVV6_PP483218
-_DZ_2013_19056CA_KT338948
02AG_TN_2014_14TN12295_KX183717
02_AG_MA_2014_P069_14_KU553164
02_AG_MA_2010_P57_MA0310_JN222617
02AG_TN_2015_15TN4555_KX183824
02AG_TN_2014_14TN8392_KX183795
02AG_TN_2014_14TN384_KX183753
02AG_TN_2022_TN-CVV177_PP483226
02AG_TN_2014_14TN3921_KX183754
02AG_TN_2013_KM006242_KM006242
02AG_TN_2013_KM006259_KM006259
02AG_TN_2015_15TN4873_KX183826
02AG_TN_2021_TN-CVV574_PP483233
02AG_TN_2020_TN-CVV365_PP483252
02AG_TN_2014_14TN4100_KX183756
02AG_TN_2014_14TN7627_KX183786
02AG_TN_2014_14TN6703_KX183770
02B_TN_2015_15TN1544_KX183808
02AG_TN_2021_TN-CVV361_PP483283
02AG_TN_2022_TN-CVV131_PP483225
02AG_TN_2021_TN-CVV172_PP483259
02AG_TN_2015_15TN403_KX183819
02AG_TN_2015_15TN3843_KX183817
02AG_TN_2013_KM006256_KM006256
02AG_TN_2021_TN-CVV201_PP483281
02AG_TN_2021_TN-CVV203_PP483263
02AG_TN_2021_TN-CVV800_PP483255
02AG_TN_2021_TN-CVV202_PP483277
02AG_TN_2015_15TN1541_KX183807
02AG_TN_2013_KM006233_KM006233
02AG_TN_2013_KM006229_KM006229
02_AG_OM_2024_218465_PX777697
A1_LB_1996_LE1_AF025721

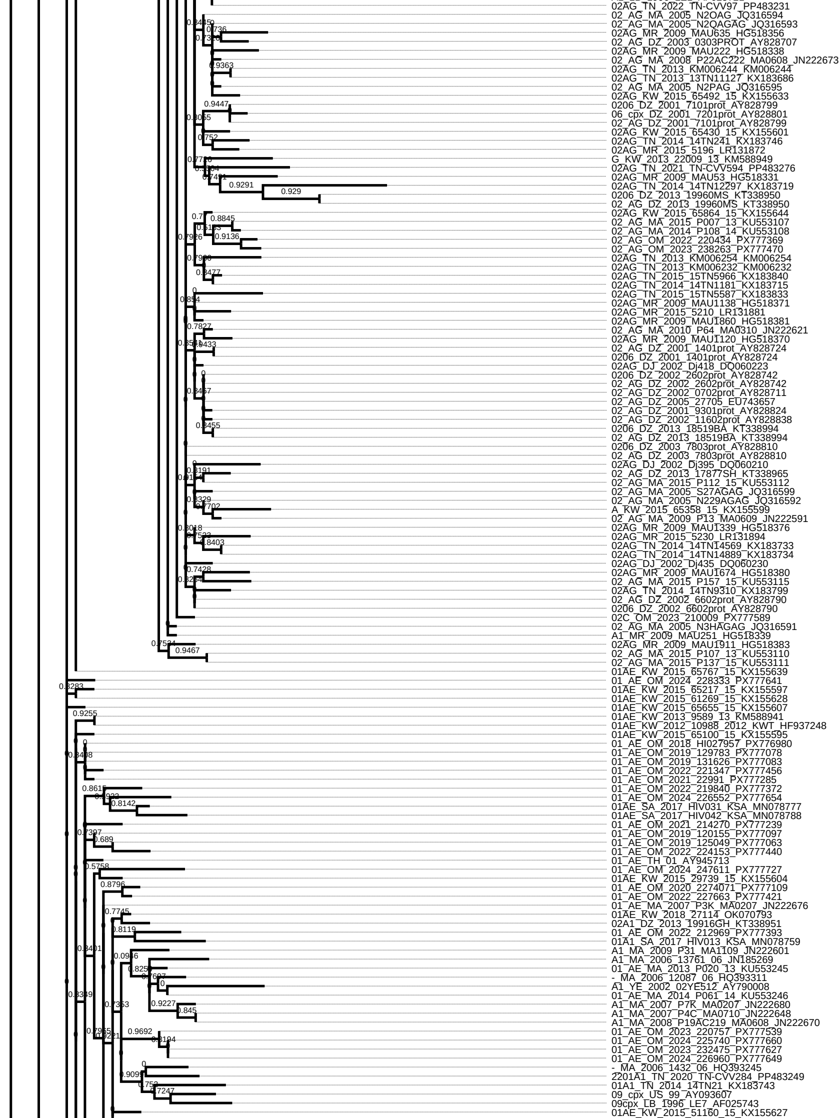

02AG_TN_2022_TN-CVV97_PP483231
02_AG_MA_2005_N2OAG_JQ316594
02_AG_MA_2005_N2QAGAG_JQ316593
02AG_MR_2009_MAU635_HG518356
02_AG_DZ_2003_0303PROT_AY828707
02AG_MR_2009_MAU222_HG518338
02_AG_MA_2008_P22AC222_MA0608_JN222673
02AG_TN_2013_KM006244_KM006244
02AG_TN_2013_13TN11127_KX183686
02_AG_MA_2005_N2PAG_JQ316595
02AG_KW_2015_65492_15_KX155633
0206_DZ_2001_7101prot_AY828799
06_cpx_DZ_2001_7201prot_AY828801
02_AG_DZ_2001_7101prot_AY828799
02AG_KW_2015_65430_15_KX155601
02AG_TN_2014_14TN241_KX183746
02AG_MR_2015_5196_LR131872
G_KW_2013_22009_13_KM588949
02AG_TN_2021_TN-CVV594_PP483276
02AG_MR_2009_MAU53_HG518331
02AG_TN_2014_14TN12297_KX183719
0206_DZ_2013_19960MS_KT338950
02_AG_DZ_2013_19960MS_KT338950
02AG_KW_2015_65864_15_KX155644
02_AG_MA_2015_P007_13_KU553107
02_AG_MA_2014_P108_14_KU553108
02_AG_OM_2022_220434_PX777369
02_AG_OM_2023_238263_PX777470
02AG_TN_2013_KM006254_KM006254
02AG_TN_2013_KM006232_KM006232
02AG_TN_2015_15TN5966_KX183840
02AG_TN_2014_14TN1181_KX183715
02AG_TN_2015_15TN5587_KX183833
02AG_MR_2009_MAU1138_HG518371
02AG_MR_2015_5210_LR131881
02AG_MR_2009_MAU1860_HG518381
02_AG_MA_2010_P64_MA0310_JN222621
02AG_MR_2009_MAU1120_HG518370
02_AG_DZ_2001_1401prot_AY828724
0206_DZ_2001_1401prot_AY828724
02AG_DJ_2002_Dj418_DQ060223
0206_DZ_2002_2602prot_AY828742
02_AG_DZ_2002_2602prot_AY828742
02_AG_DZ_2002_0702prot_AY828711
02_AG_DZ_2005_27705_EU743657
02_AG_DZ_2001_9301prot_AY828824
02_AG_DZ_2002_11602prot_AY828838
0206_DZ_2013_18519BA_KT338994
02_AG_DZ_2013_18519BA_KT338994
0206_DZ_2003_7803prot_AY828810
02_AG_DZ_2003_7803prot_AY828810
02AG_DJ_2002_Dj395_DQ060210
02_AG_DZ_2013_17877SH_KT338965
02_AG_MA_2015_P112_15_KU553112
02_AG_MA_2005_S27AGAG_JQ316599
02_AG_MA_2005_N229AGAG_JQ316592
A_KW_2015_65358_15_KX155599
02_AG_MA_2009_P13_MA0609_JN222591
02AG_MR_2009_MAU1339_HG518376
02AG_MR_2015_5230_LR131894
02AG_TN_2014_14TN14569_KX183733
02AG_TN_2014_14TN14889_KX183734
02AG_DJ_2002_Dj435_DQ060230
02AG_MR_2009_MAU1674_HG518380
02_AG_MA_2015_P157_15_KU553115
02AG_TN_2014_14TN9310_KX183799
02_AG_DZ_2002_6602prot_AY828790
0206_DZ_2002_6602prot_AY828790
02C_OM_2023_210009_PX777589
02_AG_MA_2005_N3HAGAG_JQ316591
A1_MR_2009_MAU251_HG518339
02AG_MR_2009_MAU1911_HG518383
02_AG_MA_2015_P107_13_KU553110
02_AG_MA_2015_P137_15_KU553111
01AE_KW_2015_65767_15_KX155639
01_AE_OM_2024_228333_PX777641
01AE_KW_2015_65217_15_KX155597
01AE_KW_2015_61269_15_KX155628
01AE_KW_2015_65655_15_KX155607
01AE_KW_2013_9589_13_KM588941
01AE_KW_2012_10988_2012_KWT_HF937248
01AE_KW_2015_65100_15_KX155595
01_AE_OM_2018_HI027957_PX776980
01_AE_OM_2019_129783_PX777078
01_AE_OM_2019_131626_PX777083
01_AE_OM_2022_221347_PX777456
01_AE_OM_2021_22991_PX777285
01_AE_OM_2022_219840_PX777372
01_AE_OM_2024_226552_PX777654
01AE_SA_2017_HIV031_KSA_MN078777
01AE_SA_2017_HIV042_KSA_MN078788
01_AE_OM_2021_214270_PX777239
01_AE_OM_2019_120155_PX777097
01_AE_OM_2019_125049_PX777063
01_AE_OM_2022_224153_PX777440
01_AE_TH_01_AY945713
01_AE_OM_2024_247611_PX777727
01AE_KW_2015_29739_15_KX155604
01_AE_OM_2020_2274071_PX777109
01_AE_OM_2022_227663_PX777421
01_AE_MA_2007_P3K_MA0207_JN222676
01AE_KW_2018_27114_OK070793
02A1_DZ_2013_19916GH_KT338951
01_AE_OM_2022_212969_PX777393
01A1_SA_2017_HIV013_KSA_MN078759
A1_MA_2009_P31_MA1109_JN222601
A1_MA_2006_13761_06_JN185269
01_AE_MA_2013_P020_13_KU553245
-_MA_2006_12087_06_HQ393311
A1_YE_2002_02YE512_AY790008
01_AE_MA_2014_P061_14_KU553246
A1_MA_2007_P7K_MA0207_JN222680
A1_MA_2007_P4C_MA0710_JN222648
A1_MA_2008_P19AC219_MA0608_JN222670
01_AE_OM_2023_220757_PX777539
01_AE_OM_2024_225740_PX777660
01_AE_OM_2023_232475_PX777627
01_AE_OM_2024_226960_PX777649
-_MA_2006_1432_06_HQ393245
2201A1_TN_2020_TN-CVV284_PP483249
01A1_TN_2014_14TN21_KX183743
09_cpx_US_99_AY093607
09cpx_LB_1996_LE7_AF025743
01AE_KW_2015_51160_15_KX155627

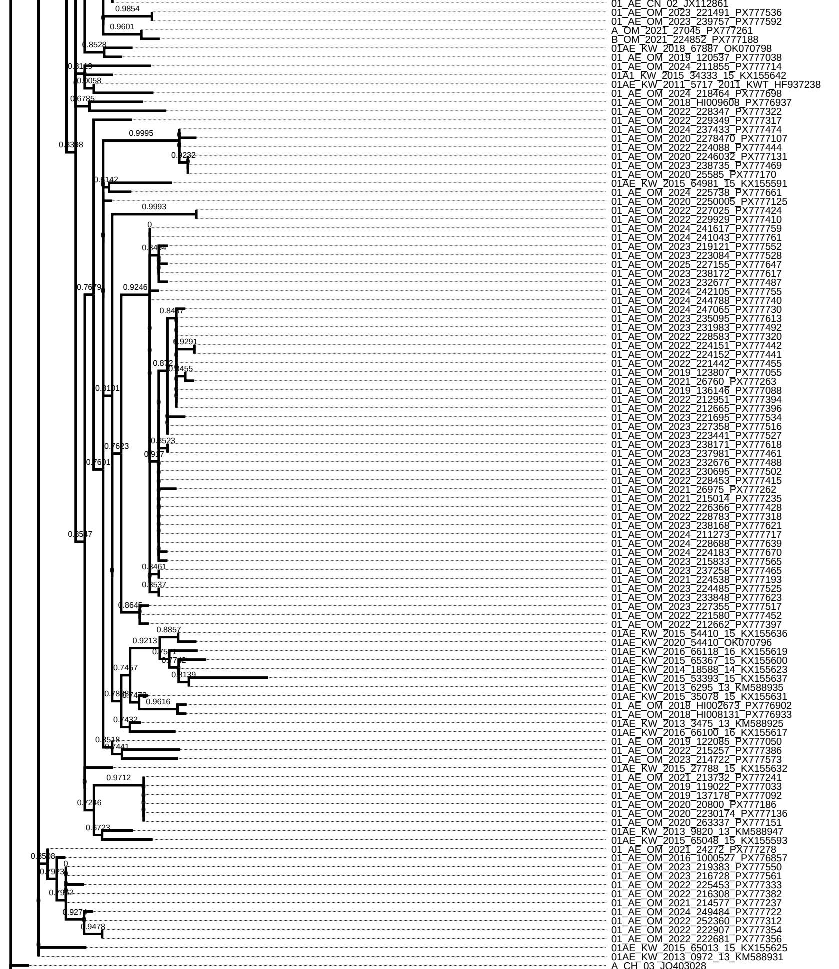
01_AE_CN_02_JX112861
01_AE_OM_2023_221491_PX777536
01_AE_OM_2023_239757_PX777592
A_OM_2021_27045_PX777261
B_OM_2021_224852_PX777188
01AE_KW_2018_67887_OK070798
01_AE_OM_2019_120537_PX777038
01_AE_OM_2024_211855_PX777714
01A1_KW_2015_34333_15_KX155642
01AE_KW_2011_5717_2011_KWT_HF937238
01_AE_OM_2024_218464_PX777698
01_AE_OM_2018_HI009608_PX776937
01_AE_OM_2022_228347_PX777322
01_AE_OM_2022_229349_PX777317
01_AE_OM_2024_237433_PX777474
01_AE_OM_2020_2278470_PX777107
01_AE_OM_2022_224088_PX777444
01_AE_OM_2020_2246032_PX777131
01_AE_OM_2023_238735_PX777469
01_AE_OM_2020_25585_PX777170
01AE_KW_2015_64981_15_KX155591
01_AE_OM_2024_225738_PX777661
01_AE_OM_2020_2250005_PX777125
01_AE_OM_2022_227025_PX777424
01_AE_OM_2022_229929_PX777410
01_AE_OM_2024_241617_PX777759
01_AE_OM_2024_241043_PX777761
01_AE_OM_2023_219121_PX777552
01_AE_OM_2023_223084_PX777528
01_AE_OM_2025_227155_PX777647
01_AE_OM_2023_238172_PX777617
01_AE_OM_2023_232677_PX777487
01_AE_OM_2024_242105_PX777755
01_AE_OM_2024_244788_PX777740
01_AE_OM_2024_247065_PX777730
01_AE_OM_2023_235095_PX777613
01_AE_OM_2023_231983_PX777492
01_AE_OM_2022_228583_PX777320
01_AE_OM_2022_224151_PX777442
01_AE_OM_2022_224152_PX777441
01_AE_OM_2022_221442_PX777455
01_AE_OM_2019_123807_PX777055
01_AE_OM_2021_26760_PX777263
01_AE_OM_2019_136146_PX777088
01_AE_OM_2022_212951_PX777394
01_AE_OM_2022_212665_PX777396
01_AE_OM_2023_221695_PX777534
01_AE_OM_2023_227358_PX777516
01_AE_OM_2023_223441_PX777527
01_AE_OM_2023_238171_PX777618
01_AE_OM_2023_237981_PX777461
01_AE_OM_2023_232676_PX777488
01_AE_OM_2023_230695_PX777502
01_AE_OM_2022_228453_PX777415
01_AE_OM_2021_26975_PX777262
01_AE_OM_2021_215014_PX777235
01_AE_OM_2022_226366_PX777428
01_AE_OM_2022_228783_PX777318
01_AE_OM_2023_238168_PX777621
01_AE_OM_2024_211273_PX777717
01_AE_OM_2024_228688_PX777639
01_AE_OM_2024_224183_PX777670
01_AE_OM_2023_215833_PX777565
01_AE_OM_2023_237258_PX777465
01_AE_OM_2021_224538_PX777193
01_AE_OM_2023_224485_PX777525
01_AE_OM_2023_233848_PX777623
01_AE_OM_2023_227355_PX777517
01_AE_OM_2022_221580_PX777452
01_AE_OM_2022_212662_PX777397
01AE_KW_2015_54410_15_KX155636
01AE_KW_2020_54410_OK070796
01AE_KW_2016_66118_16_KX155619
01AE_KW_2015_65367_15_KX155600
01AE_KW_2014_18588_14_KX155623
01AE_KW_2015_53393_15_KX155637
01AE_KW_2013_6295_13_KM588935
01AE_KW_2015_35078_15_KX155631
01_AE_OM_2018_HI002673_PX776902
01_AE_OM_2018_HI008131_PX776933
01AE_KW_2013_3475_13_KM588925
01AE_KW_2016_66100_16_KX155617
01_AE_OM_2019_122085_PX777050
01_AE_OM_2022_215257_PX777386
01_AE_OM_2023_214722_PX777573
01AE_KW_2015_27788_15_KX155632
01_AE_OM_2021_213732_PX777241
01_AE_OM_2019_119022_PX777033
01_AE_OM_2019_137178_PX777092
01_AE_OM_2020_20800_PX777186
01_AE_OM_2020_2230174_PX777136
01_AE_OM_2020_263337_PX777151
01AE_KW_2013_9820_13_KM588947
01AE_KW_2015_65048_15_KX155593
01_AE_OM_2021_24272_PX777278
01_AE_OM_2016_1000527_PX776857
01_AE_OM_2023_219383_PX777550
01_AE_OM_2023_216728_PX777561
01_AE_OM_2022_225453_PX777333
01_AE_OM_2022_216308_PX777382
01_AE_OM_2021_214577_PX777237
01_AE_OM_2024_249484_PX777722
01_AE_OM_2022_252360_PX777312
01_AE_OM_2022_222907_PX777354
01_AE_OM_2022_222681_PX777356
01AE_KW_2015_65013_15_KX155625
01AE_KW_2013_0972_13_KM588931
A_CH_03_JQ403028

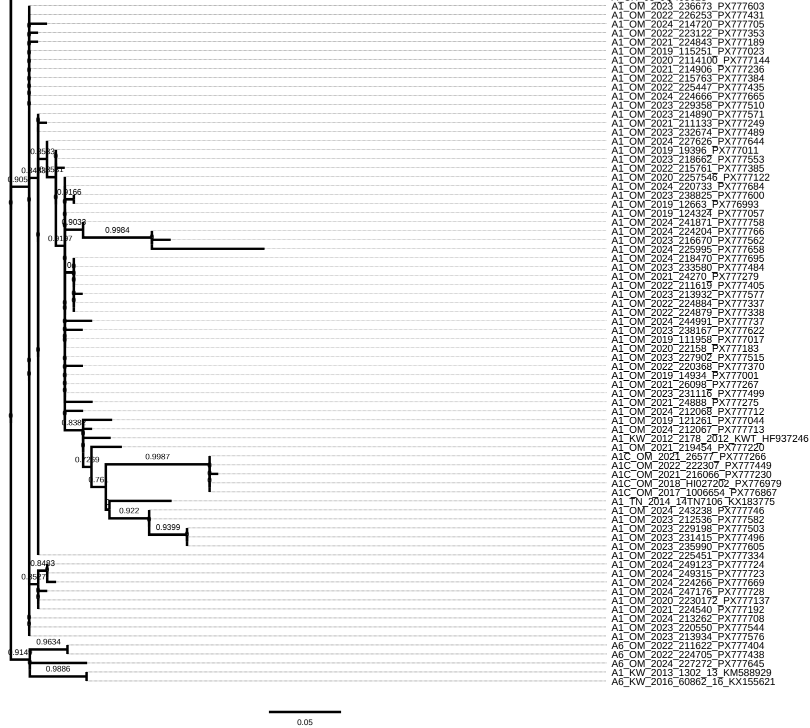
A1_OM_2023_236673_PX777603
A1_OM_2022_226253_PX777431
A1_OM_2024_214720_PX777705
A1_OM_2022_223122_PX777353
A1_OM_2021_224843_PX777189
A1_OM_2019_115251_PX777023
A1_OM_2020_2114100_PX777144
A1_OM_2021_214906_PX777236
A1_OM_2022_215763_PX777384
A1_OM_2022_225447_PX777435
A1_OM_2024_224666_PX777665
A1_OM_2023_229358_PX777510
A1_OM_2023_214890_PX777571
A1_OM_2021_211133_PX777249
A1_OM_2023_232674_PX777489
A1_OM_2024_227626_PX777644
A1_OM_2019_19396_PX777011
A1_OM_2023_218662_PX777553
A1_OM_2022_215761_PX777385
A1_OM_2020_2257546_PX777122
A1_OM_2024_220733_PX777684
A1_OM_2023_238825_PX777600
A1_OM_2019_12663_PX776993
A1_OM_2019_124324_PX777057
A1_OM_2024_241871_PX777758
A1_OM_2024_224204_PX777766
A1_OM_2023_216670_PX777562
A1_OM_2024_225995_PX777658
A1_OM_2024_218470_PX777695
A1_OM_2023_233580_PX777484
A1_OM_2021_24270_PX777279
A1_OM_2022_211619_PX777405
A1_OM_2023_213932_PX777577
A1_OM_2022_224884_PX777337
A1_OM_2022_224879_PX777338
A1_OM_2024_244991_PX777737
A1_OM_2023_238167_PX777622
A1_OM_2019_111958_PX777017
A1_OM_2020_22158_PX777183
A1_OM_2023_227902_PX777515
A1_OM_2022_220368_PX777370
A1_OM_2019_14934_PX777001
A1_OM_2021_26098_PX777267
A1_OM_2023_231116_PX777499
A1_OM_2021_24888_PX777275
A1_OM_2024_212068_PX777712
A1_OM_2019_121261_PX777044
A1_OM_2024_212067_PX777713
A1_KW_2012_2178_2012_KWT_HF937246
A1_OM_2021_219454_PX777220
A1C_OM_2021_26577_PX777266
A1C_OM_2022_222307_PX777449
A1C_OM_2021_216066_PX777230
A1C_OM_2018_HI027202_PX776979
A1C_OM_2017_1006654_PX776867
A1_TN_2014_14TN7106_KX183775
A1_OM_2024_243238_PX777746
A1_OM_2023_212536_PX777582
A1_OM_2023_229198_PX777503
A1_OM_2023_231415_PX777496
A1_OM_2023_235990_PX777605
A1_OM_2022_225451_PX777334
A1_OM_2024_249123_PX777724
A1_OM_2024_249315_PX777723
A1_OM_2024_224266_PX777669
A1_OM_2024_247176_PX777728
A1_OM_2020_2230172_PX777137
A1_OM_2021_224540_PX777192
A1_OM_2024_213262_PX777708
A1_OM_2023_220550_PX777544
A1_OM_2023_213934_PX777576
A6_OM_2022_211622_PX777404
A6_OM_2022_224705_PX777438
A6_OM_2024_227272_PX777645
A1_KW_2013_1302_13_KM588929
A6_KW_2016_60862_16_KX155621
0.905
0.9166
0.9033
0.9984
0.9197
0.8382
0.7269
0.9987
0.761
0.922
0.9399
0.8483
0.8527
0.9634
0.914
0.9886
0.05

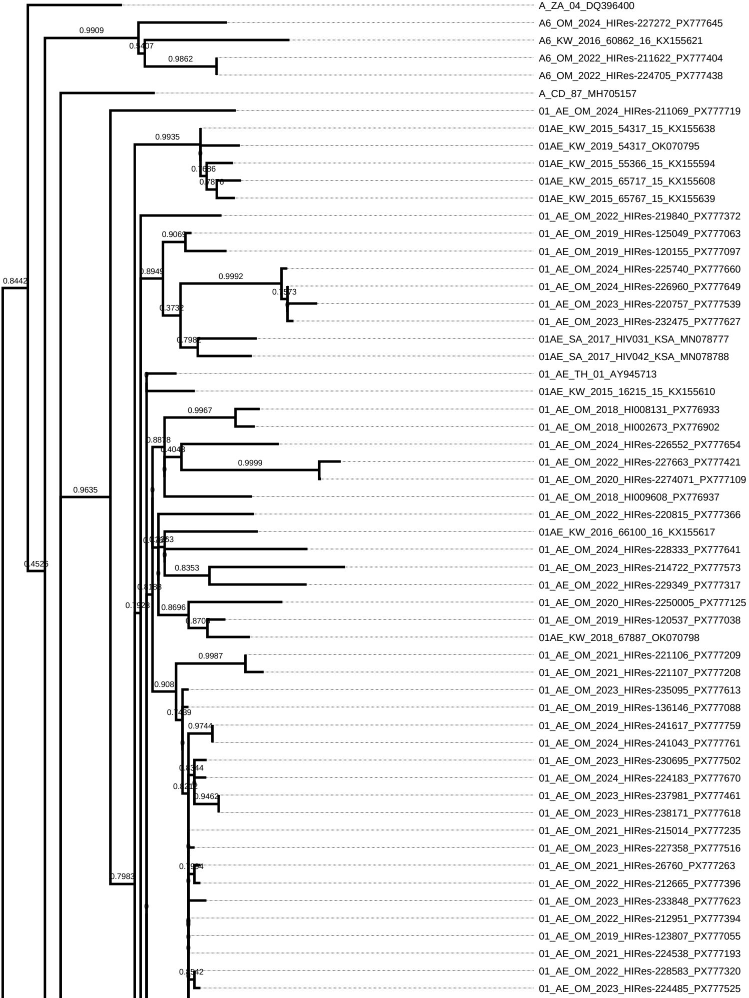
A_ZA_04_DQ396400
A6_OM_2024_HIRes-227272_PX777645
A6_KW_2016_60862_16_KX155621
A6_OM_2022_HIRes-211622_PX777404
A6_OM_2022_HIRes-224705_PX777438
A_CD_87_MH705157
01_AE_OM_2024_HIRes-211069_PX777719
01AE_KW_2015_54317_15_KX155638
01AE_KW_2019_54317_OK070795
01AE_KW_2015_55366_15_KX155594
01AE_KW_2015_65717_15_KX155608
01AE_KW_2015_65767_15_KX155639
01_AE_OM_2022_HIRes-219840_PX777372
01_AE_OM_2019_HIRes-125049_PX777063
01_AE_OM_2019_HIRes-120155_PX777097
01_AE_OM_2024_HIRes-225740_PX777660
01_AE_OM_2024_HIRes-226960_PX777649
01_AE_OM_2023_HIRes-220757_PX777539
01_AE_OM_2023_HIRes-232475_PX777627
01AE_SA_2017_HIV031_KSA_MN078777
01AE_SA_2017_HIV042_KSA_MN078788
01_AE_TH_01_AY945713
01AE_KW_2015_16215_15_KX155610
01_AE_OM_2018_HI008131_PX776933
01_AE_OM_2018_HI002673_PX776902
01_AE_OM_2024_HIRes-226552_PX777654
01_AE_OM_2022_HIRes-227663_PX777421
01_AE_OM_2020_HIRes-2274071_PX777109
01_AE_OM_2018_HI009608_PX776937
01_AE_OM_2022_HIRes-220815_PX777366
01AE_KW_2016_66100_16_KX155617
01_AE_OM_2024_HIRes-228333_PX777641
01_AE_OM_2023_HIRes-214722_PX777573
01_AE_OM_2022_HIRes-229349_PX777317
01_AE_OM_2020_HIRes-2250005_PX777125
01_AE_OM_2019_HIRes-120537_PX777038
01AE_KW_2018_67887_OK070798
01_AE_OM_2021_HIRes-221106_PX777209
01_AE_OM_2021_HIRes-221107_PX777208
01_AE_OM_2023_HIRes-235095_PX777613
01_AE_OM_2019_HIRes-136146_PX777088
01_AE_OM_2024_HIRes-241617_PX777759
01_AE_OM_2024_HIRes-241043_PX777761
01_AE_OM_2023_HIRes-230695_PX777502
01_AE_OM_2024_HIRes-224183_PX777670
01_AE_OM_2023_HIRes-237981_PX777461
01_AE_OM_2023_HIRes-238171_PX777618
01_AE_OM_2021_HIRes-215014_PX777235
01_AE_OM_2023_HIRes-227358_PX777516
01_AE_OM_2021_HIRes-26760_PX777263
01_AE_OM_2022_HIRes-212665_PX777396
01_AE_OM_2023_HIRes-233848_PX777623
01_AE_OM_2022_HIRes-212951_PX777394
01_AE_OM_2019_HIRes-123807_PX777055
01_AE_OM_2021_HIRes-224538_PX777193
01_AE_OM_2022_HIRes-228583_PX777320
01_AE_OM_2023_HIRes-224485_PX777525
0.9909
0.9862
0.9935
0.9069
0.8949
0.9992
0.8442
0.9967
0.9999
0.9635
0.8353
0.8696
0.9987
0.908
0.9744
0.9462
0.7983
0.4526

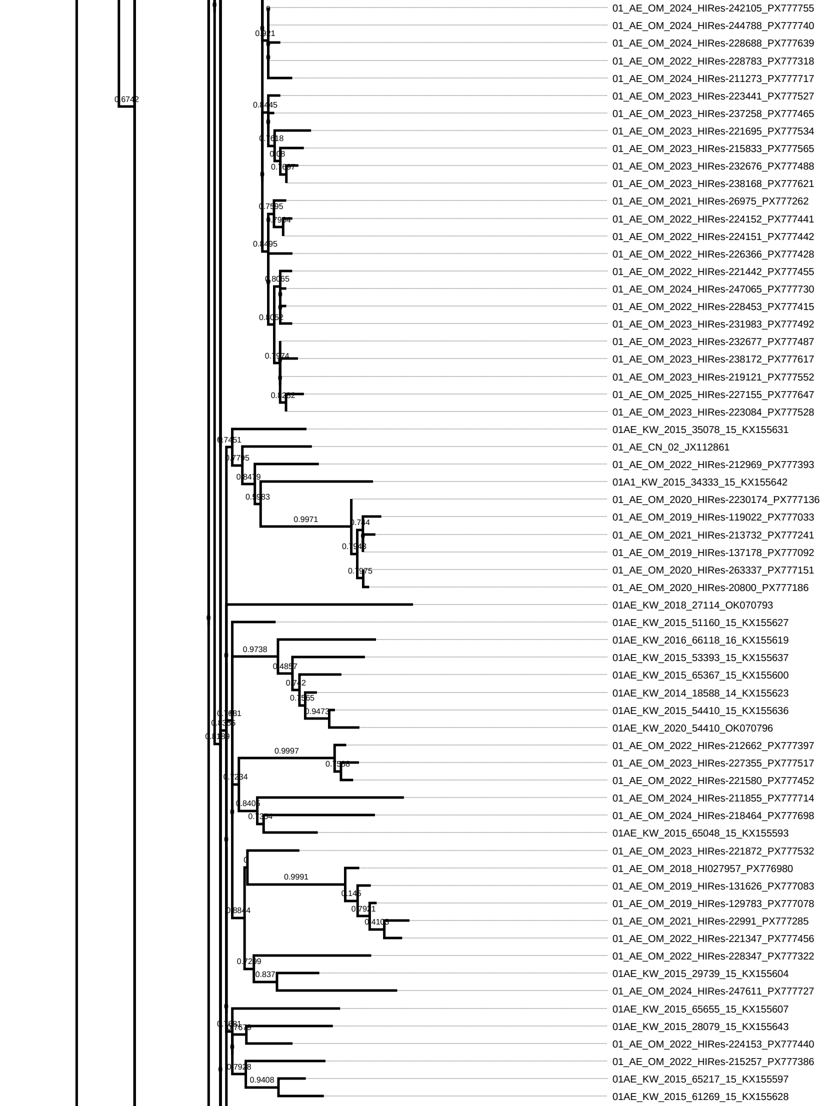
01_AE_OM_2024_HIRes-242105_PX777755
01_AE_OM_2024_HIRes-244788_PX777740
01_AE_OM_2024_HIRes-228688_PX777639
01_AE_OM_2022_HIRes-228783_PX777318
01_AE_OM_2024_HIRes-211273_PX777717
01_AE_OM_2023_HIRes-223441_PX777527
01_AE_OM_2023_HIRes-237258_PX777465
01_AE_OM_2023_HIRes-221695_PX777534
01_AE_OM_2023_HIRes-215833_PX777565
01_AE_OM_2023_HIRes-232676_PX777488
01_AE_OM_2023_HIRes-238168_PX777621
01_AE_OM_2021_HIRes-26975_PX777262
01_AE_OM_2022_HIRes-224152_PX777441
01_AE_OM_2022_HIRes-224151_PX777442
01_AE_OM_2022_HIRes-226366_PX777428
01_AE_OM_2022_HIRes-221442_PX777455
01_AE_OM_2024_HIRes-247065_PX777730
01_AE_OM_2022_HIRes-228453_PX777415
01_AE_OM_2023_HIRes-231983_PX777492
01_AE_OM_2023_HIRes-232677_PX777487
01_AE_OM_2023_HIRes-238172_PX777617
01_AE_OM_2023_HIRes-219121_PX777552
01_AE_OM_2025_HIRes-227155_PX777647
01_AE_OM_2023_HIRes-223084_PX777528
01AE_KW_2015_35078_15_KX155631
01_AE_CN_02_JX112861
01_AE_OM_2022_HIRes-212969_PX777393
01A1_KW_2015_34333_15_KX155642
01_AE_OM_2020_HIRes-2230174_PX777136
01_AE_OM_2019_HIRes-119022_PX777033
01_AE_OM_2021_HIRes-213732_PX777241
01_AE_OM_2019_HIRes-137178_PX777092
01_AE_OM_2020_HIRes-263337_PX777151
01_AE_OM_2020_HIRes-20800_PX777186
01AE_KW_2018_27114_OK070793
01AE_KW_2015_51160_15_KX155627
01AE_KW_2016_66118_16_KX155619
01AE_KW_2015_53393_15_KX155637
01AE_KW_2015_65367_15_KX155600
01AE_KW_2014_18588_14_KX155623
01AE_KW_2015_54410_15_KX155636
01AE_KW_2020_54410_OK070796
01_AE_OM_2022_HIRes-212662_PX777397
01_AE_OM_2023_HIRes-227355_PX777517
01_AE_OM_2022_HIRes-221580_PX777452
01_AE_OM_2024_HIRes-211855_PX777714
01_AE_OM_2024_HIRes-218464_PX777698
01AE_KW_2015_65048_15_KX155593
01_AE_OM_2023_HIRes-221872_PX777532
01_AE_OM_2018_HI027957_PX776980
01_AE_OM_2019_HIRes-131626_PX777083
01_AE_OM_2019_HIRes-129783_PX777078
01_AE_OM_2021_HIRes-22991_PX777285
01_AE_OM_2022_HIRes-221347_PX777456
01_AE_OM_2022_HIRes-228347_PX777322
01AE_KW_2015_29739_15_KX155604
01_AE_OM_2024_HIRes-247611_PX777727
01AE_KW_2015_65655_15_KX155607
01AE_KW_2015_28079_15_KX155643
01_AE_OM_2022_HIRes-224153_PX777440
01_AE_OM_2022_HIRes-215257_PX777386
01AE_KW_2015_65217_15_KX155597
01AE_KW_2015_61269_15_KX155628
0.6742
0.9971
0.9738
0.9997
0.9991
0.9408

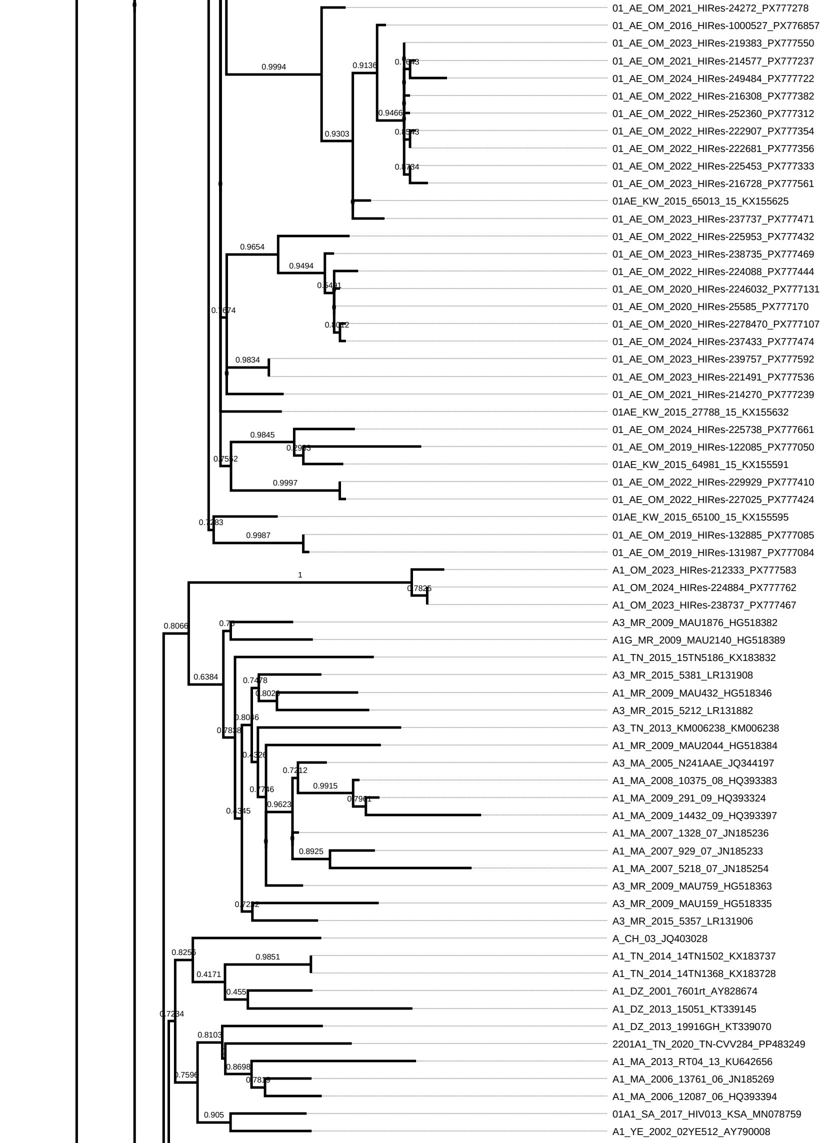
01_AE_OM_2021_HIRes-24272_PX777278
01_AE_OM_2016_HIRes-1000527_PX776857
01_AE_OM_2023_HIRes-219383_PX777550
01_AE_OM_2021_HIRes-214577_PX777237
01_AE_OM_2024_HIRes-249484_PX777722
01_AE_OM_2022_HIRes-216308_PX777382
01_AE_OM_2022_HIRes-252360_PX777312
01_AE_OM_2022_HIRes-222907_PX777354
01_AE_OM_2022_HIRes-222681_PX777356
01_AE_OM_2022_HIRes-225453_PX777333
01_AE_OM_2023_HIRes-216728_PX777561
01AE_KW_2015_65013_15_KX155625
01_AE_OM_2023_HIRes-237737_PX777471
01_AE_OM_2022_HIRes-225953_PX777432
01_AE_OM_2023_HIRes-238735_PX777469
01_AE_OM_2022_HIRes-224088_PX777444
01_AE_OM_2020_HIRes-2246032_PX777131
01_AE_OM_2020_HIRes-25585_PX777170
01_AE_OM_2020_HIRes-2278470_PX777107
01_AE_OM_2024_HIRes-237433_PX777474
01_AE_OM_2023_HIRes-239757_PX777592
01_AE_OM_2023_HIRes-221491_PX777536
01_AE_OM_2021_HIRes-214270_PX777239
01AE_KW_2015_27788_15_KX155632
01_AE_OM_2024_HIRes-225738_PX777661
01_AE_OM_2019_HIRes-122085_PX777050
01AE_KW_2015_64981_15_KX155591
01_AE_OM_2022_HIRes-229929_PX777410
01_AE_OM_2022_HIRes-227025_PX777424
01AE_KW_2015_65100_15_KX155595
01_AE_OM_2019_HIRes-132885_PX777085
01_AE_OM_2019_HIRes-131987_PX777084
A1_OM_2023_HIRes-212333_PX777583
A1_OM_2024_HIRes-224884_PX777762
A1_OM_2023_HIRes-238737_PX777467
A3_MR_2009_MAU1876_HG518382
A1G_MR_2009_MAU2140_HG518389
A1_TN_2015_15TN5186_KX183832
A3_MR_2015_5381_LR131908
A1_MR_2009_MAU432_HG518346
A3_MR_2015_5212_LR131882
A3_TN_2013_KM006238_KM006238
A1_MR_2009_MAU2044_HG518384
A3_MA_2005_N241AAE_JQ344197
A1_MA_2008_10375_08_HQ393383
A1_MA_2009_291_09_HQ393324
A1_MA_2009_14432_09_HQ393397
A1_MA_2007_1328_07_JN185236
A1_MA_2007_929_07_JN185233
A1_MA_2007_5218_07_JN185254
A3_MR_2009_MAU759_HG518363
A3_MR_2009_MAU159_HG518335
A3_MR_2015_5357_LR131906
A_CH_03_JQ403028
A1_TN_2014_14TN1502_KX183737
A1_TN_2014_14TN1368_KX183728
A1_DZ_2001_7601rt_AY828674
A1_DZ_2013_15051_KT339145
A1_DZ_2013_19916GH_KT339070
2201A1_TN_2020_TN-CVV284_PP483249
A1_MA_2013_RT04_13_KU642656
A1_MA_2006_13761_06_JN185269
A1_MA_2006_12087_06_HQ393394
01A1_SA_2017_HIV013_KSA_MN078759
A1_YE_2002_02YE512_AY790008
0.9994
0.9136
0.9466
0.9303
0.8734
0.9654
0.9494
0.9834
0.9845
0.7552
0.9997
0.7283
0.9987
1
0.7825
0.8066
0.6384
0.7478
0.8026
0.8046
0.7838
0.7212
0.9915
0.7746
0.9623
0.8925
0.7232
0.8256
0.9851
0.4171
0.455
0.7284
0.8103
0.8698
0.7596
0.905

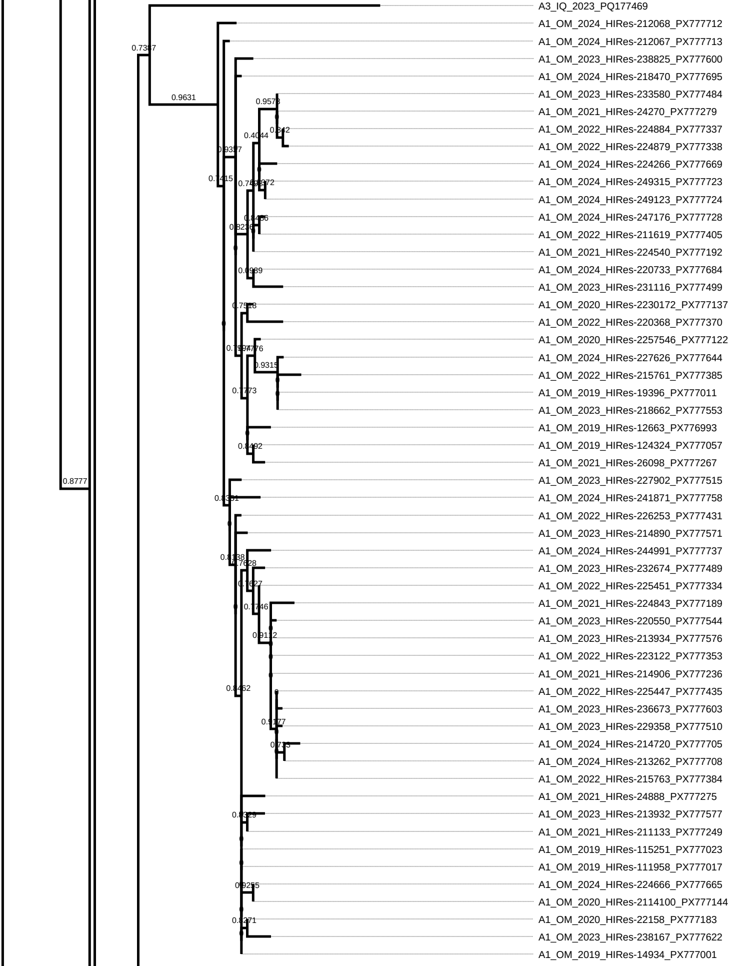
0.7387
0.9631
0.8777
A3_IQ_2023_PQ177469
A1_OM_2024_HIRes-212068_PX777712
A1_OM_2024_HIRes-212067_PX777713
A1_OM_2023_HIRes-238825_PX777600
A1_OM_2024_HIRes-218470_PX777695
A1_OM_2023_HIRes-233580_PX777484
A1_OM_2021_HIRes-24270_PX777279
A1_OM_2022_HIRes-224884_PX777337
A1_OM_2022_HIRes-224879_PX777338
A1_OM_2024_HIRes-224266_PX777669
A1_OM_2024_HIRes-249315_PX777723
A1_OM_2024_HIRes-249123_PX777724
A1_OM_2024_HIRes-247176_PX777728
A1_OM_2022_HIRes-211619_PX777405
A1_OM_2021_HIRes-224540_PX777192
A1_OM_2024_HIRes-220733_PX777684
A1_OM_2023_HIRes-231116_PX777499
A1_OM_2020_HIRes-2230172_PX777137
A1_OM_2022_HIRes-220368_PX777370
A1_OM_2020_HIRes-2257546_PX777122
A1_OM_2024_HIRes-227626_PX777644
A1_OM_2022_HIRes-215761_PX777385
A1_OM_2019_HIRes-19396_PX777011
A1_OM_2023_HIRes-218662_PX777553
A1_OM_2019_HIRes-12663_PX776993
A1_OM_2019_HIRes-124324_PX777057
A1_OM_2021_HIRes-26098_PX777267
A1_OM_2023_HIRes-227902_PX777515
A1_OM_2024_HIRes-241871_PX777758
A1_OM_2022_HIRes-226253_PX777431
A1_OM_2023_HIRes-214890_PX777571
A1_OM_2024_HIRes-244991_PX777737
A1_OM_2023_HIRes-232674_PX777489
A1_OM_2022_HIRes-225451_PX777334
A1_OM_2021_HIRes-224843_PX777189
A1_OM_2023_HIRes-220550_PX777544
A1_OM_2023_HIRes-213934_PX777576
A1_OM_2022_HIRes-223122_PX777353
A1_OM_2021_HIRes-214906_PX777236
A1_OM_2022_HIRes-225447_PX777435
A1_OM_2023_HIRes-236673_PX777603
A1_OM_2023_HIRes-229358_PX777510
A1_OM_2024_HIRes-214720_PX777705
A1_OM_2024_HIRes-213262_PX777708
A1_OM_2022_HIRes-215763_PX777384
A1_OM_2021_HIRes-24888_PX777275
A1_OM_2023_HIRes-213932_PX777577
A1_OM_2021_HIRes-211133_PX777249
A1_OM_2019_HIRes-115251_PX777023
A1_OM_2019_HIRes-111958_PX777017
A1_OM_2024_HIRes-224666_PX777665
A1_OM_2020_HIRes-2114100_PX777144
A1_OM_2020_HIRes-22158_PX777183
A1_OM_2023_HIRes-238167_PX777622
A1_OM_2019_HIRes-14934_PX777001

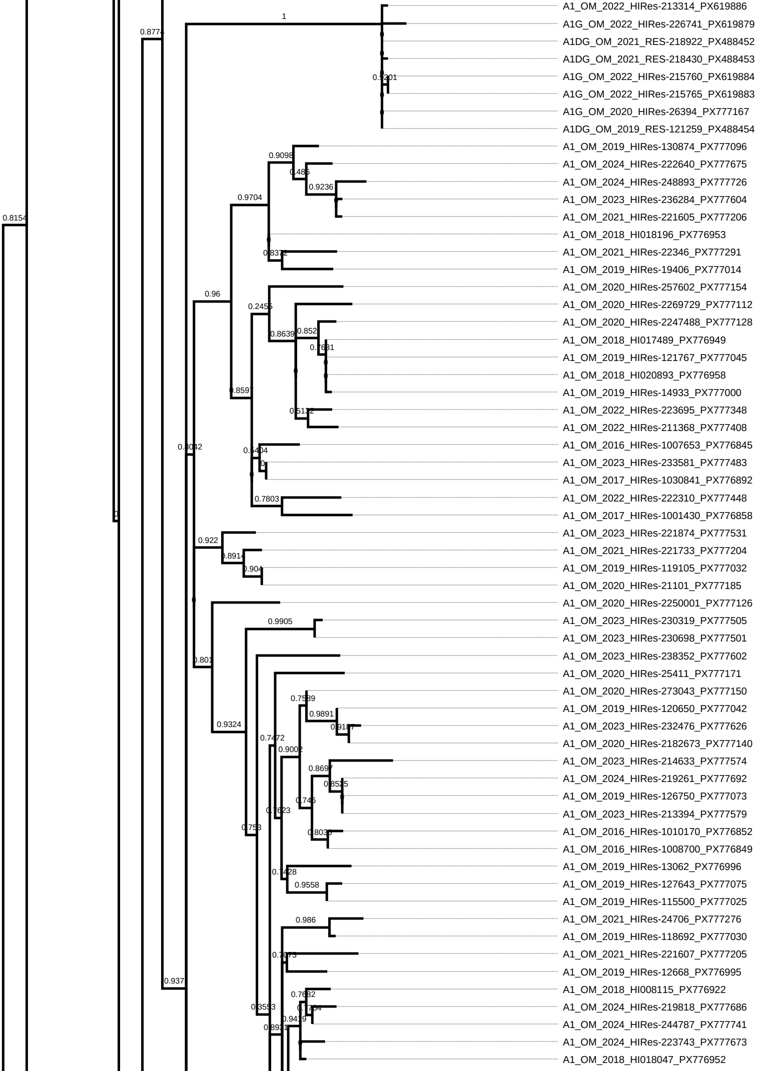

1
0.8774
0.9201
0.8154
0.9098
0.9704
0.9236
0.96
0.2455
0.8639
0.852
0.8042
0.7803
0.922
0.9905
0.9324
0.7589
0.9891
0.7472
0.9002
0.8697
0.746
0.9558
0.986
0.937
0.7682
0.9419
A1_OM_2022_HIRes-213314_PX619886
A1G_OM_2022_HIRes-226741_PX619879
A1DG_OM_2021_RES-218922_PX488452
A1DG_OM_2021_RES-218430_PX488453
A1G_OM_2022_HIRes-215760_PX619884
A1G_OM_2022_HIRes-215765_PX619883
A1G_OM_2020_HIRes-26394_PX777167
A1DG_OM_2019_RES-121259_PX488454
A1_OM_2019_HIRes-130874_PX777096
A1_OM_2024_HIRes-222640_PX777675
A1_OM_2024_HIRes-248893_PX777726
A1_OM_2023_HIRes-236284_PX777604
A1_OM_2021_HIRes-221605_PX777206
A1_OM_2018_HI018196_PX776953
A1_OM_2021_HIRes-22346_PX777291
A1_OM_2019_HIRes-19406_PX777014
A1_OM_2020_HIRes-257602_PX777154
A1_OM_2020_HIRes-2269729_PX777112
A1_OM_2020_HIRes-2247488_PX777128
A1_OM_2018_HI017489_PX776949
A1_OM_2019_HIRes-121767_PX777045
A1_OM_2018_HI020893_PX776958
A1_OM_2019_HIRes-14933_PX777000
A1_OM_2022_HIRes-223695_PX777348
A1_OM_2022_HIRes-211368_PX777408
A1_OM_2016_HIRes-1007653_PX776845
A1_OM_2023_HIRes-233581_PX777483
A1_OM_2017_HIRes-1030841_PX776892
A1_OM_2022_HIRes-222310_PX777448
A1_OM_2017_HIRes-1001430_PX776858
A1_OM_2023_HIRes-221874_PX777531
A1_OM_2021_HIRes-221733_PX777204
A1_OM_2019_HIRes-119105_PX777032
A1_OM_2020_HIRes-21101_PX777185
A1_OM_2020_HIRes-2250001_PX777126
A1_OM_2023_HIRes-230319_PX777505
A1_OM_2023_HIRes-230698_PX777501
A1_OM_2023_HIRes-238352_PX777602
A1_OM_2020_HIRes-25411_PX777171
A1_OM_2020_HIRes-273043_PX777150
A1_OM_2019_HIRes-120650_PX777042
A1_OM_2023_HIRes-232476_PX777626
A1_OM_2020_HIRes-2182673_PX777140
A1_OM_2023_HIRes-214633_PX777574
A1_OM_2024_HIRes-219261_PX777692
A1_OM_2019_HIRes-126750_PX777073
A1_OM_2023_HIRes-213394_PX777579
A1_OM_2016_HIRes-1010170_PX776852
A1_OM_2016_HIRes-1008700_PX776849
A1_OM_2019_HIRes-13062_PX776996
A1_OM_2019_HIRes-127643_PX777075
A1_OM_2019_HIRes-115500_PX777025
A1_OM_2021_HIRes-24706_PX777276
A1_OM_2019_HIRes-118692_PX777030
A1_OM_2021_HIRes-221607_PX777205
A1_OM_2019_HIRes-12668_PX776995
A1_OM_2018_HI008115_PX776922
A1_OM_2024_HIRes-219818_PX777686
A1_OM_2024_HIRes-244787_PX777741
A1_OM_2024_HIRes-223743_PX777673
A1_OM_2018_HI018047_PX776952

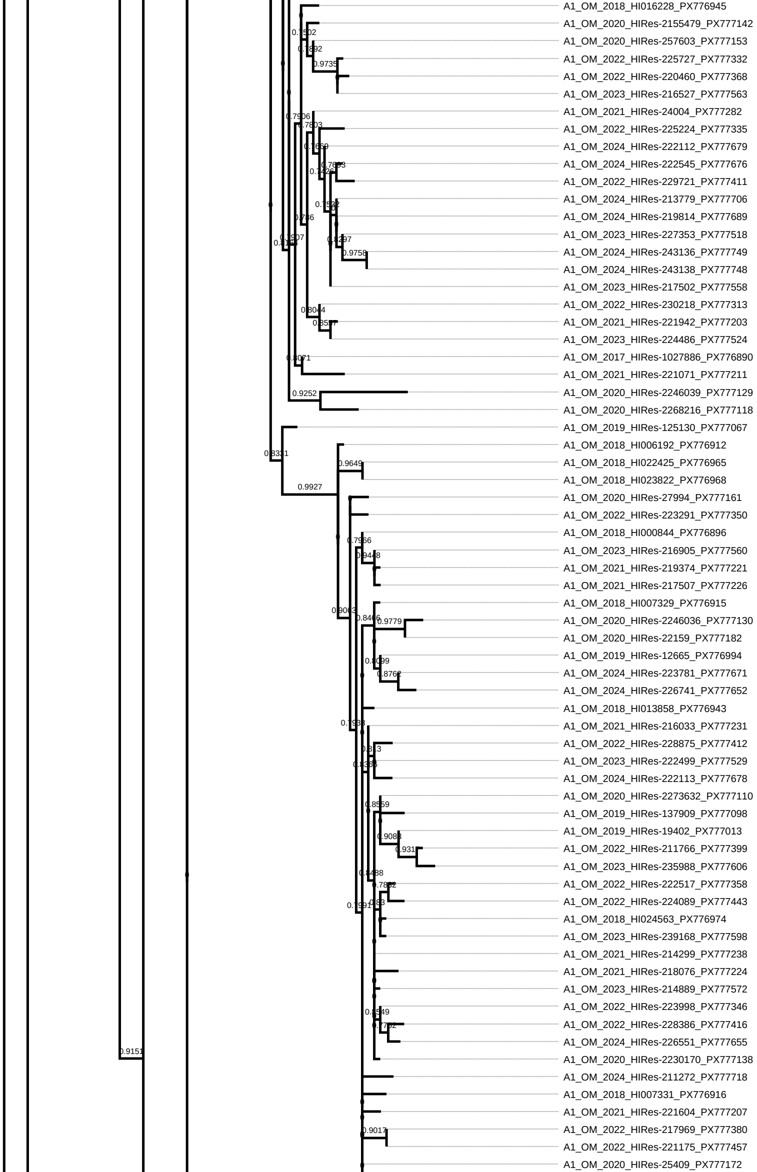
A1_OM_2018_HI016228_PX776945
A1_OM_2020_HIRes-2155479_PX777142
A1_OM_2020_HIRes-257603_PX777153
A1_OM_2022_HIRes-225727_PX777332
A1_OM_2022_HIRes-220460_PX777368
A1_OM_2023_HIRes-216527_PX777563
A1_OM_2021_HIRes-24004_PX777282
A1_OM_2022_HIRes-225224_PX777335
A1_OM_2024_HIRes-222112_PX777679
A1_OM_2024_HIRes-222545_PX777676
A1_OM_2022_HIRes-229721_PX777411
A1_OM_2024_HIRes-213779_PX777706
A1_OM_2024_HIRes-219814_PX777689
A1_OM_2023_HIRes-227353_PX777518
A1_OM_2024_HIRes-243136_PX777749
A1_OM_2024_HIRes-243138_PX777748
A1_OM_2023_HIRes-217502_PX777558
A1_OM_2022_HIRes-230218_PX777313
A1_OM_2021_HIRes-221942_PX777203
A1_OM_2023_HIRes-224486_PX777524
A1_OM_2017_HIRes-1027886_PX776890
A1_OM_2021_HIRes-221071_PX777211
A1_OM_2020_HIRes-2246039_PX777129
A1_OM_2020_HIRes-2268216_PX777118
A1_OM_2019_HIRes-125130_PX777067
A1_OM_2018_HI006192_PX776912
A1_OM_2018_HI022425_PX776965
A1_OM_2018_HI023822_PX776968
A1_OM_2020_HIRes-27994_PX777161
A1_OM_2022_HIRes-223291_PX777350
A1_OM_2018_HI000844_PX776896
A1_OM_2023_HIRes-216905_PX777560
A1_OM_2021_HIRes-219374_PX777221
A1_OM_2021_HIRes-217507_PX777226
A1_OM_2018_HI007329_PX776915
A1_OM_2020_HIRes-2246036_PX777130
A1_OM_2020_HIRes-22159_PX777182
A1_OM_2019_HIRes-12665_PX776994
A1_OM_2024_HIRes-223781_PX777671
A1_OM_2024_HIRes-226741_PX777652
A1_OM_2018_HI013858_PX776943
A1_OM_2021_HIRes-216033_PX777231
A1_OM_2022_HIRes-228875_PX777412
A1_OM_2023_HIRes-222499_PX777529
A1_OM_2024_HIRes-222113_PX777678
A1_OM_2020_HIRes-2273632_PX777110
A1_OM_2019_HIRes-137909_PX777098
A1_OM_2019_HIRes-19402_PX777013
A1_OM_2022_HIRes-211766_PX777399
A1_OM_2023_HIRes-235988_PX777606
A1_OM_2022_HIRes-222517_PX777358
A1_OM_2022_HIRes-224089_PX777443
A1_OM_2018_HI024563_PX776974
A1_OM_2023_HIRes-239168_PX777598
A1_OM_2021_HIRes-214299_PX777238
A1_OM_2021_HIRes-218076_PX777224
A1_OM_2023_HIRes-214889_PX777572
A1_OM_2022_HIRes-223998_PX777346
A1_OM_2022_HIRes-228386_PX777416
A1_OM_2024_HIRes-226551_PX777655
A1_OM_2020_HIRes-2230170_PX777138
A1_OM_2024_HIRes-211272_PX777718
A1_OM_2018_HI007331_PX776916
A1_OM_2021_HIRes-221604_PX777207
A1_OM_2022_HIRes-217969_PX777380
A1_OM_2022_HIRes-221175_PX777457
A1_OM_2020_HIRes-25409_PX777172
0.9252
0.9927
0.9649
0.9779
0.9151
0.9017

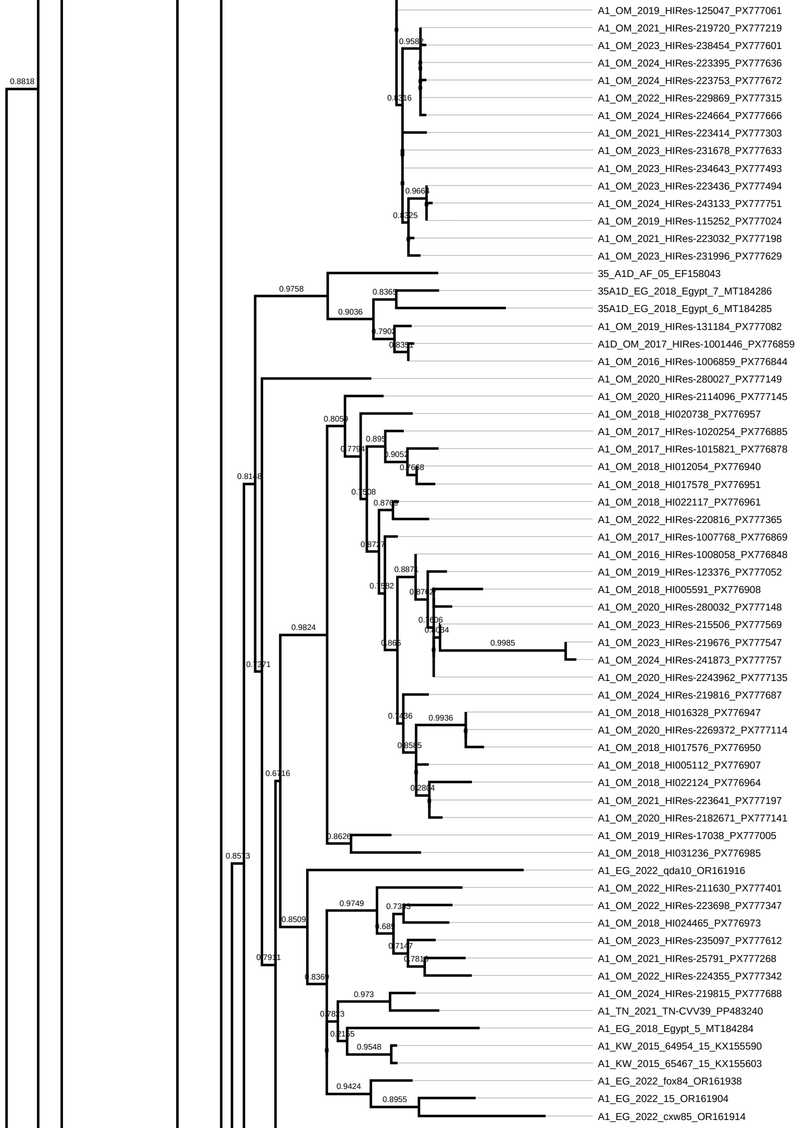
A1_OM_2019_HIRes-125047_PX777061
A1_OM_2021_HIRes-219720_PX777219
A1_OM_2023_HIRes-238454_PX777601
A1_OM_2024_HIRes-223395_PX777636
A1_OM_2024_HIRes-223753_PX777672
A1_OM_2022_HIRes-229869_PX777315
A1_OM_2024_HIRes-224664_PX777666
A1_OM_2021_HIRes-223414_PX777303
A1_OM_2023_HIRes-231678_PX777633
A1_OM_2023_HIRes-234643_PX777493
A1_OM_2023_HIRes-223436_PX777494
A1_OM_2024_HIRes-243133_PX777751
A1_OM_2019_HIRes-115252_PX777024
A1_OM_2021_HIRes-223032_PX777198
A1_OM_2023_HIRes-231996_PX777629
35_A1D_AF_05_EF158043
35A1D_EG_2018_Egypt_7_MT184286
35A1D_EG_2018_Egypt_6_MT184285
A1_OM_2019_HIRes-131184_PX777082
A1D_OM_2017_HIRes-1001446_PX776859
A1_OM_2016_HIRes-1006859_PX776844
A1_OM_2020_HIRes-280027_PX777149
A1_OM_2020_HIRes-2114096_PX777145
A1_OM_2018_HI020738_PX776957
A1_OM_2017_HIRes-1020254_PX776885
A1_OM_2017_HIRes-1015821_PX776878
A1_OM_2018_HI012054_PX776940
A1_OM_2018_HI017578_PX776951
A1_OM_2018_HI022117_PX776961
A1_OM_2022_HIRes-220816_PX777365
A1_OM_2017_HIRes-1007768_PX776869
A1_OM_2016_HIRes-1008058_PX776848
A1_OM_2019_HIRes-123376_PX777052
A1_OM_2018_HI005591_PX776908
A1_OM_2020_HIRes-280032_PX777148
A1_OM_2023_HIRes-215506_PX777569
A1_OM_2023_HIRes-219676_PX777547
A1_OM_2024_HIRes-241873_PX777757
A1_OM_2020_HIRes-2243962_PX777135
A1_OM_2024_HIRes-219816_PX777687
A1_OM_2018_HI016328_PX776947
A1_OM_2020_HIRes-2269372_PX777114
A1_OM_2018_HI017576_PX776950
A1_OM_2018_HI005112_PX776907
A1_OM_2018_HI022124_PX776964
A1_OM_2021_HIRes-223641_PX777197
A1_OM_2020_HIRes-2182671_PX777141
A1_OM_2019_HIRes-17038_PX777005
A1_OM_2018_HI031236_PX776985
A1_EG_2022_qda10_OR161916
A1_OM_2022_HIRes-211630_PX777401
A1_OM_2022_HIRes-223698_PX777347
A1_OM_2018_HI024465_PX776973
A1_OM_2023_HIRes-235097_PX777612
A1_OM_2021_HIRes-25791_PX777268
A1_OM_2022_HIRes-224355_PX777342
A1_OM_2024_HIRes-219815_PX777688
A1_TN_2021_TN-CVV39_PP483240
A1_EG_2018_Egypt_5_MT184284
A1_KW_2015_64954_15_KX155590
A1_KW_2015_65467_15_KX155603
A1_EG_2022_fox84_OR161938
A1_EG_2022_15_OR161904
A1_EG_2022_cxw85_OR161914

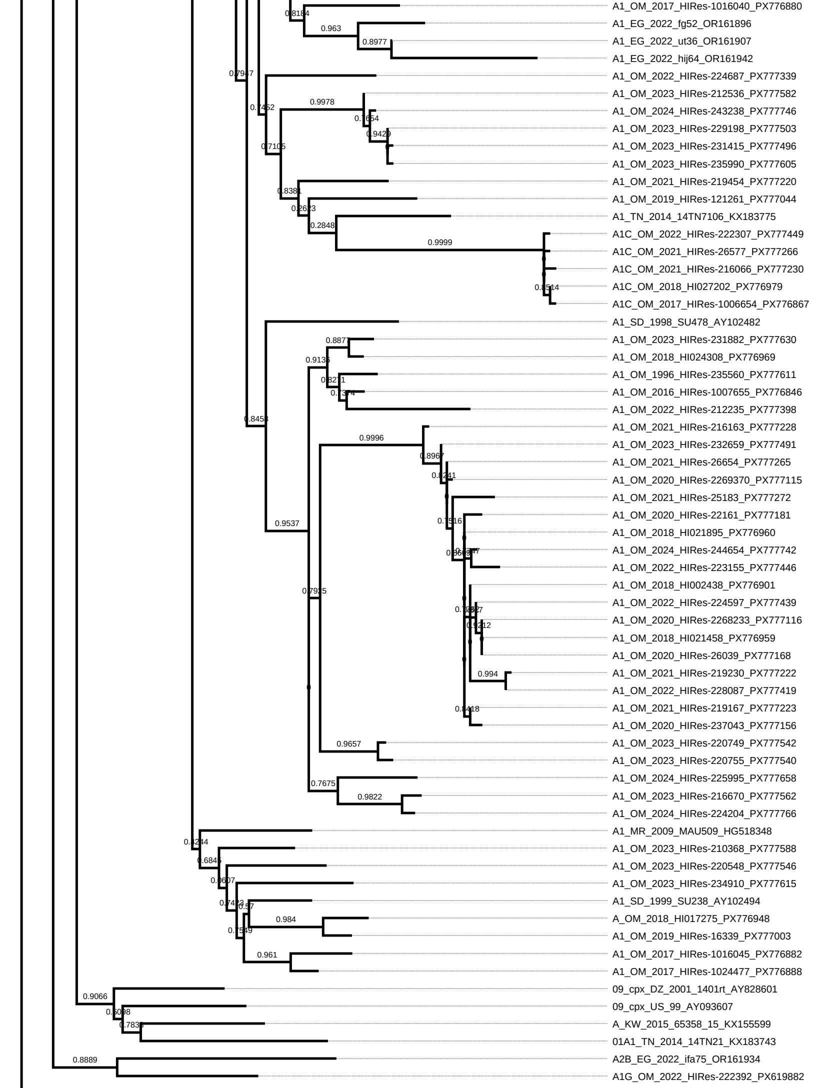
A1_OM_2017_HIRes-1016040_PX776880
A1_EG_2022_fg52_OR161896
A1_EG_2022_ut36_OR161907
A1_EG_2022_hij64_OR161942
A1_OM_2022_HIRes-224687_PX777339
A1_OM_2023_HIRes-212536_PX777582
A1_OM_2024_HIRes-243238_PX777746
A1_OM_2023_HIRes-229198_PX777503
A1_OM_2023_HIRes-231415_PX777496
A1_OM_2023_HIRes-235990_PX777605
A1_OM_2021_HIRes-219454_PX777220
A1_OM_2019_HIRes-121261_PX777044
A1_TN_2014_14TN7106_KX183775
A1C_OM_2022_HIRes-222307_PX777449
A1C_OM_2021_HIRes-26577_PX777266
A1C_OM_2021_HIRes-216066_PX777230
A1C_OM_2018_HI027202_PX776979
A1C_OM_2017_HIRes-1006654_PX776867
A1_SD_1998_SU478_AY102482
A1_OM_2023_HIRes-231882_PX777630
A1_OM_2018_HI024308_PX776969
A1_OM_1996_HIRes-235560_PX777611
A1_OM_2016_HIRes-1007655_PX776846
A1_OM_2022_HIRes-212235_PX777398
A1_OM_2021_HIRes-216163_PX777228
A1_OM_2023_HIRes-232659_PX777491
A1_OM_2021_HIRes-26654_PX777265
A1_OM_2020_HIRes-2269370_PX777115
A1_OM_2021_HIRes-25183_PX777272
A1_OM_2020_HIRes-22161_PX777181
A1_OM_2018_HI021895_PX776960
A1_OM_2024_HIRes-244654_PX777742
A1_OM_2022_HIRes-223155_PX777446
A1_OM_2018_HI002438_PX776901
A1_OM_2022_HIRes-224597_PX777439
A1_OM_2020_HIRes-2268233_PX777116
A1_OM_2018_HI021458_PX776959
A1_OM_2020_HIRes-26039_PX777168
A1_OM_2021_HIRes-219230_PX777222
A1_OM_2022_HIRes-228087_PX777419
A1_OM_2021_HIRes-219167_PX777223
A1_OM_2020_HIRes-237043_PX777156
A1_OM_2023_HIRes-220749_PX777542
A1_OM_2023_HIRes-220755_PX777540
A1_OM_2024_HIRes-225995_PX777658
A1_OM_2023_HIRes-216670_PX777562
A1_OM_2024_HIRes-224204_PX777766
A1_MR_2009_MAU509_HG518348
A1_OM_2023_HIRes-210368_PX777588
A1_OM_2023_HIRes-220548_PX777546
A1_OM_2023_HIRes-234910_PX777615
A1_SD_1999_SU238_AY102494
A_OM_2018_HI017275_PX776948
A1_OM_2019_HIRes-16339_PX777003
A1_OM_2017_HIRes-1016045_PX776882
A1_OM_2017_HIRes-1024477_PX776888
09_cpx_DZ_2001_1401rt_AY828601
09_cpx_US_99_AY093607
A_KW_2015_65358_15_KX155599
01A1_TN_2014_14TN21_KX183743
A2B_EG_2022_ifa75_OR161934
A1G_OM_2022_HIRes-222392_PX619882

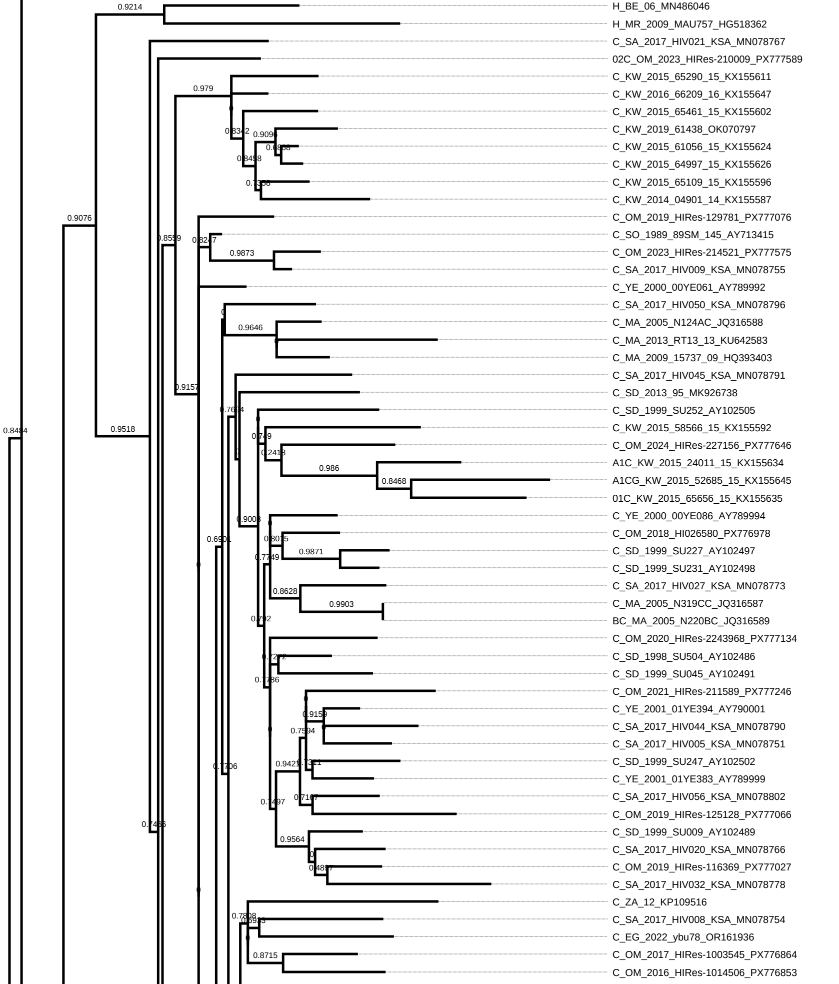
0.9214
H_BE_06_MN486046
H_MR_2009_MAU757_HG518362
C_SA_2017_HIV021_KSA_MN078767
02C_OM_2023_HIRes-210009_PX777589
0.979
C_KW_2015_65290_15_KX155611
C_KW_2016_66209_16_KX155647
C_KW_2015_65461_15_KX155602
0.8342
0.9096
C_KW_2019_61438_OK070797
C_KW_2015_61056_15_KX155624
0.8458
C_KW_2015_64997_15_KX155626
C_KW_2015_65109_15_KX155596
C_KW_2014_04901_14_KX155587
0.9076
C_OM_2019_HIRes-129781_PX777076
0.8559
0.8247
C_SO_1989_89SM_145_AY713415
0.9873
C_OM_2023_HIRes-214521_PX777575
C_SA_2017_HIV009_KSA_MN078755
C_YE_2000_00YE061_AY789992
C_SA_2017_HIV050_KSA_MN078796
0.9646
C_MA_2005_N124AC_JQ316588
C_MA_2013_RT13_13_KU642583
C_MA_2009_15737_09_HQ393403
C_SA_2017_HIV045_KSA_MN078791
0.9157
C_SD_2013_95_MK926738
C_SD_1999_SU252_AY102505
0.8484
0.9518
C_KW_2015_58566_15_KX155592
0.749
C_OM_2024_HIRes-227156_PX777646
0.2418
0.986
A1C_KW_2015_24011_15_KX155634
0.8468
A1CG_KW_2015_52685_15_KX155645
01C_KW_2015_65656_15_KX155635
C_YE_2000_00YE086_AY789994
0.6901
C_OM_2018_HI026580_PX776978
0.9871
C_SD_1999_SU227_AY102497
0.7749
C_SD_1999_SU231_AY102498
C_SA_2017_HIV027_KSA_MN078773
0.8628
0.9903
C_MA_2005_N319CC_JQ316587
BC_MA_2005_N220BC_JQ316589
C_OM_2020_HIRes-2243968_PX777134
C_SD_1998_SU504_AY102486
C_SD_1999_SU045_AY102491
0.7786
C_OM_2021_HIRes-211589_PX777246
0.9159
C_YE_2001_01YE394_AY790001
C_SA_2017_HIV044_KSA_MN078790
0.7594
C_SA_2017_HIV005_KSA_MN078751
0.7706
C_SD_1999_SU247_AY102502
C_YE_2001_01YE383_AY789999
C_SA_2017_HIV056_KSA_MN078802
0.7497
C_OM_2019_HIRes-125128_PX777066
0.7466
0.9564
C_SD_1999_SU009_AY102489
C_SA_2017_HIV020_KSA_MN078766
C_OM_2019_HIRes-116369_PX777027
C_SA_2017_HIV032_KSA_MN078778
C_ZA_12_KP109516
0.7808
C_SA_2017_HIV008_KSA_MN078754
C_EG_2022_ybu78_OR161936
0.8715
C_OM_2017_HIRes-1003545_PX776864
C_OM_2016_HIRes-1014506_PX776853

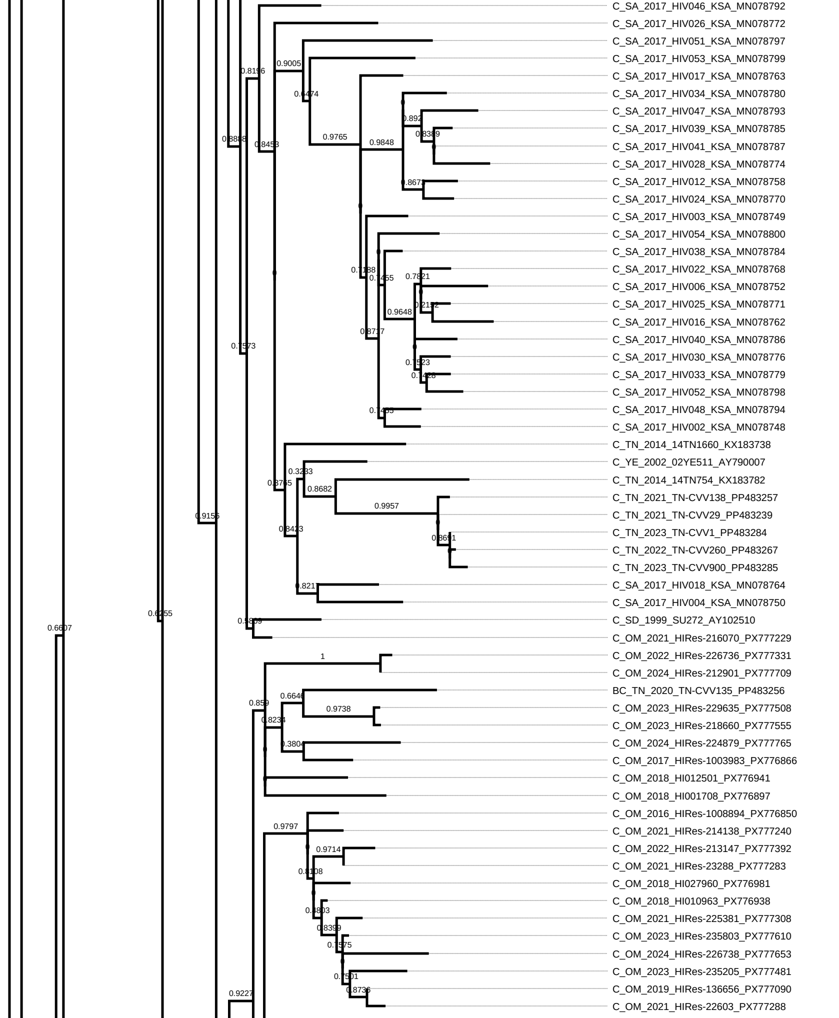
C_SA_2017_HIV046_KSA_MN078792
C_SA_2017_HIV026_KSA_MN078772
C_SA_2017_HIV051_KSA_MN078797
C_SA_2017_HIV053_KSA_MN078799
C_SA_2017_HIV017_KSA_MN078763
C_SA_2017_HIV034_KSA_MN078780
C_SA_2017_HIV047_KSA_MN078793
C_SA_2017_HIV039_KSA_MN078785
C_SA_2017_HIV041_KSA_MN078787
C_SA_2017_HIV028_KSA_MN078774
C_SA_2017_HIV012_KSA_MN078758
C_SA_2017_HIV024_KSA_MN078770
C_SA_2017_HIV003_KSA_MN078749
C_SA_2017_HIV054_KSA_MN078800
C_SA_2017_HIV038_KSA_MN078784
C_SA_2017_HIV022_KSA_MN078768
C_SA_2017_HIV006_KSA_MN078752
C_SA_2017_HIV025_KSA_MN078771
C_SA_2017_HIV016_KSA_MN078762
C_SA_2017_HIV040_KSA_MN078786
C_SA_2017_HIV030_KSA_MN078776
C_SA_2017_HIV033_KSA_MN078779
C_SA_2017_HIV052_KSA_MN078798
C_SA_2017_HIV048_KSA_MN078794
C_SA_2017_HIV002_KSA_MN078748
C_TN_2014_14TN1660_KX183738
C_YE_2002_02YE511_AY790007
C_TN_2014_14TN754_KX183782
C_TN_2021_TN-CVV138_PP483257
C_TN_2021_TN-CVV29_PP483239
C_TN_2023_TN-CVV1_PP483284
C_TN_2022_TN-CVV260_PP483267
C_TN_2023_TN-CVV900_PP483285
C_SA_2017_HIV018_KSA_MN078764
C_SA_2017_HIV004_KSA_MN078750
C_SD_1999_SU272_AY102510
C_OM_2021_HIRes-216070_PX777229
C_OM_2022_HIRes-226736_PX777331
C_OM_2024_HIRes-212901_PX777709
BC_TN_2020_TN-CVV135_PP483256
C_OM_2023_HIRes-229635_PX777508
C_OM_2023_HIRes-218660_PX777555
C_OM_2024_HIRes-224879_PX777765
C_OM_2017_HIRes-1003983_PX776866
C_OM_2018_HI012501_PX776941
C_OM_2018_HI001708_PX776897
C_OM_2016_HIRes-1008894_PX776850
C_OM_2021_HIRes-214138_PX777240
C_OM_2022_HIRes-213147_PX777392
C_OM_2021_HIRes-23288_PX777283
C_OM_2018_HI027960_PX776981
C_OM_2018_HI010963_PX776938
C_OM_2021_HIRes-225381_PX777308
C_OM_2023_HIRes-235803_PX777610
C_OM_2024_HIRes-226738_PX777653
C_OM_2023_HIRes-235205_PX777481
C_OM_2019_HIRes-136656_PX777090
C_OM_2021_HIRes-22603_PX777288
0.8196
0.9005
0.9765
0.9848
0.9648
0.8682
0.9957
0.9156
1
0.9738
0.9797
0.9714
0.9227

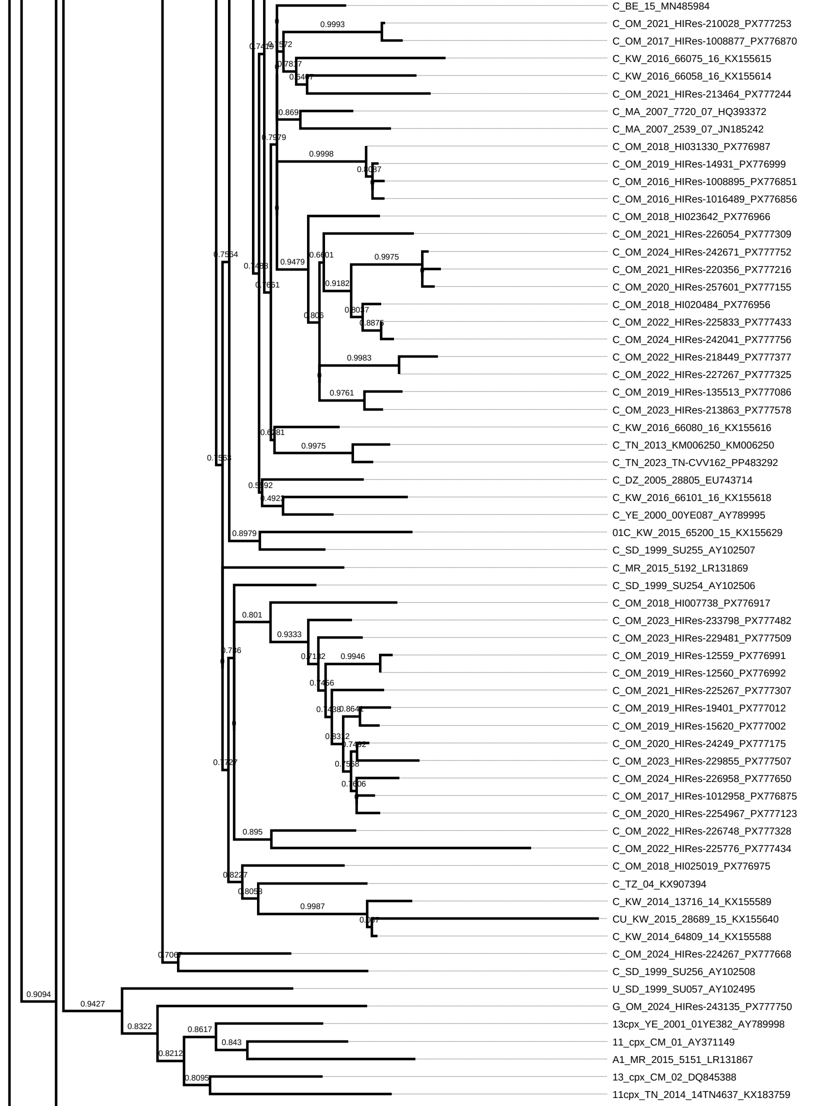

C_BE_15_MN485984
C_OM_2021_HIRes-210028_PX777253
C_OM_2017_HIRes-1008877_PX776870
C_KW_2016_66075_16_KX155615
C_KW_2016_66058_16_KX155614
C_OM_2021_HIRes-213464_PX777244
C_MA_2007_7720_07_HQ393372
C_MA_2007_2539_07_JN185242
C_OM_2018_HI031330_PX776987
C_OM_2019_HIRes-14931_PX776999
C_OM_2016_HIRes-1008895_PX776851
C_OM_2016_HIRes-1016489_PX776856
C_OM_2018_HI023642_PX776966
C_OM_2021_HIRes-226054_PX777309
C_OM_2024_HIRes-242671_PX777752
C_OM_2021_HIRes-220356_PX777216
C_OM_2020_HIRes-257601_PX777155
C_OM_2018_HI020484_PX776956
C_OM_2022_HIRes-225833_PX777433
C_OM_2024_HIRes-242041_PX777756
C_OM_2022_HIRes-218449_PX777377
C_OM_2022_HIRes-227267_PX777325
C_OM_2019_HIRes-135513_PX777086
C_OM_2023_HIRes-213863_PX777578
C_KW_2016_66080_16_KX155616
C_TN_2013_KM006250_KM006250
C_TN_2023_TN-CVV162_PP483292
C_DZ_2005_28805_EU743714
C_KW_2016_66101_16_KX155618
C_YE_2000_00YE087_AY789995
01C_KW_2015_65200_15_KX155629
C_SD_1999_SU255_AY102507
C_MR_2015_5192_LR131869
C_SD_1999_SU254_AY102506
C_OM_2018_HI007738_PX776917
C_OM_2023_HIRes-233798_PX777482
C_OM_2023_HIRes-229481_PX777509
C_OM_2019_HIRes-12559_PX776991
C_OM_2019_HIRes-12560_PX776992
C_OM_2021_HIRes-225267_PX777307
C_OM_2019_HIRes-19401_PX777012
C_OM_2019_HIRes-15620_PX777002
C_OM_2020_HIRes-24249_PX777175
C_OM_2023_HIRes-229855_PX777507
C_OM_2024_HIRes-226958_PX777650
C_OM_2017_HIRes-1012958_PX776875
C_OM_2020_HIRes-2254967_PX777123
C_OM_2022_HIRes-226748_PX777328
C_OM_2022_HIRes-225776_PX777434
C_OM_2018_HI025019_PX776975
C_TZ_04_KX907394
C_KW_2014_13716_14_KX155589
CU_KW_2015_28689_15_KX155640
C_KW_2014_64809_14_KX155588
C_OM_2024_HIRes-224267_PX777668
C_SD_1999_SU256_AY102508
U_SD_1999_SU057_AY102495
G_OM_2024_HIRes-243135_PX777750
13cpx_YE_2001_01YE382_AY789998
11_cpx_CM_01_AY371149
A1_MR_2015_5151_LR131867
13_cpx_CM_02_DQ845388
11cpx_TN_2014_14TN4637_KX183759

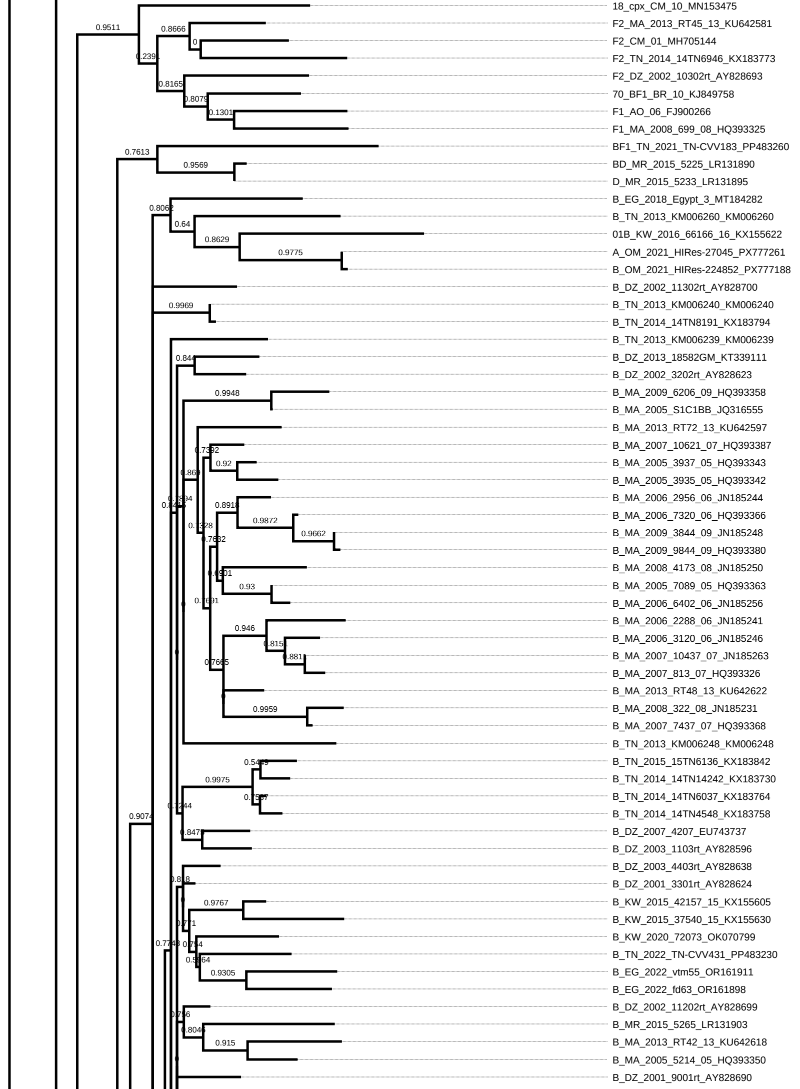
18_cpx_CM_10_MN153475
F2_MA_2013_RT45_13_KU642581
F2_CM_01_MH705144
F2_TN_2014_14TN6946_KX183773
F2_DZ_2002_10302rt_AY828693
70_BF1_BR_10_KJ849758
F1_AO_06_FJ900266
F1_MA_2008_699_08_HQ393325
BF1_TN_2021_TN-CVV183_PP483260
BD_MR_2015_5225_LR131890
D_MR_2015_5233_LR131895
B_EG_2018_Egypt_3_MT184282
B_TN_2013_KM006260_KM006260
01B_KW_2016_66166_16_KX155622
A_OM_2021_HIRes-27045_PX777261
B_OM_2021_HIRes-224852_PX777188
B_DZ_2002_11302rt_AY828700
B_TN_2013_KM006240_KM006240
B_TN_2014_14TN8191_KX183794
B_TN_2013_KM006239_KM006239
B_DZ_2013_18582GM_KT339111
B_DZ_2002_3202rt_AY828623
B_MA_2009_6206_09_HQ393358
B_MA_2005_S1C1BB_JQ316555
B_MA_2013_RT72_13_KU642597
B_MA_2007_10621_07_HQ393387
B_MA_2005_3937_05_HQ393343
B_MA_2005_3935_05_HQ393342
B_MA_2006_2956_06_JN185244
B_MA_2006_7320_06_HQ393366
B_MA_2009_3844_09_JN185248
B_MA_2009_9844_09_HQ393380
B_MA_2008_4173_08_JN185250
B_MA_2005_7089_05_HQ393363
B_MA_2006_6402_06_JN185256
B_MA_2006_2288_06_JN185241
B_MA_2006_3120_06_JN185246
B_MA_2007_10437_07_JN185263
B_MA_2007_813_07_HQ393326
B_MA_2013_RT48_13_KU642622
B_MA_2008_322_08_JN185231
B_MA_2007_7437_07_HQ393368
B_TN_2013_KM006248_KM006248
B_TN_2015_15TN6136_KX183842
B_TN_2014_14TN14242_KX183730
B_TN_2014_14TN6037_KX183764
B_TN_2014_14TN4548_KX183758
B_DZ_2007_4207_EU743737
B_DZ_2003_1103rt_AY828596
B_DZ_2003_4403rt_AY828638
B_DZ_2001_3301rt_AY828624
B_KW_2015_42157_15_KX155605
B_KW_2015_37540_15_KX155630
B_KW_2020_72073_OK070799
B_TN_2022_TN-CVV431_PP483230
B_EG_2022_vtm55_OR161911
B_EG_2022_fd63_OR161898
B_DZ_2002_11202rt_AY828699
B_MR_2015_5265_LR131903
B_MA_2013_RT42_13_KU642618
B_MA_2005_5214_05_HQ393350
B_DZ_2001_9001rt_AY828690

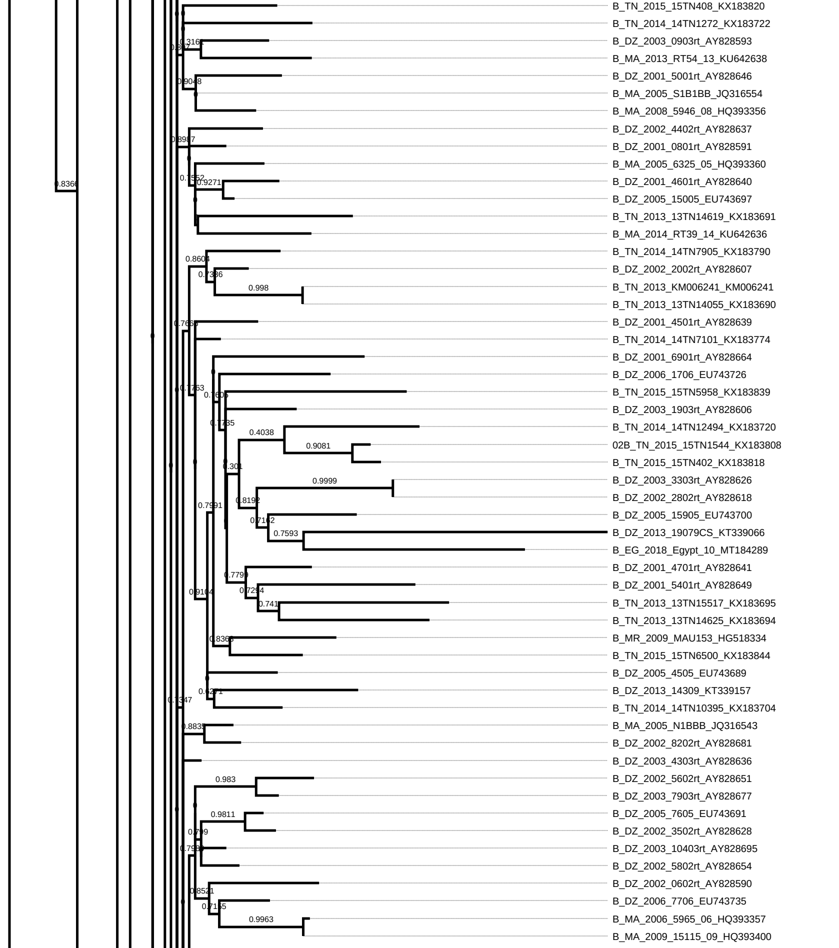
B_TN_2015_15TN408_KX183820
B_TN_2014_14TN1272_KX183722
B_DZ_2003_0903rt_AY828593
B_MA_2013_RT54_13_KU642638
B_DZ_2001_5001rt_AY828646
B_MA_2005_S1B1BB_JQ316554
B_MA_2008_5946_08_HQ393356
B_DZ_2002_4402rt_AY828637
B_DZ_2001_0801rt_AY828591
B_MA_2005_6325_05_HQ393360
B_DZ_2001_4601rt_AY828640
B_DZ_2005_15005_EU743697
B_TN_2013_13TN14619_KX183691
B_MA_2014_RT39_14_KU642636
B_TN_2014_14TN7905_KX183790
B_DZ_2002_2002rt_AY828607
B_TN_2013_KM006241_KM006241
B_TN_2013_13TN14055_KX183690
B_DZ_2001_4501rt_AY828639
B_TN_2014_14TN7101_KX183774
B_DZ_2001_6901rt_AY828664
B_DZ_2006_1706_EU743726
B_TN_2015_15TN5958_KX183839
B_DZ_2003_1903rt_AY828606
B_TN_2014_14TN12494_KX183720
02B_TN_2015_15TN1544_KX183808
B_TN_2015_15TN402_KX183818
B_DZ_2003_3303rt_AY828626
B_DZ_2002_2802rt_AY828618
B_DZ_2005_15905_EU743700
B_DZ_2013_19079CS_KT339066
B_EG_2018_Egypt_10_MT184289
B_DZ_2001_4701rt_AY828641
B_DZ_2001_5401rt_AY828649
B_TN_2013_13TN15517_KX183695
B_TN_2013_13TN14625_KX183694
B_MR_2009_MAU153_HG518334
B_TN_2015_15TN6500_KX183844
B_DZ_2005_4505_EU743689
B_DZ_2013_14309_KT339157
B_TN_2014_14TN10395_KX183704
B_MA_2005_N1BBB_JQ316543
B_DZ_2002_8202rt_AY828681
B_DZ_2003_4303rt_AY828636
B_DZ_2002_5602rt_AY828651
B_DZ_2003_7903rt_AY828677
B_DZ_2005_7605_EU743691
B_DZ_2002_3502rt_AY828628
B_DZ_2003_10403rt_AY828695
B_DZ_2002_5802rt_AY828654
B_DZ_2002_0602rt_AY828590
B_DZ_2006_7706_EU743735
B_MA_2006_5965_06_HQ393357
B_MA_2009_15115_09_HQ393400
0.9271
0.998
0.4038
0.9081
0.9999
0.8192
0.7593
0.741
0.983
0.9811
0.9963

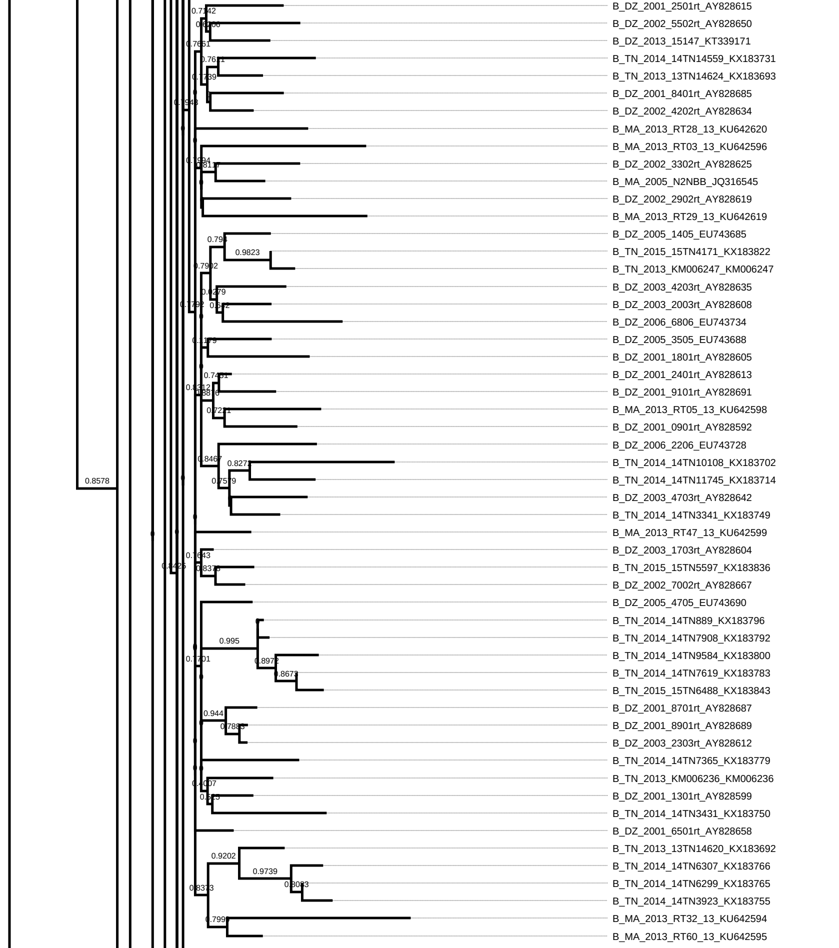
B_DZ_2001_2501rt_AY828615
B_DZ_2002_5502rt_AY828650
B_DZ_2013_15147_KT339171
B_TN_2014_14TN14559_KX183731
B_TN_2013_13TN14624_KX183693
B_DZ_2001_8401rt_AY828685
B_DZ_2002_4202rt_AY828634
B_MA_2013_RT28_13_KU642620
B_MA_2013_RT03_13_KU642596
B_DZ_2002_3302rt_AY828625
B_MA_2005_N2NBB_JQ316545
B_DZ_2002_2902rt_AY828619
B_MA_2013_RT29_13_KU642619
B_DZ_2005_1405_EU743685
B_TN_2015_15TN4171_KX183822
B_TN_2013_KM006247_KM006247
B_DZ_2003_4203rt_AY828635
B_DZ_2003_2003rt_AY828608
B_DZ_2006_6806_EU743734
B_DZ_2005_3505_EU743688
B_DZ_2001_1801rt_AY828605
B_DZ_2001_2401rt_AY828613
B_DZ_2001_9101rt_AY828691
B_MA_2013_RT05_13_KU642598
B_DZ_2001_0901rt_AY828592
B_DZ_2006_2206_EU743728
B_TN_2014_14TN10108_KX183702
B_TN_2014_14TN11745_KX183714
B_DZ_2003_4703rt_AY828642
B_TN_2014_14TN3341_KX183749
B_MA_2013_RT47_13_KU642599
B_DZ_2003_1703rt_AY828604
B_TN_2015_15TN5597_KX183836
B_DZ_2002_7002rt_AY828667
B_DZ_2005_4705_EU743690
B_TN_2014_14TN889_KX183796
B_TN_2014_14TN7908_KX183792
B_TN_2014_14TN9584_KX183800
B_TN_2014_14TN7619_KX183783
B_TN_2015_15TN6488_KX183843
B_DZ_2001_8701rt_AY828687
B_DZ_2001_8901rt_AY828689
B_DZ_2003_2303rt_AY828612
B_TN_2014_14TN7365_KX183779
B_TN_2013_KM006236_KM006236
B_DZ_2001_1301rt_AY828599
B_TN_2014_14TN3431_KX183750
B_DZ_2001_6501rt_AY828658
B_TN_2013_13TN14620_KX183692
B_TN_2014_14TN6307_KX183766
B_TN_2014_14TN6299_KX183765
B_TN_2014_14TN3923_KX183755
B_MA_2013_RT32_13_KU642594
B_MA_2013_RT60_13_KU642595
0.7142
0.7661
0.794
0.9823
0.7902
0.8578
0.8467
0.7579
0.995
0.8972
0.944
0.9202
0.9739
0.8373

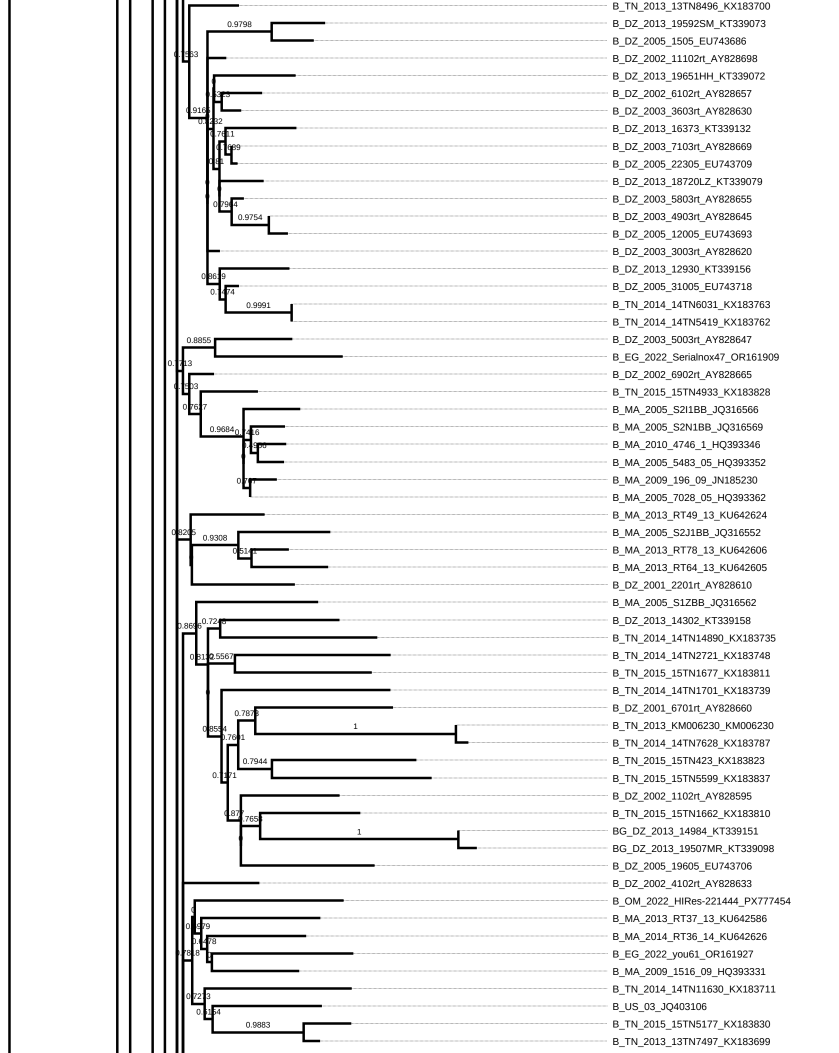

B_TN_2013_13TN8496_KX183700
B_DZ_2013_19592SM_KT339073
B_DZ_2005_1505_EU743686
B_DZ_2002_11102rt_AY828698
B_DZ_2013_19651HH_KT339072
B_DZ_2002_6102rt_AY828657
B_DZ_2003_3603rt_AY828630
B_DZ_2013_16373_KT339132
B_DZ_2003_7103rt_AY828669
B_DZ_2005_22305_EU743709
B_DZ_2013_18720LZ_KT339079
B_DZ_2003_5803rt_AY828655
B_DZ_2003_4903rt_AY828645
B_DZ_2005_12005_EU743693
B_DZ_2003_3003rt_AY828620
B_DZ_2013_12930_KT339156
B_DZ_2005_31005_EU743718
B_TN_2014_14TN6031_KX183763
B_TN_2014_14TN5419_KX183762
B_DZ_2003_5003rt_AY828647
B_EG_2022_Serialnox47_OR161909
B_DZ_2002_6902rt_AY828665
B_TN_2015_15TN4933_KX183828
B_MA_2005_S2I1BB_JQ316566
B_MA_2005_S2N1BB_JQ316569
B_MA_2010_4746_1_HQ393346
B_MA_2005_5483_05_HQ393352
B_MA_2009_196_09_JN185230
B_MA_2005_7028_05_HQ393362
B_MA_2013_RT49_13_KU642624
B_MA_2005_S2J1BB_JQ316552
B_MA_2013_RT78_13_KU642606
B_MA_2013_RT64_13_KU642605
B_DZ_2001_2201rt_AY828610
B_MA_2005_S1ZBB_JQ316562
B_DZ_2013_14302_KT339158
B_TN_2014_14TN14890_KX183735
B_TN_2014_14TN2721_KX183748
B_TN_2015_15TN1677_KX183811
B_TN_2014_14TN1701_KX183739
B_DZ_2001_6701rt_AY828660
B_TN_2013_KM006230_KM006230
B_TN_2014_14TN7628_KX183787
B_TN_2015_15TN423_KX183823
B_TN_2015_15TN5599_KX183837
B_DZ_2002_1102rt_AY828595
B_TN_2015_15TN1662_KX183810
BG_DZ_2013_14984_KT339151
BG_DZ_2013_19507MR_KT339098
B_DZ_2005_19605_EU743706
B_DZ_2002_4102rt_AY828633
B_OM_2022_HIRes-221444_PX777454
B_MA_2013_RT37_13_KU642586
B_MA_2014_RT36_14_KU642626
B_EG_2022_you61_OR161927
B_MA_2009_1516_09_HQ393331
B_TN_2014_14TN11630_KX183711
B_US_03_JQ403106
B_TN_2015_15TN5177_KX183830
B_TN_2013_13TN7497_KX183699

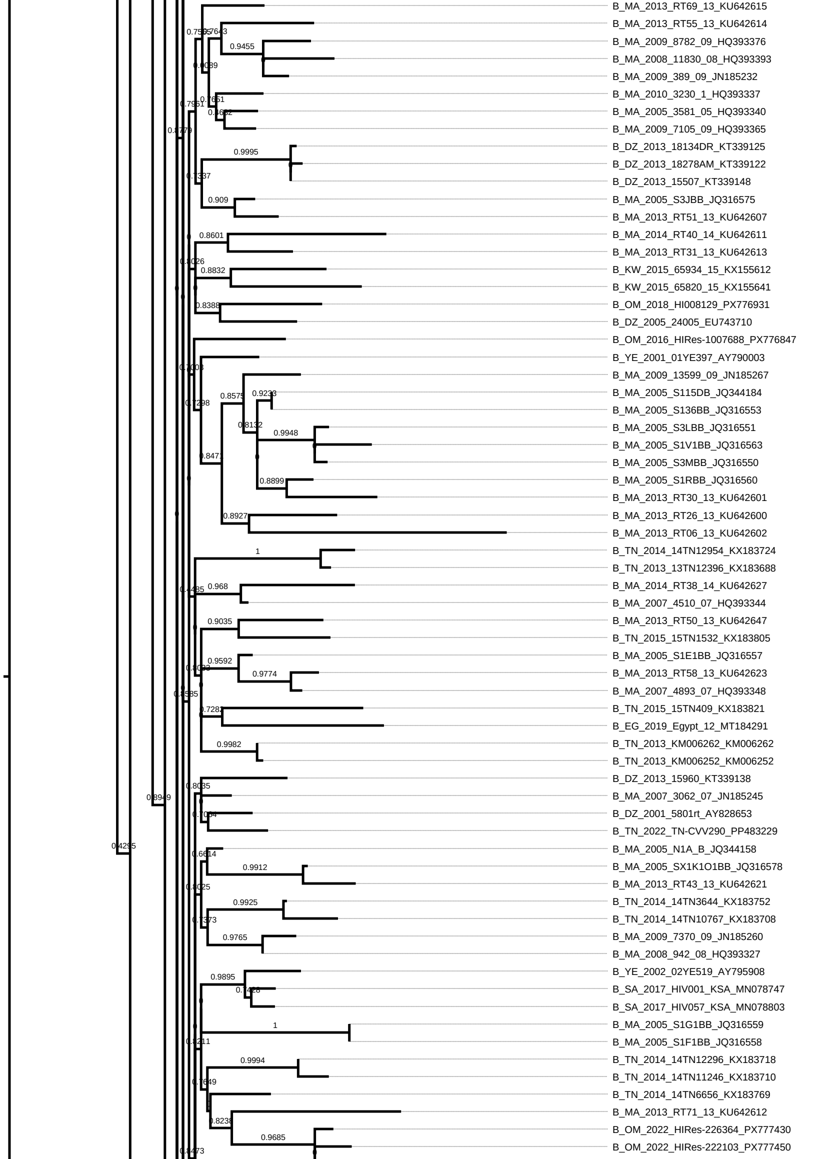
B_MA_2013_RT69_13_KU642615
B_MA_2013_RT55_13_KU642614
B_MA_2009_8782_09_HQ393376
B_MA_2008_11830_08_HQ393393
B_MA_2009_389_09_JN185232
B_MA_2010_3230_1_HQ393337
B_MA_2005_3581_05_HQ393340
B_MA_2009_7105_09_HQ393365
B_DZ_2013_18134DR_KT339125
B_DZ_2013_18278AM_KT339122
B_DZ_2013_15507_KT339148
B_MA_2005_S3JBB_JQ316575
B_MA_2013_RT51_13_KU642607
B_MA_2014_RT40_14_KU642611
B_MA_2013_RT31_13_KU642613
B_KW_2015_65934_15_KX155612
B_KW_2015_65820_15_KX155641
B_OM_2018_HI008129_PX776931
B_DZ_2005_24005_EU743710
B_OM_2016_HIRes-1007688_PX776847
B_YE_2001_01YE397_AY790003
B_MA_2009_13599_09_JN185267
B_MA_2005_S115DB_JQ344184
B_MA_2005_S136BB_JQ316553
B_MA_2005_S3LBB_JQ316551
B_MA_2005_S1V1BB_JQ316563
B_MA_2005_S3MBB_JQ316550
B_MA_2005_S1RBB_JQ316560
B_MA_2013_RT30_13_KU642601
B_MA_2013_RT26_13_KU642600
B_MA_2013_RT06_13_KU642602
B_TN_2014_14TN12954_KX183724
B_TN_2013_13TN12396_KX183688
B_MA_2014_RT38_14_KU642627
B_MA_2007_4510_07_HQ393344
B_MA_2013_RT50_13_KU642647
B_TN_2015_15TN1532_KX183805
B_MA_2005_S1E1BB_JQ316557
B_MA_2013_RT58_13_KU642623
B_MA_2007_4893_07_HQ393348
B_TN_2015_15TN409_KX183821
B_EG_2019_Egypt_12_MT184291
B_TN_2013_KM006262_KM006262
B_TN_2013_KM006252_KM006252
B_DZ_2013_15960_KT339138
B_MA_2007_3062_07_JN185245
B_DZ_2001_5801rt_AY828653
B_TN_2022_TN-CVV290_PP483229
B_MA_2005_N1A_B_JQ344158
B_MA_2005_SX1K1O1BB_JQ316578
B_MA_2013_RT43_13_KU642621
B_TN_2014_14TN3644_KX183752
B_TN_2014_14TN10767_KX183708
B_MA_2009_7370_09_JN185260
B_MA_2008_942_08_HQ393327
B_YE_2002_02YE519_AY795908
B_SA_2017_HIV001_KSA_MN078747
B_SA_2017_HIV057_KSA_MN078803
B_MA_2005_S1G1BB_JQ316559
B_MA_2005_S1F1BB_JQ316558
B_TN_2014_14TN12296_KX183718
B_TN_2014_14TN11246_KX183710
B_TN_2014_14TN6656_KX183769
B_MA_2013_RT71_13_KU642612
B_OM_2022_HIRes-226364_PX777430
B_OM_2022_HIRes-222103_PX777450
0.9455
0.9995
0.909
0.8601
0.8832
0.8388
0.8575
0.9948
0.8899
0.8927
1
0.968
0.9035
0.9592
0.9774
0.9982
0.8949
0.9912
0.9925
0.9765
0.9895
1
0.9994
0.9685

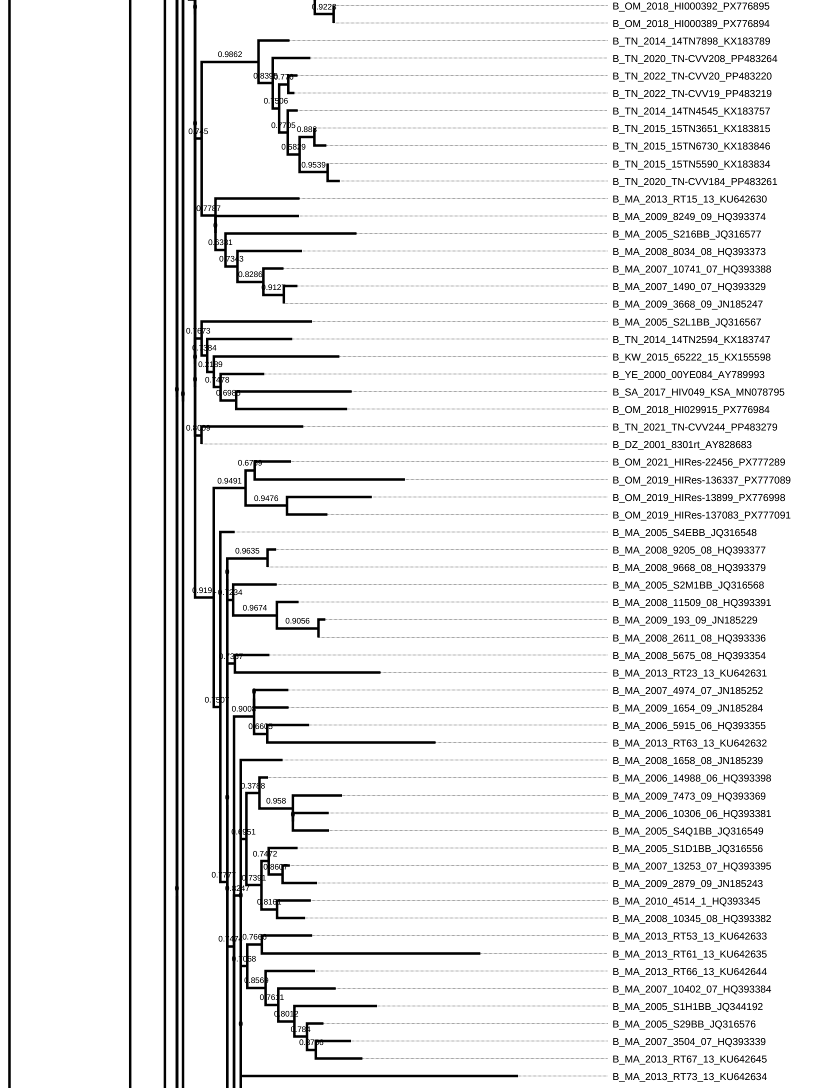
B_OM_2018_HI000392_PX776895
B_OM_2018_HI000389_PX776894
B_TN_2014_14TN7898_KX183789
B_TN_2020_TN-CVV208_PP483264
B_TN_2022_TN-CVV20_PP483220
B_TN_2022_TN-CVV19_PP483219
B_TN_2014_14TN4545_KX183757
B_TN_2015_15TN3651_KX183815
B_TN_2015_15TN6730_KX183846
B_TN_2015_15TN5590_KX183834
B_TN_2020_TN-CVV184_PP483261
B_MA_2013_RT15_13_KU642630
B_MA_2009_8249_09_HQ393374
B_MA_2005_S216BB_JQ316577
B_MA_2008_8034_08_HQ393373
B_MA_2007_10741_07_HQ393388
B_MA_2007_1490_07_HQ393329
B_MA_2009_3668_09_JN185247
B_MA_2005_S2L1BB_JQ316567
B_TN_2014_14TN2594_KX183747
B_KW_2015_65222_15_KX155598
B_YE_2000_00YE084_AY789993
B_SA_2017_HIV049_KSA_MN078795
B_OM_2018_HI029915_PX776984
B_TN_2021_TN-CVV244_PP483279
B_DZ_2001_8301rt_AY828683
B_OM_2021_HIRes-22456_PX777289
B_OM_2019_HIRes-136337_PX777089
B_OM_2019_HIRes-13899_PX776998
B_OM_2019_HIRes-137083_PX777091
B_MA_2005_S4EBB_JQ316548
B_MA_2008_9205_08_HQ393377
B_MA_2008_9668_08_HQ393379
B_MA_2005_S2M1BB_JQ316568
B_MA_2008_11509_08_HQ393391
B_MA_2009_193_09_JN185229
B_MA_2008_2611_08_HQ393336
B_MA_2008_5675_08_HQ393354
B_MA_2013_RT23_13_KU642631
B_MA_2007_4974_07_JN185252
B_MA_2009_1654_09_JN185284
B_MA_2006_5915_06_HQ393355
B_MA_2013_RT63_13_KU642632
B_MA_2008_1658_08_JN185239
B_MA_2006_14988_06_HQ393398
B_MA_2009_7473_09_HQ393369
B_MA_2006_10306_06_HQ393381
B_MA_2005_S4Q1BB_JQ316549
B_MA_2005_S1D1BB_JQ316556
B_MA_2007_13253_07_HQ393395
B_MA_2009_2879_09_JN185243
B_MA_2010_4514_1_HQ393345
B_MA_2008_10345_08_HQ393382
B_MA_2013_RT53_13_KU642633
B_MA_2013_RT61_13_KU642635
B_MA_2013_RT66_13_KU642644
B_MA_2007_10402_07_HQ393384
B_MA_2005_S1H1BB_JQ344192
B_MA_2005_S29BB_JQ316576
B_MA_2007_3504_07_HQ393339
B_MA_2013_RT67_13_KU642645
B_MA_2013_RT73_13_KU642634
0.9862
0.9539
0.9491
0.9476
0.9635
0.9674
0.9056
0.958

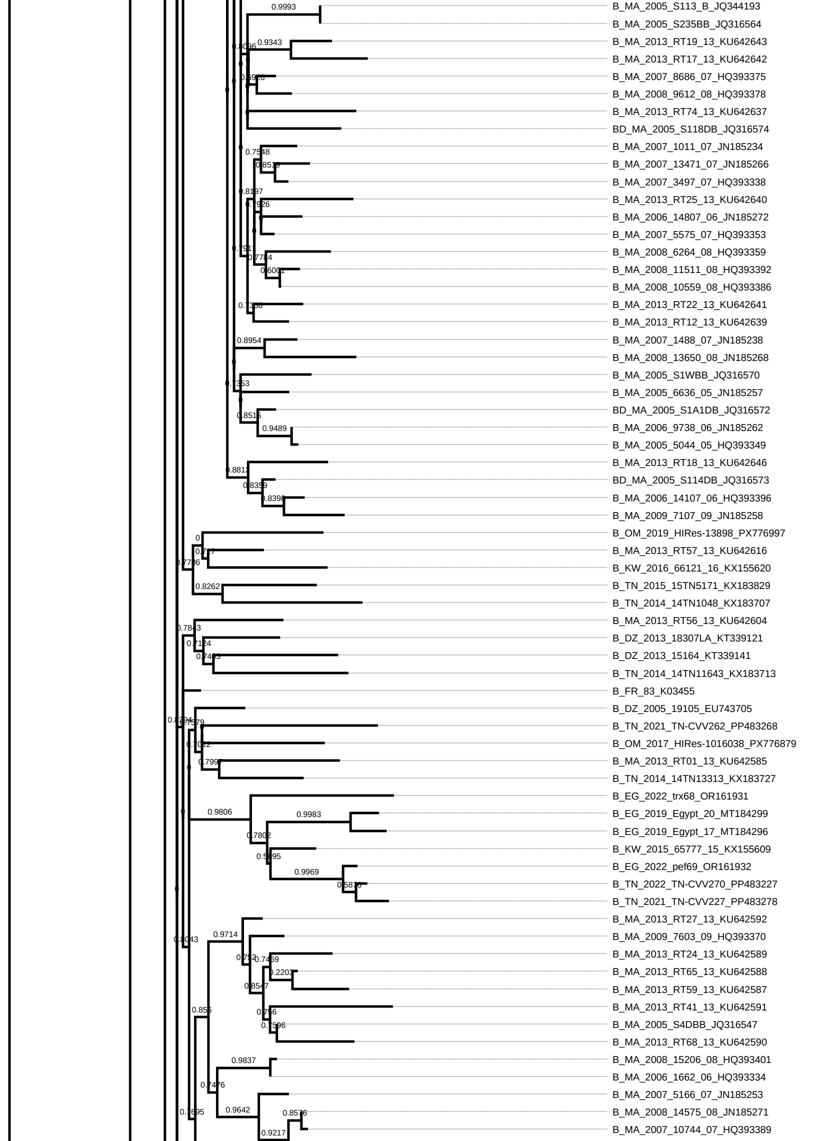
B_MA_2005_S113_B_JQ344193
B_MA_2005_S235BB_JQ316564
B_MA_2013_RT19_13_KU642643
B_MA_2013_RT17_13_KU642642
B_MA_2007_8686_07_HQ393375
B_MA_2008_9612_08_HQ393378
B_MA_2013_RT74_13_KU642637
BD_MA_2005_S118DB_JQ316574
B_MA_2007_1011_07_JN185234
B_MA_2007_13471_07_JN185266
B_MA_2007_3497_07_HQ393338
B_MA_2013_RT25_13_KU642640
B_MA_2006_14807_06_JN185272
B_MA_2007_5575_07_HQ393353
B_MA_2008_6264_08_HQ393359
B_MA_2008_11511_08_HQ393392
B_MA_2008_10559_08_HQ393386
B_MA_2013_RT22_13_KU642641
B_MA_2013_RT12_13_KU642639
B_MA_2007_1488_07_JN185238
B_MA_2008_13650_08_JN185268
B_MA_2005_S1WBB_JQ316570
B_MA_2005_6636_05_JN185257
BD_MA_2005_S1A1DB_JQ316572
B_MA_2006_9738_06_JN185262
B_MA_2005_5044_05_HQ393349
B_MA_2013_RT18_13_KU642646
BD_MA_2005_S114DB_JQ316573
B_MA_2006_14107_06_HQ393396
B_MA_2009_7107_09_JN185258
B_OM_2019_HIRes-13898_PX776997
B_MA_2013_RT57_13_KU642616
B_KW_2016_66121_16_KX155620
B_TN_2015_15TN5171_KX183829
B_TN_2014_14TN1048_KX183707
B_MA_2013_RT56_13_KU642604
B_DZ_2013_18307LA_KT339121
B_DZ_2013_15164_KT339141
B_TN_2014_14TN11643_KX183713
B_FR_83_K03455
B_DZ_2005_19105_EU743705
B_TN_2021_TN-CVV262_PP483268
B_OM_2017_HIRes-1016038_PX776879
B_MA_2013_RT01_13_KU642585
B_TN_2014_14TN13313_KX183727
B_EG_2022_trx68_OR161931
B_EG_2019_Egypt_20_MT184299
B_EG_2019_Egypt_17_MT184296
B_KW_2015_65777_15_KX155609
B_EG_2022_pef69_OR161932
B_TN_2022_TN-CVV270_PP483227
B_TN_2021_TN-CVV227_PP483278
B_MA_2013_RT27_13_KU642592
B_MA_2009_7603_09_HQ393370
B_MA_2013_RT24_13_KU642589
B_MA_2013_RT65_13_KU642588
B_MA_2013_RT59_13_KU642587
B_MA_2013_RT41_13_KU642591
B_MA_2005_S4DBB_JQ316547
B_MA_2013_RT68_13_KU642590
B_MA_2008_15206_08_HQ393401
B_MA_2006_1662_06_HQ393334
B_MA_2007_5166_07_JN185253
B_MA_2008_14575_08_JN185271
B_MA_2007_10744_07_HQ393389

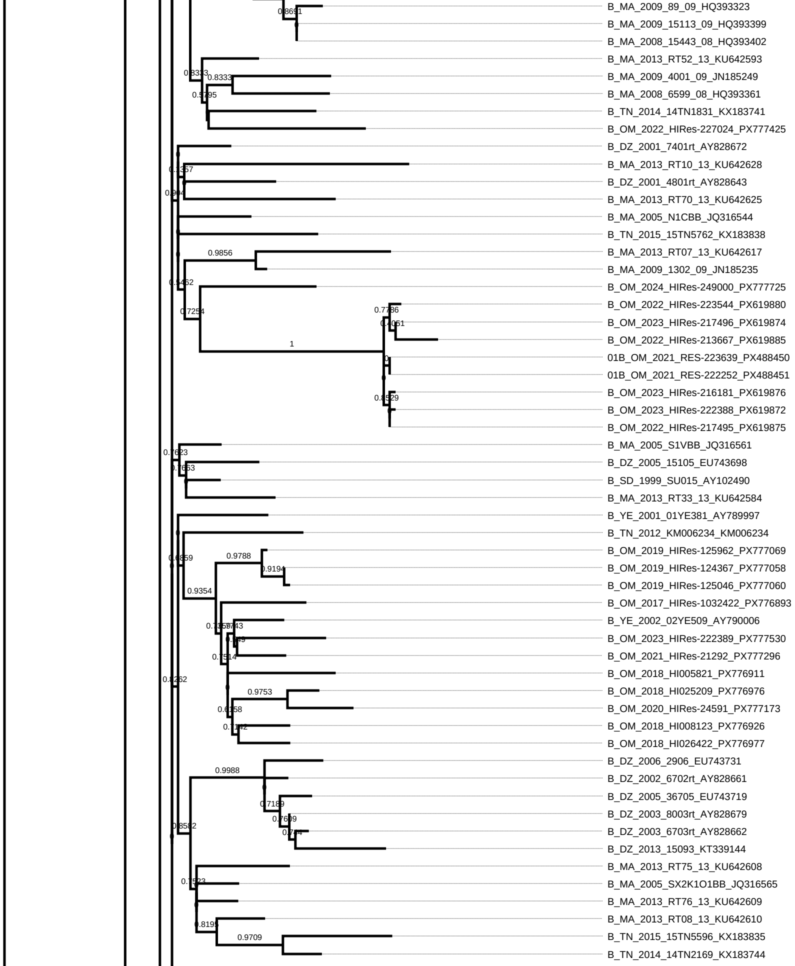
B_MA_2009_89_09_HQ393323
B_MA_2009_15113_09_HQ393399
B_MA_2008_15443_08_HQ393402
B_MA_2013_RT52_13_KU642593
B_MA_2009_4001_09_JN185249
B_MA_2008_6599_08_HQ393361
B_TN_2014_14TN1831_KX183741
B_OM_2022_HIRes-227024_PX777425
B_DZ_2001_7401rt_AY828672
B_MA_2013_RT10_13_KU642628
B_DZ_2001_4801rt_AY828643
B_MA_2013_RT70_13_KU642625
B_MA_2005_N1CBB_JQ316544
B_TN_2015_15TN5762_KX183838
B_MA_2013_RT07_13_KU642617
B_MA_2009_1302_09_JN185235
B_OM_2024_HIRes-249000_PX777725
B_OM_2022_HIRes-223544_PX619880
B_OM_2023_HIRes-217496_PX619874
B_OM_2022_HIRes-213667_PX619885
01B_OM_2021_RES-223639_PX488450
01B_OM_2021_RES-222252_PX488451
B_OM_2023_HIRes-216181_PX619876
B_OM_2023_HIRes-222388_PX619872
B_OM_2022_HIRes-217495_PX619875
B_MA_2005_S1VBB_JQ316561
B_DZ_2005_15105_EU743698
B_SD_1999_SU015_AY102490
B_MA_2013_RT33_13_KU642584
B_YE_2001_01YE381_AY789997
B_TN_2012_KM006234_KM006234
B_OM_2019_HIRes-125962_PX777069
B_OM_2019_HIRes-124367_PX777058
B_OM_2019_HIRes-125046_PX777060
B_OM_2017_HIRes-1032422_PX776893
B_YE_2002_02YE509_AY790006
B_OM_2023_HIRes-222389_PX777530
B_OM_2021_HIRes-21292_PX777296
B_OM_2018_HI005821_PX776911
B_OM_2018_HI025209_PX776976
B_OM_2020_HIRes-24591_PX777173
B_OM_2018_HI008123_PX776926
B_OM_2018_HI026422_PX776977
B_DZ_2006_2906_EU743731
B_DZ_2002_6702rt_AY828661
B_DZ_2005_36705_EU743719
B_DZ_2003_8003rt_AY828679
B_DZ_2003_6703rt_AY828662
B_DZ_2013_15093_KT339144
B_MA_2013_RT75_13_KU642608
B_MA_2005_SX2K1O1BB_JQ316565
B_MA_2013_RT76_13_KU642609
B_MA_2013_RT08_13_KU642610
B_TN_2015_15TN5596_KX183835
B_TN_2014_14TN2169_KX183744
0.8333
0.9856
0.7254
1
0.7786
0.9788
0.9354
0.9753
0.9988
0.7189
0.9709

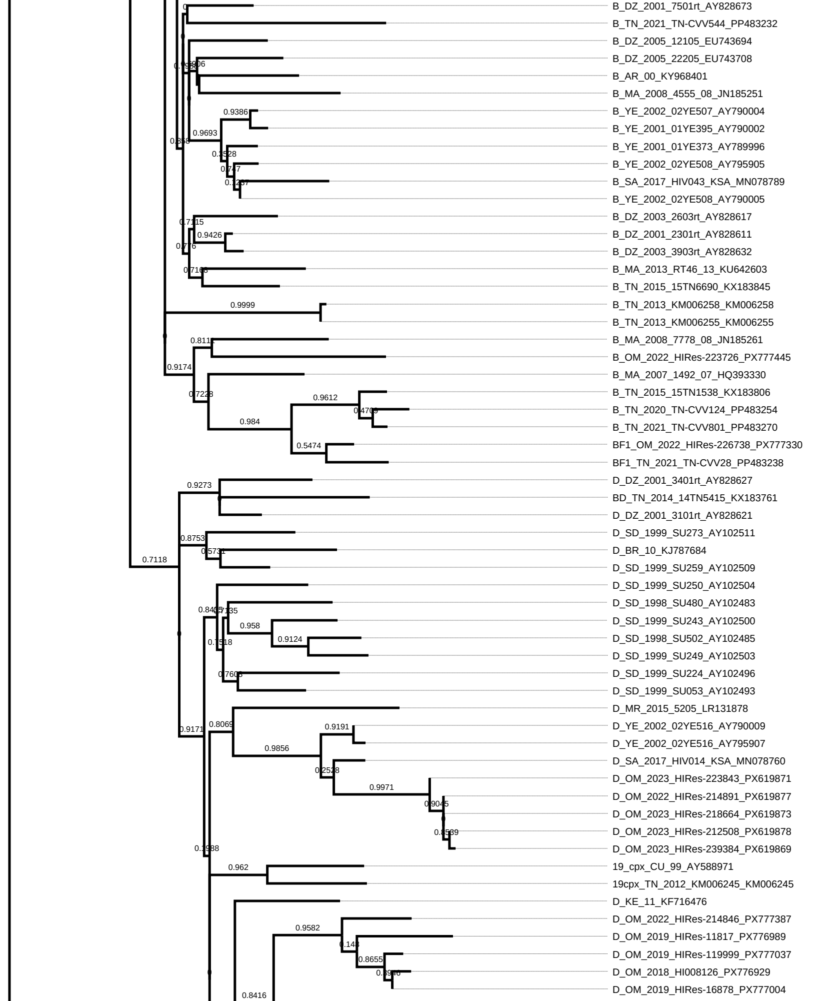

B_DZ_2001_7501rt_AY828673
B_TN_2021_TN-CVV544_PP483232
B_DZ_2005_12105_EU743694
B_DZ_2005_22205_EU743708
B_AR_00_KY968401
B_MA_2008_4555_08_JN185251
B_YE_2002_02YE507_AY790004
B_YE_2001_01YE395_AY790002
B_YE_2001_01YE373_AY789996
B_YE_2002_02YE508_AY795905
B_SA_2017_HIV043_KSA_MN078789
B_YE_2002_02YE508_AY790005
B_DZ_2003_2603rt_AY828617
B_DZ_2001_2301rt_AY828611
B_DZ_2003_3903rt_AY828632
B_MA_2013_RT46_13_KU642603
B_TN_2015_15TN6690_KX183845
B_TN_2013_KM006258_KM006258
B_TN_2013_KM006255_KM006255
B_MA_2008_7778_08_JN185261
B_OM_2022_HIRes-223726_PX777445
B_MA_2007_1492_07_HQ393330
B_TN_2015_15TN1538_KX183806
B_TN_2020_TN-CVV124_PP483254
B_TN_2021_TN-CVV801_PP483270
BF1_OM_2022_HIRes-226738_PX777330
BF1_TN_2021_TN-CVV28_PP483238
D_DZ_2001_3401rt_AY828627
BD_TN_2014_14TN5415_KX183761
D_DZ_2001_3101rt_AY828621
D_SD_1999_SU273_AY102511
D_BR_10_KJ787684
D_SD_1999_SU259_AY102509
D_SD_1999_SU250_AY102504
D_SD_1998_SU480_AY102483
D_SD_1999_SU243_AY102500
D_SD_1998_SU502_AY102485
D_SD_1999_SU249_AY102503
D_SD_1999_SU224_AY102496
D_SD_1999_SU053_AY102493
D_MR_2015_5205_LR131878
D_YE_2002_02YE516_AY790009
D_YE_2002_02YE516_AY795907
D_SA_2017_HIV014_KSA_MN078760
D_OM_2023_HIRes-223843_PX619871
D_OM_2022_HIRes-214891_PX619877
D_OM_2023_HIRes-218664_PX619873
D_OM_2023_HIRes-212508_PX619878
D_OM_2023_HIRes-239384_PX619869
19_cpx_CU_99_AY588971
19cpx_TN_2012_KM006245_KM006245
D_KE_11_KF716476
D_OM_2022_HIRes-214846_PX777387
D_OM_2019_HIRes-11817_PX776989
D_OM_2019_HIRes-119999_PX777037
D_OM_2018_HI008126_PX776929
D_OM_2019_HIRes-16878_PX777004
0.9386
0.9693
0.9426
0.9999
0.9174
0.9612
0.984
0.5474
0.9273
0.8753
0.7118
0.958
0.9124
0.9171
0.8069
0.9191
0.9856
0.9971
0.962
0.9582
0.8655
0.8416

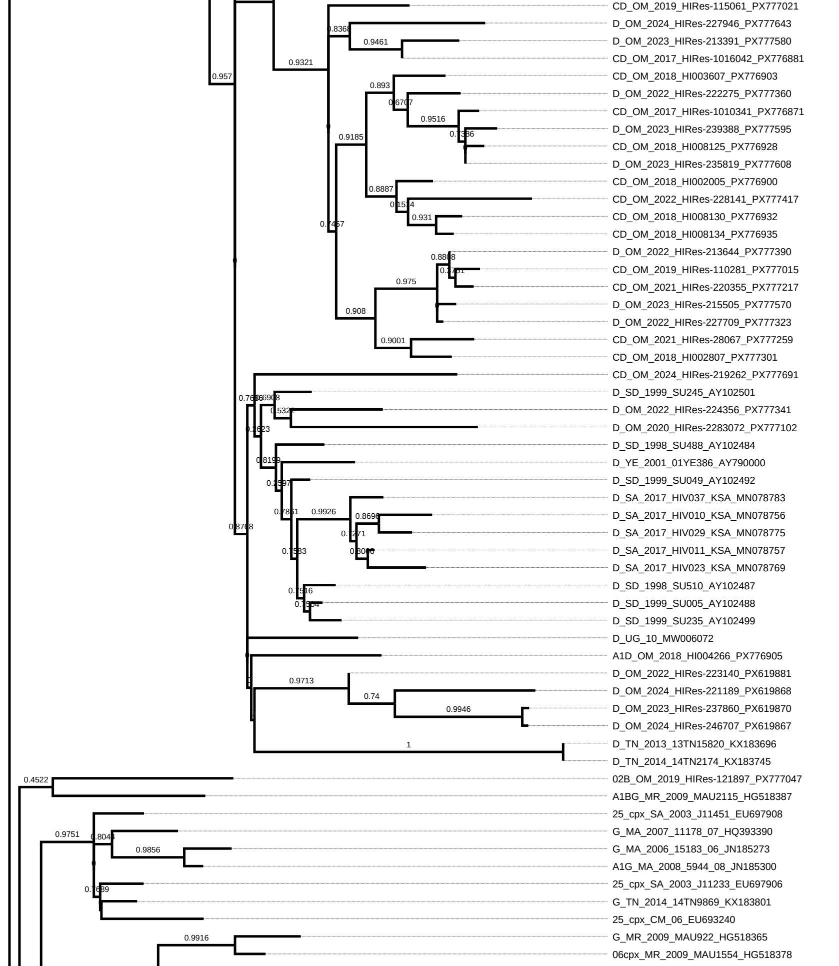

CD_OM_2019_HIRes-115061_PX777021
D_OM_2024_HIRes-227946_PX777643
D_OM_2023_HIRes-213391_PX777580
CD_OM_2017_HIRes-1016042_PX776881
CD_OM_2018_HI003607_PX776903
D_OM_2022_HIRes-222275_PX777360
CD_OM_2017_HIRes-1010341_PX776871
D_OM_2023_HIRes-239388_PX777595
CD_OM_2018_HI008125_PX776928
D_OM_2023_HIRes-235819_PX777608
CD_OM_2018_HI002005_PX776900
CD_OM_2022_HIRes-228141_PX777417
CD_OM_2018_HI008130_PX776932
CD_OM_2018_HI008134_PX776935
D_OM_2022_HIRes-213644_PX777390
CD_OM_2019_HIRes-110281_PX777015
CD_OM_2021_HIRes-220355_PX777217
D_OM_2023_HIRes-215505_PX777570
D_OM_2022_HIRes-227709_PX777323
CD_OM_2021_HIRes-28067_PX777259
CD_OM_2018_HI002807_PX777301
CD_OM_2024_HIRes-219262_PX777691
D_SD_1999_SU245_AY102501
D_OM_2022_HIRes-224356_PX777341
D_OM_2020_HIRes-2283072_PX777102
D_SD_1998_SU488_AY102484
D_YE_2001_01YE386_AY790000
D_SD_1999_SU049_AY102492
D_SA_2017_HIV037_KSA_MN078783
D_SA_2017_HIV010_KSA_MN078756
D_SA_2017_HIV029_KSA_MN078775
D_SA_2017_HIV011_KSA_MN078757
D_SA_2017_HIV023_KSA_MN078769
D_SD_1998_SU510_AY102487
D_SD_1999_SU005_AY102488
D_SD_1999_SU235_AY102499
D_UG_10_MW006072
A1D_OM_2018_HI004266_PX776905
D_OM_2022_HIRes-223140_PX619881
D_OM_2024_HIRes-221189_PX619868
D_OM_2023_HIRes-237860_PX619870
D_OM_2024_HIRes-246707_PX619867
D_TN_2013_13TN15820_KX183696
D_TN_2014_14TN2174_KX183745
02B_OM_2019_HIRes-121897_PX777047
A1BG_MR_2009_MAU2115_HG518387
25_cpx_SA_2003_J11451_EU697908
G_MA_2007_11178_07_HQ393390
G_MA_2006_15183_06_JN185273
A1G_MA_2008_5944_08_JN185300
25_cpx_SA_2003_J11233_EU697906
G_TN_2014_14TN9869_KX183801
25_cpx_CM_06_EU693240
G_MR_2009_MAU922_HG518365
06cpx_MR_2009_MAU1554_HG518378
0.957
0.9321
0.8368
0.9461
0.893
0.6707
0.9516
0.9185
0.8887
0.931
0.7457
0.8808
0.975
0.908
0.9001
0.8199
0.7861
0.9926
0.8696
0.7516
0.8768
0.9713
0.74
0.9946
1
0.4522
0.9751
0.8044
0.9856
0.7689
0.9916

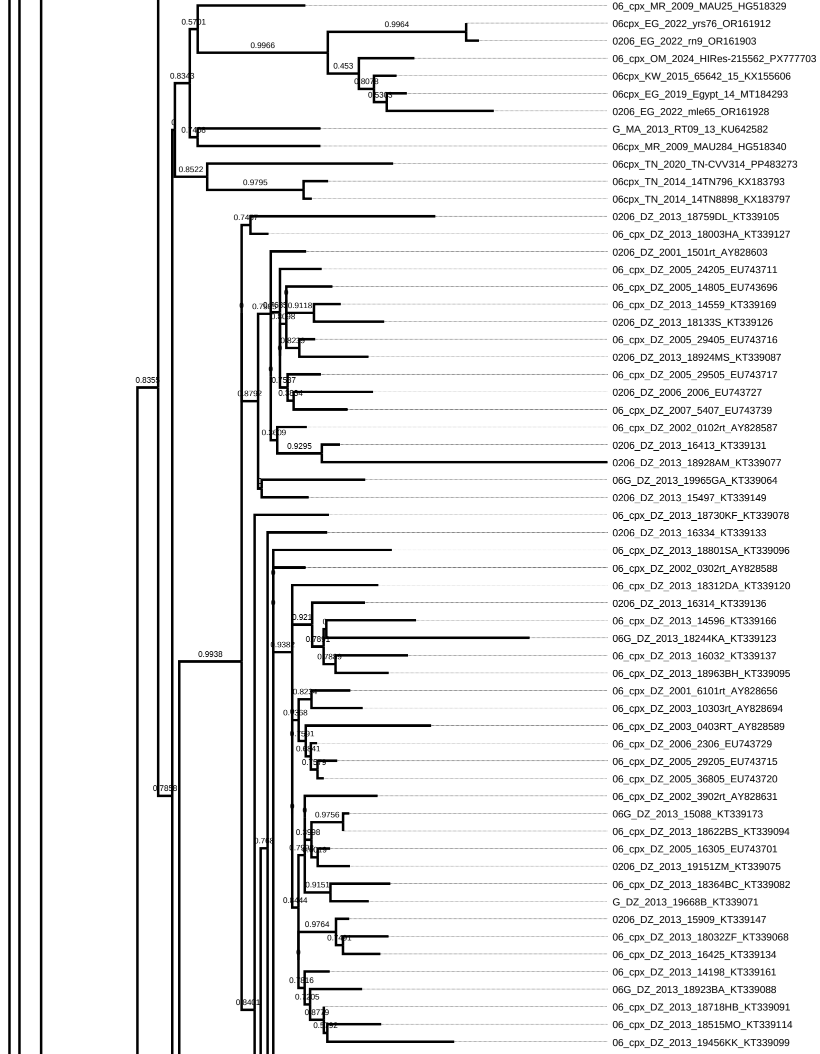

06_cpx_MR_2009_MAU25_HG518329
06cpx_EG_2022_yrs76_OR161912
0206_EG_2022_rn9_OR161903
06_cpx_OM_2024_HIRes-215562_PX777703
06cpx_KW_2015_65642_15_KX155606
06cpx_EG_2019_Egypt_14_MT184293
0206_EG_2022_mle65_OR161928
G_MA_2013_RT09_13_KU642582
06cpx_MR_2009_MAU284_HG518340
06cpx_TN_2020_TN-CVV314_PP483273
06cpx_TN_2014_14TN796_KX183793
06cpx_TN_2014_14TN8898_KX183797
0206_DZ_2013_18759DL_KT339105
06_cpx_DZ_2013_18003HA_KT339127
0206_DZ_2001_1501rt_AY828603
06_cpx_DZ_2005_24205_EU743711
06_cpx_DZ_2005_14805_EU743696
06_cpx_DZ_2013_14559_KT339169
0206_DZ_2013_18133S_KT339126
06_cpx_DZ_2005_29405_EU743716
0206_DZ_2013_18924MS_KT339087
06_cpx_DZ_2005_29505_EU743717
0206_DZ_2006_2006_EU743727
06_cpx_DZ_2007_5407_EU743739
06_cpx_DZ_2002_0102rt_AY828587
0206_DZ_2013_16413_KT339131
0206_DZ_2013_18928AM_KT339077
06G_DZ_2013_19965GA_KT339064
0206_DZ_2013_15497_KT339149
06_cpx_DZ_2013_18730KF_KT339078
0206_DZ_2013_16334_KT339133
06_cpx_DZ_2013_18801SA_KT339096
06_cpx_DZ_2002_0302rt_AY828588
06_cpx_DZ_2013_18312DA_KT339120
0206_DZ_2013_16314_KT339136
06_cpx_DZ_2013_14596_KT339166
06G_DZ_2013_18244KA_KT339123
06_cpx_DZ_2013_16032_KT339137
06_cpx_DZ_2013_18963BH_KT339095
06_cpx_DZ_2001_6101rt_AY828656
06_cpx_DZ_2003_10303rt_AY828694
06_cpx_DZ_2003_0403RT_AY828589
06_cpx_DZ_2006_2306_EU743729
06_cpx_DZ_2005_29205_EU743715
06_cpx_DZ_2005_36805_EU743720
06_cpx_DZ_2002_3902rt_AY828631
06G_DZ_2013_15088_KT339173
06_cpx_DZ_2013_18622BS_KT339094
06_cpx_DZ_2005_16305_EU743701
0206_DZ_2013_19151ZM_KT339075
06_cpx_DZ_2013_18364BC_KT339082
G_DZ_2013_19668B_KT339071
0206_DZ_2013_15909_KT339147
06_cpx_DZ_2013_18032ZF_KT339068
06_cpx_DZ_2013_16425_KT339134
06_cpx_DZ_2013_14198_KT339161
06G_DZ_2013_18923BA_KT339088
06_cpx_DZ_2013_18718HB_KT339091
06_cpx_DZ_2013_18515MO_KT339114
06_cpx_DZ_2013_19456KK_KT339099
0.5701
0.9964
0.9966
0.453
0.8343
0.8522
0.9795
0.8355
0.8792
0.9118
0.9295
0.9938
0.9382
0.7858
0.9756
0.9151
0.9764
0.8401

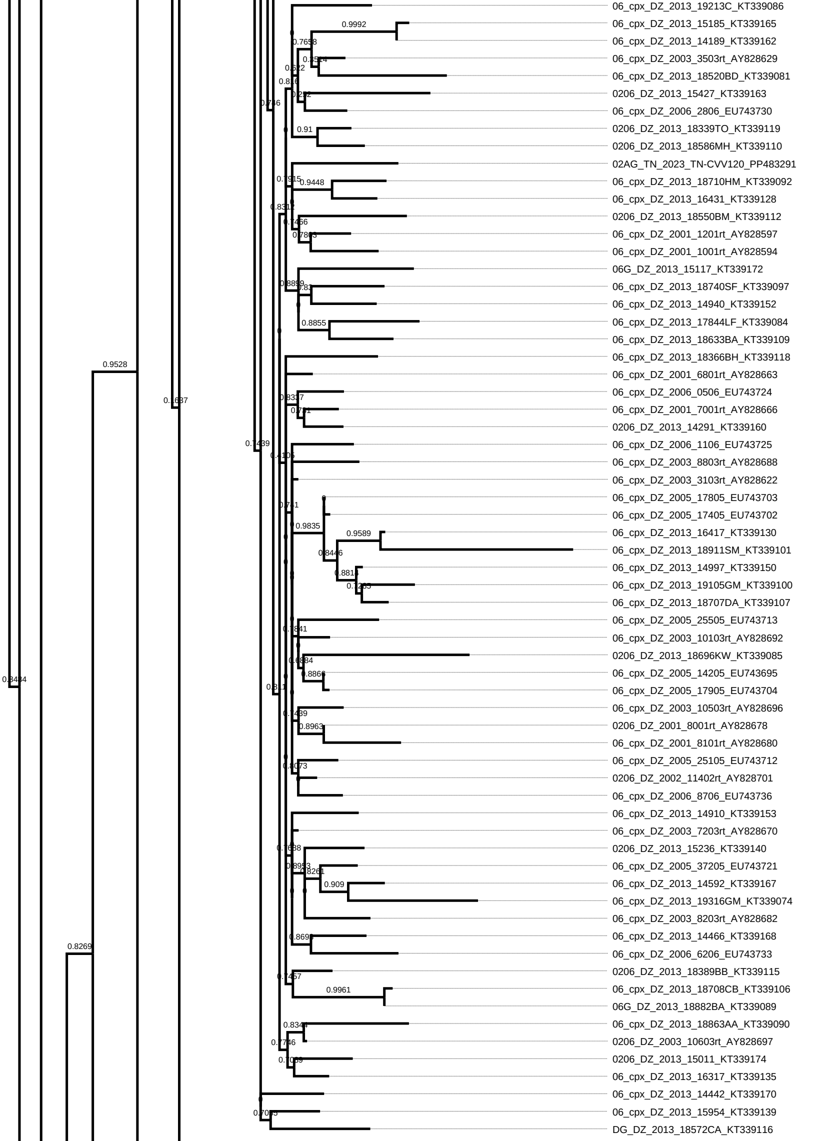
06_cpx_DZ_2013_19213C_KT339086
0.9992
06_cpx_DZ_2013_15185_KT339165
06_cpx_DZ_2013_14189_KT339162
06_cpx_DZ_2003_3503rt_AY828629
06_cpx_DZ_2013_18520BD_KT339081
0206_DZ_2013_15427_KT339163
06_cpx_DZ_2006_2806_EU743730
0.91
0206_DZ_2013_18339TO_KT339119
0206_DZ_2013_18586MH_KT339110
02AG_TN_2023_TN-CVV120_PP483291
0.9448
06_cpx_DZ_2013_18710HM_KT339092
06_cpx_DZ_2013_16431_KT339128
0206_DZ_2013_18550BM_KT339112
06_cpx_DZ_2001_1201rt_AY828597
06_cpx_DZ_2001_1001rt_AY828594
06G_DZ_2013_15117_KT339172
06_cpx_DZ_2013_18740SF_KT339097
06_cpx_DZ_2013_14940_KT339152
0.8855
06_cpx_DZ_2013_17844LF_KT339084
06_cpx_DZ_2013_18633BA_KT339109
0.9528
06_cpx_DZ_2013_18366BH_KT339118
06_cpx_DZ_2001_6801rt_AY828663
06_cpx_DZ_2006_0506_EU743724
06_cpx_DZ_2001_7001rt_AY828666
0206_DZ_2013_14291_KT339160
06_cpx_DZ_2006_1106_EU743725
06_cpx_DZ_2003_8803rt_AY828688
06_cpx_DZ_2003_3103rt_AY828622
06_cpx_DZ_2005_17805_EU743703
06_cpx_DZ_2005_17405_EU743702
0.9835
0.9589
06_cpx_DZ_2013_16417_KT339130
06_cpx_DZ_2013_18911SM_KT339101
06_cpx_DZ_2013_14997_KT339150
06_cpx_DZ_2013_19105GM_KT339100
06_cpx_DZ_2013_18707DA_KT339107
06_cpx_DZ_2005_25505_EU743713
06_cpx_DZ_2003_10103rt_AY828692
0206_DZ_2013_18696KW_KT339085
06_cpx_DZ_2005_14205_EU743695
06_cpx_DZ_2005_17905_EU743704
06_cpx_DZ_2003_10503rt_AY828696
0.8963
0206_DZ_2001_8001rt_AY828678
06_cpx_DZ_2001_8101rt_AY828680
06_cpx_DZ_2005_25105_EU743712
0206_DZ_2002_11402rt_AY828701
06_cpx_DZ_2006_8706_EU743736
06_cpx_DZ_2013_14910_KT339153
06_cpx_DZ_2003_7203rt_AY828670
0206_DZ_2013_15236_KT339140
06_cpx_DZ_2005_37205_EU743721
0.909
06_cpx_DZ_2013_14592_KT339167
06_cpx_DZ_2013_19316GM_KT339074
06_cpx_DZ_2003_8203rt_AY828682
0.8269
06_cpx_DZ_2013_14466_KT339168
06_cpx_DZ_2006_6206_EU743733
0206_DZ_2013_18389BB_KT339115
0.9961
06_cpx_DZ_2013_18708CB_KT339106
06G_DZ_2013_18882BA_KT339089
06_cpx_DZ_2013_18863AA_KT339090
0206_DZ_2003_10603rt_AY828697
0206_DZ_2013_15011_KT339174
06_cpx_DZ_2013_16317_KT339135
06_cpx_DZ_2013_14442_KT339170
06_cpx_DZ_2013_15954_KT339139
DG_DZ_2013_18572CA_KT339116

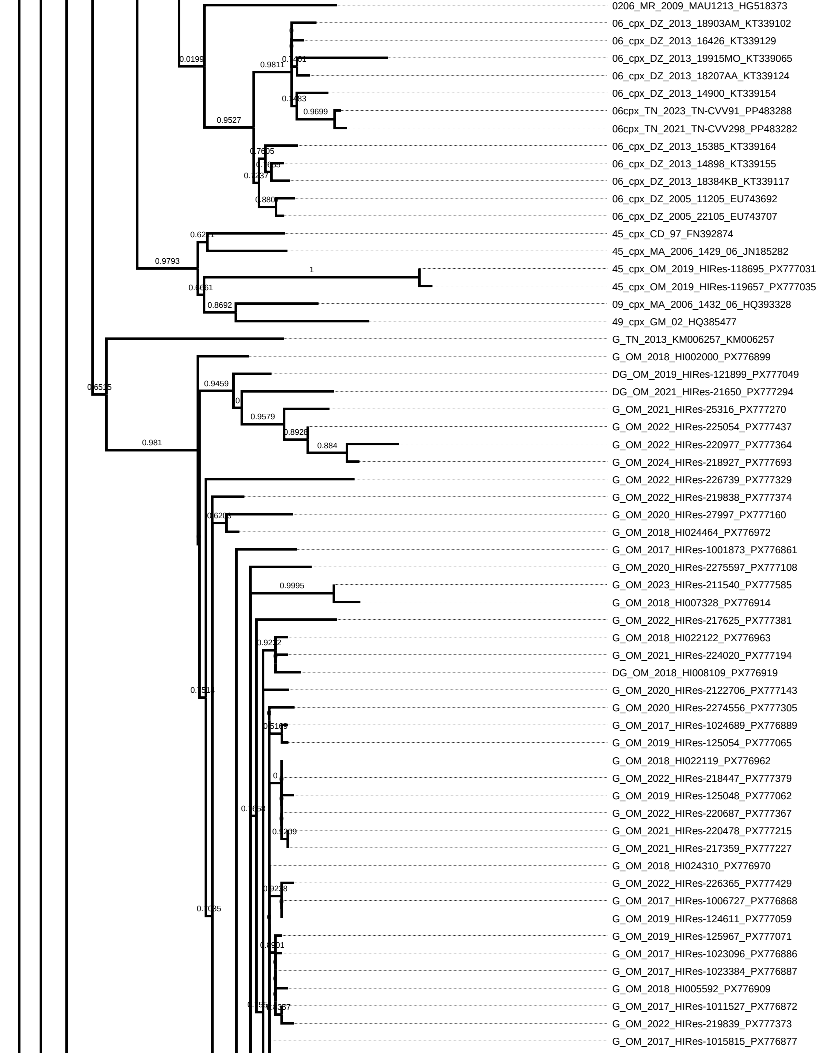
0206_MR_2009_MAU1213_HG518373
06_cpx_DZ_2013_18903AM_KT339102
06_cpx_DZ_2013_16426_KT339129
06_cpx_DZ_2013_19915MO_KT339065
06_cpx_DZ_2013_18207AA_KT339124
06_cpx_DZ_2013_14900_KT339154
06cpx_TN_2023_TN-CVV91_PP483288
06cpx_TN_2021_TN-CVV298_PP483282
06_cpx_DZ_2013_15385_KT339164
06_cpx_DZ_2013_14898_KT339155
06_cpx_DZ_2013_18384KB_KT339117
06_cpx_DZ_2005_11205_EU743692
06_cpx_DZ_2005_22105_EU743707
45_cpx_CD_97_FN392874
45_cpx_MA_2006_1429_06_JN185282
45_cpx_OM_2019_HIRes-118695_PX777031
45_cpx_OM_2019_HIRes-119657_PX777035
09_cpx_MA_2006_1432_06_HQ393328
49_cpx_GM_02_HQ385477
G_TN_2013_KM006257_KM006257
G_OM_2018_HI002000_PX776899
DG_OM_2019_HIRes-121899_PX777049
DG_OM_2021_HIRes-21650_PX777294
G_OM_2021_HIRes-25316_PX777270
G_OM_2022_HIRes-225054_PX777437
G_OM_2022_HIRes-220977_PX777364
G_OM_2024_HIRes-218927_PX777693
G_OM_2022_HIRes-226739_PX777329
G_OM_2022_HIRes-219838_PX777374
G_OM_2020_HIRes-27997_PX777160
G_OM_2018_HI024464_PX776972
G_OM_2017_HIRes-1001873_PX776861
G_OM_2020_HIRes-2275597_PX777108
G_OM_2023_HIRes-211540_PX777585
G_OM_2018_HI007328_PX776914
G_OM_2022_HIRes-217625_PX777381
G_OM_2018_HI022122_PX776963
G_OM_2021_HIRes-224020_PX777194
DG_OM_2018_HI008109_PX776919
G_OM_2020_HIRes-2122706_PX777143
G_OM_2020_HIRes-2274556_PX777305
G_OM_2017_HIRes-1024689_PX776889
G_OM_2019_HIRes-125054_PX777065
G_OM_2018_HI022119_PX776962
G_OM_2022_HIRes-218447_PX777379
G_OM_2019_HIRes-125048_PX777062
G_OM_2022_HIRes-220687_PX777367
G_OM_2021_HIRes-220478_PX777215
G_OM_2021_HIRes-217359_PX777227
G_OM_2018_HI024310_PX776970
G_OM_2022_HIRes-226365_PX777429
G_OM_2017_HIRes-1006727_PX776868
G_OM_2019_HIRes-124611_PX777059
G_OM_2019_HIRes-125967_PX777071
G_OM_2017_HIRes-1023096_PX776886
G_OM_2017_HIRes-1023384_PX776887
G_OM_2018_HI005592_PX776909
G_OM_2017_HIRes-1011527_PX776872
G_OM_2022_HIRes-219839_PX777373
G_OM_2017_HIRes-1015815_PX776877
0.0199
0.9811
0.9527
0.9699
0.9793
1
0.8692
0.9459
0.9579
0.981
0.884
0.9995

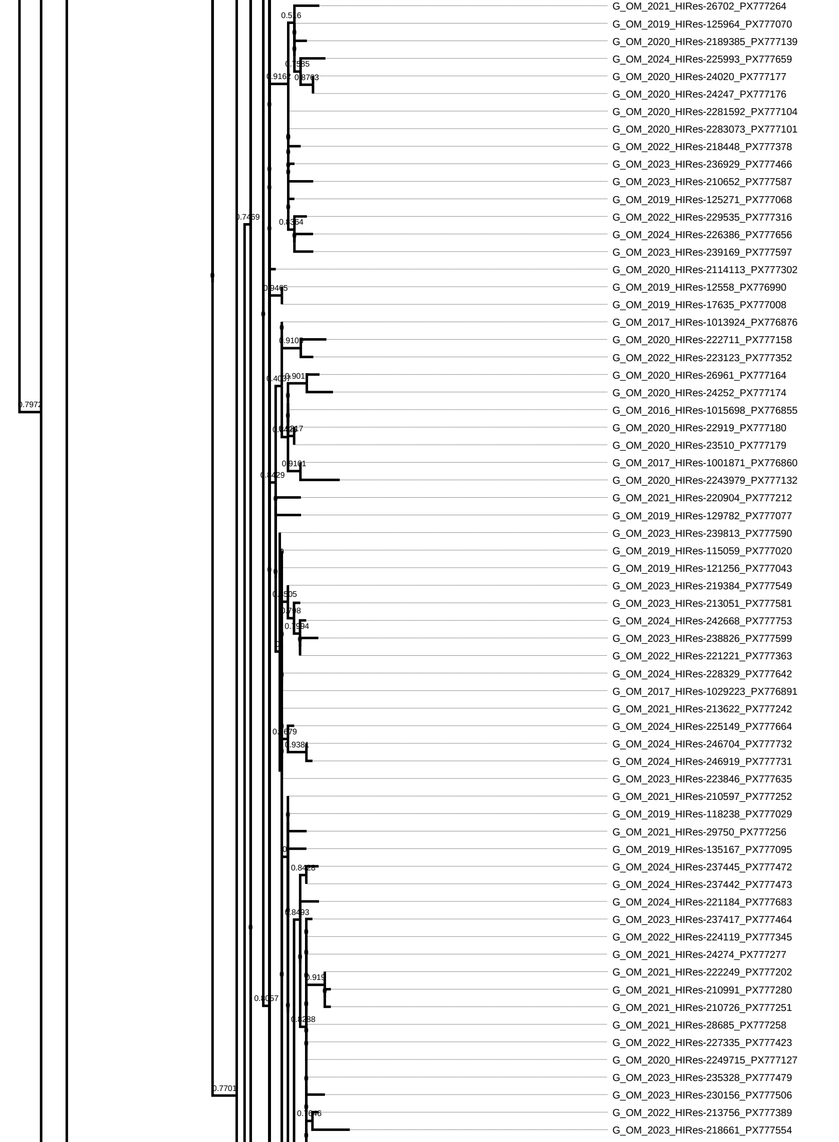

G_OM_2021_HIRes-26702_PX777264
G_OM_2019_HIRes-125964_PX777070
G_OM_2020_HIRes-2189385_PX777139
G_OM_2024_HIRes-225993_PX777659
G_OM_2020_HIRes-24020_PX777177
G_OM_2020_HIRes-24247_PX777176
G_OM_2020_HIRes-2281592_PX777104
G_OM_2020_HIRes-2283073_PX777101
G_OM_2022_HIRes-218448_PX777378
G_OM_2023_HIRes-236929_PX777466
G_OM_2023_HIRes-210652_PX777587
G_OM_2019_HIRes-125271_PX777068
G_OM_2022_HIRes-229535_PX777316
G_OM_2024_HIRes-226386_PX777656
G_OM_2023_HIRes-239169_PX777597
G_OM_2020_HIRes-2114113_PX777302
G_OM_2019_HIRes-12558_PX776990
G_OM_2019_HIRes-17635_PX777008
G_OM_2017_HIRes-1013924_PX776876
G_OM_2020_HIRes-222711_PX777158
G_OM_2022_HIRes-223123_PX777352
G_OM_2020_HIRes-26961_PX777164
G_OM_2020_HIRes-24252_PX777174
G_OM_2016_HIRes-1015698_PX776855
G_OM_2020_HIRes-22919_PX777180
G_OM_2020_HIRes-23510_PX777179
G_OM_2017_HIRes-1001871_PX776860
G_OM_2020_HIRes-2243979_PX777132
G_OM_2021_HIRes-220904_PX777212
G_OM_2019_HIRes-129782_PX777077
G_OM_2023_HIRes-239813_PX777590
G_OM_2019_HIRes-115059_PX777020
G_OM_2019_HIRes-121256_PX777043
G_OM_2023_HIRes-219384_PX777549
G_OM_2023_HIRes-213051_PX777581
G_OM_2024_HIRes-242668_PX777753
G_OM_2023_HIRes-238826_PX777599
G_OM_2022_HIRes-221221_PX777363
G_OM_2024_HIRes-228329_PX777642
G_OM_2017_HIRes-1029223_PX776891
G_OM_2021_HIRes-213622_PX777242
G_OM_2024_HIRes-225149_PX777664
G_OM_2024_HIRes-246704_PX777732
G_OM_2024_HIRes-246919_PX777731
G_OM_2023_HIRes-223846_PX777635
G_OM_2021_HIRes-210597_PX777252
G_OM_2019_HIRes-118238_PX777029
G_OM_2021_HIRes-29750_PX777256
G_OM_2019_HIRes-135167_PX777095
G_OM_2024_HIRes-237445_PX777472
G_OM_2024_HIRes-237442_PX777473
G_OM_2024_HIRes-221184_PX777683
G_OM_2023_HIRes-237417_PX777464
G_OM_2022_HIRes-224119_PX777345
G_OM_2021_HIRes-24274_PX777277
G_OM_2021_HIRes-222249_PX777202
G_OM_2021_HIRes-210991_PX777280
G_OM_2021_HIRes-210726_PX777251
G_OM_2021_HIRes-28685_PX777258
G_OM_2022_HIRes-227335_PX777423
G_OM_2020_HIRes-2249715_PX777127
G_OM_2023_HIRes-235328_PX777479
G_OM_2023_HIRes-230156_PX777506
G_OM_2022_HIRes-213756_PX777389
G_OM_2023_HIRes-218661_PX777554
0.9162
0.7469
0.7701
0.8057

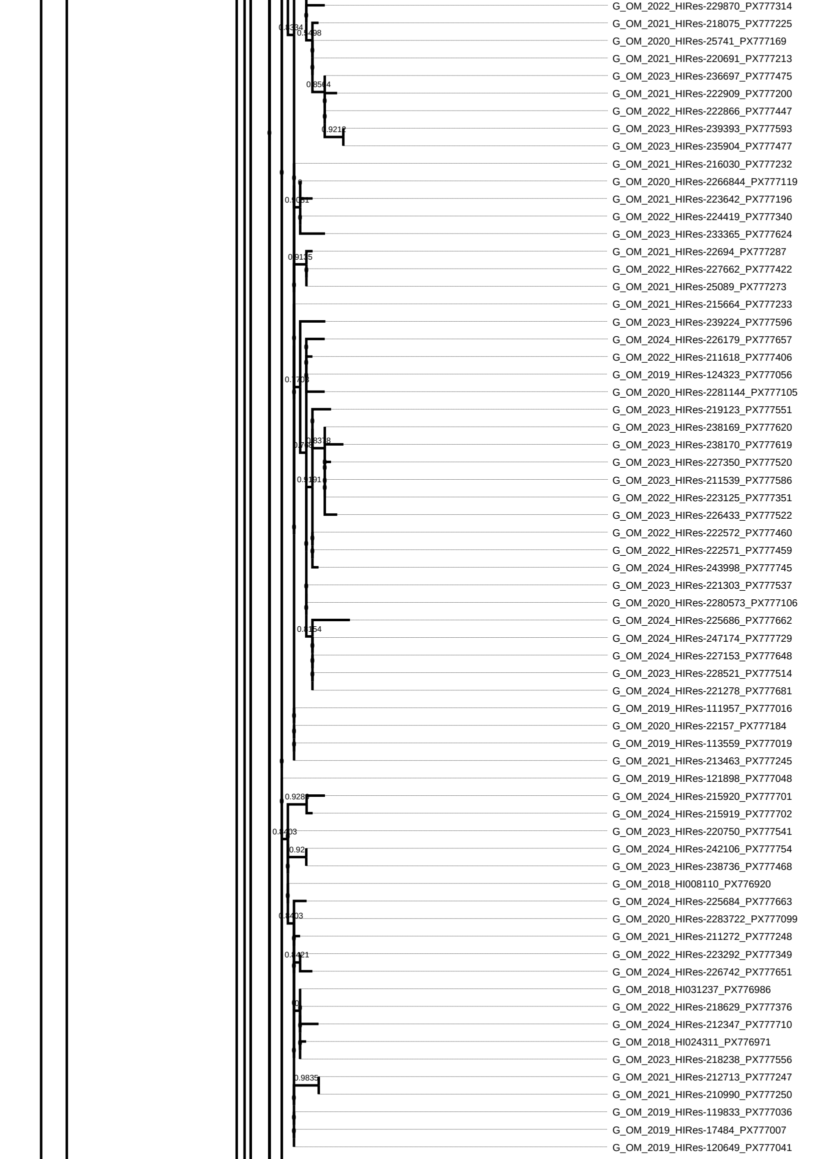
G_OM_2022_HIRes-229870_PX777314
G_OM_2021_HIRes-218075_PX777225
G_OM_2020_HIRes-25741_PX777169
G_OM_2021_HIRes-220691_PX777213
G_OM_2023_HIRes-236697_PX777475
G_OM_2021_HIRes-222909_PX777200
G_OM_2022_HIRes-222866_PX777447
G_OM_2023_HIRes-239393_PX777593
G_OM_2023_HIRes-235904_PX777477
G_OM_2021_HIRes-216030_PX777232
G_OM_2020_HIRes-2266844_PX777119
G_OM_2021_HIRes-223642_PX777196
G_OM_2022_HIRes-224419_PX777340
G_OM_2023_HIRes-233365_PX777624
G_OM_2021_HIRes-22694_PX777287
G_OM_2022_HIRes-227662_PX777422
G_OM_2021_HIRes-25089_PX777273
G_OM_2021_HIRes-215664_PX777233
G_OM_2023_HIRes-239224_PX777596
G_OM_2024_HIRes-226179_PX777657
G_OM_2022_HIRes-211618_PX777406
G_OM_2019_HIRes-124323_PX777056
G_OM_2020_HIRes-2281144_PX777105
G_OM_2023_HIRes-219123_PX777551
G_OM_2023_HIRes-238169_PX777620
G_OM_2023_HIRes-238170_PX777619
G_OM_2023_HIRes-227350_PX777520
G_OM_2023_HIRes-211539_PX777586
G_OM_2022_HIRes-223125_PX777351
G_OM_2023_HIRes-226433_PX777522
G_OM_2022_HIRes-222572_PX777460
G_OM_2022_HIRes-222571_PX777459
G_OM_2024_HIRes-243998_PX777745
G_OM_2023_HIRes-221303_PX777537
G_OM_2020_HIRes-2280573_PX777106
G_OM_2024_HIRes-225686_PX777662
G_OM_2024_HIRes-247174_PX777729
G_OM_2024_HIRes-227153_PX777648
G_OM_2023_HIRes-228521_PX777514
G_OM_2024_HIRes-221278_PX777681
G_OM_2019_HIRes-111957_PX777016
G_OM_2020_HIRes-22157_PX777184
G_OM_2019_HIRes-113559_PX777019
G_OM_2021_HIRes-213463_PX777245
G_OM_2019_HIRes-121898_PX777048
G_OM_2024_HIRes-215920_PX777701
G_OM_2024_HIRes-215919_PX777702
G_OM_2023_HIRes-220750_PX777541
G_OM_2024_HIRes-242106_PX777754
G_OM_2023_HIRes-238736_PX777468
G_OM_2018_HI008110_PX776920
G_OM_2024_HIRes-225684_PX777663
G_OM_2020_HIRes-2283722_PX777099
G_OM_2021_HIRes-211272_PX777248
G_OM_2022_HIRes-223292_PX777349
G_OM_2024_HIRes-226742_PX777651
G_OM_2018_HI031237_PX776986
G_OM_2022_HIRes-218629_PX777376
G_OM_2024_HIRes-212347_PX777710
G_OM_2018_HI024311_PX776971
G_OM_2023_HIRes-218238_PX777556
G_OM_2021_HIRes-212713_PX777247
G_OM_2021_HIRes-210990_PX777250
G_OM_2019_HIRes-119833_PX777036
G_OM_2019_HIRes-17484_PX777007
G_OM_2019_HIRes-120649_PX777041

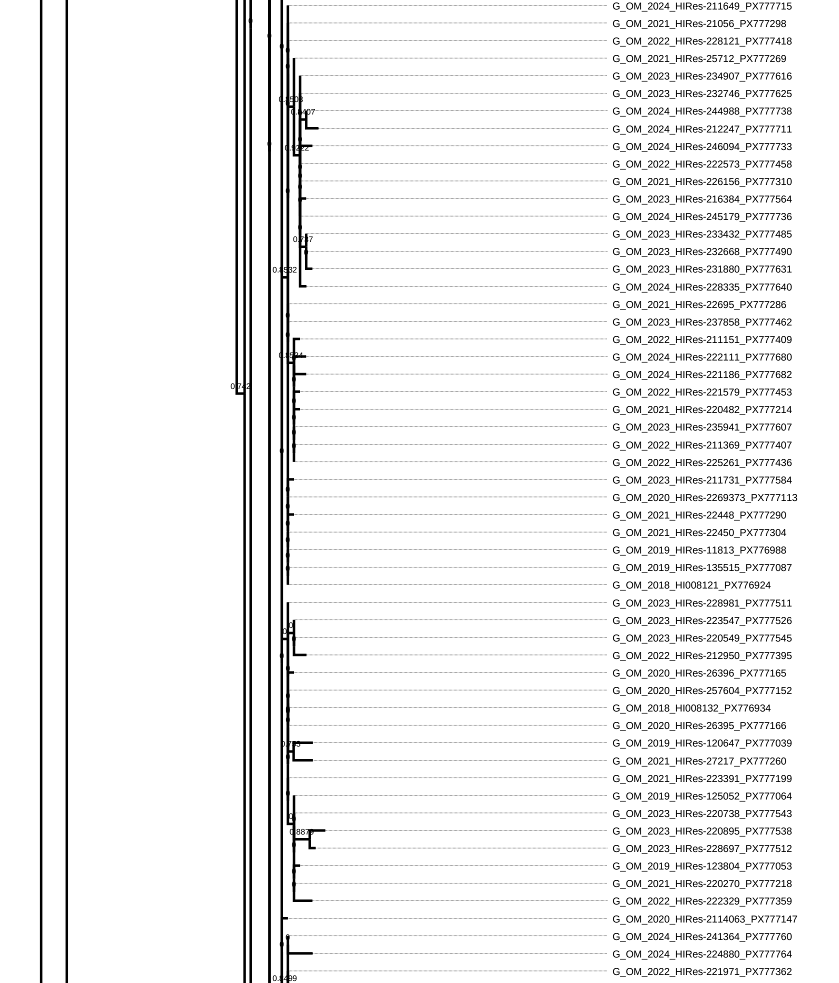
G_OM_2024_HIRes-211649_PX777715
G_OM_2021_HIRes-21056_PX777298
G_OM_2022_HIRes-228121_PX777418
G_OM_2021_HIRes-25712_PX777269
G_OM_2023_HIRes-234907_PX777616
G_OM_2023_HIRes-232746_PX777625
G_OM_2024_HIRes-244988_PX777738
G_OM_2024_HIRes-212247_PX777711
G_OM_2024_HIRes-246094_PX777733
G_OM_2022_HIRes-222573_PX777458
G_OM_2021_HIRes-226156_PX777310
G_OM_2023_HIRes-216384_PX777564
G_OM_2024_HIRes-245179_PX777736
G_OM_2023_HIRes-233432_PX777485
G_OM_2023_HIRes-232668_PX777490
G_OM_2023_HIRes-231880_PX777631
G_OM_2024_HIRes-228335_PX777640
G_OM_2021_HIRes-22695_PX777286
G_OM_2023_HIRes-237858_PX777462
G_OM_2022_HIRes-211151_PX777409
G_OM_2024_HIRes-222111_PX777680
G_OM_2024_HIRes-221186_PX777682
G_OM_2022_HIRes-221579_PX777453
G_OM_2021_HIRes-220482_PX777214
G_OM_2023_HIRes-235941_PX777607
G_OM_2022_HIRes-211369_PX777407
G_OM_2022_HIRes-225261_PX777436
G_OM_2023_HIRes-211731_PX777584
G_OM_2020_HIRes-2269373_PX777113
G_OM_2021_HIRes-22448_PX777290
G_OM_2021_HIRes-22450_PX777304
G_OM_2019_HIRes-11813_PX776988
G_OM_2019_HIRes-135515_PX777087
G_OM_2018_HI008121_PX776924
G_OM_2023_HIRes-228981_PX777511
G_OM_2023_HIRes-223547_PX777526
G_OM_2023_HIRes-220549_PX777545
G_OM_2022_HIRes-212950_PX777395
G_OM_2020_HIRes-26396_PX777165
G_OM_2020_HIRes-257604_PX777152
G_OM_2018_HI008132_PX776934
G_OM_2020_HIRes-26395_PX777166
G_OM_2019_HIRes-120647_PX777039
G_OM_2021_HIRes-27217_PX777260
G_OM_2021_HIRes-223391_PX777199
G_OM_2019_HIRes-125052_PX777064
G_OM_2023_HIRes-220738_PX777543
G_OM_2023_HIRes-220895_PX777538
G_OM_2023_HIRes-228697_PX777512
G_OM_2019_HIRes-123804_PX777053
G_OM_2021_HIRes-220270_PX777218
G_OM_2022_HIRes-222329_PX777359
G_OM_2020_HIRes-2114063_PX777147
G_OM_2024_HIRes-241364_PX777760
G_OM_2024_HIRes-224880_PX777764
G_OM_2022_HIRes-221971_PX777362
0.742

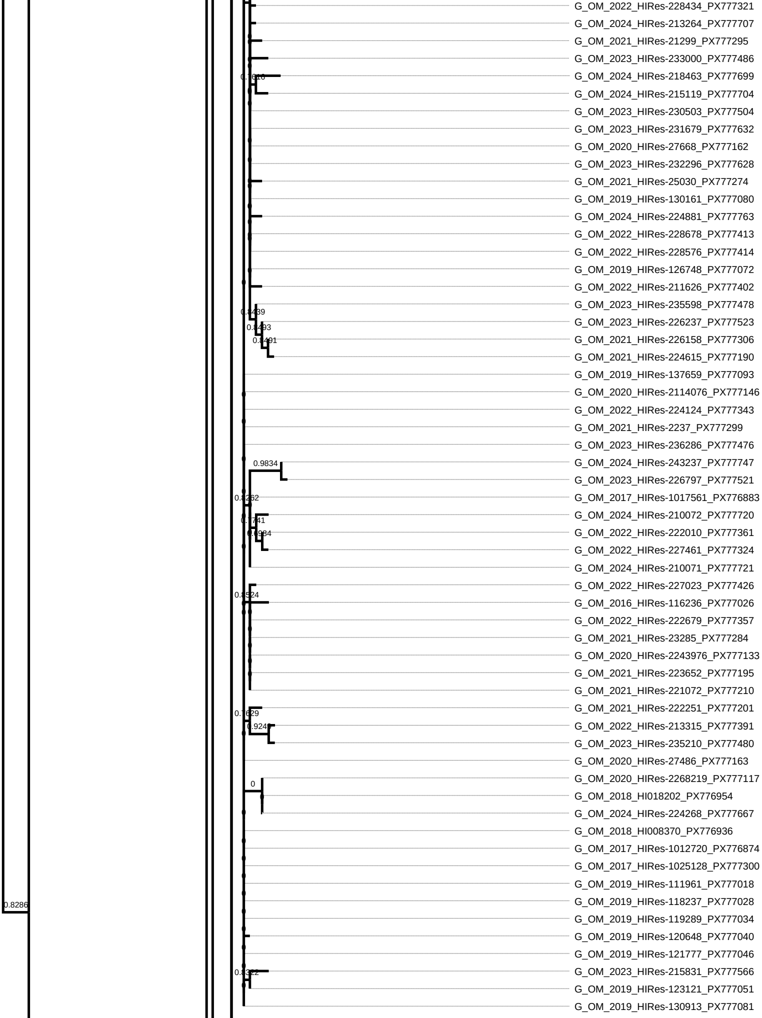
G_OM_2022_HIRes-228434_PX777321
G_OM_2024_HIRes-213264_PX777707
G_OM_2021_HIRes-21299_PX777295
G_OM_2023_HIRes-233000_PX777486
G_OM_2024_HIRes-218463_PX777699
G_OM_2024_HIRes-215119_PX777704
G_OM_2023_HIRes-230503_PX777504
G_OM_2023_HIRes-231679_PX777632
G_OM_2020_HIRes-27668_PX777162
G_OM_2023_HIRes-232296_PX777628
G_OM_2021_HIRes-25030_PX777274
G_OM_2019_HIRes-130161_PX777080
G_OM_2024_HIRes-224881_PX777763
G_OM_2022_HIRes-228678_PX777413
G_OM_2022_HIRes-228576_PX777414
G_OM_2019_HIRes-126748_PX777072
G_OM_2022_HIRes-211626_PX777402
G_OM_2023_HIRes-235598_PX777478
0.8439
G_OM_2023_HIRes-226237_PX777523
0.8493
0.8491
G_OM_2021_HIRes-226158_PX777306
G_OM_2021_HIRes-224615_PX777190
G_OM_2019_HIRes-137659_PX777093
G_OM_2020_HIRes-2114076_PX777146
G_OM_2022_HIRes-224124_PX777343
G_OM_2021_HIRes-2237_PX777299
G_OM_2023_HIRes-236286_PX777476
0.9834
G_OM_2024_HIRes-243237_PX777747
G_OM_2023_HIRes-226797_PX777521
0.8262
G_OM_2017_HIRes-1017561_PX776883
G_OM_2024_HIRes-210072_PX777720
G_OM_2022_HIRes-222010_PX777361
G_OM_2022_HIRes-227461_PX777324
G_OM_2024_HIRes-210071_PX777721
G_OM_2022_HIRes-227023_PX777426
0.8524
G_OM_2016_HIRes-116236_PX777026
G_OM_2022_HIRes-222679_PX777357
G_OM_2021_HIRes-23285_PX777284
G_OM_2020_HIRes-2243976_PX777133
G_OM_2021_HIRes-223652_PX777195
G_OM_2021_HIRes-221072_PX777210
G_OM_2021_HIRes-222251_PX777201
0.7629
G_OM_2022_HIRes-213315_PX777391
G_OM_2023_HIRes-235210_PX777480
G_OM_2020_HIRes-27486_PX777163
0
G_OM_2020_HIRes-2268219_PX777117
G_OM_2018_HI018202_PX776954
G_OM_2024_HIRes-224268_PX777667
G_OM_2018_HI008370_PX776936
G_OM_2017_HIRes-1012720_PX776874
G_OM_2017_HIRes-1025128_PX777300
G_OM_2019_HIRes-111961_PX777018
0.8286
G_OM_2019_HIRes-118237_PX777028
G_OM_2019_HIRes-119289_PX777034
G_OM_2019_HIRes-120648_PX777040
G_OM_2019_HIRes-121777_PX777046
G_OM_2023_HIRes-215831_PX777566
G_OM_2019_HIRes-123121_PX777051
G_OM_2019_HIRes-130913_PX777081

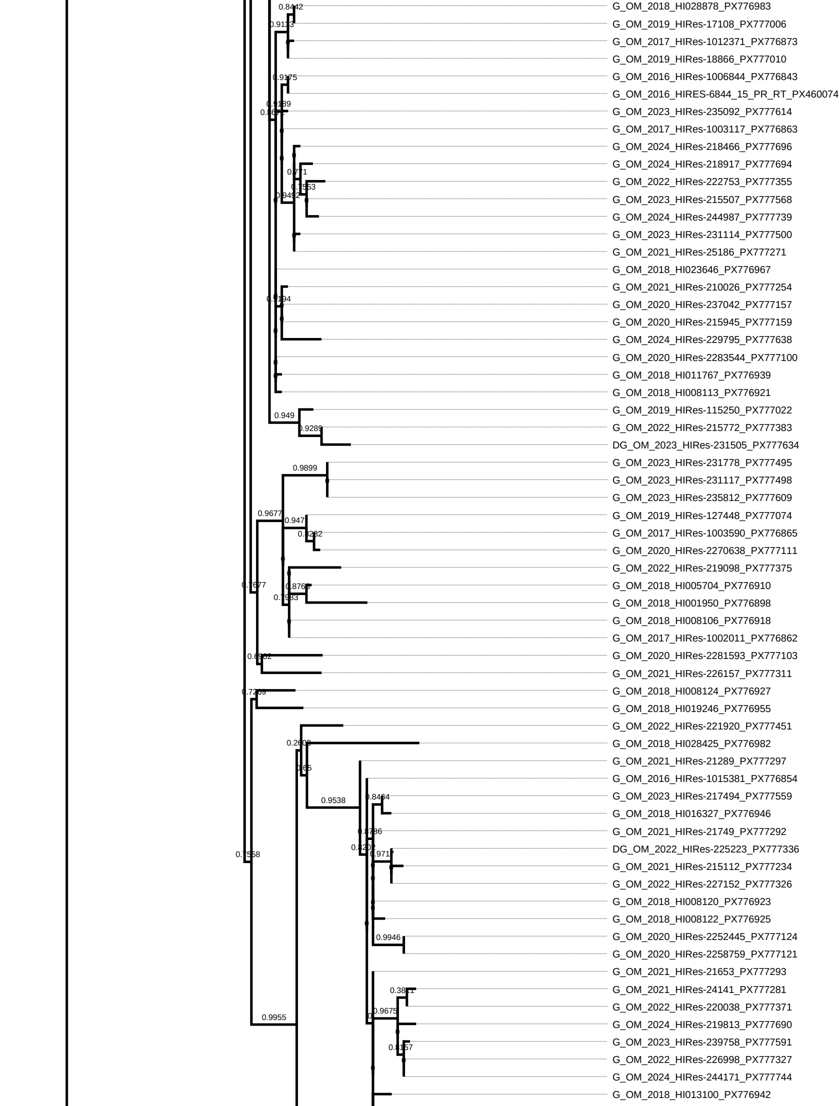

0.8442
G_OM_2018_HI028878_PX776983
G_OM_2019_HIRes-17108_PX777006
G_OM_2017_HIRes-1012371_PX776873
G_OM_2019_HIRes-18866_PX777010
0.9175
G_OM_2016_HIRes-1006844_PX776843
G_OM_2016_HIRES-6844_15_PR_RT_PX460074
G_OM_2023_HIRes-235092_PX777614
G_OM_2017_HIRes-1003117_PX776863
G_OM_2024_HIRes-218466_PX777696
G_OM_2024_HIRes-218917_PX777694
G_OM_2022_HIRes-222753_PX777355
G_OM_2023_HIRes-215507_PX777568
G_OM_2024_HIRes-244987_PX777739
G_OM_2023_HIRes-231114_PX777500
G_OM_2021_HIRes-25186_PX777271
G_OM_2018_HI023646_PX776967
G_OM_2021_HIRes-210026_PX777254
G_OM_2020_HIRes-237042_PX777157
G_OM_2020_HIRes-215945_PX777159
G_OM_2024_HIRes-229795_PX777638
G_OM_2020_HIRes-2283544_PX777100
G_OM_2018_HI011767_PX776939
G_OM_2018_HI008113_PX776921
0.949
G_OM_2019_HIRes-115250_PX777022
G_OM_2022_HIRes-215772_PX777383
DG_OM_2023_HIRes-231505_PX777634
0.9899
G_OM_2023_HIRes-231778_PX777495
G_OM_2023_HIRes-231117_PX777498
G_OM_2023_HIRes-235812_PX777609
0.9677
0.947
G_OM_2019_HIRes-127448_PX777074
G_OM_2017_HIRes-1003590_PX776865
G_OM_2020_HIRes-2270638_PX777111
G_OM_2022_HIRes-219098_PX777375
G_OM_2018_HI005704_PX776910
G_OM_2018_HI001950_PX776898
G_OM_2018_HI008106_PX776918
G_OM_2017_HIRes-1002011_PX776862
G_OM_2020_HIRes-2281593_PX777103
G_OM_2021_HIRes-226157_PX777311
G_OM_2018_HI008124_PX776927
G_OM_2018_HI019246_PX776955
G_OM_2022_HIRes-221920_PX777451
G_OM_2018_HI028425_PX776982
G_OM_2021_HIRes-21289_PX777297
G_OM_2016_HIRes-1015381_PX776854
0.9538
G_OM_2023_HIRes-217494_PX777559
G_OM_2018_HI016327_PX776946
G_OM_2021_HIRes-21749_PX777292
DG_OM_2022_HIRes-225223_PX777336
G_OM_2021_HIRes-215112_PX777234
G_OM_2022_HIRes-227152_PX777326
G_OM_2018_HI008120_PX776923
G_OM_2018_HI008122_PX776925
0.9946
G_OM_2020_HIRes-2252445_PX777124
G_OM_2020_HIRes-2258759_PX777121
G_OM_2021_HIRes-21653_PX777293
G_OM_2021_HIRes-24141_PX777281
G_OM_2022_HIRes-220038_PX777371
0.9675
0.9955
G_OM_2024_HIRes-219813_PX777690
G_OM_2023_HIRes-239758_PX777591
G_OM_2022_HIRes-226998_PX777327
G_OM_2024_HIRes-244171_PX777744
G_OM_2018_HI013100_PX776942

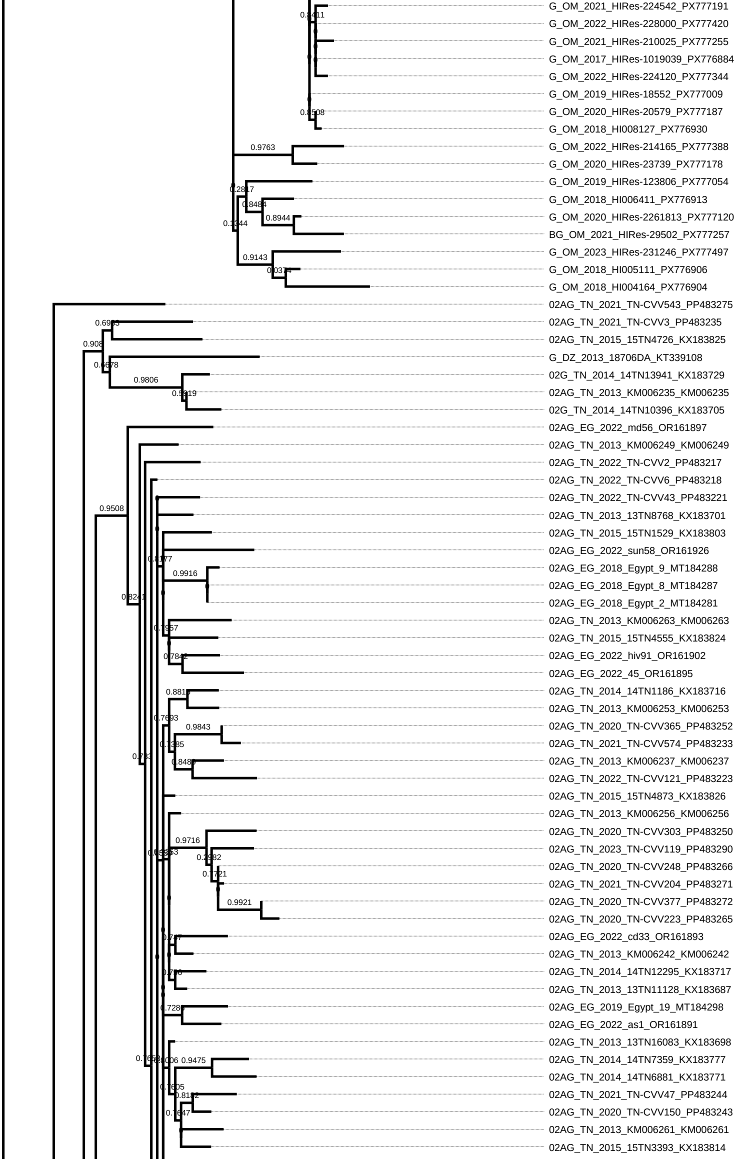
G_OM_2021_HIRes-224542_PX777191
G_OM_2022_HIRes-228000_PX777420
G_OM_2021_HIRes-210025_PX777255
G_OM_2017_HIRes-1019039_PX776884
G_OM_2022_HIRes-224120_PX777344
G_OM_2019_HIRes-18552_PX777009
G_OM_2020_HIRes-20579_PX777187
G_OM_2018_HI008127_PX776930
0.9763
G_OM_2022_HIRes-214165_PX777388
G_OM_2020_HIRes-23739_PX777178
G_OM_2019_HIRes-123806_PX777054
G_OM_2018_HI006411_PX776913
0.8944
G_OM_2020_HIRes-2261813_PX777120
BG_OM_2021_HIRes-29502_PX777257
0.9143
G_OM_2023_HIRes-231246_PX777497
G_OM_2018_HI005111_PX776906
G_OM_2018_HI004164_PX776904
02AG_TN_2021_TN-CVV543_PP483275
02AG_TN_2021_TN-CVV3_PP483235
02AG_TN_2015_15TN4726_KX183825
G_DZ_2013_18706DA_KT339108
0.9806
02G_TN_2014_14TN13941_KX183729
02AG_TN_2013_KM006235_KM006235
02G_TN_2014_14TN10396_KX183705
02AG_EG_2022_md56_OR161897
02AG_TN_2013_KM006249_KM006249
02AG_TN_2022_TN-CVV2_PP483217
02AG_TN_2022_TN-CVV6_PP483218
02AG_TN_2022_TN-CVV43_PP483221
0.9508
02AG_TN_2013_13TN8768_KX183701
02AG_TN_2015_15TN1529_KX183803
02AG_EG_2022_sun58_OR161926
0.9916
02AG_EG_2018_Egypt_9_MT184288
02AG_EG_2018_Egypt_8_MT184287
02AG_EG_2018_Egypt_2_MT184281
02AG_TN_2013_KM006263_KM006263
02AG_TN_2015_15TN4555_KX183824
02AG_EG_2022_hiv91_OR161902
02AG_EG_2022_45_OR161895
02AG_TN_2014_14TN1186_KX183716
02AG_TN_2013_KM006253_KM006253
0.9843
02AG_TN_2020_TN-CVV365_PP483252
02AG_TN_2021_TN-CVV574_PP483233
02AG_TN_2013_KM006237_KM006237
02AG_TN_2022_TN-CVV121_PP483223
02AG_TN_2015_15TN4873_KX183826
02AG_TN_2013_KM006256_KM006256
0.9716
02AG_TN_2020_TN-CVV303_PP483250
02AG_TN_2023_TN-CVV119_PP483290
02AG_TN_2020_TN-CVV248_PP483266
02AG_TN_2021_TN-CVV204_PP483271
0.9921
02AG_TN_2020_TN-CVV377_PP483272
02AG_TN_2020_TN-CVV223_PP483265
02AG_EG_2022_cd33_OR161893
02AG_TN_2013_KM006242_KM006242
02AG_TN_2014_14TN12295_KX183717
02AG_TN_2013_13TN11128_KX183687
02AG_EG_2019_Egypt_19_MT184298
02AG_EG_2022_as1_OR161891
02AG_TN_2013_13TN16083_KX183698
0.9475
02AG_TN_2014_14TN7359_KX183777
02AG_TN_2014_14TN6881_KX183771
02AG_TN_2021_TN-CVV47_PP483244
02AG_TN_2020_TN-CVV150_PP483243
02AG_TN_2013_KM006261_KM006261
02AG_TN_2015_15TN3393_KX183814

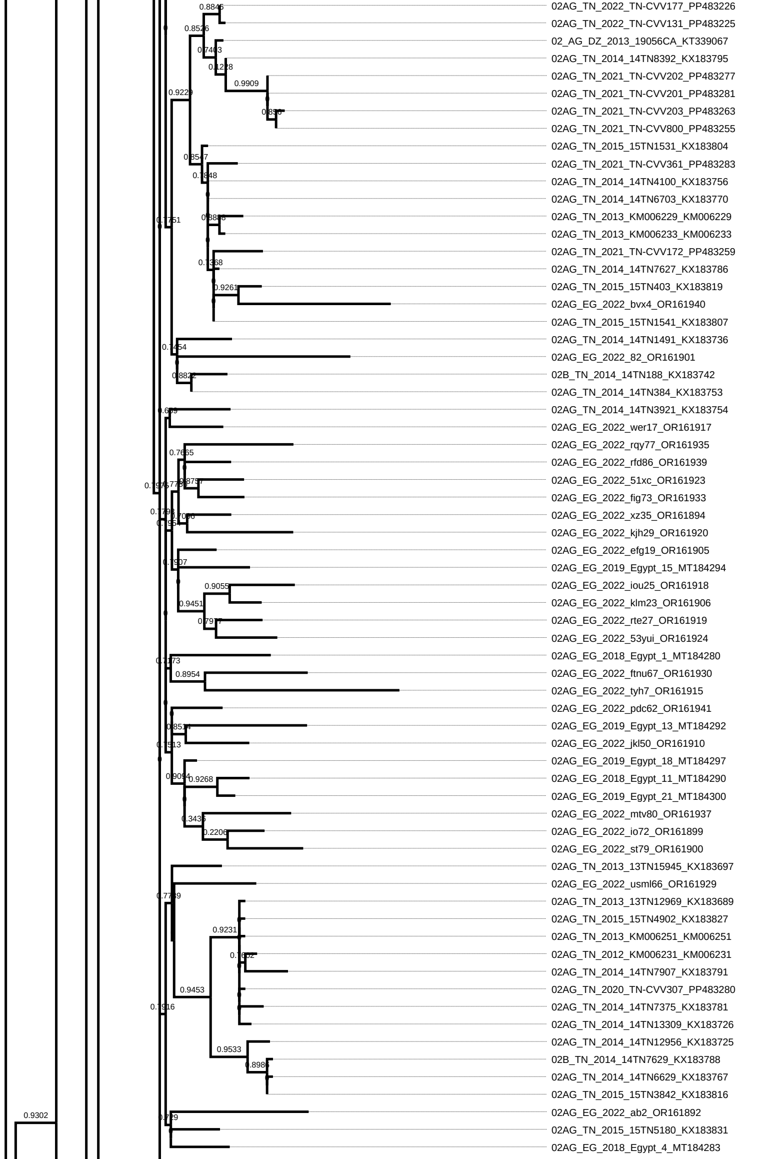

02AG_TN_2022_TN-CVV177_PP483226
02AG_TN_2022_TN-CVV131_PP483225
02_AG_DZ_2013_19056CA_KT339067
02AG_TN_2014_14TN8392_KX183795
02AG_TN_2021_TN-CVV202_PP483277
02AG_TN_2021_TN-CVV201_PP483281
02AG_TN_2021_TN-CVV203_PP483263
02AG_TN_2021_TN-CVV800_PP483255
02AG_TN_2015_15TN1531_KX183804
02AG_TN_2021_TN-CVV361_PP483283
02AG_TN_2014_14TN4100_KX183756
02AG_TN_2014_14TN6703_KX183770
02AG_TN_2013_KM006229_KM006229
02AG_TN_2013_KM006233_KM006233
02AG_TN_2021_TN-CVV172_PP483259
02AG_TN_2014_14TN7627_KX183786
02AG_TN_2015_15TN403_KX183819
02AG_EG_2022_bvx4_OR161940
02AG_TN_2015_15TN1541_KX183807
02AG_TN_2014_14TN1491_KX183736
02AG_EG_2022_82_OR161901
02B_TN_2014_14TN188_KX183742
02AG_TN_2014_14TN384_KX183753
02AG_TN_2014_14TN3921_KX183754
02AG_EG_2022_wer17_OR161917
02AG_EG_2022_rqy77_OR161935
02AG_EG_2022_rfd86_OR161939
02AG_EG_2022_51xc_OR161923
02AG_EG_2022_fig73_OR161933
02AG_EG_2022_xz35_OR161894
02AG_EG_2022_kjh29_OR161920
02AG_EG_2022_efg19_OR161905
02AG_EG_2019_Egypt_15_MT184294
02AG_EG_2022_iou25_OR161918
02AG_EG_2022_klm23_OR161906
02AG_EG_2022_rte27_OR161919
02AG_EG_2022_53yui_OR161924
02AG_EG_2018_Egypt_1_MT184280
02AG_EG_2022_ftnu67_OR161930
02AG_EG_2022_tyh7_OR161915
02AG_EG_2022_pdc62_OR161941
02AG_EG_2019_Egypt_13_MT184292
02AG_EG_2022_jkl50_OR161910
02AG_EG_2019_Egypt_18_MT184297
02AG_EG_2018_Egypt_11_MT184290
02AG_EG_2019_Egypt_21_MT184300
02AG_EG_2022_mtv80_OR161937
02AG_EG_2022_io72_OR161899
02AG_EG_2022_st79_OR161900
02AG_TN_2013_13TN15945_KX183697
02AG_EG_2022_usml66_OR161929
02AG_TN_2013_13TN12969_KX183689
02AG_TN_2015_15TN4902_KX183827
02AG_TN_2013_KM006251_KM006251
02AG_TN_2012_KM006231_KM006231
02AG_TN_2014_14TN7907_KX183791
02AG_TN_2020_TN-CVV307_PP483280
02AG_TN_2014_14TN7375_KX183781
02AG_TN_2014_14TN13309_KX183726
02AG_TN_2014_14TN12956_KX183725
02B_TN_2014_14TN7629_KX183788
02AG_TN_2014_14TN6629_KX183767
02AG_TN_2015_15TN3842_KX183816
02AG_EG_2022_ab2_OR161892
02AG_TN_2015_15TN5180_KX183831
02AG_EG_2018_Egypt_4_MT184283
0.9909
0.9261
0.9055
0.9451
0.8954
0.9268
0.9231
0.9453
0.9533
0.9302

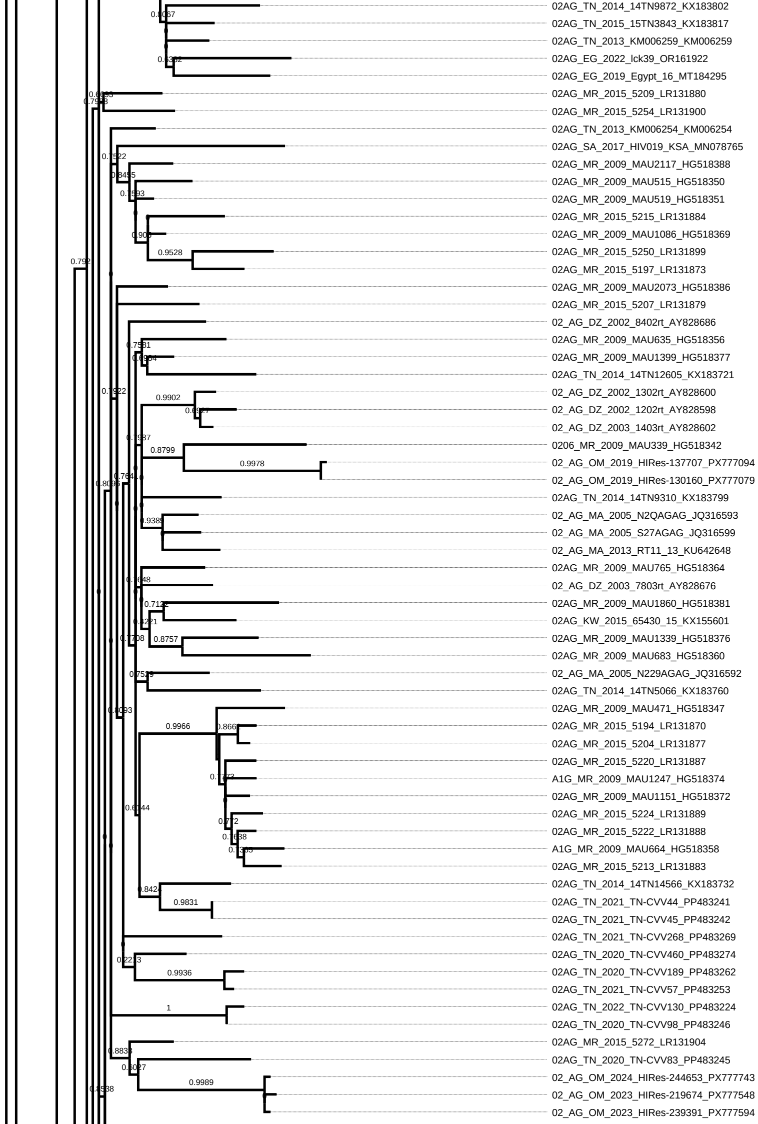

02AG_TN_2014_14TN9872_KX183802
02AG_TN_2015_15TN3843_KX183817
02AG_TN_2013_KM006259_KM006259
02AG_EG_2022_Ick39_OR161922
02AG_EG_2019_Egypt_16_MT184295
02AG_MR_2015_5209_LR131880
02AG_MR_2015_5254_LR131900
02AG_TN_2013_KM006254_KM006254
02AG_SA_2017_HIV019_KSA_MN078765
02AG_MR_2009_MAU2117_HG518388
02AG_MR_2009_MAU515_HG518350
02AG_MR_2009_MAU519_HG518351
02AG_MR_2015_5215_LR131884
02AG_MR_2009_MAU1086_HG518369
0.9528
02AG_MR_2015_5250_LR131899
0.792
02AG_MR_2015_5197_LR131873
02AG_MR_2009_MAU2073_HG518386
02AG_MR_2015_5207_LR131879
02_AG_DZ_2002_8402rt_AY828686
02AG_MR_2009_MAU635_HG518356
02AG_MR_2009_MAU1399_HG518377
02AG_TN_2014_14TN12605_KX183721
0.9902
02_AG_DZ_2002_1302rt_AY828600
02_AG_DZ_2002_1202rt_AY828598
02_AG_DZ_2003_1403rt_AY828602
0.8799
0206_MR_2009_MAU339_HG518342
0.9978
02_AG_OM_2019_HIRes-137707_PX777094
02_AG_OM_2019_HIRes-130160_PX777079
02AG_TN_2014_14TN9310_KX183799
02_AG_MA_2005_N2QAGAG_JQ316593
02_AG_MA_2005_S27AGAG_JQ316599
02_AG_MA_2013_RT11_13_KU642648
02AG_MR_2009_MAU765_HG518364
02_AG_DZ_2003_7803rt_AY828676
02AG_MR_2009_MAU1860_HG518381
02AG_KW_2015_65430_15_KX155601
0.8757
02AG_MR_2009_MAU1339_HG518376
02AG_MR_2009_MAU683_HG518360
02_AG_MA_2005_N229AGAG_JQ316592
02AG_TN_2014_14TN5066_KX183760
02AG_MR_2009_MAU471_HG518347
0.9966
02AG_MR_2015_5194_LR131870
02AG_MR_2015_5204_LR131877
02AG_MR_2015_5220_LR131887
A1G_MR_2009_MAU1247_HG518374
02AG_MR_2009_MAU1151_HG518372
02AG_MR_2015_5224_LR131889
02AG_MR_2015_5222_LR131888
A1G_MR_2009_MAU664_HG518358
02AG_MR_2015_5213_LR131883
02AG_TN_2014_14TN14566_KX183732
0.9831
02AG_TN_2021_TN-CVV44_PP483241
02AG_TN_2021_TN-CVV45_PP483242
02AG_TN_2021_TN-CVV268_PP483269
02AG_TN_2020_TN-CVV460_PP483274
0.9936
02AG_TN_2020_TN-CVV189_PP483262
02AG_TN_2021_TN-CVV57_PP483253
1
02AG_TN_2022_TN-CVV130_PP483224
02AG_TN_2020_TN-CVV98_PP483246
02AG_MR_2015_5272_LR131904
02AG_TN_2020_TN-CVV83_PP483245
0.9989
02_AG_OM_2024_HIRes-244653_PX777743
02_AG_OM_2023_HIRes-219674_PX777548
02_AG_OM_2023_HIRes-239391_PX777594

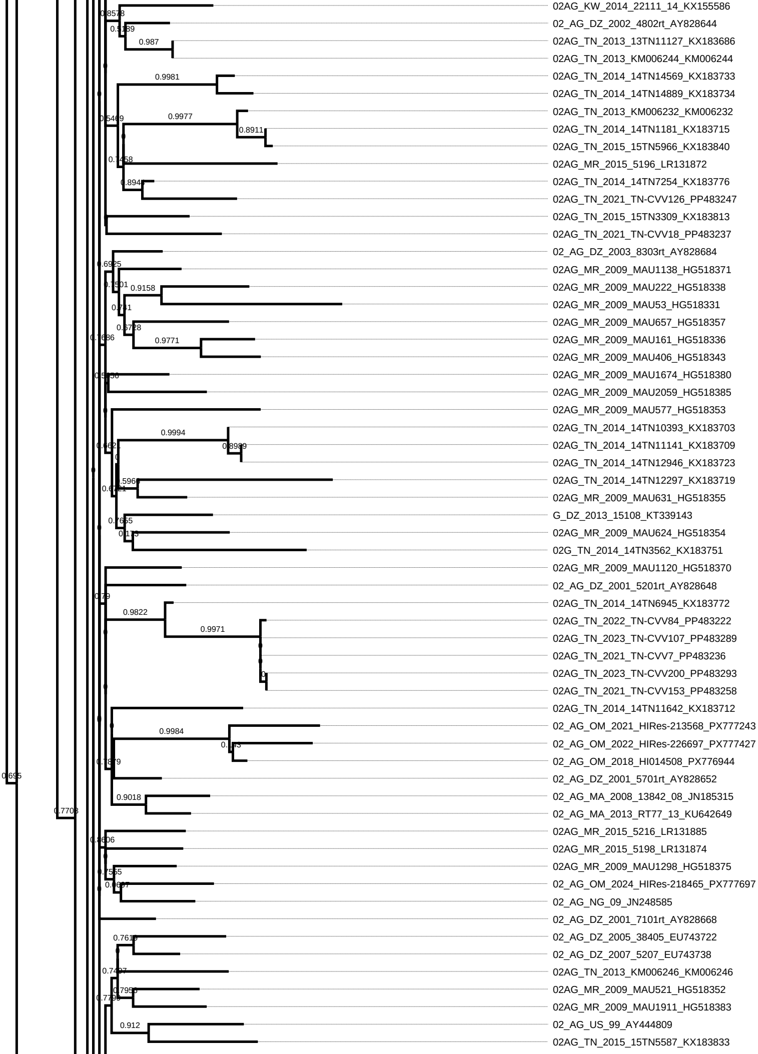
02AG_KW_2014_22111_14_KX155586
02_AG_DZ_2002_4802rt_AY828644
02AG_TN_2013_13TN11127_KX183686
02AG_TN_2013_KM006244_KM006244
02AG_TN_2014_14TN14569_KX183733
02AG_TN_2014_14TN14889_KX183734
02AG_TN_2013_KM006232_KM006232
02AG_TN_2014_14TN1181_KX183715
02AG_TN_2015_15TN5966_KX183840
02AG_MR_2015_5196_LR131872
02AG_TN_2014_14TN7254_KX183776
02AG_TN_2021_TN-CVV126_PP483247
02AG_TN_2015_15TN3309_KX183813
02AG_TN_2021_TN-CVV18_PP483237
02_AG_DZ_2003_8303rt_AY828684
02AG_MR_2009_MAU1138_HG518371
02AG_MR_2009_MAU222_HG518338
02AG_MR_2009_MAU53_HG518331
02AG_MR_2009_MAU657_HG518357
02AG_MR_2009_MAU161_HG518336
02AG_MR_2009_MAU406_HG518343
02AG_MR_2009_MAU1674_HG518380
02AG_MR_2009_MAU2059_HG518385
02AG_MR_2009_MAU577_HG518353
02AG_TN_2014_14TN10393_KX183703
02AG_TN_2014_14TN11141_KX183709
02AG_TN_2014_14TN12946_KX183723
02AG_TN_2014_14TN12297_KX183719
02AG_MR_2009_MAU631_HG518355
G_DZ_2013_15108_KT339143
02AG_MR_2009_MAU624_HG518354
02G_TN_2014_14TN3562_KX183751
02AG_MR_2009_MAU1120_HG518370
02_AG_DZ_2001_5201rt_AY828648
02AG_TN_2014_14TN6945_KX183772
02AG_TN_2022_TN-CVV84_PP483222
02AG_TN_2023_TN-CVV107_PP483289
02AG_TN_2021_TN-CVV7_PP483236
02AG_TN_2023_TN-CVV200_PP483293
02AG_TN_2021_TN-CVV153_PP483258
02AG_TN_2014_14TN11642_KX183712
02_AG_OM_2021_HIRes-213568_PX777243
02_AG_OM_2022_HIRes-226697_PX777427
02_AG_OM_2018_HI014508_PX776944
02_AG_DZ_2001_5701rt_AY828652
02_AG_MA_2008_13842_08_JN185315
02_AG_MA_2013_RT77_13_KU642649
02AG_MR_2015_5216_LR131885
02AG_MR_2015_5198_LR131874
02AG_MR_2009_MAU1298_HG518375
02_AG_OM_2024_HIRes-218465_PX777697
02_AG_NG_09_JN248585
02_AG_DZ_2001_7101rt_AY828668
02_AG_DZ_2005_38405_EU743722
02_AG_DZ_2007_5207_EU743738
02AG_TN_2013_KM006246_KM006246
02AG_MR_2009_MAU521_HG518352
02AG_MR_2009_MAU1911_HG518383
02_AG_US_99_AY444809
02AG_TN_2015_15TN5587_KX183833
0.8578
0.987
0.9981
0.9977
0.8911
0.9158
0.9771
0.9994
0.9822
0.9971
0.9984
0.9018
0.912

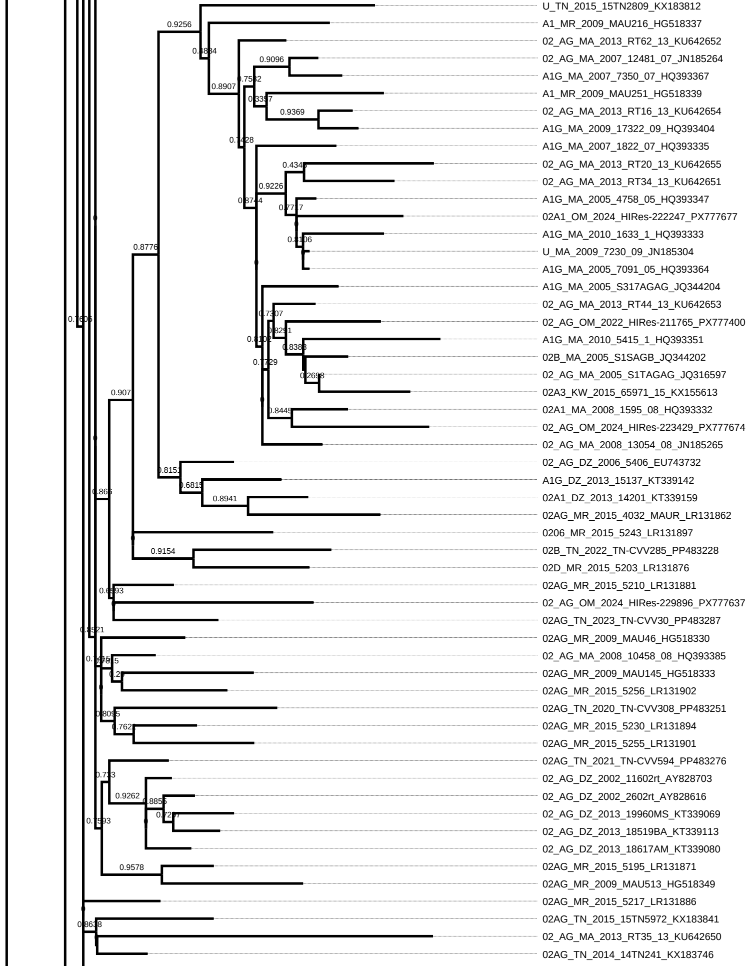
U_TN_2015_15TN2809_KX183812
A1_MR_2009_MAU216_HG518337
02_AG_MA_2013_RT62_13_KU642652
02_AG_MA_2007_12481_07_JN185264
A1G_MA_2007_7350_07_HQ393367
A1_MR_2009_MAU251_HG518339
02_AG_MA_2013_RT16_13_KU642654
A1G_MA_2009_17322_09_HQ393404
A1G_MA_2007_1822_07_HQ393335
02_AG_MA_2013_RT20_13_KU642655
02_AG_MA_2013_RT34_13_KU642651
A1G_MA_2005_4758_05_HQ393347
02A1_OM_2024_HIRes-222247_PX777677
A1G_MA_2010_1633_1_HQ393333
U_MA_2009_7230_09_JN185304
A1G_MA_2005_7091_05_HQ393364
A1G_MA_2005_S317AGAG_JQ344204
02_AG_MA_2013_RT44_13_KU642653
02_AG_OM_2022_HIRes-211765_PX777400
A1G_MA_2010_5415_1_HQ393351
02B_MA_2005_S1SAGB_JQ344202
02_AG_MA_2005_S1TAGAG_JQ316597
02A3_KW_2015_65971_15_KX155613
02A1_MA_2008_1595_08_HQ393332
02_AG_OM_2024_HIRes-223429_PX777674
02_AG_MA_2008_13054_08_JN185265
02_AG_DZ_2006_5406_EU743732
A1G_DZ_2013_15137_KT339142
02A1_DZ_2013_14201_KT339159
02AG_MR_2015_4032_MAUR_LR131862
0206_MR_2015_5243_LR131897
02B_TN_2022_TN-CVV285_PP483228
02D_MR_2015_5203_LR131876
02AG_MR_2015_5210_LR131881
02_AG_OM_2024_HIRes-229896_PX777637
02AG_TN_2023_TN-CVV30_PP483287
02AG_MR_2009_MAU46_HG518330
02_AG_MA_2008_10458_08_HQ393385
02AG_MR_2009_MAU145_HG518333
02AG_MR_2015_5256_LR131902
02AG_TN_2020_TN-CVV308_PP483251
02AG_MR_2015_5230_LR131894
02AG_MR_2015_5255_LR131901
02AG_TN_2021_TN-CVV594_PP483276
02_AG_DZ_2002_11602rt_AY828703
02_AG_DZ_2002_2602rt_AY828616
02_AG_DZ_2013_19960MS_KT339069
02_AG_DZ_2013_18519BA_KT339113
02_AG_DZ_2013_18617AM_KT339080
02AG_MR_2015_5195_LR131871
02AG_MR_2009_MAU513_HG518349
02AG_MR_2015_5217_LR131886
02AG_TN_2015_15TN5972_KX183841
02_AG_MA_2013_RT35_13_KU642650
02AG_TN_2014_14TN241_KX183746
0.9256
0.9096
0.8907
0.9369
0.9226
0.8776
0.907
0.8941
0.9154
0.9262
0.9578

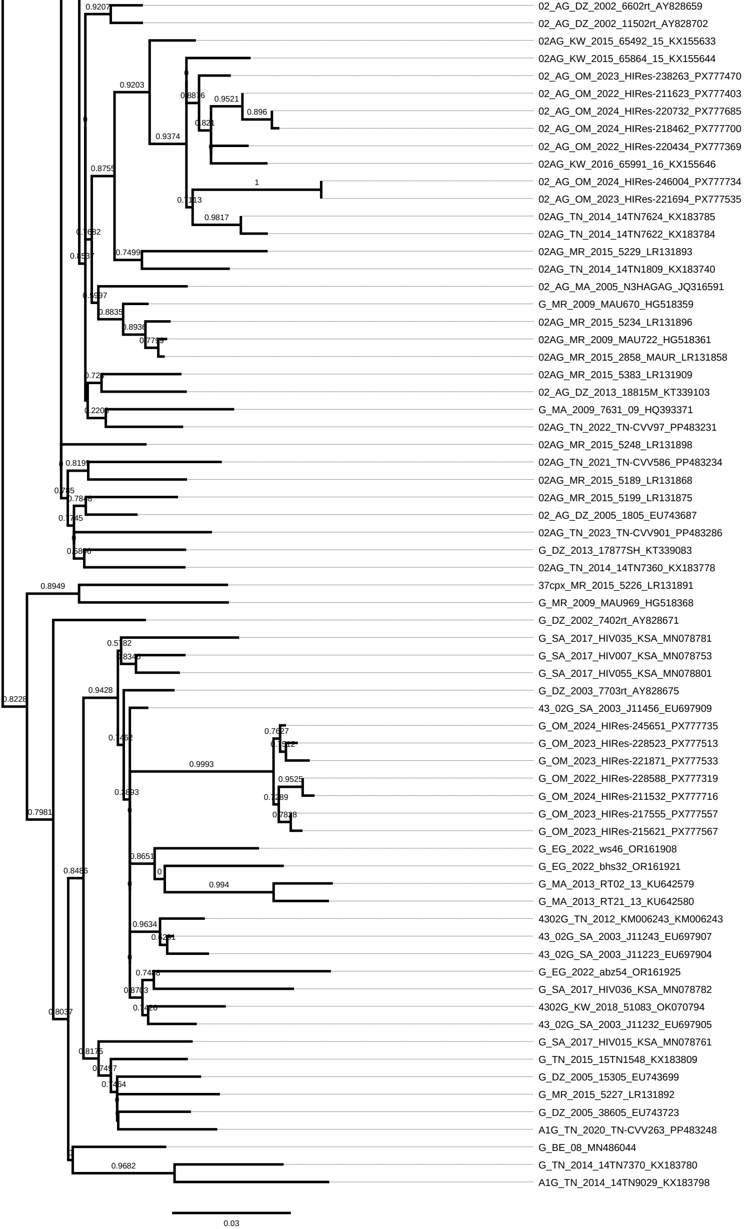
02_AG_DZ_2002_6602rt_AY828659
02_AG_DZ_2002_11502rt_AY828702
02AG_KW_2015_65492_15_KX155633
02AG_KW_2015_65864_15_KX155644
02_AG_OM_2023_HIRes-238263_PX777470
02_AG_OM_2022_HIRes-211623_PX777403
02_AG_OM_2024_HIRes-220732_PX777685
02_AG_OM_2024_HIRes-218462_PX777700
02_AG_OM_2022_HIRes-220434_PX777369
02AG_KW_2016_65991_16_KX155646
02_AG_OM_2024_HIRes-246004_PX777734
02_AG_OM_2023_HIRes-221694_PX777535
02AG_TN_2014_14TN7624_KX183785
02AG_TN_2014_14TN7622_KX183784
02AG_MR_2015_5229_LR131893
02AG_TN_2014_14TN1809_KX183740
02_AG_MA_2005_N3HAGAG_JQ316591
G_MR_2009_MAU670_HG518359
02AG_MR_2015_5234_LR131896
02AG_MR_2009_MAU722_HG518361
02AG_MR_2015_2858_MAUR_LR131858
02AG_MR_2015_5383_LR131909
02_AG_DZ_2013_18815M_KT339103
G_MA_2009_7631_09_HQ393371
02AG_TN_2022_TN-CVV97_PP483231
02AG_MR_2015_5248_LR131898
02AG_TN_2021_TN-CVV586_PP483234
02AG_MR_2015_5189_LR131868
02AG_MR_2015_5199_LR131875
02_AG_DZ_2005_1805_EU743687
02AG_TN_2023_TN-CVV901_PP483286
G_DZ_2013_17877SH_KT339083
02AG_TN_2014_14TN7360_KX183778
37cpx_MR_2015_5226_LR131891
G_MR_2009_MAU969_HG518368
G_DZ_2002_7402rt_AY828671
G_SA_2017_HIV035_KSA_MN078781
G_SA_2017_HIV007_KSA_MN078753
G_SA_2017_HIV055_KSA_MN078801
G_DZ_2003_7703rt_AY828675
43_02G_SA_2003_J11456_EU697909
G_OM_2024_HIRes-245651_PX777735
G_OM_2023_HIRes-228523_PX777513
G_OM_2023_HIRes-221871_PX777533
G_OM_2022_HIRes-228588_PX777319
G_OM_2024_HIRes-211532_PX777716
G_OM_2023_HIRes-217555_PX777557
G_OM_2023_HIRes-215621_PX777567
G_EG_2022_ws46_OR161908
G_EG_2022_bhs32_OR161921
G_MA_2013_RT02_13_KU642579
G_MA_2013_RT21_13_KU642580
4302G_TN_2012_KM006243_KM006243
43_02G_SA_2003_J11243_EU697907
43_02G_SA_2003_J11223_EU697904
G_EG_2022_abz54_OR161925
G_SA_2017_HIV036_KSA_MN078782
4302G_KW_2018_51083_OK070794
43_02G_SA_2003_J11232_EU697905
G_SA_2017_HIV015_KSA_MN078761
G_TN_2015_15TN1548_KX183809
G_DZ_2005_15305_EU743699
G_MR_2015_5227_LR131892
G_DZ_2005_38605_EU743723
A1G_TN_2020_TN-CVV263_PP483248
G_BE_08_MN486044
G_TN_2014_14TN7370_KX183780
A1G_TN_2014_14TN9029_KX183798
0.03
